# Profiles of *Plasmodium falciparum* infections detected by microscopy through the first year of life in Kintampo a high transmission area of Ghana

**DOI:** 10.1371/journal.pone.0240814

**Published:** 2020-10-19

**Authors:** Akua Kyerewaa Botwe, Seth Owusu-Agyei, Muhammad Asghar, Ulf Hammar, Felix Boakye Oppong, Stephaney Gyaase, David Dosoo, Gabriel Jakpa, Ellen Boamah, Mieks Frenken Twumasi, Faith Osier, Anna Färnert, Kwaku Poku Asante

**Affiliations:** 1 Kintampo Health Research Centre, Kintampo, Ghana; 2 Division of Infectious Diseases, Department of Medicine Solna, Karolinska Institutet, Stockholm, Sweden; 3 Kenya Medical Research Institute-Wellcome Trust Research Programme, Kilifi, Kenya; 4 Institute of Health Research, University of Health and Allied Sciences, Ho, Ghana; 5 Department of Infectious Diseases, Karolinska University Hospital, Stockholm, Sweden; 6 Center for Molecular Medicine, Karolinska Institutet, Stockholm, Sweden; 7 Unit of Biostatistics, Department of Epidemiology, Institute for Environmental Medicine, Karolinska Institutet, Stockholm, Sweden; 8 Heidelberg University Hospital, Heidelberg, Germany; Instituto Rene Rachou, BRAZIL

## Abstract

Although malaria mortality among children under five years of age is high, the characteristics of their infection patterns are not well described. The aim of this study was to examine the longitudinal sequence pattern of *Plasmodium falciparum* infections in the first year of life within a birth cohort in Kintampo, Ghana (N = 1855). Infants were monitored at home with monthly sampling and also at the clinic for any febrile illness between 2008 and 2011. Light microscopy was performed on monthly scheduled visits and febrile ill visits over twelve months of follow-ups (n = 19231). Microscopy-positive visits accompanied with or without symptoms were rare during the first five months of life but were common from six to twelve months of age. Among 1264 infants with microscopy data over a minimum of eight monthly visits and also throughout in sick visits, some were microscopy negative (36%), and others positive: only-symptomatic (35%), alternating (22%) and only-asymptomatic (7%). The median age of microscopic infection was seven months for the alternating group and eight months for both the only-symptomatic and only-asymptomatic groups. The alternating group had the highest cumulative incidence of microscopic infections, the lowest age at first infection and 87 different infection patterns. Parasite densities detected by microscopy were significantly higher for symptomatic versus asymptomatic infection. We conclude that infants in malaria endemic areas experience diverse infection profiles throughout their first year of life. Further investigations should include submicroscopic reservoir and may shed more light on the factors that determine susceptibility to malaria during infancy.

## Introduction

*Plasmodium falciparum* malaria is often uncomplicated, but can also be fatal, and children below the age of five are most vulnerable [1, 2]. Malaria parasite infections may be accompanied with or without symptoms (asymptomatic) in semi immune individuals in endemic areas [3–5]. Symptomatic malaria has been reported to occur less frequently than asymptomatic infections in both high and low transmission settings [4, 6]. However among infants, symptomatic malaria is considered to be a major public health problem. Symptomatic malaria in this age-group is relatively well-studied and yet asymptomatic infections may be informative for studies of immunity and accelerate efforts to eliminate malaria [1, 7].

Infants living in endemic areas experience both symptomatic malaria and asymptomatic infections [8, 9]. During the first six months of life, infections are reported to be mainly asymptomatic, while between six and twelve months of age the incidence of both symptomatic malaria and asymptomatic infections tend to increase [8, 10–13]. The low incidence of symptomatic malaria below the age of six months has been attributed to the presence of fetal hemoglobin [14, 15] and passively acquired maternal IgG [5]. Malaria-specific antibodies at birth (in maternal and/or cord blood) have also been associated with protection against some malaria parasite antigens [5, 16–20]. Nevertheless, detailed descriptions of the longitudinal patterns of symptomatic malaria and asymptomatic infections within the first year of life are not available, as most research focuses primarily on the direct effects of clinical symptoms [10, 20–25].

Studying the temporal patterns of both symptomatic malaria and asymptomatic infections in infants is important for understanding the relationships between susceptibility and immunity, and for guiding control interventions such as presumptive treatment and vaccines. Studying large cohorts of infants who undergo frequent sampling over longer periods of time is also useful for exploring correlates of protection [7]. Previous studies assessing malaria morbidity in the first year of life either had limited sampling opportunities [9, 21] or had relatively small cohorts [5, 18]. Consequently, the longitudinal profiles and age-specific patterns of infections during the first year of life have not been extensively studied and the potential correlates of protection during infancy remain uncertain [20, 25].

Ghana is currently considered by the World Health Organizations (WHO) to be one of the eleven countries with the highest burden of malaria in the world as the malaria status in 2010 is the same as in 2018 and the malaria-related infant mortality is still high [1]. We conducted a large birth cohort study in a high transmission area of Ghana to determine whether exposure to placental malaria increased the risk of malaria during infancy [26]. To detect malaria parasites, microscopy was performed on blood samples collected during scheduled monthly home visits and whenever an infant reported to the study hospital with an illness [26]. Here, we report the age-specific patterns of microscopy positivity with or without symptoms, among 1819 infants with in total 19231 visits in the first year of life.

## Materials and methods

### Study location and population

**Fig 1:**
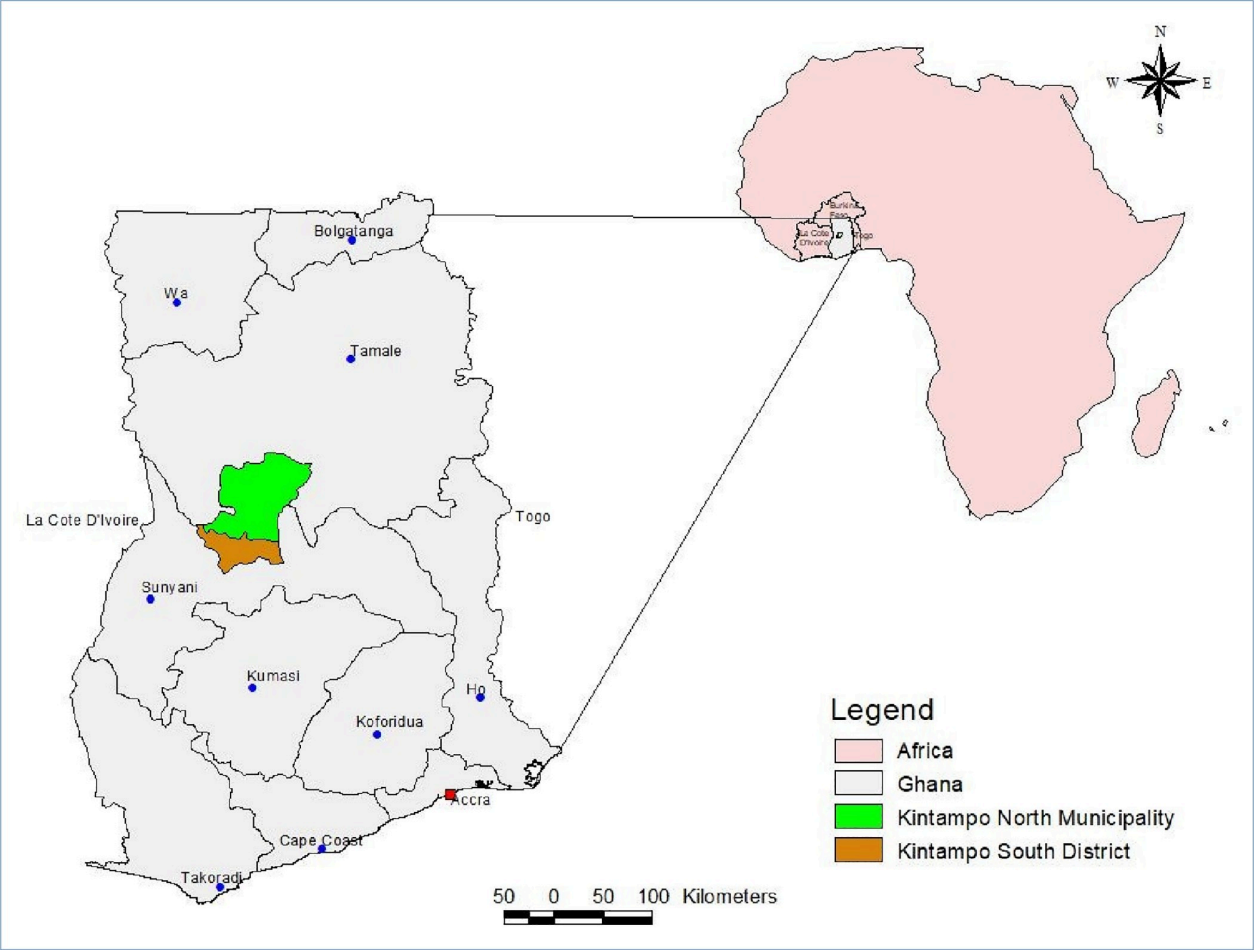
Map of study location. Credit: Kintampo Health Research Centre Geographic Information System Unit (2020).

Kintampo North Municipality (KNM) and Kintampo South District (KSD) comprises 156 communities and covers an area of 7 162 km^2^ in Ghana (Fig 1). Malaria transmission in this area is high and perennial, peaking during the rainy season between April and November [27, 28].

In October 2008, a birth cohort study enrolled pregnant women in the study area [26]. Infants were included in the study at birth between November 2008 and January 2011. They were followed up by scheduled home visits every month until twelve months of age or until the end of the study in May 2011. The duration of infant follow-up varied over the study period, as they were enrolled at birth. Infants also exited the study at various times due to death, migration or loss to follow-up [26].

### Collection of samples and identification of parasites

Each child was visited at home on a monthly basis, by trained field staff who collected blood samples and completed standard questionnaires. Caregivers were encouraged to seek care from the study clinician, and for the infant’s blood to be taken if the infant was unwell, in-between the home visits. During the clinical visits, infants with reported fever or axillary temperature ≥ 37.5°C (elevated temperature) and malaria parasites detected by microscopy were treated with artesunate/amodiaquine in accordance with the national health policies. All infants received free health insurance, which was paid for by the study.

Blood samples (500 μl– 1 ml) were collected into EDTA vacutainer blood-collection tubes during the scheduled/home visits and when malaria was suspected at the unscheduled/clinical visits. Malaria parasites were examined by Giemsa-stained thin and thick blood films. Parasite densities were estimated per microlitre (μl) of blood, assuming 8000 leukocytes/μl of blood, counting the number of parasites per 200 leukocytes and multiplying the leukocyte fraction by 40. Parasites were counted per 500 leukocytes and multiplied by 16 if the parasitaemia was less than 100 per μl. If the infant had temperature below 37.5°C during the unscheduled/clinical visit and the caregiver did not report fever in the previous 48 hours, the clinician did not suspect malaria and did not request a microscopy test. Thus, there were unscheduled/clinical visits with no accompanying microscopy tests. Consequently, unscheduled/clinical visits in subsequent seven days after parasite detection on a scheduled/home visit were factored into the definition of symptomatic malaria. Insecticide-treated bed nets were used to prevent malaria among infants. Details including enrolment and follow-up visits have been previously described [26].

### Quality control

Microscopists were certified experts by the National Institute for Communicable Diseases (NICD), South Africa and the United Kingdom National External Quality Assessment Service (UKNEQAS). The NICD microscopy certification was performed three times in each year of the study while the UKNEQAS certification was conducted monthly. In addition, 10% of the blood films were examined by an independent expert microscopist who attained 100% agreement with the other microscopists.

### Definitions of symptomatic malaria and asymptomatic infection

At either the scheduled/home or unscheduled/clinical visit, symptomatic malaria (adapted from the WHO) was defined as a positive malaria microscopy reading together with a history of reported fever or elevated temperature and illnesses/symptoms (vomiting, chills, fever, diarrhoea, cough, difficulty breathing, blood in urine or inability to suckle, drink or eat) within the past 48 hours or subsequent seven days of parasite detection [1, 29]. An asymptomatic infection was defined as positive malaria microscopy tests at the monthly scheduled/home visits with temperature < 37.5°C and neither reported fever, illnesses/without a clinical sign of malaria nor unscheduled/clinical visits in the preceding 48 hours or subsequent seven days following parasite detection.

### Statistical analyses and profiling of temporal infections

Data was analyzed using Stata Version 14 (STATA Corporation, College Station, TX) and JMP statistical software. For a detailed description of the dynamics of infection status in visits where microscopy was positive throughout the first year of life (temporal infections), we identified infants who had microscopy tests for eight or more scheduled/home visits and any number of unscheduled/clinical visits (S1 Fig). The scheduled/home and unscheduled/clinical visits that had missing parasite density values, measured temperature or reported fever in past 48 hours were excluded as these were necessary for the longitudinal classifications (Table 1). The presence of malaria parasites detected by microscopy, in addition to measured temperature and/or history of fever and illnesses at scheduled/home or unscheduled/clinical visits were used to determine symptomatic malaria and asymptomatic infections and to group the longitudinal sequence pattern of infections as: i) “parasite negative” if an infant did not have malaria parasites by microscopy during all follow up visits, ii) “only-symptomatic” if an infant had symptomatic malaria on any occasion parasites were detected by microscopy. iii) “only-asymptomatic” if an infant was asymptomatic when parasites were detected by microscopy on any scheduled/home visit, and iv) “alternating” if both asymptomatic infection and symptomatic malaria were intermittently detected by microscopy, in no specific order, throughout all follow up visits in the first year of life.

**Table 1:** Characteristics of infants and visits.

Characteristic	Level	Scheduled/home visits		Unscheduled/clinical visits*	
		Value	Proportion (%)	Value	Proportion (%)
**Number of births**	All	1819	100	-	-
**Sex**	Male	928	51.0	-	-
	Female	891	49.0	-	-
**Transmission season at birth†**	High	1308	71.9		
	Low	511	28.1	-	-
**Infants visited at least once**	Yes	1819	100	1488	81.8
	No	-	-	331	18.2
**Infants having microscopy records**	Yes	1674	92.0	1437	96.6
	No	145	8.0	51	3.4
**Infants having ≥ 8 microscopy records/visits**	Yes	1264	69.5	1177	93.0
	No	0	0	87	7.0
**Number of visits for 1819 infants**		19231	78.5	5 254	21.5
**Expected scheduled/home visits for 1819 infants**		21828	88.1	-	-

*Scheduled/home visit (and sampling) during which infants were found to be febrile and taken to hospital were regarded as unscheduled/clinical visit/sample, thus these scheduled/home visits and samples were recorded as unavailable/missing. Microscopy tests were not requested during clinical visit if malaria was not suspected.

†High transmission is from April to November and low from December to March.

The frequency distribution mean, cumulative incidence, age and median parasite density at infection were described for infants in the entire cohort as well as the sub-groups defined above. To explore the removal-time by microscopy for each group, the time (in days) intervals between any two infections were examined for infants with two or more infections.

The Kruskal Wallis test was used to assess age, infection and parasite density differences between groups, while the Wilcoxon signed-rank tests were performed for within group analysis. In order to control the skewness and make the analysis more valid, parasite densities were log10 transformed. Estimates with p-values < 0.05 were considered statistically significant.

### Ethical approval and consent to participate

This study was granted ethical approval by the Kintampo Health Research Centre (KHRC) Institutional Ethics Committee (Office for Human Research Protections federal wide assurance number is 00011103 and Institutional Research Board registration number is 0004854). Additional approval for this study was granted by the Regional Ethics Board of Stockholm (Dnr 2018/1967-32). Ethical approvals for the original birth cohort study were provided by the KHRC, Ghana Health Service and London School of Hygiene and Tropical Medicine. Mothers gave written consent to participate in the birth cohort study for themselves and their infants.

## Results

### Characteristics of the cohort

**Fig 2:**
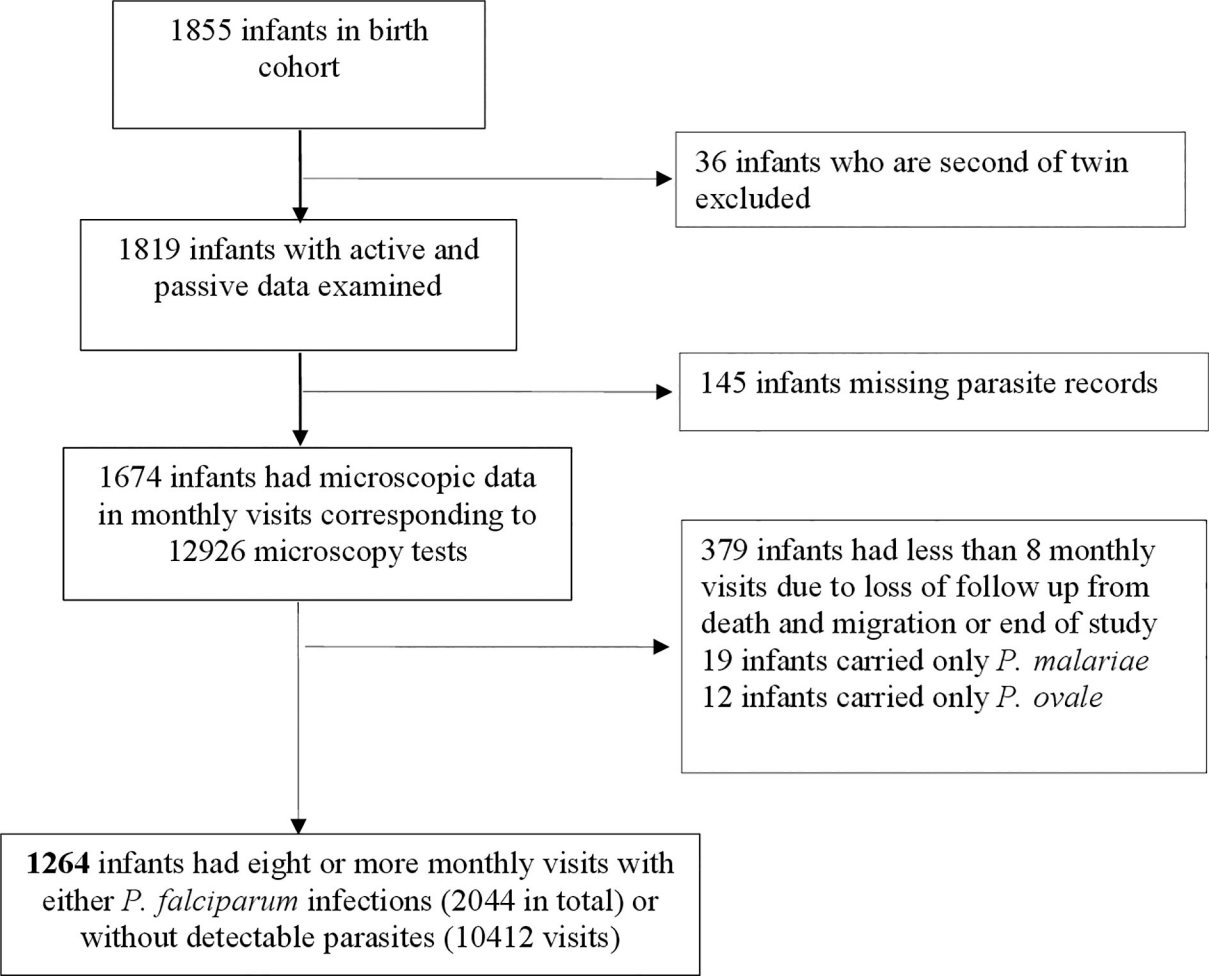
Flow chart of the selection of infants to profile the temporal infections.

The birth cohort included 1855 infants. The second child of 36 twin births was excluded in order to prevent clustering effects during analysis (Fig 2). The 1819 infants included in the analysis had a total of 19231 monthly scheduled/home visits, corresponding to a compliance rate (observed visits/expected visits × 100) of 88.1% (Table 1). For infants who reported illness between any two successive scheduled/home visits, 73.7% (1341/1819) had a corresponding unscheduled/clinical visit record.

**Fig 3:**
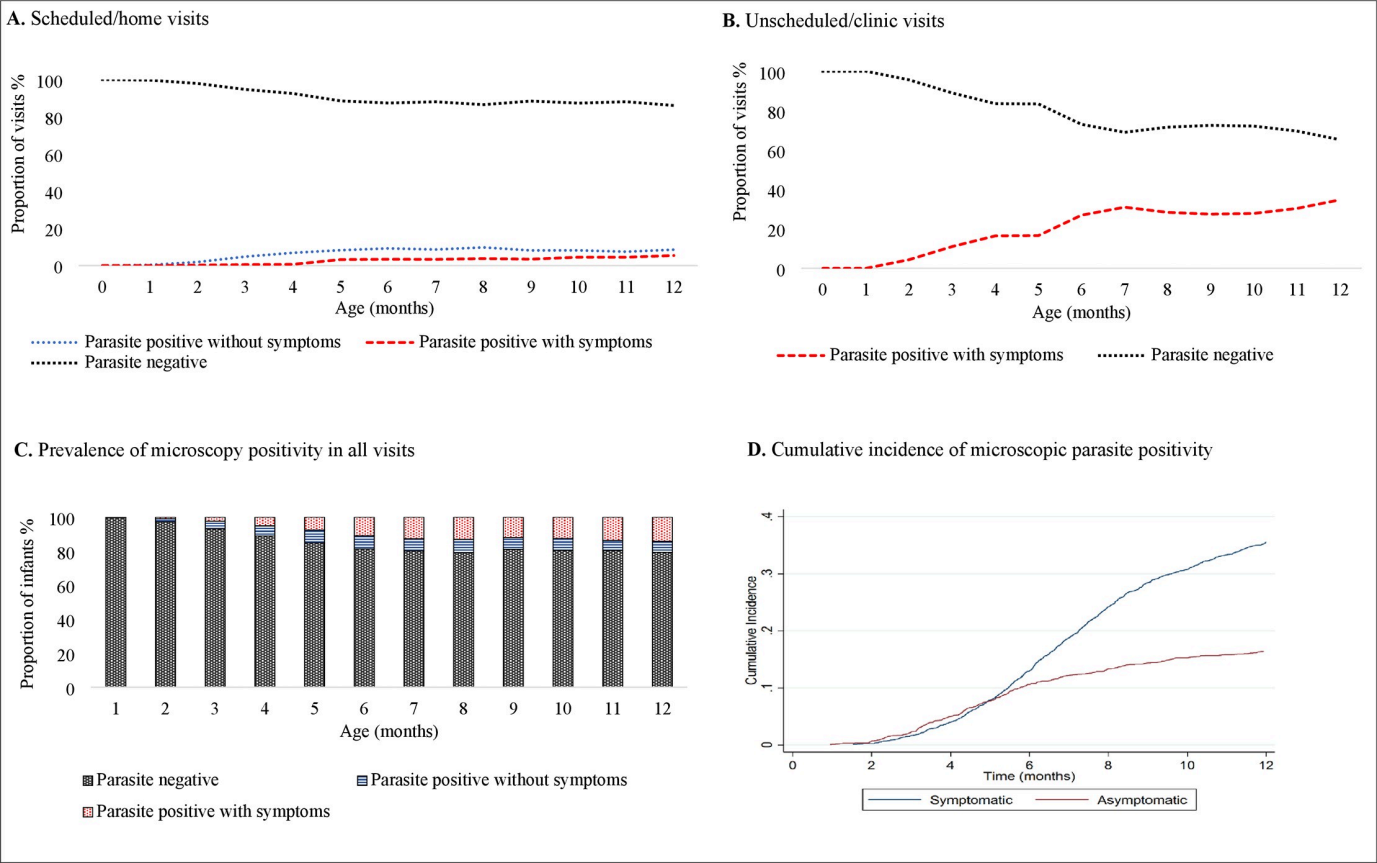
Distribution of malaria parasite negative and positive microscopy tests among 1674 infants. (A) Parasite negative and positive outcomes (with and without symptoms) at scheduled monthly home visits. (B) Microscopy negative and positive outcomes during symptomatic unscheduled hospital visits. (C) Monthly prevalence of microscopy positive visits with and without symptoms. (D) The cumulative incidence shows the rate of new microscopy positivity with or without symptoms in visits from birth to one year of age.

Malaria microscopy tests were performed for 92.0% (1674/1819) of infants during their scheduled/home and/or unscheduled/clinical visits (Table 1 and Fig 2). The infants had a mean of 6 (SD: ± 3) monthly scheduled visits with microscopy data. A summary of the microscopy data for infants is in S1 Fig, while the details of sex, month of birth and the infection status in scheduled/home or unscheduled/hospital visits is in S1 Table. The proportion of parasite positive tests, both among scheduled and unscheduled visits, increased with age (Fig 3A and 3B). Malaria parasites were detected by microscopy among 58.0% (971/1674) of infants at least once during the follow-up period. In total, 54.9% (919/1674) of infants had malaria parasites and corresponding values of measured temperature and/or history of fever in the past 48 hours and illnesses during their scheduled/home and unscheduled/clinical visits.

### Malaria microscopy positivity

Out of a total of 12926 microscopy tests performed for 1674 infants, 2394 (18.5%) tests, among 971 infants, were positive for malaria parasites. The majority of infections were *P*. *falciparum* (95.8%; 2293/2394), followed by *P*. *malariae* (2%; 41/2394) and *P*. *ovale* (1.4%; 33/2394). *Plasmodium vivax* and *P*. *knowlesi* were not identified. Mixed species infections were rare (1.1%; 27/2394) and were mainly *P*. *falciparum* and *P*. *malariae* co-infections (70.3%; 19/27). Gametocytes were detected in 1.6% (39/2394) of all infections. *P*. *falciparum* contributed 84.6% (33/39), *P*. *malariae* contributed 5.1% (2/39) and *P*. *ovale* contributed 10.3% (4/39) of all gametocytes.

Of the 2394 microscopy positive tests, 2194 (91.6%) tests among 919 infants were with corresponding values for measured temperature, history of fever in the past 48 hours and illnesses at scheduled/home and unscheduled/clinical visits. Of these, 61.1% (1340/2194) were accompanied by malaria symptoms; 68.1% (913/1340) of the symptomatic malaria infection were detected during unscheduled/clinical visits while 31.9% (427/1340) were detected during the scheduled/home visits. Microscopy positivity, both with (symptomatic malaria) and without symptoms (asymptomatic infections), increased with age in scheduled/home and unscheduled/clinical visits (Fig 3A and 3B).

Microscopy positivity increased from birth to twelve months of age and was highest at seven months of age (20%, 261 microscopy positive infants / 1314 infants tested). The microscopy detected infections were predominantly without symptoms (asymptomatic) from birth to five months of age. From six to twelve months of age, visits in which both symptomatic and asymptomatic microscopic infections were identified became more frequent among the infants (Fig 3C). This trend was also reflected in the cumulative incidence of visits in which microscopic infections were with or without symptoms, up to five months of age, after which most microscopy positivity were symptomatic (Fig 3D). The median *P*. *falciparum* parasite density was significantly lower for both symptomatic and asymptomatic infections from birth to five months of age compared with six months of age and above (S2 Fig).

### Temporal profiles of microscopy positivity in relation to symptoms

Infants with at least eight scheduled home visits with microscopy and clinical data were included in the longitudinal profile over the first year of life. Among these 1264 infants, four main groups were identified: i) 459 (36%) infants were microscopy negative at all visits (both scheduled and unscheduled) (“parasite negative” group), ii) 444 (35%) infants had symptoms when parasites were detected by microscopy (symptomatic malaria) in both scheduled and unscheduled visits (“only-symptomatic” group), iii) 87 (7%) did not have symptoms (asymptomatic infection) when parasites were detected by microscopy in the scheduled visits (“only-asymptomatic” group), and iv) 274 (22%) infants had both symptomatic malaria and asymptomatic microscopy infection in scheduled or unscheduled visits (“alternating” group).

**Fig 4:**
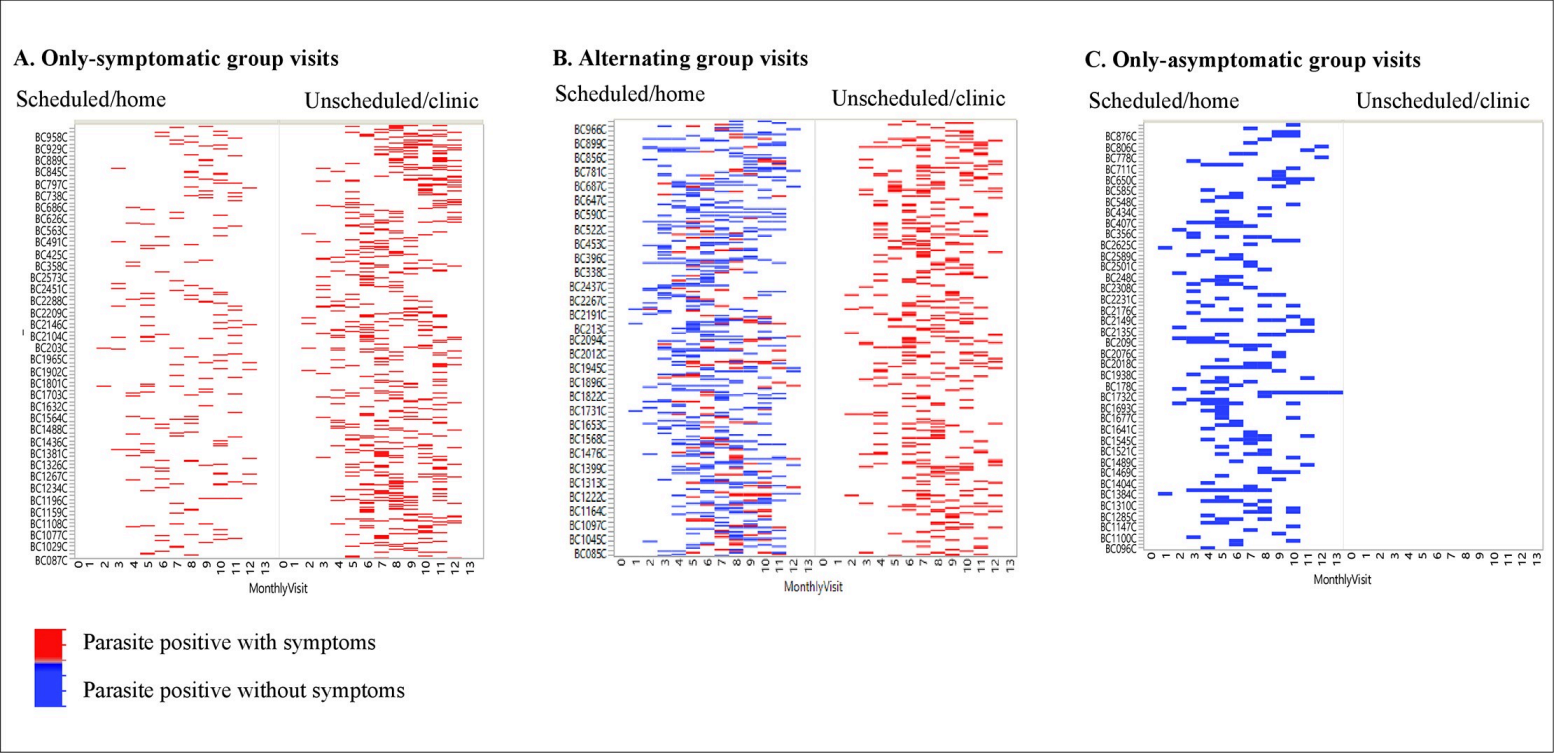
Temporal distribution of microscopic infections through the first year of life during scheduled/home or unscheduled/clinic visits. During both scheduled and unscheduled visits (A) symptomatic malaria was detected by microscopy within the only-symptomatic group, (B) symptomatic malaria and asymptomatic infections were detected by microscopy among infants in the alternating group and (C) Infants in the only-asymptomatic group were identified at the scheduled/home visits and none had unscheduled/hospital visit or symptoms in the past 48 hours of parasite detection or in the subsequent 7 days following infection.

In the group of infants with malaria parasites detected by microscopy, the infections occurred intermittently throughout the year (Fig 4A, 4B and 4C). Microscopic infections among infants in the only-asymptomatic group were identified only during scheduled/home visits (Fig 4C), unlike in other groups where microscopic infections were identified during both the scheduled/home and unscheduled/clinical visits (Fig 4A and 4B).

**Fig 5:**
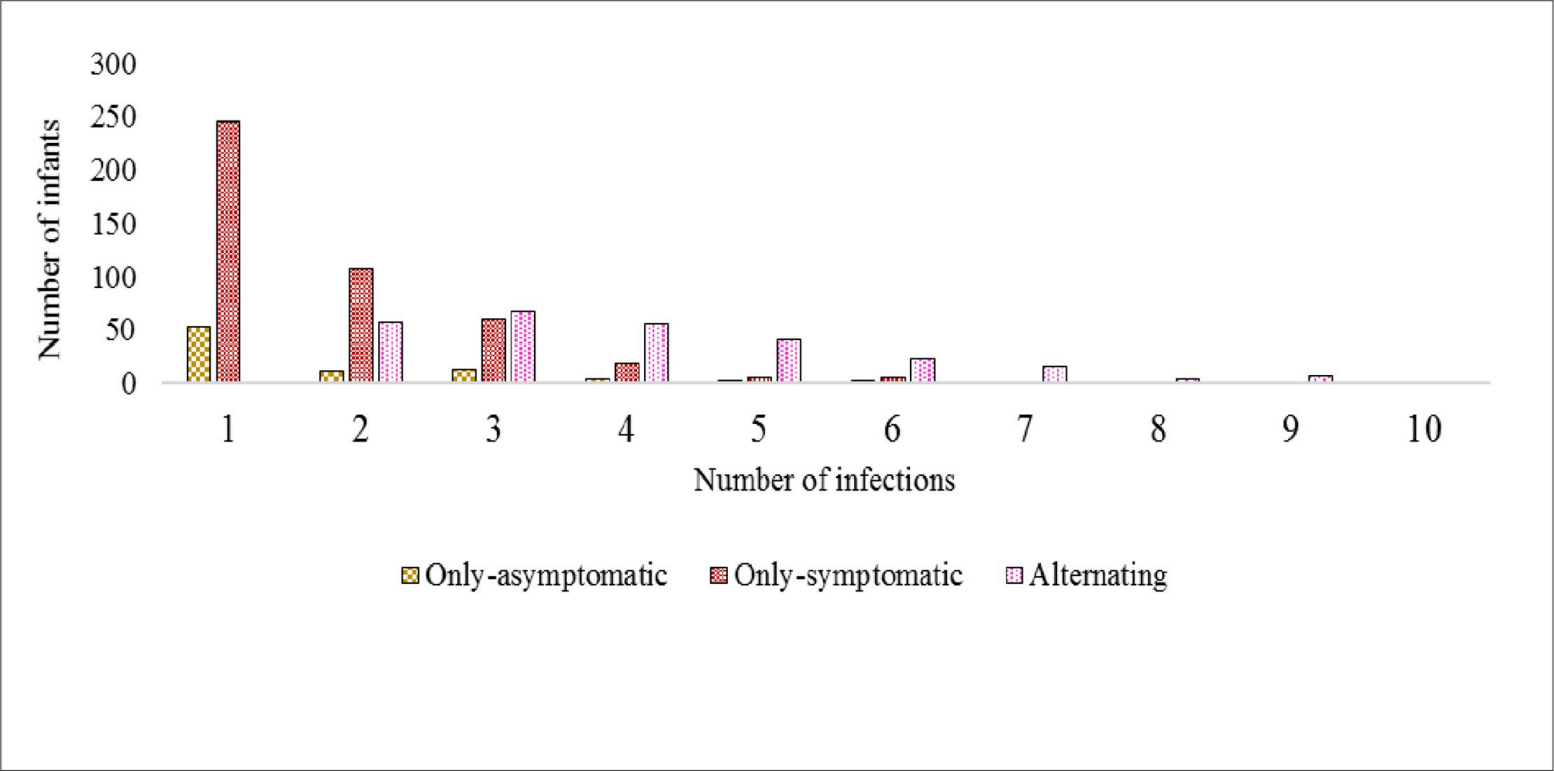
Overall microscopy positivity per group of infants. The number of times parasites were detected in the visits where microscopy was positive in each group is shown. S4 Fig shows the microscopy positive, negative or unavailable/missing visits/samples among the cohort for the only-symptomatic, alternating and only-asymptomatic groups.

The overall infection profile showed that malaria parasites were detected by microscopy at least once during both the scheduled/home and unscheduled/clinical visits among 64% (805/1264) of infants in this sub cohort. Among the infants with parasites, 37% (299/805) had only one microscopy positive visit over the twelve-months follow-up and were in either the only-symptomatic or only-asymptomatic groups (Fig 5). Visits where microscopy was positive are distinguished from the negative or unavailable/missing visits/samples in S4 Fig.

Among the infants with microscopy positive visits, 63% (506/805) had two or more separate microscopic infections over the one-year period. Of these, 54% (274/506) had both symptomatic and asymptomatic microscopic infections and were in the alternating group, 39% (198/506) were in the only-symptomatic group and 7% (34/506) were in the only-asymptomatic group.

**Fig 6:**
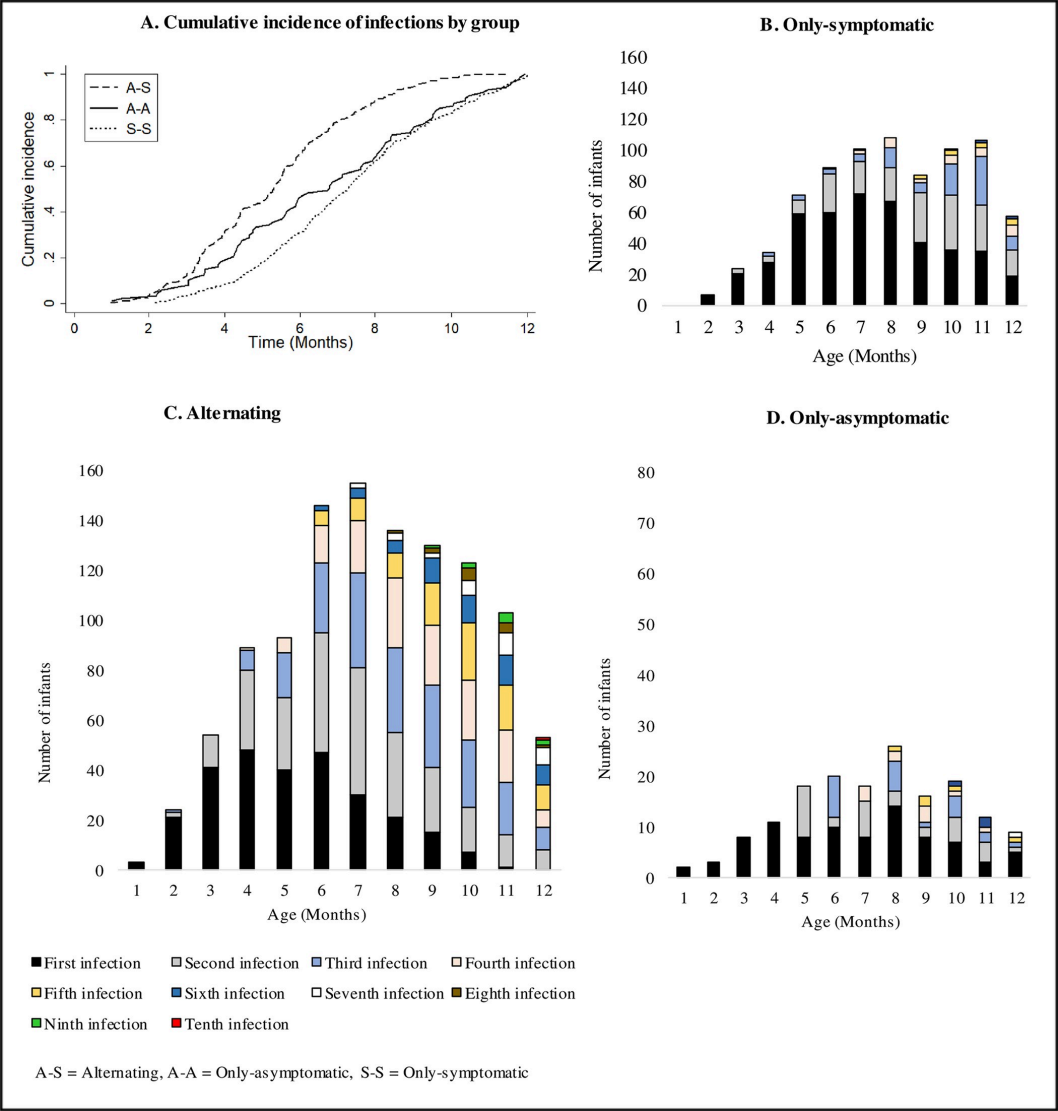
Age specific number of microscopic infections from birth to twelve months of age. (A) The cumulative incidence of microscopic infections shows the rate at which new microscopy positive visits occurred in each infection profile. The monthly breakdown of microscopy positivity shows the number of infants having (B) microscopic infection accompanied with symptoms within the only-symptomatic group, (C) microscopic infections with or without symptoms within the alternating group and (D) microscopic infection without symptoms within the only-asymptomatic group.

The cumulative incidence of microscopic infections from birth to twelve months of age was highest in the alternating group, followed by the only-asymptomatic group (Fig 6A). The cumulative incidence of microscopy positivity was similar between the only-asymptomatic group and the only-symptomatic group from eight months of age onwards (Fig 6A).

The mean number of microscopy positive visits was similar between the only symptomatic [1.75 visits/child (779/444)] and only-asymptomatic groups [1.85 visits/child (161/87)] (p = 0.715). Within the alternating group, the mean number of microscopy positive visits that were with symptoms was 2.17 infections/child (595/274) and without symptoms was 1.86 infections/child (509/274) (p < 0.001).

When comparing infants with two or more microscopic infections, the alternating group had the highest mean number of infections [4.03 infections/child (1104/274)] compared to the only-symptomatic [2.69 infections/child (533/198)] and only-asymptomatic groups [3.18 infections/child (108/34)] (p = 0.001).

Analysis of the number as well as the sequence patterns of microscopic infections in the alternating group showed 87 different sequences of symptomatic malaria (S) and asymptomatic microscopic infections (A) (Table 2). Half (59%; 51/87) of the sequence patterns of alternating symptomatic malaria and asymptomatic microscopic infections were unique to individuals (Table 2).

**Table 2:** The sequence patterns of microscopic positive visits among 805 infants.

**Only-symptomatic**		**Alternating patterns with symptomatic first infections**							
**One sequence pattern**	**Number of infants (N = 444)**	**Two sequence pattern**	**Number of infants (N = 51)**	**Three sequence pattern**	**Number of infants (N = 42)**	**Four sequence pattern**	**Number of infants (N = 7)**	**≥5 sequence pattern**	**Number of infants (N = 7)**
S	246	SA	16	SAS	7	SASA	2	**5 sequence pattern**	
SS	107	SAA	8	SASS	4	SASAA	1	SASASS	2
SSS	61	SSA	8	SSAS	7	SSASA	1	SASSSAS	1
SSSS	19	SAAA	4	SAAS	5	SSSASA	1	SAASSASS	1
SSSSS	6	SSAA	4	SASSS	2	SASSSSSA	1		
SSSSSS	5	SSSA	3	SAAAS	2	SAAASSAA	1	**6 sequence pattern**	
		SSSSA	2	SAASS	3			SASASA	1
		SAAAA	2	SSSAS	3			SSSASASSA	1
		SSSSAA	1	SSAAS	2				
		SSSAAA	1	SSASS	1			**7 sequence pattern**	
		SSSSAAA	1	SSSASS	1			SSASASASS	1
		SAAAAAA	1	SAAASS	1				
				SSASSS	1				
				SAAAASS	1				
				SSSSSSSAS	1				
				SASSSSSSSS	1				
**Only-asymptomatic**		**Alternating patterns with asymptomatic first infections**							
**One sequence pattern**	**Number of infants (N = 87)**	**Two sequence pattern**	**Number of infants (N = 125)**	**Three sequence pattern**	**Number of infants (N = 19)**	**Four sequence pattern**	**Number of infants (N = 15)**	**≥5 sequence pattern**	**Number of infants (N = 8)**
A	53	AS	42	ASA	5	ASAS	2	**5 sequence pattern**	
AA	12	ASS	23	ASSA	4	AASAS	2	ASASA	2
AAA	13	AAS	17	AASA	1	ASAAS	1	AASASA	1
AAAA	4	ASSS	8	AASAA	1	ASSASS	2	ASASSSA	1
AAAAA	2	AASS	7	ASSAA	1	AAASAS	1	ASASSAA	1
AAAAAA	2	AAAS	5	ASAAA	1	ASASSS	1	AAASSASAA	1
AAAAAAA	1	ASSSS	6	AAASSA	1	AASSASS	1		
		AAAAS	4	AAAASA	1	ASSSAAS	1	**6 sequence pattern**	
		AAASS	2	ASSSAA	1	ASAAASS	1	ASASSAASS	1
		AASSS	2	AAAAASA	1	ASSASSS	1		
		AAAAAS	3	AASAAAA	1	AAAASAS	1	**8 sequence pattern**	
		ASSSSS	1	ASSSSSSA	1	AAASSSASS	1	ASASAASAS	1
		AASSSS	1						
		AAASSS	1						
		ASSSSSS	2						
		AASSSSS	1						

S = symptomatic malaria and A = asymptomatic infection. Upper table = Infants in the only-symptomatic group (have a maximum of 6 intermittent symptomatic malaria) and alternating group who first experienced a symptomatic episode (have a maximum of 10 intermittent infections). Lower table = Infants in only-asymptomatic group (have a maximum of 7 intermittent asymptomatic infections) and alternating group who first experienced an asymptomatic infection (have a maximum of 9 intermittent infections). More details on time at infection are available in S1 Table.

An analysis of the number of visits in which microscopy positivity was with or without symptoms within the alternating group showed a maximum of ten intermittent microscopic infections, while three microscopic infections were frequent [observed among 25% (68/274) of infants in six different sequence patterns] (Table 2). For the most common alternating sequence pattern, the first microscopy positivity was identified in visits without symptoms (asymptomatic) that were followed by other microscopic infections where the visits were symptomatic (46%; 125/274) (Table 2).

The time span (removal time) between any two visits in which microscopy was positive peaked between 27 and 38 days for all groups. Visits in which microscopy positivity were six months (180 days) or more apart were rarely observed in all groups, while positivity which were less than 27 days apart were rare in the only-asymptomatic group (S3 Fig).

### Age at infection among groups of infants

The first visits in which microscopy was found positive were between one and twelve months of age [median = 6 months, (IQR: 5–8)]. The age at first visit where microscopy was positive was lowest in the alternating group [median = 5 months (IQR: 4–7)] compared to the only-asymptomatic group [median = 7 months (IQR: 4–9)] or the only-symptomatic group [median = 7 months (IQR: 5–9)] (all p < 0.001).

Whereas the first symptomatic malaria episodes were detected between two and twelve months of age for infants in the only-symptomatic group (Fig 6B), the first microscopic infections were detected between one and twelve months of age for infants in the only-asymptomatic group (Fig 6D). In the alternating group, the first microscopic infections occurred between one and eleven months of age (Fig 6C) and the first microscopic infections which were without symptoms [61% (167/274) of infants] were more frequent than the first microscopic infections which were with symptoms [39% (107/274) of infants] (p = 0.003).

Although microscopic infections were detected at all ages, a relatively small proportion of infants (5%; 36/805) had visits in which microscopy was positive at two months of age and were mainly in the alternating group [69% (25/36)]. For all the groups with malaria parasites, the number of times that infants had visits in which microscopy was positive (on separate occasions) increased with age, but the proportion of infected infants decreased with age (Fig 6B, 6C and 6D). The median age of microscopic infection was seven months (IQR: 6–10) for the alternating group and eight months for both only-asymptomatic (IQR: 5–9) and only-symptomatic groups (IQR: 6–10).

### Parasite densities among groups of infants

**Fig 7:**
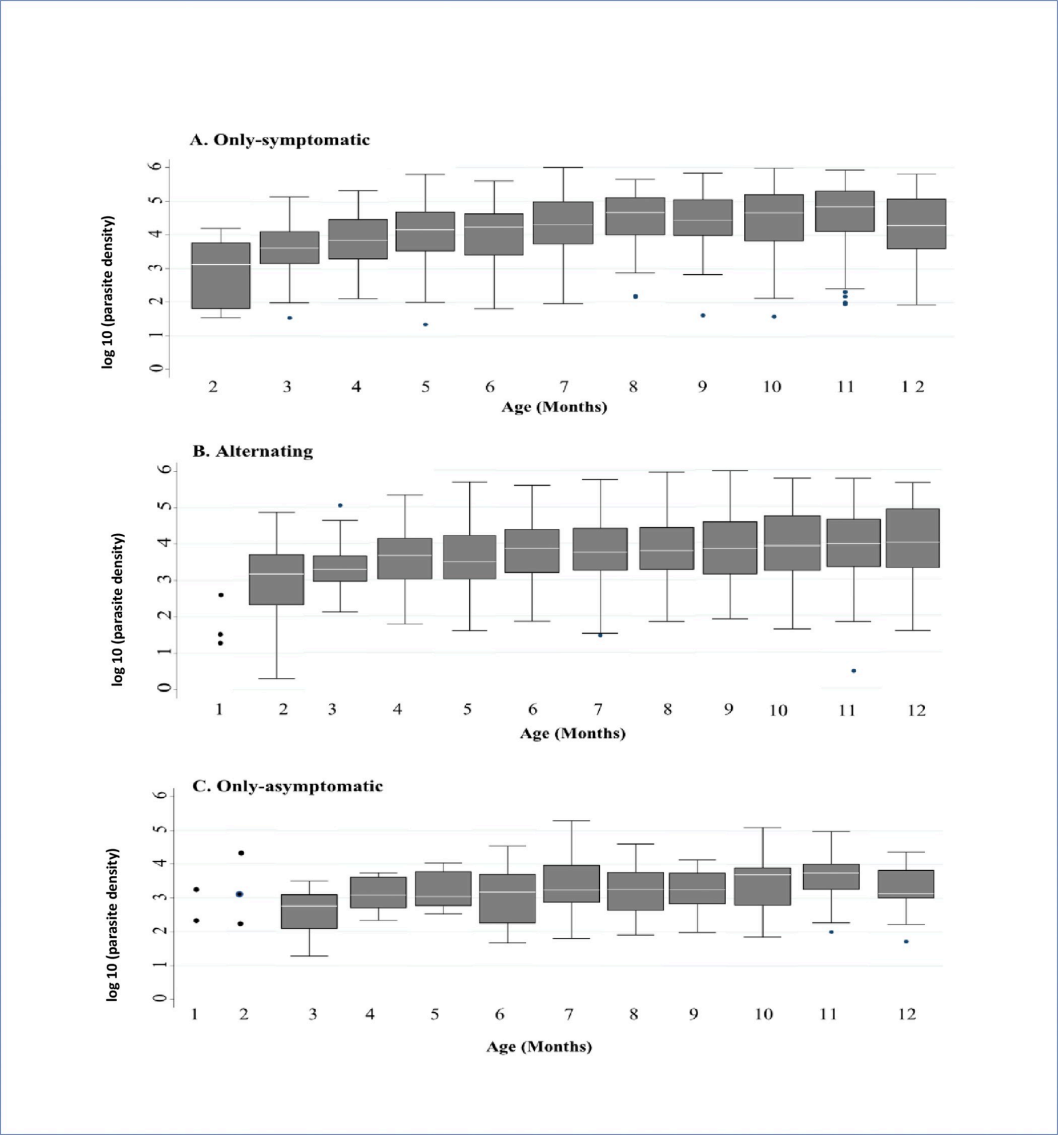
Age specific distribution of parasite densities through the first year of life. Monthly median parasite densities corresponding with (A) symptomatic malaria within the only-symptomatic group, (B) both asymptomatic infections and symptomatic malaria within the alternating group and (C) asymptomatic infections within the only-asymptomatic group.

The overall range of asexual *P*. *falciparum* parasite density was 2–974759 parasite per μl. Parasite density increased with age in all the groups (Fig 7), and median parasite density at first infection was highest in the only-symptomatic group (p < 0.001) (Table 3). Compared to other groups, the only-symptomatic group had the highest median parasite density between one and six months of age (p < 0.001) as well as between seven and twelve months of age (p < 0.001) (Table 3).

**Table 3:** Distribution of asexual *P*. *falciparum* among the groups of infants.

Median parasite density (parasites/μl)	Only-symptomatic group	Alternating group	Only-asymptomatic group	P-value
*Range	22–974759	2–932795	19–190959	-
**Overall (**†**IQR)**	23177 (4354–101733)	5421 (1404–26355)	1702 (544–5748)	< 0.001
**At first infections (IQR)**	16717 (2912–72055)	2674 (755–12570)	1569 (388–5748)	< 0.001
**1 to 6 months of age (IQR)**	10623 (2107–35137)	4088 (1032–16275)	1143 (388–4020)	< 0.001
**7 to 12 months of age (IQR)**	38302 (7281–132975)	6843 (1794–36948)	1929 (608–6854)	< 0.001

* Not median of parasite densities.

† = Interquartile range.

Within the alternating group, the median parasite density [14812 parasite per μl (IQR: 2847–77843)] was higher when infants were symptomatic compared to when they were asymptomatic [2848 parasite/μl (IQR: 858–8228)] (p < 0.001). In all groups, the parasite density was lower in the first six months compared to seven months of age and above (all p < 0.001).

## Discussion

The longitudinal profiles and age-specific patterns of microscopy positivity with or without symptoms in 19231 monthly and ill visits were examined in a birth cohort of 1819 infants from a high malaria transmission area of Ghana. Despite living in the same area during the first year of life, infants experienced highly diverse patterns of microscopy positivity. Four main groups of infants were identified, namely: parasite negative (36%), only-symptomatic (35%), alternating (22%) and only-asymptomatic (7%). Over half of the 87 different patterns of alternating symptomatic malaria and asymptomatic microscopic infection were unique to individuals, indicating no uniform sequence of alternating symptomatic and asymptomatic microscopic infections.

In unscheduled/clinical visits where microscopy positivity was with symptoms, microscopy was performed on the same day and infants were treated, while on scheduled/home visits when infants were ‘healthy’, microscopy was performed at a later date and there was no intention to treat at the visit. Nevertheless, all visits where microscopy positivity was with or without symptoms (asymptomatic microscopic infections) were intermittent, with the asymptomatic microscopic infections spontaneously clearing up in the only-asymptomatic group. Given the gradual development of naturally acquired immunity to malaria at intermittent exposures [30, 31] and the observed intermittent distribution of infections, the natural course of malaria in the first year of life may follow the four different temporal microscopic infection profiles observed. Each time point of parasite exposure may have been accompanied by factors (placental malaria exposure, decreasing maternal antibodies, use of medications, ITN use or host genetic factors) that could modulate symptoms or morbidity [23, 32]. Modifications arising out of interactions between the infected child and combinations of these factors may have led to microscopy positivity that were always accompanied with symptoms for some infants or with no symptoms for others in the first year of life.

The higher cumulative incidence of symptomatic and asymptomatic microscopy positive visits in the alternating group suggests that in vivo or extrinsic protection against infections may be relatively poor for infants in this group. Importantly, the only-asymptomatic or only-symptomatic patterns of infection may be observed less frequently among infants in high transmission areas, compared to the alternating pattern, reducing their detection and significance to malaria in the first year of life. For instance, microscopic infections which were asymptomatic, were frequently followed by symptomatic malaria (alternating pattern only) among children aged six months to five years of age, in urban Uganda [33]. Prospectively, our findings offer new approaches for analyzing data from longitudinal studies to provide a better understanding of immunity and malaria susceptibility in infancy. For example, comparing individuals who are repeatedly microscopy positive but do not show symptoms (asymptomatic) versus those that are symptomatic, or studying the same individuals when they are asymptomatic versus when they are symptomatic.

The age at first microscopy positivity was lower for the alternating group (seven months) than for both only-asymptomatic and only-symptomatic groups (eight months). However, Wagner and others [5] reported that the median age at first infection was ten months among infants in coastal Ghana. The small sample size (71 infants) and the malaria-case definitions, based on microscopy and polymerase chain reactions (PCR) and temperature ≥ 37.5°C at the time of sample collection alone, may have contributed to the dis-similar age at first infection observed by Wagner and others [5]. The first infection which was asymptomatic increased the risk of subsequent symptomatic malaria among children who are six months to five years of age living in urban Uganda [33], but reduced the risk of subsequent symptomatic malaria among two to ten year old children in rural Mali [34]. Presently, the time frame that is a risk of a subsequent symptomatic malaria following an asymptomatic infection in the first year of life is unknown. Although we did not focus on modelling the risk of symptomatic malaria, the results suggest that after birth, asymptomatic microscopic infection occurred first before symptomatic malaria, possibly due to the protective effects of sickle cell, G6PD deficiency and/or passively transferred maternal antibodies [35, 36]. Follow-up studies are recommended, to examine the risk factors of these temporal infection profiles.

Consistent with the classic age distribution of infections in infants [12, 37, 38], we observed frequent asymptomatic microscopic infections between birth and five months of age and increased asymptomatic microscopic infections and symptomatic malaria from six to twelve months of age in the entire cohort and the groups of infants. Also, two or more discrete infections were more common than one and, as infants aged, the number of times they were infected increased in all groups. However, the rising number of infections with age were among fewer infants in all groups. These observations support the notion that in infancy, there are mechanisms that reduce the risk of symptoms [5, 18]. Furthermore, the possibility that the groups of infants have different underlying mechanisms (including host genetics, bed-net usage, exposure to maternal antibodies or parasite parameters) that reduce the risk of symptoms cannot be ruled out, as there were significant differences in the cumulative incidence of infection, parasite density and age at first infection. Additional studies focusing on the effects of exposure may be useful.

The frequency of the asexual stages of *P*. *falciparum* (96%), *P*. *malariae* (2%) and *P*. *ovale* (1.4%) was similar to that observed in other malaria endemic areas of Africa [39]. The sexual stage was of relatively low frequency (1.6%) and is consistent with observed frequencies in other endemic areas [40, 41].

Generally, studies in malaria endemic areas detect low parasite density in both asymptomatic infections and symptomatic malaria in infants below six months of age [9, 13]. Similarly, the parasite density below six months of age (where infections were mostly asymptomatic) was significantly lower than above six months of age, when all the microscopy positivity, irrespective of the profiling, were assessed. In addition, the parasite density from one to six months of age was highest in the only-symptomatic group; and throughout the entire year was significantly higher during the symptomatic periods, compared to the asymptomatic, within the alternating group. These observations suggest that low parasite densities may be protective against symptoms or that symptoms manifest due to the presence of additional factors (eg. placental malaria), as Sylvester and others have postulated [24].

In this birth cohort study, we had access to a large set of microscopy data, including the parasite density, among infants living in a high transmission area. We did not assess the substantial proportion of submicroscopic infections (may be symptomatic or asymptomatic) that can be detected using more sensitive molecular tools such as PCR [5, 42]. We described only the microscopic infections considering the design of the birth cohort study, the capacity and practicality of testing a large sample size (19,231 samples in 1819 infants) and the WHO recommendation to use the available parasitological test to confirm an infection and to state the definitions that were applied [29].

Despite its limited sensitivity compared to PCR, it is worth noting that microscopy has been extensively used to learn about the etiology, epidemiology and dynamics of malaria; it is considered a gold standard and is a more helpful method for detecting and managing clinical cases as described in the WHO malaria management guidelines [1, 43]. Standard definitions for asymptomatic infections are lacking and current research suggests that the asymptomatic reservoir is composed of microscopic and submicroscopic components [44–46]. The microscopic, compared to the submicroscopic component, is frequent in high malaria transmission settings compared to the low [4, 47, 48]. However, compared to the microscopic, there is an anticipated likelihood that the number of PCR detected submicroscopic infections may be frequent in this cohort [49]. Nevertheless, PCR may also overestimate infection rates in longitudinal cohorts that detect and treat symptomatic malaria, as PCR positivity could persist for weeks even after successful clearance of parasites with antimalarials [48, 50].

The sampling interval is important for monitoring the incidence of asymptomatic carriage and our study is one of few studies that have sampled young children monthly and even provided additional samples during ill-visits which occurred in-between the monthly sampling [51–53]. Typically, asymptomatic infections persist compared to being transient in infants [12]. Given the frequency of sampling in this study, we anticipate a reduced likelihood of missing the transient asymptomatic microscopic infections. Notwithstanding, the inability to identify submicroscopic infections may have influenced the allocation of individuals to the different groups, so it is important to restrict interpretations to microscopy.

In view of these limitations and strengths, it may be necessary to agree unanimously on definitions which consider sampling intervals for various malaria exposure groups and transmission settings, to reduce the under-reporting of asymptomatic infections. Further, intervention studies aimed at reducing malaria transmission may need to ascertain the entire burden of asymptomatic infections, including those that are submicroscopic, for optimal impact. In summary, this study’s limitation lies primarily in the use of microscopy detection methods, while the strength lies in the frequency of sampling and the analyses of a large number of monthly samples over the first twelve months of life.

Given the observed complex heterogeneity of microscopic infection patterns during infancy, studies based on single/few cross-sectional samples may briefly capture the effects of malaria exposure and consequently restrict the understanding of susceptibility and immunity to malaria in infancy. Grouping infants on the bases of their temporal malaria infections may help to better understand the immunological mechanisms of protection against malaria. The results potentially open up new opportunities for the improved analyses of host (genetic and immunological) and external determinants (behavioral and parasite parameters), which can guide future studies designed to control and eliminate malaria in endemic areas.

In conclusion, within the same transmission area, we detected by microscopy groups of infants that remained either parasite-free or with only asymptomatic infections or symptomatic malaria alone, or that intermittently alternated between asymptomatic infections and symptomatic malaria, through their first year life. These groups of infants were significantly different per their malaria parasite density and age at microscopy positivity. In-depth investigations involving the submicroscopic reservoir, to identify the underlying determinants of the variation between the groups of infants, can contribute to the understanding of malaria susceptibility during infancy.

## Supporting information

S1 Fig Frequency of monthly microscopy results. (PDF)

S2 Fig Parasite densities for asymptomatic infections and symptomatic malaria in 1674 infants. (PDF)

S3 Fig Time to an additional infection by group of infant. (PDF)

S4 Fig Temporality of infections. (PDF)

S1 Table Details of the infection periods in cohort. (PDF)

## References

[1] World Malaria Report. [Internet]. 2019. Available from: https://www.who.int/publications-detail/world-malaria-report-2019.

[2] Bartoloni A, Zammarchi L. Clinical aspects of uncomplicated and severe malaria. Mediterranean journal of hematology and infectious diseases. 2012;4(1):e2012026 10.4084/MJHID.2012.026 22708041 PMC3375727

[3] Laishram DD, Sutton PL, Nanda N, Sharma VL, Sobti RC, Carlton JM, et al The complexities of malaria disease manifestations with a focus on asymptomatic malaria. Malaria journal. 2012;11:29 10.1186/1475-2875-11-29 22289302 PMC3342920

[4] Lin JT, Saunders DL, Meshnick SR. The role of submicroscopic parasitemia in malaria transmission: what is the evidence? Trends in parasitology. 2014;30(4):183–90. 10.1016/j.pt.2014.02.004 24642035 PMC4049069

[5] Wagner G, Koram K, McGuinness D, Bennett S, Nkrumah F, Riley E. High incidence of asymptomatic malara infections in a birth cohort of children less than one year of age in Ghana, detected by multicopy gene polymerase chain reaction. The American journal of tropical medicine and hygiene. 1998;59(1):115–23. 10.4269/ajtmh.1998.59.115 9684638

[6] Lindblade KA, Steinhardt L, Samuels A, Kachur SP, Slutsker L. The silent threat: asymptomatic parasitemia and malaria transmission. Expert review of anti-infective therapy. 2013;11(6):623–39. 10.1586/eri.13.45 23750733

[7] Kangoye DT, Mensah VA, Murungi LM, Nkumama I, Nebie I, Marsh K, et al Dynamics and role of antibodies to Plasmodium falciparum merozoite antigens in children living in two settings with differing malaria transmission intensity. Vaccine. 2016;34(1):160–6. 10.1016/j.vaccine.2015.10.058 26541134 PMC4683095

[8] Ceesay SJ, Koivogui L, Nahum A, Taal MA, Okebe J, Affara M, et al Malaria prevalence among young infants in different transmission settings, Africa. Emerg Infect Dis. 2015;21(7):1114–21. 10.3201/eid2107.142036 26079062 PMC4480393

[9] Natama HM, Rovira-Vallbona E, Some MA, Zango SH, Sorgho H, Guetens P, et al Malaria incidence and prevalence during the first year of life in Nanoro, Burkina Faso: a birth-cohort study. Malaria journal. 2018;17(1):163 10.1186/s12936-018-2315-4 29650007 PMC5898041

[10] Afolabi BM, Salako LA, Mafe AG, Ovwigho UB, Rabiu KA, Sanyaolu NO, et al Malaria in the first 6 months of life in urban African infants with anemia. The American journal of tropical medicine and hygiene. 2001;65(6):822–7. 10.4269/ajtmh.2001.65.822 11791980

[11] Nweneka CV, Eneh AU. Malaria parasitaemia in neonates in Port Harcourt, Nigeria. Journal of tropical pediatrics. 2004;50(2):114–6. 10.1093/tropej/50.2.114 15088802

[12] Franks S, Koram KA, Wagner GE, Tetteh K, McGuinness D, Wheeler JG, et al Frequent and persistent, asymptomatic Plasmodium falciparum infections in African infants, characterized by multilocus genotyping. The Journal of infectious diseases. 2001;183(5):796–804. 10.1086/318834 11181157

[13] McGuinness D, Koram K, Bennett S, Wagner G, Nkrumah F, Riley E. Clinical case definitions for malaria: clinical malaria associated with very low parasite densities in African infants. Transactions of the Royal Society of Tropical Medicine and Hygiene. 1998;92(5):527–31. 10.1016/s0035-9203(98)90902-6 9861370

[14] Pasvol G, Weatherall DJ, Wilson RJ. Effects of foetal haemoglobin on susceptibility of red cells to Plasmodium falciparum. Nature. 1977;270(5633):171–3. 10.1038/270171a0 337159

[15] Amaratunga C, Lopera-Mesa TM, Brittain NJ, Cholera R, Arie T, Fujioka H, et al A role for fetal hemoglobin and maternal immune IgG in infant resistance to Plasmodium falciparum malaria. PloS one. 2011;6(4):e14798 10.1371/journal.pone.0014798 21532754 PMC3075246

[16] Hogh B, Marbiah NT, Burghaus PA, Andersen PK. Relationship between maternally derived anti-Plasmodium falciparum antibodies and risk of infection and disease in infants living in an area of Liberia, west Africa, in which malaria is highly endemic. Infection and immunity. 1995;63(10):4034–8. 10.1128/IAI.63.10.4034-4038.1995 7558316 PMC173567

[17] Khattab A, Chia YS, May J, Le Hesran JY, Deloron P, Klinkert MQ. The impact of IgG antibodies to recombinant Plasmodium falciparum 732var CIDR-1alpha domain in mothers and their newborn babies. Parasitology research. 2007;101(3):767–74. 10.1007/s00436-007-0548-1 17525854

[18] Riley EM, Wagner GE, Ofori MF, Wheeler JG, Akanmori BD, Tetteh K, et al Lack of association between maternal antibody and protection of African infants from malaria infection. Infection and immunity. 2000;68(10):5856–63. 10.1128/iai.68.10.5856-5863.2000 10992495 PMC101547

[19] Sehgal VM, Siddjiqui WA, Alpers MP. A seroepidemiological study to evaluate the role of passive maternal immunity to malaria in infants. Transactions of the Royal Society of Tropical Medicine and Hygiene. 1989;83 Suppl:105–6. 2696154 10.1016/0035-9203(89)90616-0

[20] Kangoye DT, Nebie I, Yaro JB, Debe S, Traore S, Ouedraogo O, et al Plasmodium falciparum malaria in children aged 0–2 years: the role of foetal haemoglobin and maternal antibodies to two asexual malaria vaccine candidates (MSP3 and GLURP). PloS one. 2014;9(9):e107965 10.1371/journal.pone.0107965 25238160 PMC4169582

[21] Apinjoh TO, Anchang-Kimbi JK, Mugri RN, Njua-Yafi C, Tata RB, Chi HF, et al Determinants of infant susceptibility to malaria during the first year of life in South Western Cameroon. Open forum infectious diseases. 2015;2(1):ofv012 10.1093/ofid/ofv012 26034763 PMC4438893

[22] Asante KP, Abdulla S, Agnandji S, Lyimo J, Vekemans J, Soulanoudjingar S, et al Safety and efficacy of the RTS,S/AS01E candidate malaria vaccine given with expanded-programme-on-immunisation vaccines: 19 month follow-up of a randomised, open-label, phase 2 trial. The Lancet Infectious diseases. 2011;11(10):741–9. 10.1016/S1473-3099(11)70100-1 21782519

[23] Awine T, Belko MM, Oduro AR, Oyakhirome S, Tagbor H, Chandramohan D, et al The risk of malaria in Ghanaian infants born to women managed in pregnancy with intermittent screening and treatment for malaria or intermittent preventive treatment with sulfadoxine/pyrimethamine. Malaria journal. 2016;15:46 10.1186/s12936-016-1094-z 26821532 PMC4730594

[24] Sylvester B, Gasarasi DB, Aboud S, Tarimo D, Massawe S, Mpembeni R, et al Hyperparasitaemia during clinical malaria episodes in infants aged 0–24 months and its association with in utero exposure to Plasmodium falciparum. BMC research notes. 2018;11(1):232 10.1186/s13104-018-3339-0 29618382 PMC5885461

[25] Riley EM. Is T-cell priming required for initiation of pathology in malaria infections? Immunology today. 1999;20(5):228–33. 10.1016/s0167-5699(99)01456-5 10322302

[26] Asante KP, Owusu-Agyei S, Cairns M, Dodoo D, Boamah EA, Gyasi R, et al Placental malaria and the risk of malaria in infants in a high malaria transmission area in Ghana: a prospective cohort study. The Journal of infectious diseases. 2013;208(9):1504–13. 10.1093/infdis/jit366 23908483 PMC3789576

[27] Owusu-Agyei S, Nettey OE, Zandoh C, Sulemana A, Adda R, Amenga-Etego S, et al Demographic patterns and trends in Central Ghana: baseline indicators from the Kintampo Health and Demographic Surveillance System. Global health action. 2012;5:1–11. 10.3402/gha.v5i0.19033 PMC3529298 23273249

[28] Dery DB, Brown C, Asante KP, Adams M, Dosoo D, Amenga-Etego S, et al Patterns and seasonality of malaria transmission in the forest-savannah transitional zones of Ghana. Malaria journal. 2010;9:314 10.1186/1475-2875-9-314 21054895 PMC2989982

[29] WHO Malaria Terminology [Internet]. 2018 [cited 24 September 2019]. Available from: http://www.who.int/malaria/visual-refresh/en/WHO/HTM/GMP/2016.6.

[30] Doolan DL, Dobano C, Baird JK. Acquired immunity to malaria. Clinical microbiology reviews. 2009;22(1):13–36. 10.1128/CMR.00025-08 19136431 PMC2620631

[31] Trape JF, Rogier C, Konate L, Diagne N, Bouganali H, Canque B, et al The Dielmo project: a longitudinal study of natural malaria infection and the mechanisms of protective immunity in a community living in a holoendemic area of Senegal. The American journal of tropical medicine and hygiene. 1994;51(2):123–37. 10.4269/ajtmh.1994.51.123 8074247

[32] McLean ARD, Stanisic D, McGready R, Chotivanich K, Clapham C, Baiwog F, et al P. falciparum infection and maternofetal antibody transfer in malaria-endemic settings of varying transmission. PloS one. 2017;12(10):e0186577 10.1371/journal.pone.0186577 29028827 PMC5640245

[33] Njama-Meya D, Kamya MR, Dorsey G. Asymptomatic parasitaemia as a risk factor for symptomatic malaria in a cohort of Ugandan children. Tropical Medicine and International Health. 2004;9(8):862–8. 10.1111/j.1365-3156.2004.01277.x 15303990

[34] Crompton PD, Traore B, Kayentao K, Doumbo S, Ongoiba A, Diakite SA, et al Sickle cell trait is associated with a delayed onset of malaria: implications for time-to-event analysis in clinical studies of malaria. The Journal of infectious diseases. 2008;198(9):1265–75. 10.1086/592224 18752444 PMC2574881

[35] Dobbs KR, Dent AE. Plasmodium malaria and antimalarial antibodies in the first year of life. Parasitology. 2016;143(2):129–38. 10.1017/S0031182015001626 26743626 PMC4825094

[36] Amoako N, Asante KP, Adjei G, Awandare GA, Bimi L, Owusu-Agyei S. Associations between red cell polymorphisms and Plasmodium falciparum infection in the middle belt of Ghana. PloS one. 2014;9(12):e112868 10.1371/journal.pone.0112868 25470251 PMC4254276

[37] D'Alessandro U, Ubben D, Hamed K, Ceesay SJ, Okebe J, Taal M, et al Malaria in infants aged less than six months—is it an area of unmet medical need? Malaria journal. 2012;11:400 10.1186/1475-2875-11-400 23198986 PMC3529680

[38] Binka FN, Morris SS, Ross DA, Arthur P, Aryeetey ME. Patterns of malaria morbidity and mortality in children in northern Ghana. Transactions of the Royal Society of Tropical Medicine and Hygiene. 1994;88(4):381–5. 10.1016/0035-9203(94)90391-3 7570811

[39] Culleton RL, Mita T, Ndounga M, Unger H, Cravo PV, Paganotti GM, et al Failure to detect Plasmodium vivax in West and Central Africa by PCR species typing. Malaria journal. 2008;7:174 10.1186/1475-2875-7-174 18783630 PMC2546428

[40] Coalson JE, Walldorf JA, Cohee LM, Ismail MD, Mathanga D, Cordy RJ, et al High prevalence of Plasmodium falciparum gametocyte infections in school-age children using molecular detection: patterns and predictors of risk from a cross-sectional study in southern Malawi. Malaria journal. 2016;15(1):527 10.1186/s12936-016-1587-9 27809907 PMC5096312

[41] Ayanful-Torgby R, Oppong A, Abankwa J, Acquah F, Williamson KC, Amoah LE. Plasmodium falciparum genotype and gametocyte prevalence in children with uncomplicated malaria in coastal Ghana. Malaria journal. 2016;15(1):592 10.1186/s12936-016-1640-8 27938356 PMC5148883

[42] Morassin B, Fabre R, Berry A, Magnaval JF. One year's experience with the polymerase chain reaction as a routine method for the diagnosis of imported malaria. The American journal of tropical medicine and hygiene. 2002;66(5):503–8. 10.4269/ajtmh.2002.66.503 12201583

[43] Guidelines for the treatment of malaria—Third Edition. [Internet]. 2015 [cited 11th September 2020]. Available from: https://www.who.int/docs/default-source/documents/publications/gmp/guidelines-for-the-treatment-of-malaria-eng.pdf?sfvrsn = a0138b77_2.

[44] Buchwald GA, Sixpence A, Chimenya M, Damson M, Sorkin DJ, Wilson LM, et al Clinical implications of asymptomatic Plasmodium falciparum infections in Malawi. Clinical Infectious Diseases. 2019;68:106–12. 10.1093/cid/ciy427 29788054 PMC6293006

[45] Kangoye DT, Noor A, Midega J, Mwongeli J, Mkabili D, Mogeni P, et al Malaria hotspots defined by clinical malaria, asymptomatic carriage, PCR and vector numbers in a low transmission area on the Kenyan Coast. Malaria journal. 2016;15:213 10.1186/s12936-016-1260-3 27075879 PMC4831169

[46] Males S, Gaye O, Garcia A. Long-term asymptomatic carriage of Plasmodium falciparum protects from malaria attacks: a prospective study among Senegalese children. Clinical Infectious Diseases. 2008;46(4):516–22. 10.1086/526529 18199040

[47] Alves FP, Gil LH, Marrelli MT, Ribolla PE, Camargo EP, Da Silva LH. Asymptomatic carriers of Plasmodium spp. as infection source for malaria vector mosquitoes in the Brazilian Amazon. Journal of medical entomology. 2005;42(5):777–9. 10.1093/jmedent/42.5.777 16363160

[48] Okell LC, Bousema T, Griffin JT, Ouedraogo AL, Ghani AC, Drakeley CJ. Factors determining the occurrence of submicroscopic malaria infections and their relevance for control. Nature communications. 2012;3:1237 10.1038/ncomms2241 23212366 PMC3535331

[49] Okell LC, Ghani AC, Lyons E, Drakeley CJ. Submicroscopic infection in Plasmodium falciparum-endemic populations: a systematic review and meta-analysis. The Journal of infectious diseases. 2009;200(10):1509–17. 10.1086/644781 19848588

[50] Vafa Homann M, Emami SN, Yman V, Stenstrom C, Sonden K, Ramstrom H, et al Detection of Malaria Parasites After Treatment in Travelers: A 12-months Longitudinal Study and Statistical Modelling Analysis. EBioMedicine. 2017;25:66–72. 10.1016/j.ebiom.2017.10.003 29050948 PMC5704054

[51] Farnert A, Snounou G, Rooth I, Bjorkman A. Daily dynamics of Plasmodium falciparum subpopulations in asymptomatic children in a holoendemic area. The American journal of tropical medicine and hygiene. 1997;56(5):538–47. 10.4269/ajtmh.1997.56.538 9180605

[52] Liljander A, Chandramohan D, Kweku M, Olsson D, Montgomery SM, Greenwood B, et al Influences of intermittent preventive treatment and persistent multiclonal Plasmodium falciparum infections on clinical malaria risk. PloS one. 2010;5(10):e13649 10.1371/journal.pone.0013649 21048970 PMC2965101

[53] Le Port A, Watier L, Cottrell G, Ouedraogo S, Dechavanne C, Pierrat C, et al Infections in infants during the first 12 months of life: role of placental malaria and environmental factors. PloS one. 2011;6(11):e27516 10.1371/journal.pone.0027516 22096588 PMC3214070

