# Correction: Profiles of *Plasmodium falciparum* infections detected by microscopy through the first year of life in Kintampo a high transmission area of Ghana

**DOI:** 10.1371/journal.pone.0246773

**Published:** 2021-02-03

**Authors:** Akua Kyerewaa Botwe, Seth Owusu-Agyei, Muhammad Asghar, Ulf Hammar, Felix Boakye Oppong, Stephaney Gyaase, David Dosoo, Gabriel Jakpa, Ellen Boamah, Mieks Frenken Twumasi, Faith Osier, Anna Färnert, Kwaku Poku Asante

The following information is missing from the Funding statement: This is a SMART project that was supported in part by the EDCTP2 Programme through the European Union (grant number TMA-2015-SF1001). This work was also supported in part by Swedish Research Links Grant from the Swedish Research Council to AF.
