# Supplementary material for: Profiles of Plasmodium falciparum infections detected by microscopy through the first year of life in Kintampo a high transmission area of Ghana
Source: PLoS One. 2020 Oct 19;15(10):e0240814. doi: 10.1371/journal.pone.0240814 (PMC7571695; doi:10.1371/journal.pone.0240814)

A. Number of visits among 1674 infants with values for at least one microscopy test

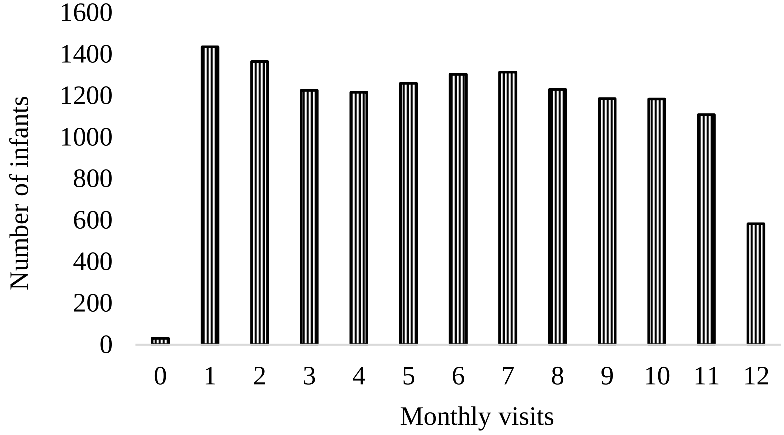

B. Age-specific distribution for 1264 infants having eight or more microscopy values

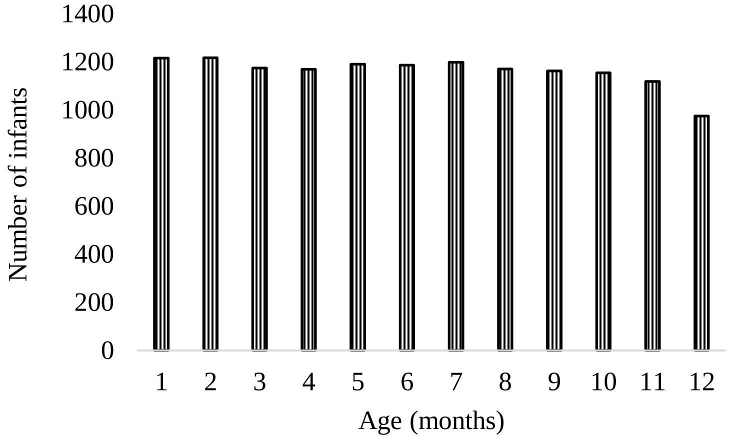

Supplement: S1 Fig — (PDF) [file pone.0240814.s001.pdf]
