# Supplementary material for: Profiles of Plasmodium falciparum infections detected by microscopy through the first year of life in Kintampo a high transmission area of Ghana
Source: PLoS One. 2020 Oct 19;15(10):e0240814. doi: 10.1371/journal.pone.0240814 (PMC7571695; doi:10.1371/journal.pone.0240814)
Supplement: S2 Fig — (PDF) [file pone.0240814.s002.pdf]

(Asymptomatic infections)

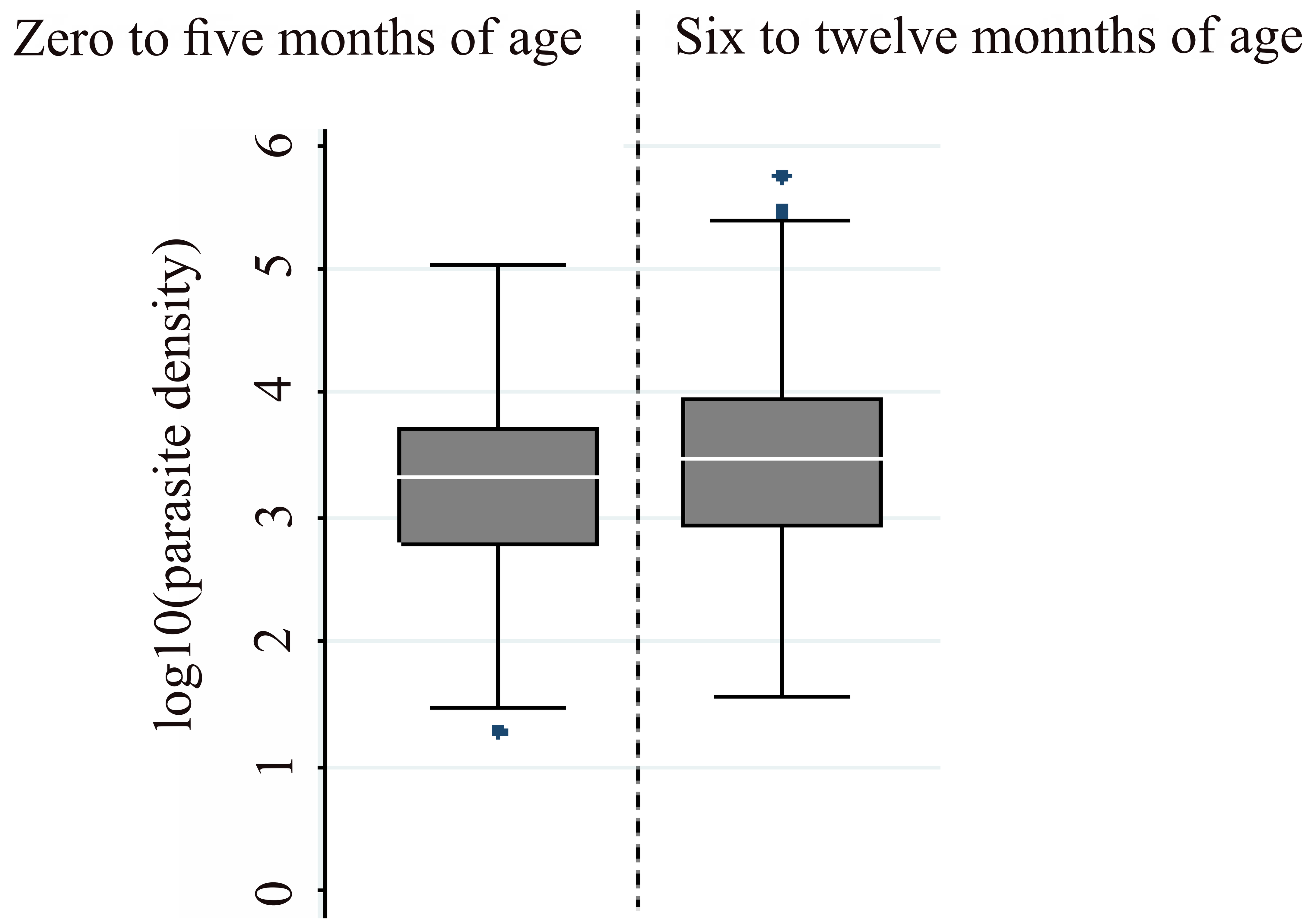

(Symptomatic infections)

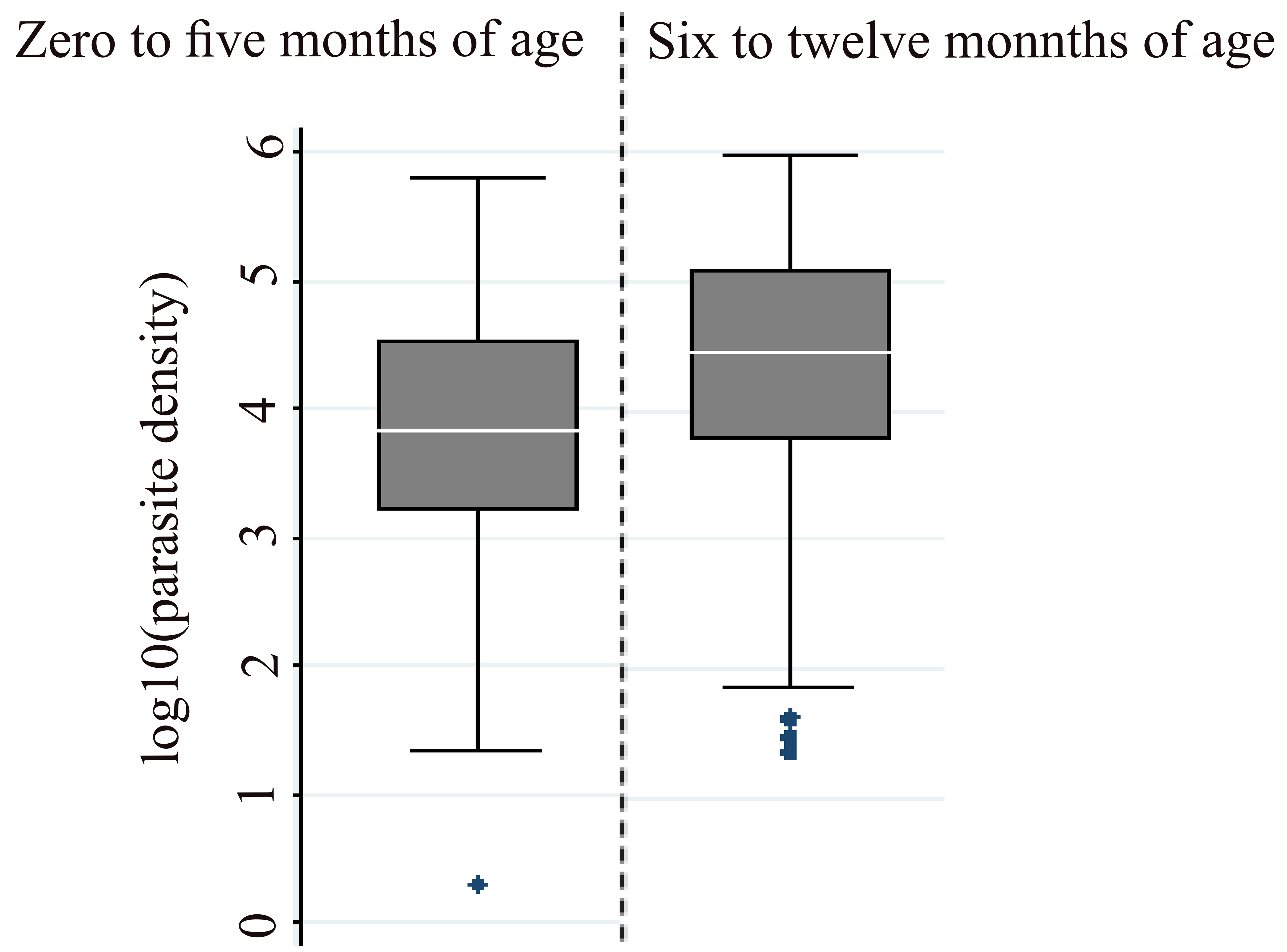

**Median parasite density (parasites/µl)**	**Asymptomatic infections**	**Symptomatic malaria**
0 – 5 Months (IQR)	2119 (598 – 5294)	6850 (1686 – 967299)
6 – 12 Months (IQR)	2949 (887 – 8727)	27766 (5966 – 118998)
P-value	< 0.001	< 0.001
