# Supplementary material for: Profiles of Plasmodium falciparum infections detected by microscopy through the first year of life in Kintampo a high transmission area of Ghana
Source: PLoS One. 2020 Oct 19;15(10):e0240814. doi: 10.1371/journal.pone.0240814 (PMC7571695; doi:10.1371/journal.pone.0240814)
Supplement: S4 Fig — (PDF) [file pone.0240814.s004.pdf]

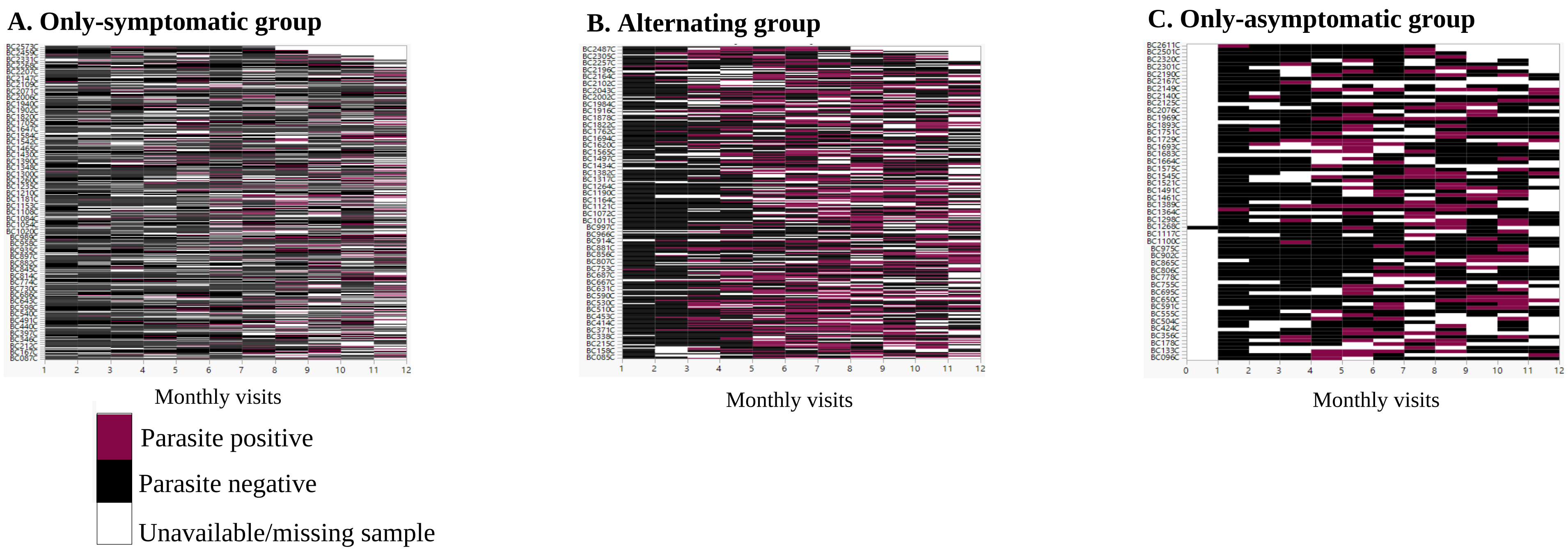

NB: The number of infants in Figs A (N = 444) and B (N = 274) were more than C (N = 87); thus in Figs A and B, a pink line is produced for overlapping missing (white line) and positive (purple line) visit data or a gray line for overlapping missing and negative (black line) visit data between any two closely spaced infants with different visit outcomes.
