# Supplementary material for: Profiles of Plasmodium falciparum infections detected by microscopy through the first year of life in Kintampo a high transmission area of Ghana
Source: PLoS One. 2020 Oct 19;15(10):e0240814. doi: 10.1371/journal.pone.0240814 (PMC7571695; doi:10.1371/journal.pone.0240814)
Supplement: S1 Table — (PDF) [file pone.0240814.s005.pdf]

Child follow up	Date of birth	Sex	Malaria at birth	Date visited	Visit type	Asexual counts	Temperature	Previous fever
1	13-Nov-08	Female	high transmission	5-Jan-09	active follow	9	36.5	No
2	13-Nov-08	Female	high transmission	6-Feb-09	active follow	9	36.2	No
3	13-Nov-08	Female	high transmission	12-Mar-09	active follow	31626	36.3	No
4	13-Nov-08	Female	high transmission	6-Apr-09	passive follow	31626	39.8	Yes
5	13-Nov-08	Female	high transmission	13-Apr-09	active follow	9	36.3	No
6	13-Nov-08	Female	high transmission	19-May-09	active follow	9	36.8	No
7	13-Nov-08	Female	high transmission	22-Jun-09	active follow		36.9	No
8	13-Nov-08	Female	high transmission	27-Jul-09	active follow	3086	36.5	No
9	13-Nov-08	Female	high transmission	10-Aug-09	passive follow	4614	38.6	Yes
10	13-Nov-08	Female	high transmission	30-Aug-09	active follow		36.8	No
11	13-Nov-08	Female	high transmission	30-Sep-09	active follow	9	36.8	No
12	13-Nov-08	Female	high transmission	30-Oct-09	active follow	9	36.9	No
13	13-Nov-08	Female	high transmission	9-Nov-09	passive follow	9	38.9	Yes
1	2-Nov-09	Male	high transmission	12-Dec-09	active follow	9	36.6	No
2	2-Nov-09	Male	high transmission	29-Dec-09	passive follow	9	36.2	No
3	2-Nov-09	Male	high transmission	13-Jan-10	active follow	9	36.5	No
4	2-Nov-09	Male	high transmission	13-Feb-10	active follow	9	36.2	No
5	2-Nov-09	Male	high transmission	18-Mar-10	active follow	324	36.5	No
6	2-Nov-09	Male	high transmission	25-Mar-10	passive follow	9	36.6	Yes
7	2-Nov-09	Male	high transmission	15-Apr-10	active follow	9	36.8	No
8	2-Nov-09	Male	high transmission	13-May-10	active follow	9	36.3	No
9	2-Nov-09	Male	high transmission	10-Jun-10	active follow	9	36	No
10	2-Nov-09	Male	high transmission	16-Jun-10	passive follow	63943	38.7	Yes
11	2-Nov-09	Male	high transmission	7-Jul-10	active follow	9	36	No
12	2-Nov-09	Male	high transmission	27-Jul-10	passive follow	437	38	Yes
13	2-Nov-09	Male	high transmission	10-Aug-10	active follow	9	36.4	No
14	2-Nov-09	Male	high transmission	7-Sep-10	active follow	9	36.4	No
15	2-Nov-09	Male	high transmission	5-Oct-10	active follow	1640	36.1	No
16	2-Nov-09	Male	high transmission	6-Oct-10	passive follow	36981	38.6	Yes
1	30-Oct-09	Female	high transmission	12-Dec-09	passive follow	9	36.3	No
2	30-Oct-09	Female	high transmission	14-Jan-10	active follow	9	36.2	No
3	30-Oct-09	Female	high transmission	8-Feb-10	passive follow	35899	36.3	Yes
4	30-Oct-09	Female	high transmission	15-Feb-10	active follow	9	35.8	No
5	30-Oct-09	Female	high transmission	17-Mar-10	active follow	9	36.4	No
6	30-Oct-09	Female	high transmission	16-Apr-10	active follow	95565	38.3	Yes
7	30-Oct-09	Female	high transmission	22-May-10	active follow		36.9	No
8	30-Oct-09	Female	high transmission	16-Jun-10	passive follow	1917	37.3	Yes
9	30-Oct-09	Female	high transmission	23-Jun-10	active follow	9	36.4	No
10	30-Oct-09	Female	high transmission	20-Jul-10	active follow	26095	36.4	No
11	30-Oct-09	Female	high transmission	16-Aug-10	active follow		38.5	Yes
12	30-Oct-09	Female	high transmission	13-Sep-10	active follow	49977	36.4	Yes
13	30-Oct-09	Female	high transmission	12-Oct-10	active follow	1321	36	No
1	24-Oct-09	Male	high transmission	25-Nov-09	active follow	9	36.7	No
2	24-Oct-09	Male	high transmission	21-Dec-09	passive follow	9	36.2	Yes
3	24-Oct-09	Male	high transmission	28-Dec-09	active follow	9	36.7	No
4	24-Oct-09	Male	high transmission	27-Jan-10	active follow	2156	36.3	No

5	24-Oct-09	Male	high transmission	2-Mar-10	active follow	349	36.6	No
6	24-Oct-09	Male	high transmission	11-Apr-10	active follow	9	37	Yes
7	24-Oct-09	Male	high transmission	14-May-10	active follow	9	36.7	No
8	24-Oct-09	Male	high transmission	17-Jun-10	active follow	9	36	No
9	24-Oct-09	Male	high transmission	11-Jul-10	active follow	24370	39.1	Yes
10	24-Oct-09	Male	high transmission	17-Aug-10	active follow			
11	24-Oct-09	Male	high transmission	27-Aug-10	passive follow	149196	38.7	Yes
12	24-Oct-09	Male	high transmission	4-Sep-10	passive follow	9	36.6	Yes
13	24-Oct-09	Male	high transmission	20-Sep-10	active follow	9	36.5	No
14	24-Oct-09	Male	high transmission	19-Oct-10	active follow	9	35.9	No
1	4-Nov-09	Female	high transmission	2-Dec-09	active follow	9	35.7	No
2	4-Nov-09	Female	high transmission	1-Jan-10	active follow	9	36.3	No
3	4-Nov-09	Female	high transmission	5-Feb-10	active follow	9	36	No
4	4-Nov-09	Female	high transmission	15-Mar-10	active follow	9	36	No
5	4-Nov-09	Female	high transmission	14-Apr-10	active follow	9	36	No
6	4-Nov-09	Female	high transmission	12-May-10	active follow	9	36.5	No
7	4-Nov-09	Female	high transmission	11-Jun-10	active follow	1304	39.6	Yes
8	4-Nov-09	Female	high transmission	14-Jul-10	active follow	27681	36.5	No
9	4-Nov-09	Female	high transmission	28-Jul-10	passive follow	105159	38.8	Yes
10	4-Nov-09	Female	high transmission	11-Aug-10	active follow	9	36	No
11	4-Nov-09	Female	high transmission	8-Sep-10	active follow	9	36	No
12	4-Nov-09	Female	high transmission	5-Oct-10	active follow	9	36	No
13	4-Nov-09	Female	high transmission	3-Nov-10	active follow	9	36.1	No
1	3-Nov-09	Female	high transmission	4-Dec-09	active follow	9	37	No
2	3-Nov-09	Female	high transmission	9-Dec-09	active follow	9	38	Yes
3	3-Nov-09	Female	high transmission	9-Jan-10	active follow	9	36.7	No
4	3-Nov-09	Female	high transmission	16-Feb-10	active follow	9	36.4	No
5	3-Nov-09	Female	high transmission	11-Mar-10	passive follow	9	36.4	No
6	3-Nov-09	Female	high transmission	21-Mar-10	active follow	9	36.7	No
7	3-Nov-09	Female	high transmission	28-Apr-10	active follow	41983	36.5	No
8	3-Nov-09	Female	high transmission	10-May-10	passive follow		36.7	Yes
9	3-Nov-09	Female	high transmission	18-May-10	active follow	9	36.6	No
10	3-Nov-09	Female	high transmission	15-Jun-10	active follow	9	37.8	Yes
11	3-Nov-09	Female	high transmission	13-Jul-10	active follow	9	36.8	No
12	3-Nov-09	Female	high transmission	14-Jul-10	passive follow	9	36.4	Yes
13	3-Nov-09	Female	high transmission	19-Aug-10	active follow			
14	3-Nov-09	Female	high transmission	25-Sep-10	active follow			
15	3-Nov-09	Female	high transmission	30-Sep-10	passive follow	3	37.2	No
16	3-Nov-09	Female	high transmission	24-Oct-10	active follow	9	36.7	Yes
1	3-Nov-09	Female	high transmission	4-Dec-09	active follow	9	36.4	No
2	3-Nov-09	Female	high transmission	8-Jan-10	active follow	9	35.6	No
3	3-Nov-09	Female	high transmission	16-Jan-10	passive follow	9	38.5	Yes
4	3-Nov-09	Female	high transmission	14-Feb-10	active follow		35.6	No
5	3-Nov-09	Female	high transmission	18-Mar-10	active follow	9	36.8	Yes
6	3-Nov-09	Female	high transmission	13-Apr-10	active follow	9	37	Yes
7	3-Nov-09	Female	high transmission	14-May-10	active follow	9	36.5	No
8	3-Nov-09	Female	high transmission	15-Jun-10	active follow	9	36.8	No

9	3-Nov-09	Female	high transmission	19-Jun-10	passive follow	53059	38.1	Yes
10	3-Nov-09	Female	high transmission	21-Jun-10	passive follow	9	36.2	No
11	3-Nov-09	Female	high transmission	14-Jul-10	active follow	9	36.8	No
12	3-Nov-09	Female	high transmission	6-Aug-10	passive follow		38.1	Yes
13	3-Nov-09	Female	high transmission	18-Aug-10	active follow	2353	36.3	No
14	3-Nov-09	Female	high transmission	14-Sep-10	active follow	9	36.3	No
15	3-Nov-09	Female	high transmission	5-Oct-10	passive follow	341868	37.6	Yes
16	3-Nov-09	Female	high transmission	19-Oct-10	active follow	4470	36.6	No
1	18-Nov-08	Female	high transmission	18-Dec-08	active follow	9	36.1	No
2	18-Nov-08	Female	high transmission	26-Jan-09	active follow	9	36.8	No
3	18-Nov-08	Female	high transmission	17-Feb-09	passive follow		37.7	Yes
4	18-Nov-08	Female	high transmission	25-Feb-09	active follow	9	36.3	No
5	18-Nov-08	Female	high transmission	18-Mar-09	passive follow		36.5	Yes
6	18-Nov-08	Female	high transmission	4-Apr-09	active follow	9	36.1	No
7	18-Nov-08	Female	high transmission	8-May-09	active follow	5192	36.3	No
8	18-Nov-08	Female	high transmission	13-Jun-09	active follow	12003	37.1	Yes
9	18-Nov-08	Female	high transmission	15-Jun-09	passive follow		37	Yes
10	18-Nov-08	Female	high transmission	15-Jul-09	active follow	9	35.2	No
11	18-Nov-08	Female	high transmission	18-Aug-09	active follow	31775	37	Yes
12	18-Nov-08	Female	high transmission	16-Sep-09	active follow	9	35.4	No
13	18-Nov-08	Female	high transmission	20-Oct-09	active follow	2237	36.4	No
1	14-Nov-09	Male	high transmission	21-Dec-09	active follow	9	36.8	No
2	14-Nov-09	Male	high transmission	25-Jan-10	active follow	9	37.2	No
3	14-Nov-09	Male	high transmission	4-Mar-10	active follow	9	39.1	Yes
4	14-Nov-09	Male	high transmission	3-Apr-10	active follow	9	36.5	No
5	14-Nov-09	Male	high transmission	14-Apr-10	passive follow	9	36.7	No
6	14-Nov-09	Male	high transmission	5-May-10	active follow	9	36.8	No
7	14-Nov-09	Male	high transmission	2-Jun-10	active follow	1910	36.1	No
8	14-Nov-09	Male	high transmission	1-Jul-10	active follow	9	36.8	No
9	14-Nov-09	Male	high transmission	29-Jul-10	active follow	21694	38	Yes
10	14-Nov-09	Male	high transmission	26-Aug-10	active follow	9	36.3	No
11	14-Nov-09	Male	high transmission	21-Sep-10	active follow	9	36.7	No
12	14-Nov-09	Male	high transmission	26-Oct-10	active follow	9	36.8	No
1	15-Nov-09	Female	high transmission	19-Dec-09	active follow	9	36.5	No
2	15-Nov-09	Female	high transmission	23-Jan-10	active follow	63	37	No
3	15-Nov-09	Female	high transmission	28-Jan-10	passive follow	9	36.4	Yes
4	15-Nov-09	Female	high transmission	10-Feb-10	passive follow	9	35.5	No
5	15-Nov-09	Female	high transmission	26-Feb-10	active follow	9	36.3	No
6	15-Nov-09	Female	high transmission	18-Mar-10	passive follow	9	36.1	Yes
7	15-Nov-09	Female	high transmission	12-Apr-10	active follow	9	35.7	No
8	15-Nov-09	Female	high transmission	19-May-10	active follow			
9	15-Nov-09	Female	high transmission	28-May-10	passive follow	105166		
10	15-Nov-09	Female	high transmission	31-May-10	passive follow			No
11	15-Nov-09	Female	high transmission	15-Jun-10	active follow	9	35	No
12	15-Nov-09	Female	high transmission	1-Jul-10	passive follow	379581	40.4	Yes
13	15-Nov-09	Female	high transmission	19-Jul-10	active follow		35.7	No
14	15-Nov-09	Female	high transmission	22-Jul-10	passive follow	9	35.8	No

15	15-Nov-09	Female	high transmission	18-Aug-10	active follow	9	35.7	No
16	15-Nov-09	Female	high transmission	19-Sep-10	active follow		36.3	No
17	15-Nov-09	Female	high transmission	14-Oct-10	passive follow		35.7	Yes
18	15-Nov-09	Female	high transmission	26-Oct-10	active follow			
1	16-Nov-09	Female	high transmission	17-Dec-09	active follow	9	35.6	No
2	16-Nov-09	Female	high transmission	15-Jan-10	active follow	9	36	No
3	16-Nov-09	Female	high transmission	22-Feb-10	active follow	9	36	No
4	16-Nov-09	Female	high transmission	26-Mar-10	passive follow	9	37	Yes
5	16-Nov-09	Female	high transmission	4-May-10	active follow		36.5	Yes
6	16-Nov-09	Female	high transmission	25-May-10	passive follow	9	38	Yes
7	16-Nov-09	Female	high transmission	2-Jun-10	active follow	9	36.5	No
8	16-Nov-09	Female	high transmission	9-Jun-10	passive follow	9	36.7	Yes
9	16-Nov-09	Female	high transmission	18-Jun-10	passive follow	9	36.7	Yes
10	16-Nov-09	Female	high transmission	5-Jul-10	active follow	9	36.2	No
11	16-Nov-09	Female	high transmission	3-Aug-10	active follow	55035	38.5	Yes
12	16-Nov-09	Female	high transmission	25-Aug-10	passive follow	24333	36	Yes
13	16-Nov-09	Female	high transmission	21-Sep-10	active follow	3246	36.2	No
14	16-Nov-09	Female	high transmission	21-Oct-10	active follow	2498	38.4	Yes
15	16-Nov-09	Female	high transmission	13-Nov-10	active follow	9	38.9	Yes
1	19-Nov-08	Male	high transmission	20-Dec-08	active follow	9	36.6	No
2	19-Nov-08	Male	high transmission	20-Jan-09	active follow		36.2	No
3	19-Nov-08	Male	high transmission	19-Feb-09	active follow	9	36	No
4	19-Nov-08	Male	high transmission	17-Mar-09	active follow	9	36	No
5	19-Nov-08	Male	high transmission	23-Apr-09	active follow	9	36.1	No
6	19-Nov-08	Male	high transmission	22-May-09	active follow	9	36	No
7	19-Nov-08	Male	high transmission	29-Jun-09	active follow	9	36.2	No
8	19-Nov-08	Male	high transmission	3-Aug-09	active follow	6708	36	No
9	19-Nov-08	Male	high transmission	6-Sep-09	active follow	892	36.6	No
10	19-Nov-08	Male	high transmission	21-Sep-09	passive follow	9	36.7	Yes
11	19-Nov-08	Male	high transmission	12-Oct-09	active follow	2733	36.8	No
12	19-Nov-08	Male	high transmission	4-Nov-09	passive follow	20419	36.6	Yes
13	19-Nov-08	Male	high transmission	15-Nov-09	active follow	726	36.3	No
1	20-Nov-09	Male	high transmission	27-Dec-09	active follow	9	36.9	No
2	20-Nov-09	Male	high transmission	30-Jan-10	active follow	9	36.6	No
3	20-Nov-09	Male	high transmission	5-Mar-10	active follow	9	36.6	No
4	20-Nov-09	Male	high transmission	9-Apr-10	active follow	9	36.4	No
5	20-Nov-09	Male	high transmission	14-May-10	active follow	9	36.1	No
6	20-Nov-09	Male	high transmission	25-Jun-10	active follow	9	36.8	No
7	20-Nov-09	Male	high transmission	27-Jul-10	active follow	1223	36.6	No
8	20-Nov-09	Male	high transmission	30-Jul-10	passive follow	10199	37.6	Yes
9	20-Nov-09	Male	high transmission	26-Aug-10	active follow	1847	36	No
10	20-Nov-09	Male	high transmission	30-Sep-10	active follow		36.5	No
11	20-Nov-09	Male	high transmission	31-Oct-10	active follow	9	36.6	No
1	22-Nov-09	Female	high transmission	24-Dec-09	active follow	9	36.2	No
2	22-Nov-09	Female	high transmission	22-Jan-10	active follow	9	36.4	No
3	22-Nov-09	Female	high transmission	25-Feb-10	active follow	9	36	No
4	22-Nov-09	Female	high transmission	23-Mar-10	active follow	9	35.3	No

5	22-Nov-09	Female	high transmission	21-Apr-10	active follow	9	36.5	No
6	22-Nov-09	Female	high transmission	20-May-10	active follow	9	36.5	No
7	22-Nov-09	Female	high transmission	15-Jun-10	active follow		38.1	Yes
8	22-Nov-09	Female	high transmission	14-Jul-10	active follow	3482	36.2	No
9	22-Nov-09	Female	high transmission	7-Aug-10	active follow	113905	36.6	Yes
10	22-Nov-09	Female	high transmission	22-Sep-10	passive follow	9	36.2	Yes
11	22-Nov-09	Female	high transmission	14-Oct-10	passive follow	9	36.4	Yes
12	22-Nov-09	Female	high transmission	19-Oct-10	active follow			
13	22-Nov-09	Female	high transmission	18-Nov-10	active follow			
1	15-Nov-09	Female	high transmission	21-Dec-09	active follow	9	36.5	No
2	15-Nov-09	Female	high transmission	30-Jan-10	active follow	9	36	No
3	15-Nov-09	Female	high transmission	9-Mar-10	active follow	9	36.3	No
4	15-Nov-09	Female	high transmission	17-Mar-10	passive follow	9	36.2	Yes
5	15-Nov-09	Female	high transmission	8-Apr-10	active follow		35.8	No
6	15-Nov-09	Female	high transmission	23-Apr-10	passive follow	3058	38.1	Yes
7	15-Nov-09	Female	high transmission	15-May-10	active follow	1190	36	No
8	15-Nov-09	Female	high transmission	17-Jun-10	active follow	9	35.9	No
9	15-Nov-09	Female	high transmission	20-Jul-10	active follow	7171	36.4	Yes
10	15-Nov-09	Female	high transmission	19-Aug-10	active follow	9	36.5	No
11	15-Nov-09	Female	high transmission	22-Sep-10	active follow	9	36.5	No
12	15-Nov-09	Female	high transmission	27-Oct-10	active follow	490	36.8	Yes
1	24-Nov-09	Male	high transmission	25-Dec-09	active follow	9	36.5	No
2	24-Nov-09	Male	high transmission	1-Feb-10	active follow	9	36	No
3	24-Nov-09	Male	high transmission	4-Mar-10	active follow	9	36.3	No
4	24-Nov-09	Male	high transmission	5-Apr-10	active follow	9	36	No
5	24-Nov-09	Male	high transmission	4-May-10	active follow		36.3	No
6	24-Nov-09	Male	high transmission	2-Jun-10	active follow	9	36.3	No
7	24-Nov-09	Male	high transmission	10-Jun-10	passive follow	88448	36.6	Yes
8	24-Nov-09	Male	high transmission	5-Jul-10	active follow	13640	36.4	No
9	24-Nov-09	Male	high transmission	5-Aug-10	active follow	6547	37	No
10	24-Nov-09	Male	high transmission	6-Sep-10	active follow	9	36.6	No
11	24-Nov-09	Male	high transmission	8-Oct-10	active follow	28444	36.4	No
12	24-Nov-09	Male	high transmission	16-Nov-10	active follow		36.2	No
1	20-Nov-09	Male	high transmission	25-Dec-09	active follow	9	36.8	No
2	20-Nov-09	Male	high transmission	1-Feb-10	active follow	9	36.7	No
3	20-Nov-09	Male	high transmission	7-Mar-10	active follow	9	37	No
4	20-Nov-09	Male	high transmission	11-Mar-10	passive follow	9	37.4	Yes
5	20-Nov-09	Male	high transmission	9-Apr-10	active follow	9	36.8	No
6	20-Nov-09	Male	high transmission	13-May-10	active follow		36.6	No
7	20-Nov-09	Male	high transmission	25-May-10	passive follow	9	39.5	Yes
8	20-Nov-09	Male	high transmission	12-Jun-10	active follow	9	36.9	No
9	20-Nov-09	Male	high transmission	17-Jul-10	active follow	123	36.8	No
10	20-Nov-09	Male	high transmission	20-Jul-10	passive follow	300119	39.6	Yes
11	20-Nov-09	Male	high transmission	18-Aug-10	active follow	9	36	No
12	20-Nov-09	Male	high transmission	16-Sep-10	active follow	9	36.6	No
13	20-Nov-09	Male	high transmission	1-Oct-10	passive follow	9	36.5	No
14	20-Nov-09	Male	high transmission	19-Oct-10	active follow	9	36.5	No

15	20-Nov-09	Male	high transmission	18-Nov-10	active follow	2010	36.8	No
1	15-Nov-08	Female	high transmission	15-Dec-08	active follow	9	36.5	No
2	15-Nov-08	Female	high transmission	16-Dec-08	passive follow		36.7	No
3	15-Nov-08	Female	high transmission	19-Jan-09	active follow		36.6	No
4	15-Nov-08	Female	high transmission	28-Jan-09	passive follow		36	Yes
5	15-Nov-08	Female	high transmission	23-Feb-09	active follow		36	No
6	15-Nov-08	Female	high transmission	20-Mar-09	passive follow		37.1	Yes
7	15-Nov-08	Female	high transmission	24-Mar-09	active follow	9	36.3	No
8	15-Nov-08	Female	high transmission	24-Apr-09	active follow	2267	35.9	No
9	15-Nov-08	Female	high transmission	30-May-09	active follow	65353	36.2	No
10	15-Nov-08	Female	high transmission	3-Jul-09	active follow	9	36.7	Yes
11	15-Nov-08	Female	high transmission	10-Aug-09	active follow	9	36	No
12	15-Nov-08	Female	high transmission	12-Aug-09	passive follow	9		
13	15-Nov-08	Female	high transmission	22-Aug-09	passive follow	932795	37.7	Yes
14	15-Nov-08	Female	high transmission	16-Sep-09	active follow			
15	15-Nov-08	Female	high transmission	18-Sep-09	passive follow		40.7	Yes
16	15-Nov-08	Female	high transmission	9-Oct-09	passive follow		35.8	Yes
17	15-Nov-08	Female	high transmission	14-Oct-09	active follow	9	36	No
1	24-Nov-09	Male	high transmission	26-Dec-09	active follow	9	36.6	No
2	24-Nov-09	Male	high transmission	29-Jan-10	passive follow	9	36.9	Yes
3	24-Nov-09	Male	high transmission	30-Jan-10	active follow		36.9	Yes
4	24-Nov-09	Male	high transmission	7-Mar-10	active follow	9	36.8	No
5	24-Nov-09	Male	high transmission	24-Mar-10	passive follow	9	36.1	Yes
6	24-Nov-09	Male	high transmission	13-Apr-10	active follow	9	36	No
7	24-Nov-09	Male	high transmission	7-May-10	passive follow	9	36.6	Yes
8	24-Nov-09	Male	high transmission	11-May-10	active follow	9	36.5	No
9	24-Nov-09	Male	high transmission	10-Jun-10	active follow	9	36.4	No
10	24-Nov-09	Male	high transmission	12-Jul-10	passive follow	9	36.3	Yes
11	24-Nov-09	Male	high transmission	11-Aug-10	active follow	920	36.1	No
12	24-Nov-09	Male	high transmission	20-Aug-10	passive follow	2218	37.3	Yes
13	24-Nov-09	Male	high transmission	15-Sep-10	active follow	8868	36.6	No
14	24-Nov-09	Male	high transmission	2-Oct-10	passive follow	28667	39.4	Yes
15	24-Nov-09	Male	high transmission	13-Oct-10	active follow	281	36.3	No
16	24-Nov-09	Male	high transmission	18-Nov-10	active follow	1645	36.5	No
17	24-Nov-09	Male	high transmission	21-Nov-10	passive follow	38	38	Yes
1	26-Nov-09	Female	high transmission	30-Dec-09	active follow	9	35.8	No
2	26-Nov-09	Female	high transmission	1-Feb-10	active follow	9	36.3	No
3	26-Nov-09	Female	high transmission	4-Mar-10	active follow	9	36.8	No
4	26-Nov-09	Female	high transmission	5-Apr-10	active follow	9	36	No
5	26-Nov-09	Female	high transmission	4-May-10	active follow	9	35.6	No
6	26-Nov-09	Female	high transmission	3-Jun-10	active follow	9	35.6	No
7	26-Nov-09	Female	high transmission	7-Jul-10	active follow	9117	36.9	No
8	26-Nov-09	Female	high transmission	6-Aug-10	active follow	9899	36.3	Yes
9	26-Nov-09	Female	high transmission	10-Sep-10	active follow	15515	38.4	Yes
10	26-Nov-09	Female	high transmission	13-Sep-10	active follow	15515	36	Yes
11	26-Nov-09	Female	high transmission	18-Oct-10	active follow			
12	26-Nov-09	Female	high transmission	22-Nov-10	active follow	9	36.3	No

1	30-Nov-09	Female	high transmission	3-Jan-10	active follow	9	36.3	No
2	30-Nov-09	Female	high transmission	2-Feb-10	passive follow	9	36.3	Yes
3	30-Nov-09	Female	high transmission	9-Feb-10	active follow	9	36	No
4	30-Nov-09	Female	high transmission	16-Mar-10	active follow		36.2	Yes
5	30-Nov-09	Female	high transmission	19-Apr-10	active follow	9	36.8	Yes
6	30-Nov-09	Female	high transmission	11-May-10	passive follow	9	36.5	Yes
7	30-Nov-09	Female	high transmission	25-May-10	active follow	9	36.3	No
8	30-Nov-09	Female	high transmission	29-May-10	passive follow	9	36.6	No
9	30-Nov-09	Female	high transmission	21-Jun-10	active follow	9	36.7	Yes
10	30-Nov-09	Female	high transmission	20-Jul-10	active follow	9		No
11	30-Nov-09	Female	high transmission	28-Jul-10	passive follow	9	36.4	Yes
12	30-Nov-09	Female	high transmission	14-Aug-10	passive follow	12078	37.8	Yes
13	30-Nov-09	Female	high transmission	25-Aug-10	active follow	9	36.5	No
14	30-Nov-09	Female	high transmission	13-Sep-10	passive follow	9	36.5	No
15	30-Nov-09	Female	high transmission	21-Sep-10	active follow	1491	36.7	No
16	30-Nov-09	Female	high transmission	13-Oct-10	passive follow	2197	37.3	Yes
17	30-Nov-09	Female	high transmission	21-Oct-10	active follow	9	36.5	No
18	30-Nov-09	Female	high transmission	16-Nov-10	active follow	9	36	No
1	1-Dec-09	Female	low transmission	4-Jan-10	active follow	9	36.6	No
2	1-Dec-09	Female	low transmission	9-Feb-10	passive follow	9	36.4	Yes
3	1-Dec-09	Female	low transmission	20-Mar-10	active follow	9	36	No
4	1-Dec-09	Female	low transmission	27-Apr-10	active follow	9	36.1	No
5	1-Dec-09	Female	low transmission	3-May-10	passive follow	9	37	Yes
6	1-Dec-09	Female	low transmission	29-May-10	active follow		36	No
7	1-Dec-09	Female	low transmission	5-Jul-10	active follow	9	36.3	Yes
8	1-Dec-09	Female	low transmission	27-Jul-10	passive follow	10788	36	Yes
9	1-Dec-09	Female	low transmission	10-Aug-10	active follow	18589	36.5	No
10	1-Dec-09	Female	low transmission	6-Sep-10	passive follow	116228	38.3	Yes
11	1-Dec-09	Female	low transmission	16-Sep-10	active follow	9	36.8	No
12	1-Dec-09	Female	low transmission	18-Oct-10	active follow	9	35.8	No
13	1-Dec-09	Female	low transmission	22-Nov-10	active follow	9	36.3	No
1	5-Dec-09	Female	low transmission	11-Jan-10	active follow	9	36.7	No
2	5-Dec-09	Female	low transmission	12-Feb-10	active follow	9	37.7	Yes
3	5-Dec-09	Female	low transmission	18-Mar-10	active follow	9	36.8	Yes
4	5-Dec-09	Female	low transmission	16-Apr-10	active follow	9	36.9	No
5	5-Dec-09	Female	low transmission	19-Apr-10	passive follow	9		
6	5-Dec-09	Female	low transmission	24-May-10	active follow	279	36.8	Yes
7	5-Dec-09	Female	low transmission	25-Jun-10	active follow	54079	38.6	Yes
8	5-Dec-09	Female	low transmission	21-Jul-10	passive follow		38.6	Yes
9	5-Dec-09	Female	low transmission	1-Aug-10	active follow		36.6	No
10	5-Dec-09	Female	low transmission	7-Sep-10	active follow		36	Yes
11	5-Dec-09	Female	low transmission	14-Oct-10	active follow	5598	36.2	No
12	5-Dec-09	Female	low transmission	15-Nov-10	active follow		36.4	No
1	3-Dec-09	Male	low transmission	4-Jan-10	active follow	9	36.3	No
2	3-Dec-09	Male	low transmission	9-Feb-10	active follow	9	36.3	No
3	3-Dec-09	Male	low transmission	18-Mar-10	active follow	9	36.1	No
4	3-Dec-09	Male	low transmission	19-Apr-10	active follow	9		No

5	3-Dec-09	Male	low transmission	22-May-10	active follow	595	39.9	Yes
6	3-Dec-09	Male	low transmission	25-Jun-10	active follow	94944	36.8	Yes
7	3-Dec-09	Male	low transmission	29-Jul-10	active follow	9	36.6	No
8	3-Dec-09	Male	low transmission	25-Aug-10	active follow	9	36.6	No
9	3-Dec-09	Male	low transmission	1-Oct-10	active follow		37.1	Yes
10	3-Dec-09	Male	low transmission	5-Nov-10	active follow	98440	36	No
1	6-Dec-09	Female	low transmission	7-Jan-10	active follow	9	36.8	No
2	6-Dec-09	Female	low transmission	9-Feb-10	active follow	9	36.3	No
3	6-Dec-09	Female	low transmission	13-Mar-10	active follow	9	36.5	No
4	6-Dec-09	Female	low transmission	16-Apr-10	active follow	9		No
5	6-Dec-09	Female	low transmission	24-May-10	active follow		37.3	Yes
6	6-Dec-09	Female	low transmission	9-Jun-10	passive follow	9	36.2	Yes
7	6-Dec-09	Female	low transmission	28-Jun-10	active follow	9	36.8	No
8	6-Dec-09	Female	low transmission	2-Aug-10	active follow	72	35.5	No
9	6-Dec-09	Female	low transmission	8-Sep-10	active follow		36.4	Yes
10	6-Dec-09	Female	low transmission	18-Sep-10	passive follow	189091	38.4	Yes
11	6-Dec-09	Female	low transmission	6-Oct-10	active follow	9	36.4	No
12	6-Dec-09	Female	low transmission	3-Nov-10	active follow	9	36.5	No
14	6-Dec-09	Female	low transmission	6-Dec-10	active follow	9	36.1	No
1	6-Dec-09	Female	low transmission	7-Jan-10	active follow	9	35.8	No
2	6-Dec-09	Female	low transmission	9-Feb-10	active follow	9	35.8	No
3	6-Dec-09	Female	low transmission	18-Mar-10	active follow	9	37.1	Yes
4	6-Dec-09	Female	low transmission	20-Apr-10	active follow	9	37.1	Yes
5	6-Dec-09	Female	low transmission	27-May-10	active follow	9	36.3	No
6	6-Dec-09	Female	low transmission	28-Jun-10	active follow	1743	36.5	No
7	6-Dec-09	Female	low transmission	29-Jul-10	passive follow		36.5	Yes
8	6-Dec-09	Female	low transmission	30-Jul-10	active follow		36.5	Yes
9	6-Dec-09	Female	low transmission	8-Sep-10	passive follow	154658	36.3	Yes
10	6-Dec-09	Female	low transmission	8-Oct-10	active follow	9	36.3	No
11	6-Dec-09	Female	low transmission	12-Nov-10	active follow	12560	38.6	Yes
1	6-Dec-09	Female	low transmission	6-Jan-10	active follow	9	36.9	No
2	6-Dec-09	Female	low transmission	9-Feb-10	active follow	9	36.8	No
3	6-Dec-09	Female	low transmission	17-Mar-10	active follow	9	36	No
4	6-Dec-09	Female	low transmission	15-Apr-10	active follow	439	36	No
5	6-Dec-09	Female	low transmission	28-Apr-10	passive follow		36.7	Yes
6	6-Dec-09	Female	low transmission	18-May-10	passive follow		36.5	No
7	6-Dec-09	Female	low transmission	22-May-10	active follow			
8	6-Dec-09	Female	low transmission	1-Jul-10	active follow	9	36.6	No
9	6-Dec-09	Female	low transmission	20-Jul-10	passive follow	33	36	No
10	6-Dec-09	Female	low transmission	3-Aug-10	active follow	9	36.7	No
11	6-Dec-09	Female	low transmission	6-Aug-10	passive follow	9	38	Yes
12	6-Dec-09	Female	low transmission	7-Sep-10	active follow	9	36.9	No
13	6-Dec-09	Female	low transmission	17-Sep-10	passive follow	80687	37.8	Yes
14	6-Dec-09	Female	low transmission	21-Oct-10	active follow	9	36.6	No
15	6-Dec-09	Female	low transmission	9-Nov-10	passive follow	771	37.2	Yes
16	6-Dec-09	Female	low transmission	30-Nov-10	active follow	9	36	No
1	2-Dec-09	Female	low transmission	31-Dec-09	active follow	9	36.3	No

2	2-Dec-09	Female	low transmission	1-Feb-10	active follow	9	36.6	No
3	2-Dec-09	Female	low transmission	7-Mar-10	active follow	9	36.3	No
4	2-Dec-09	Female	low transmission	9-Apr-10	active follow	9	36.6	No
5	2-Dec-09	Female	low transmission	13-May-10	active follow	398	37	No
6	2-Dec-09	Female	low transmission	12-Jun-10	active follow	2712	35.4	No
7	2-Dec-09	Female	low transmission	22-Jun-10	passive follow		39.6	Yes
8	2-Dec-09	Female	low transmission	23-Jun-10	passive follow	9		
9	2-Dec-09	Female	low transmission	17-Jul-10	active follow	9	36.5	No
10	2-Dec-09	Female	low transmission	19-Aug-10	active follow	36637	36.7	No
11	2-Dec-09	Female	low transmission	15-Sep-10	active follow	5646	36.6	No
12	2-Dec-09	Female	low transmission	13-Oct-10	passive follow	1178	39	Yes
13	2-Dec-09	Female	low transmission	22-Oct-10	active follow			
14	2-Dec-09	Female	low transmission	25-Nov-10	active follow	9	36.3	No
1	8-Dec-09	Female	low transmission	7-Jan-10	active follow	9	35.8	No
2	8-Dec-09	Female	low transmission	9-Feb-10	active follow	9	36.8	No
3	8-Dec-09	Female	low transmission	22-Mar-10	active follow	9	37	Yes
4	8-Dec-09	Female	low transmission	12-Apr-10	passive follow	9	38.9	Yes
5	8-Dec-09	Female	low transmission	28-Apr-10	active follow		36.9	Yes
6	8-Dec-09	Female	low transmission	17-May-10	passive follow		37	Yes
7	8-Dec-09	Female	low transmission	25-May-10	active follow		35.2	No
8	8-Dec-09	Female	low transmission	27-Jun-10	active follow	9	36.3	No
9	8-Dec-09	Female	low transmission	28-Jul-10	passive follow	66919	38.6	Yes
10	8-Dec-09	Female	low transmission	27-Aug-10	active follow	1848	35.5	No
11	8-Dec-09	Female	low transmission	1-Oct-10	active follow	24087	36.3	Yes
12	8-Dec-09	Female	low transmission	5-Nov-10	active follow	247411	38.3	Yes
13	8-Dec-09	Female	low transmission	3-Dec-10	passive follow	286845	37.3	Yes
1	9-Dec-09	Female	low transmission	6-Jan-10	active follow	9	36.9	No
2	9-Dec-09	Female	low transmission	9-Feb-10	active follow	9	36.4	No
3	9-Dec-09	Female	low transmission	15-Mar-10	active follow	9	37.1	Yes
4	9-Dec-09	Female	low transmission	15-Apr-10	active follow		35.8	No
5	9-Dec-09	Female	low transmission	16-Apr-10	active follow	9	35.8	No
6	9-Dec-09	Female	low transmission	22-May-10	active follow			
7	9-Dec-09	Female	low transmission	11-Jun-10	passive follow	151893		
8	9-Dec-09	Female	low transmission	28-Jun-10	active follow	9	36.5	No
9	9-Dec-09	Female	low transmission	27-Jul-10	active follow	34114	36	No
10	9-Dec-09	Female	low transmission	3-Aug-10	passive follow		38	Yes
11	9-Dec-09	Female	low transmission	4-Aug-10	passive follow	7518		
12	9-Dec-09	Female	low transmission	30-Aug-10	active follow		38.8	Yes
13	9-Dec-09	Female	low transmission	16-Sep-10	passive follow	9	37.8	Yes
14	9-Dec-09	Female	low transmission	8-Oct-10	active follow	9	36.7	No
15	9-Dec-09	Female	low transmission	15-Nov-10	active follow	264346	37.4	Yes
1	11-Dec-09	Female	low transmission	14-Jan-10	active follow	9	36.8	No
2	11-Dec-09	Female	low transmission	16-Feb-10	active follow			
3	11-Dec-09	Female	low transmission	17-Mar-10	active follow	9	36.6	No
4	11-Dec-09	Female	low transmission	30-Apr-10	active follow		36	No
5	11-Dec-09	Female	low transmission	7-May-10	passive follow	9	35	Yes
6	11-Dec-09	Female	low transmission	3-Jun-10	active follow	9	36.1	No

7	11-Dec-09	Female	low transmission	3-Jul-10	active follow	9	36	No
8	11-Dec-09	Female	low transmission	11-Aug-10	active follow	1687	36.6	No
9	11-Dec-09	Female	low transmission	7-Sep-10	active follow	2307	36.3	No
10	11-Dec-09	Female	low transmission	21-Sep-10	passive follow	140132	36.8	Yes
11	11-Dec-09	Female	low transmission	7-Oct-10	active follow	9	36.5	No
12	11-Dec-09	Female	low transmission	4-Nov-10	active follow	9	36.2	No
13	11-Dec-09	Female	low transmission	30-Nov-10	active follow	9	36.5	No
1	11-Dec-09	Male	low transmission	15-Jan-10	active follow	9	36.8	No
2	11-Dec-09	Male	low transmission	18-Feb-10	active follow		36.4	No
3	11-Dec-09	Male	low transmission	19-Mar-10	active follow		35.3	No
4	11-Dec-09	Male	low transmission	22-Apr-10	active follow	4420	36.5	No
5	11-Dec-09	Male	low transmission	6-May-10	passive follow	212477	39.1	Yes
6	11-Dec-09	Male	low transmission	25-May-10	active follow	9	36.7	No
7	11-Dec-09	Male	low transmission	31-May-10	passive follow	272702		
8	11-Dec-09	Male	low transmission	28-Jun-10	active follow	9	36.4	No
9	11-Dec-09	Male	low transmission	9-Jul-10	passive follow	211518	36.7	Yes
10	11-Dec-09	Male	low transmission	29-Jul-10	active follow	9	36.4	No
11	11-Dec-09	Male	low transmission	14-Aug-10	passive follow	9		
12	11-Dec-09	Male	low transmission	28-Aug-10	active follow	9	36.5	No
13	11-Dec-09	Male	low transmission	29-Sep-10	active follow	9	37.2	No
14	11-Dec-09	Male	low transmission	12-Oct-10	passive follow	122012	37	Yes
15	11-Dec-09	Male	low transmission	26-Oct-10	active follow	9	36.4	No
16	11-Dec-09	Male	low transmission	4-Nov-10	passive follow	95900	38.4	Yes
17	11-Dec-09	Male	low transmission	25-Nov-10	active follow	9	35.5	No
18	11-Dec-09	Male	low transmission	4-Dec-10	passive follow	224964		
1	16-Dec-09	Female	low transmission	17-Jan-10	active follow	9	36.3	No
2	16-Dec-09	Female	low transmission	18-Feb-10	active follow		36.3	No
3	16-Dec-09	Female	low transmission	17-Mar-10	active follow	9	35.5	No
4	16-Dec-09	Female	low transmission	15-Apr-10	active follow	9	36	No
5	16-Dec-09	Female	low transmission	12-May-10	active follow	9	36.1	No
6	16-Dec-09	Female	low transmission	13-Jun-10	active follow	1305	36.3	No
7	16-Dec-09	Female	low transmission	14-Jun-10	passive follow	7632	37.2	Yes
8	16-Dec-09	Female	low transmission	14-Jul-10	active follow	5864	36	No
9	16-Dec-09	Female	low transmission	13-Aug-10	active follow	5184	37.3	Yes
10	16-Dec-09	Female	low transmission	15-Sep-10	active follow	9	36	No
11	16-Dec-09	Female	low transmission	25-Oct-10	active follow		36.1	No
12	16-Dec-09	Female	low transmission	1-Dec-10	active follow	9	36	No
1	12-Dec-09	Female	low transmission	16-Jan-10	active follow	9	36.6	No
2	12-Dec-09	Female	low transmission	22-Feb-10	active follow	9	36.8	No
3	12-Dec-09	Female	low transmission	24-Mar-10	active follow	9	36.4	No
4	12-Dec-09	Female	low transmission	27-Mar-10	passive follow	4316		
5	12-Dec-09	Female	low transmission	27-Apr-10	active follow	9	36	No
6	12-Dec-09	Female	low transmission	25-May-10	active follow	9207	37	No
7	12-Dec-09	Female	low transmission	29-Jun-10	active follow	664	36.4	No
8	12-Dec-09	Female	low transmission	26-Jul-10	passive follow	9	36.3	Yes
9	12-Dec-09	Female	low transmission	29-Jul-10	active follow		36.3	Yes
10	12-Dec-09	Female	low transmission	24-Aug-10	active follow	3887	37.1	No

11	12-Dec-09	Female	low transmission	22-Sep-10	active follow	4897	35.5	No
12	12-Dec-09	Female	low transmission	22-Oct-10	active follow	865	36.3	No
13	12-Dec-09	Female	low transmission	15-Nov-10	passive follow	5751	36.9	Yes
14	12-Dec-09	Female	low transmission	28-Nov-10	active follow			
15	12-Dec-09	Female	low transmission	8-Dec-10	passive follow	9	37	Yes
1	14-Dec-09	Male	low transmission	18-Jan-10	active follow	9	36.3	No
2	14-Dec-09	Male	low transmission	23-Feb-10	active follow	9	36.1	No
3	14-Dec-09	Male	low transmission	24-Mar-10	active follow	9	36.2	No
4	14-Dec-09	Male	low transmission	29-Apr-10	active follow	9	35.8	No
5	14-Dec-09	Male	low transmission	29-May-10	active follow		36.3	No
6	14-Dec-09	Male	low transmission	1-Jul-10	active follow	2184	36.5	No
7	14-Dec-09	Male	low transmission	30-Jul-10	active follow		37.2	No
8	14-Dec-09	Male	low transmission	3-Aug-10	passive follow	1848	37.6	Yes
9	14-Dec-09	Male	low transmission	6-Sep-10	active follow	7018	36.3	No
10	14-Dec-09	Male	low transmission	11-Oct-10	active follow	937	35.6	No
11	14-Dec-09	Male	low transmission	12-Nov-10	active follow	915	36.2	No
12	14-Dec-09	Male	low transmission	25-Nov-10	passive follow	9	36	Yes
1	23-Nov-08	Male	high transmission	16-Dec-08	active follow	9	36.6	No
2	23-Nov-08	Male	high transmission	15-Jan-09	active follow	9	36.6	No
3	23-Nov-08	Male	high transmission	15-Feb-09	active follow	9	35.1	No
4	23-Nov-08	Male	high transmission	17-Mar-09	active follow	9	35.9	No
5	23-Nov-08	Male	high transmission	15-Apr-09	active follow	9	36.6	No
6	23-Nov-08	Male	high transmission	18-May-09	active follow	9	36	No
7	23-Nov-08	Male	high transmission	22-Jun-09	active follow	9	36.4	No
8	23-Nov-08	Male	high transmission	23-Jun-09	passive follow	9	36.3	Yes
9	23-Nov-08	Male	high transmission	21-Jul-09	passive follow		37.5	Yes
10	23-Nov-08	Male	high transmission	28-Jul-09	active follow	9		
11	23-Nov-08	Male	high transmission	29-Jul-09	active follow	9	36.4	No
12	23-Nov-08	Male	high transmission	27-Aug-09	active follow	160031	36.2	No
13	23-Nov-08	Male	high transmission	1-Sep-09	passive follow	6250	36.3	Yes
14	23-Nov-08	Male	high transmission	28-Sep-09	active follow	7165	35.8	No
15	23-Nov-08	Male	high transmission	2-Nov-09	active follow	30735	36.5	No
1	19-Dec-09	Female	low transmission	31-Jan-10	active follow	9	36.3	No
2	19-Dec-09	Female	low transmission	5-Mar-10	active follow	9	36.3	No
3	19-Dec-09	Female	low transmission	6-Apr-10	active follow	9	35.8	No
4	19-Dec-09	Female	low transmission	4-May-10	active follow	9	35.7	No
5	19-Dec-09	Female	low transmission	1-Jun-10	active follow	9	35.2	No
6	19-Dec-09	Female	low transmission	21-Jun-10	passive follow	9	37.1	Yes
7	19-Dec-09	Female	low transmission	7-Jul-10	active follow	9	36.3	No
8	19-Dec-09	Female	low transmission	2-Aug-10	passive follow	2363	39.1	Yes
9	19-Dec-09	Female	low transmission	13-Aug-10	active follow		35.9	No
10	19-Dec-09	Female	low transmission	17-Sep-10	active follow	9	36	No
11	19-Dec-09	Female	low transmission	22-Oct-10	active follow	9	36.5	No
12	19-Dec-09	Female	low transmission	26-Oct-10	passive follow	9	37.1	Yes
13	19-Dec-09	Female	low transmission	24-Nov-10	active follow	3832	36.6	No
1	22-Dec-09	Male	low transmission	21-Jan-10	active follow	9	36.4	No
2	22-Dec-09	Male	low transmission	16-Feb-10	passive follow	9	36.6	Yes

3	22-Dec-09	Male	low transmission	23-Feb-10	active follow	9	36.6	No
4	22-Dec-09	Male	low transmission	23-Mar-10	active follow	9	36.8	No
5	22-Dec-09	Male	low transmission	29-Mar-10	passive follow	9	37.4	Yes
6	22-Dec-09	Male	low transmission	22-Apr-10	active follow	9	36.6	No
7	22-Dec-09	Male	low transmission	26-May-10	active follow	538	36.3	Yes
8	22-Dec-09	Male	low transmission	23-Jun-10	active follow	9	36.1	No
9	22-Dec-09	Male	low transmission	30-Jul-10	active follow	9088	36.7	No
10	22-Dec-09	Male	low transmission	5-Sep-10	active follow		36.4	No
11	22-Dec-09	Male	low transmission	11-Oct-10	active follow	9	37.2	Yes
12	22-Dec-09	Male	low transmission	11-Nov-10	active follow	9	36.9	No
13	22-Dec-09	Male	low transmission	23-Nov-10	passive follow	9		
14	22-Dec-09	Male	low transmission	6-Dec-10	active follow	9	37.1	No
1	29-Dec-09	Female	low transmission	27-Jan-10	active follow	9	35.3	No
2	29-Dec-09	Female	low transmission	3-Mar-10	active follow	9	36.4	No
3	29-Dec-09	Female	low transmission	11-Mar-10	passive follow	2		
4	29-Dec-09	Female	low transmission	6-Apr-10	active follow	9	36.3	No
5	29-Dec-09	Female	low transmission	15-May-10	active follow	829	36	No
6	29-Dec-09	Female	low transmission	15-Jun-10	passive follow		37	Yes
7	29-Dec-09	Female	low transmission	3-Jul-10	passive follow	9	36	Yes
8	29-Dec-09	Female	low transmission	16-Jul-10	active follow	9	37	No
9	29-Dec-09	Female	low transmission	26-Jul-10	passive follow	95312	38.2	Yes
10	29-Dec-09	Female	low transmission	15-Aug-10	passive follow	564	36.4	Yes
11	29-Dec-09	Female	low transmission	22-Aug-10	active follow			
12	29-Dec-09	Female	low transmission	5-Sep-10	passive follow	1645	36.7	Yes
13	29-Dec-09	Female	low transmission	28-Sep-10	active follow	18380	36.4	Yes
14	29-Dec-09	Female	low transmission	20-Oct-10	passive follow	2709	36.2	Yes
15	29-Dec-09	Female	low transmission	1-Nov-10	active follow	7154	36.2	Yes
16	29-Dec-09	Female	low transmission	1-Dec-10	active follow	6983	35.2	Yes
17	29-Dec-09	Female	low transmission	18-Dec-10	passive follow	2347	36	Yes
1	31-Dec-09	Female	low transmission	29-Jan-10	active follow	9	36.1	No
2	31-Dec-09	Female	low transmission	29-Jan-10	active follow	9	36	No
3	31-Dec-09	Female	low transmission	3-Mar-10	active follow	9	36.4	No
4	31-Dec-09	Female	low transmission	3-Mar-10	active follow	9	36.2	No
5	31-Dec-09	Female	low transmission	8-Apr-10	active follow	9	35.8	No
6	31-Dec-09	Female	low transmission	8-Apr-10	active follow	9	36.3	No
7	31-Dec-09	Female	low transmission	23-Apr-10	passive follow		36.1	Yes
8	31-Dec-09	Female	low transmission	23-Apr-10	passive follow	9	36.4	Yes
9	31-Dec-09	Female	low transmission	8-May-10	active follow	9	35.7	No
10	31-Dec-09	Female	low transmission	8-May-10	active follow	9	36.9	No
11	31-Dec-09	Female	low transmission	8-Jun-10	active follow	9	36.7	No
12	31-Dec-09	Female	low transmission	8-Jun-10	active follow	9	36.2	No
13	31-Dec-09	Female	low transmission	16-Jun-10	passive follow			No
14	31-Dec-09	Female	low transmission	6-Jul-10	active follow	9		No
15	31-Dec-09	Female	low transmission	6-Jul-10	active follow	9	36.7	No
16	31-Dec-09	Female	low transmission	28-Jul-10	passive follow	1343	35.7	No
17	31-Dec-09	Female	low transmission	28-Jul-10	passive follow	9	35.8	Yes
18	31-Dec-09	Female	low transmission	6-Aug-10	active follow	9	36	No

19	31-Dec-09	Female	low transmission	6-Aug-10	active follow	9	35.7	No
20	31-Dec-09	Female	low transmission	1-Sep-10	active follow	402	35.7	Yes
21	31-Dec-09	Female	low transmission	1-Sep-10	active follow	2177	35.7	Yes
22	31-Dec-09	Female	low transmission	22-Sep-10	active follow	51376	36	Yes
23	31-Dec-09	Female	low transmission	22-Sep-10	active follow	9	35.7	No
24	31-Dec-09	Female	low transmission	20-Oct-10	active follow	9	35.7	Yes
25	31-Dec-09	Female	low transmission	20-Oct-10	active follow	3378	35.7	Yes
26	31-Dec-09	Female	low transmission	16-Nov-10	active follow	1044	36.1	No
27	31-Dec-09	Female	low transmission	16-Nov-10	active follow	9524	36.7	No
28	31-Dec-09	Female	low transmission	14-Dec-10	active follow	977	36	No
29	31-Dec-09	Female	low transmission	14-Dec-10	active follow	17255	35.7	No
1	28-Dec-09	Male	low transmission	28-Jan-10	active follow	9	36.6	No
2	28-Dec-09	Male	low transmission	23-Feb-10	active follow	9	36.5	No
3	28-Dec-09	Male	low transmission	25-Mar-10	active follow	9	36.5	No
4	28-Dec-09	Male	low transmission	22-Apr-10	active follow		36.5	No
5	28-Dec-09	Male	low transmission	28-May-10	active follow	9	36.6	No
6	28-Dec-09	Male	low transmission	30-Jun-10	active follow	761	36	No
7	28-Dec-09	Male	low transmission	27-Jul-10	active follow	1565	38.9	Yes
8	28-Dec-09	Male	low transmission	26-Aug-10	active follow	9	37	No
9	28-Dec-09	Male	low transmission	28-Sep-10	active follow	9	36.5	No
10	28-Dec-09	Male	low transmission	4-Nov-10	active follow			
11	28-Dec-09	Male	low transmission	7-Dec-10	active follow			
1	28-Dec-09	Male	low transmission	30-Jan-10	active follow	9	36.8	No
2	28-Dec-09	Male	low transmission	5-Mar-10	active follow	9	36.3	No
3	28-Dec-09	Male	low transmission	15-Mar-10	passive follow	9	36.3	No
4	28-Dec-09	Male	low transmission	9-Apr-10	active follow	9	36.6	No
5	28-Dec-09	Male	low transmission	14-May-10	active follow	9	36.3	No
6	28-Dec-09	Male	low transmission	17-Jun-10	passive follow	16789	36.3	Yes
7	28-Dec-09	Male	low transmission	21-Jun-10	active follow	9	35.7	No
8	28-Dec-09	Male	low transmission	2-Jul-10	passive follow	9	36.8	No
9	28-Dec-09	Male	low transmission	27-Jul-10	active follow	1419	36.3	No
10	28-Dec-09	Male	low transmission	6-Aug-10	passive follow	146	36	No
11	28-Dec-09	Male	low transmission	26-Aug-10	active follow	9	35.6	No
12	28-Dec-09	Male	low transmission	30-Sep-10	active follow	9	35.8	No
13	28-Dec-09	Male	low transmission	31-Oct-10	active follow	9	36.3	No
14	28-Dec-09	Male	low transmission	11-Nov-10	passive follow	183253	37.7	Yes
15	28-Dec-09	Male	low transmission	3-Dec-10	active follow	9	36.4	No
16	28-Dec-09	Male	low transmission	21-Dec-10	passive follow	9	36.5	No
1	30-Dec-09	Male	low transmission	2-Feb-10	active follow	9	36.8	No
2	30-Dec-09	Male	low transmission	9-Mar-10	active follow	9	36.5	No
3	30-Dec-09	Male	low transmission	14-Apr-10	active follow	9	36.2	No
4	30-Dec-09	Male	low transmission	13-May-10	active follow	9	37	No
5	30-Dec-09	Male	low transmission	9-Jun-10	active follow	2119	37.3	No
6	30-Dec-09	Male	low transmission	10-Jul-10	active follow	9	36.9	No
7	30-Dec-09	Male	low transmission	16-Aug-10	active follow			
8	30-Dec-09	Male	low transmission	22-Sep-10	active follow			
9	30-Dec-09	Male	low transmission	5-Oct-10	passive follow	9	35.9	No

10	30-Dec-09	Male	low transmission	19-Oct-10	active follow	9	36.4	No
11	30-Dec-09	Male	low transmission	25-Nov-10	passive follow	37869	38.7	Yes
12	30-Dec-09	Male	low transmission	25-Dec-10	active follow			
1	4-Jan-10	Male	high transmission	6-Feb-10	active follow	9	36.5	No
2	4-Jan-10	Male	high transmission	14-Mar-10	active follow	9	36.6	No
3	4-Jan-10	Male	high transmission	15-Apr-10	active follow	9	36.5	No
4	4-Jan-10	Male	high transmission	11-May-10	active follow	9	37	No
5	4-Jan-10	Male	high transmission	9-Jun-10	active follow	9	36.8	No
6	4-Jan-10	Male	high transmission	6-Jul-10	active follow	9	36.9	No
7	4-Jan-10	Male	high transmission	25-Jul-10	passive follow	9	35.7	No
8	4-Jan-10	Male	high transmission	5-Aug-10	active follow	9	35.6	No
9	4-Jan-10	Male	high transmission	3-Sep-10	active follow	128765	35.9	No
10	4-Jan-10	Male	high transmission	19-Sep-10	passive follow	9	35.7	Yes
11	4-Jan-10	Male	high transmission	7-Oct-10	active follow	9	35.7	No
12	4-Jan-10	Male	high transmission	1-Nov-10	active follow	126976	36	Yes
13	4-Jan-10	Male	high transmission	30-Nov-10	active follow	9	35.7	No
14	4-Jan-10	Male	high transmission	27-Dec-10	active follow	158517	36.5	Yes
1	5-Jan-10	Male	high transmission	8-Feb-10	active follow	9	36.3	No
2	5-Jan-10	Male	high transmission	17-Mar-10	active follow			
3	5-Jan-10	Male	high transmission	16-Apr-10	active follow			
4	5-Jan-10	Male	high transmission	16-May-10	active follow			
5	5-Jan-10	Male	high transmission	3-Jun-10	active follow	9	36.2	No
6	5-Jan-10	Male	high transmission	5-Jul-10	active follow	9	36.3	No
7	5-Jan-10	Male	high transmission	24-Jul-10	passive follow	9	38	Yes
8	5-Jan-10	Male	high transmission	5-Aug-10	active follow	9	35.6	No
9	5-Jan-10	Male	high transmission	6-Sep-10	active follow	9	36.3	No
10	5-Jan-10	Male	high transmission	5-Oct-10	active follow	14406	38.8	Yes
11	5-Jan-10	Male	high transmission	5-Nov-10	active follow	3033	36.9	No
12	5-Jan-10	Male	high transmission	11-Nov-10	passive follow		37.9	Yes
13	5-Jan-10	Male	high transmission	10-Dec-10	active follow	12977	36.9	No
1	5-Jan-10	Male	high transmission	11-Feb-10	active follow		36.4	No
2	5-Jan-10	Male	high transmission	15-Mar-10	active follow	9	36.5	No
3	5-Jan-10	Male	high transmission	19-Apr-10	active follow	9	36.6	No
4	5-Jan-10	Male	high transmission	10-May-10	passive follow	9	37	Yes
5	5-Jan-10	Male	high transmission	24-May-10	active follow		36.1	No
6	5-Jan-10	Male	high transmission	26-Jun-10	active follow	9	36.5	No
7	5-Jan-10	Male	high transmission	30-Jul-10	active follow	9	36.7	No
8	5-Jan-10	Male	high transmission	19-Aug-10	passive follow	16925	38.4	Yes
9	5-Jan-10	Male	high transmission	3-Sep-10	active follow	9	36.2	No
10	5-Jan-10	Male	high transmission	8-Oct-10	active follow	1131	36.8	No
11	5-Jan-10	Male	high transmission	19-Oct-10	passive follow	3771	36	Yes
12	5-Jan-10	Male	high transmission	10-Nov-10	active follow	9	36	Yes
13	5-Jan-10	Male	high transmission	17-Dec-10	active follow			
1	4-Jan-10	Female	high transmission	22-Jan-10	passive follow	9	36.3	No
2	4-Jan-10	Female	high transmission	10-Feb-10	active follow	9	36.3	No
3	4-Jan-10	Female	high transmission	20-Mar-10	active follow	9	36.1	No
4	4-Jan-10	Female	high transmission	6-Apr-10	passive follow	9	36	Yes

5	4-Jan-10	Female	high transmission	27-Apr-10	active follow	9	36	Yes
6	4-Jan-10	Female	high transmission	2-Jun-10	active follow	9	37	No
7	4-Jan-10	Female	high transmission	9-Jul-10	active follow	6666	37.6	No
8	4-Jan-10	Female	high transmission	15-Aug-10	active follow	2420	36.5	No
9	4-Jan-10	Female	high transmission	30-Aug-10	passive follow	9	36	Yes
10	4-Jan-10	Female	high transmission	20-Sep-10	active follow	2040	35.6	No
11	4-Jan-10	Female	high transmission	22-Oct-10	active follow	865	35.8	No
12	4-Jan-10	Female	high transmission	26-Nov-10	active follow	1442	35.8	No
13	4-Jan-10	Female	high transmission	29-Nov-10	passive follow		37.4	Yes
14	4-Jan-10	Female	high transmission	27-Dec-10	active follow	4968	36	No
15	4-Jan-10	Female	high transmission	30-Dec-10	passive follow	1439	37.1	Yes
1	2-Jan-10	Male	high transmission	8-Feb-10	active follow	9	36.5	No
2	2-Jan-10	Male	high transmission	14-Mar-10	active follow	9	36.8	No
3	2-Jan-10	Male	high transmission	15-Apr-10	active follow	9	36.5	No
4	2-Jan-10	Male	high transmission	13-May-10	active follow	9	36.3	No
5	2-Jan-10	Male	high transmission	15-Jun-10	active follow	9	35.7	No
6	2-Jan-10	Male	high transmission	13-Jul-10	active follow	20943	36.8	No
7	2-Jan-10	Male	high transmission	20-Jul-10	passive follow	19953	38.1	Yes
8	2-Jan-10	Male	high transmission	10-Aug-10	active follow	418	36.1	Yes
9	2-Jan-10	Male	high transmission	11-Aug-10	active follow		36.3	No
10	2-Jan-10	Male	high transmission	7-Sep-10	active follow	9	36.3	No
11	2-Jan-10	Male	high transmission	7-Oct-10	active follow	9	36.5	No
12	2-Jan-10	Male	high transmission	9-Nov-10	active follow	9	36.3	No
13	2-Jan-10	Male	high transmission	30-Nov-10	passive follow	12225	37.1	Yes
14	2-Jan-10	Male	high transmission	7-Dec-10	active follow	9	36.5	No
1	8-Jan-10	Male	high transmission	13-Feb-10	active follow	9	36.8	No
2	8-Jan-10	Male	high transmission	22-Mar-10	active follow	9	36.2	No
3	8-Jan-10	Male	high transmission	1-May-10	active follow	72	36.3	No
4	8-Jan-10	Male	high transmission	5-Jun-10	active follow	9	35.8	No
5	8-Jan-10	Male	high transmission	10-Jul-10	active follow	9	36.7	No
6	8-Jan-10	Male	high transmission	14-Aug-10	active follow	21379	36.2	No
7	8-Jan-10	Male	high transmission	17-Sep-10	active follow	2862	36.7	No
8	8-Jan-10	Male	high transmission	4-Oct-10	passive follow	1067	37	Yes
9	8-Jan-10	Male	high transmission	27-Oct-10	active follow	9	35.6	No
10	8-Jan-10	Male	high transmission	24-Nov-10	active follow	9	36.2	No
11	8-Jan-10	Male	high transmission	21-Dec-10	active follow	9	35.8	No
12	8-Jan-10	Male	high transmission	30-Dec-10	passive follow	9	38.1	Yes
1	9-Jan-10	Female	high transmission	19-Feb-10	passive follow	9	36.9	Yes
2	9-Jan-10	Female	high transmission	16-Mar-10	passive follow	9	36.8	Yes
3	9-Jan-10	Female	high transmission	25-Mar-10	active follow	9	36.4	No
4	9-Jan-10	Female	high transmission	22-Apr-10	active follow	9	35.8	No
5	9-Jan-10	Female	high transmission	30-Apr-10	passive follow	9	35.8	No
6	9-Jan-10	Female	high transmission	24-May-10	active follow		36	No
7	9-Jan-10	Female	high transmission	28-Jun-10	active follow	9	36.2	Yes
8	9-Jan-10	Female	high transmission	22-Jul-10	passive follow	12235	38	Yes
9	9-Jan-10	Female	high transmission	4-Aug-10	active follow	9	36.9	No
10	9-Jan-10	Female	high transmission	16-Aug-10	passive follow	9	36.5	Yes

11	9-Jan-10	Female	high transmission	2-Sep-10	active follow			
12	9-Jan-10	Female	high transmission	30-Sep-10	active follow	9	36.4	Yes
13	9-Jan-10	Female	high transmission	27-Oct-10	active follow	9	36	No
14	9-Jan-10	Female	high transmission	15-Nov-10	passive follow	9	36.8	Yes
15	9-Jan-10	Female	high transmission	2-Dec-10	active follow	382	36.9	No
1	13-Jan-10	Female	high transmission	12-Feb-10	active follow	9	36.5	No
2	13-Jan-10	Female	high transmission	12-Feb-10	active follow	9	36.1	No
3	13-Jan-10	Female	high transmission	8-Mar-10	passive follow	9	36.8	Yes
4	13-Jan-10	Female	high transmission	22-Mar-10	active follow			
5	13-Jan-10	Female	high transmission	22-Mar-10	active follow	9	36.3	No
6	13-Jan-10	Female	high transmission	26-Apr-10	active follow			
7	13-Jan-10	Female	high transmission	26-Apr-10	active follow	9	36.3	No
8	13-Jan-10	Female	high transmission	28-May-10	active follow	9	36.1	No
9	13-Jan-10	Female	high transmission	2-Jun-10	active follow			
10	13-Jan-10	Female	high transmission	30-Jun-10	active follow			
11	13-Jan-10	Female	high transmission	5-Jul-10	active follow	9	36.4	No
12	13-Jan-10	Female	high transmission	6-Aug-10	active follow			
13	13-Jan-10	Female	high transmission	6-Aug-10	active follow	2849	35.8	No
14	13-Jan-10	Female	high transmission	8-Sep-10	active follow	9	35.8	No
15	13-Jan-10	Female	high transmission	10-Sep-10	active follow			
16	13-Jan-10	Female	high transmission	28-Sep-10	passive follow	3566	38.4	Yes
17	13-Jan-10	Female	high transmission	7-Oct-10	active follow	9	36.6	No
18	13-Jan-10	Female	high transmission	14-Oct-10	active follow			
19	13-Jan-10	Female	high transmission	3-Nov-10	active follow	9	36.6	No
20	13-Jan-10	Female	high transmission	10-Nov-10	active follow			
21	13-Jan-10	Female	high transmission	1-Dec-10	active follow	9	36.4	No
22	13-Jan-10	Female	high transmission	8-Dec-10	active follow			
23	13-Jan-10	Female	high transmission	30-Dec-10	active follow	34171	37	No
24	13-Jan-10	Female	high transmission	4-Jan-11	active follow			
1	16-Jan-10	Female	high transmission	20-Feb-10	active follow	9	36.3	No
2	16-Jan-10	Female	high transmission	26-Mar-10	active follow	9	36.5	No
3	16-Jan-10	Female	high transmission	28-Apr-10	active follow	9	36	No
4	16-Jan-10	Female	high transmission	10-May-10	passive follow	9	37.1	No
5	16-Jan-10	Female	high transmission	24-May-10	active follow		36.1	No
6	16-Jan-10	Female	high transmission	23-Jun-10	active follow	9	36.4	No
7	16-Jan-10	Female	high transmission	20-Jul-10	active follow	9	36.4	No
8	16-Jan-10	Female	high transmission	18-Aug-10	active follow	31450	37.5	Yes
9	16-Jan-10	Female	high transmission	20-Aug-10	passive follow	17829	36.6	Yes
10	16-Jan-10	Female	high transmission	13-Sep-10	active follow	83773	36	Yes
11	16-Jan-10	Female	high transmission	12-Oct-10	active follow	810	36	No
12	16-Jan-10	Female	high transmission	23-Oct-10	passive follow	9	37.6	Yes
13	16-Jan-10	Female	high transmission	8-Nov-10	active follow	9	36.7	No
14	16-Jan-10	Female	high transmission	6-Dec-10	active follow	9	36.7	No
15	16-Jan-10	Female	high transmission	6-Jan-11	active follow	9	36.2	No
1	15-Jan-10	Male	high transmission	23-Feb-10	active follow	9	36.8	No
2	15-Jan-10	Male	high transmission	25-Mar-10	active follow	9	36.6	No
3	15-Jan-10	Male	high transmission	28-Apr-10	active follow	9	36.5	No

4	15-Jan-10	Male	high transmission	27-May-10	active follow	141	36	No
5	15-Jan-10	Male	high transmission	29-Jun-10	active follow	197	36.3	No
6	15-Jan-10	Male	high transmission	30-Jul-10	active follow	9	36.4	No
7	15-Jan-10	Male	high transmission	13-Aug-10	passive follow		37.6	No
8	15-Jan-10	Male	high transmission	6-Sep-10	active follow	9	36.2	No
9	15-Jan-10	Male	high transmission	13-Sep-10	passive follow	101510		
10	15-Jan-10	Male	high transmission	8-Oct-10	active follow	9	36.6	No
11	15-Jan-10	Male	high transmission	27-Oct-10	passive follow	83199	37.2	No
12	15-Jan-10	Male	high transmission	12-Nov-10	active follow	16216	36	No
13	15-Jan-10	Male	high transmission	16-Dec-10	active follow	9	36	No
1	16-Jan-10	Female	high transmission	18-Feb-10	active follow	9	36.5	No
2	16-Jan-10	Female	high transmission	17-Mar-10	active follow	9	36.6	No
3	16-Jan-10	Female	high transmission	21-Apr-10	active follow	9	36.1	No
4	16-Jan-10	Female	high transmission	27-May-10	active follow	9	36.5	No
5	16-Jan-10	Female	high transmission	3-Jul-10	active follow			
6	16-Jan-10	Female	high transmission	12-Aug-10	active follow		36.5	No
7	16-Jan-10	Female	high transmission	16-Sep-10	active follow		37.2	Yes
8	16-Jan-10	Female	high transmission	21-Sep-10	passive follow	1339	37.1	Yes
9	16-Jan-10	Female	high transmission	15-Oct-10	active follow	9	37	No
10	16-Jan-10	Female	high transmission	18-Nov-10	active follow	171	36.8	No
11	16-Jan-10	Female	high transmission	13-Dec-10	active follow	10262	37.1	Yes
12	16-Jan-10	Female	high transmission	12-Jan-11	active follow		36.3	No
1	24-Jan-10	Male	high transmission	26-Feb-10	active follow	9	37	No
2	24-Jan-10	Male	high transmission	5-Apr-10	active follow	9	36.3	No
3	24-Jan-10	Male	high transmission	11-May-10	active follow	9	36.5	No
4	24-Jan-10	Male	high transmission	16-Jun-10	active follow	3116	36.6	No
5	24-Jan-10	Male	high transmission	14-Jul-10	active follow	8406	36.1	No
6	24-Jan-10	Male	high transmission	21-Jul-10	passive follow		36.6	Yes
7	24-Jan-10	Male	high transmission	28-Aug-10	active follow		35.4	No
8	24-Jan-10	Male	high transmission	29-Sep-10	active follow	9	36.8	No
9	24-Jan-10	Male	high transmission	16-Oct-10	passive follow	3164	36.6	Yes
10	24-Jan-10	Male	high transmission	10-Nov-10	active follow	9	36.7	No
11	24-Jan-10	Male	high transmission	30-Nov-10	passive follow	6936	37.9	Yes
12	24-Jan-10	Male	high transmission	9-Dec-10	active follow	9	36.8	No
13	24-Jan-10	Male	high transmission	15-Jan-11	active follow			
1	23-Jan-10	Female	high transmission	24-Feb-10	active follow	9	36.4	No
2	23-Jan-10	Female	high transmission	27-Feb-10	passive follow	9	36.3	Yes
3	23-Jan-10	Female	high transmission	24-Mar-10	active follow	9	36.4	Yes
4	23-Jan-10	Female	high transmission	24-Apr-10	active follow	9	37.2	No
5	23-Jan-10	Female	high transmission	13-May-10	passive follow		36.8	Yes
6	23-Jan-10	Female	high transmission	14-May-10	passive follow	9		
7	23-Jan-10	Female	high transmission	25-May-10	active follow		35.5	No
8	23-Jan-10	Female	high transmission	24-Jun-10	active follow	1089	35.5	No
9	23-Jan-10	Female	high transmission	21-Jul-10	active follow	6058	35.7	No
10	23-Jan-10	Female	high transmission	2-Aug-10	passive follow		35.7	No
11	23-Jan-10	Female	high transmission	18-Aug-10	active follow	9	35.7	No
12	23-Jan-10	Female	high transmission	14-Sep-10	active follow	6851	36	No

13	23-Jan-10	Female	high transmission	15-Sep-10	passive follow	1878	35.7	Yes
14	23-Jan-10	Female	high transmission	12-Oct-10	active follow	9	36	No
15	23-Jan-10	Female	high transmission	9-Nov-10	active follow	9	36	No
16	23-Jan-10	Female	high transmission	10-Nov-10	passive follow	9	36	No
17	23-Jan-10	Female	high transmission	28-Nov-10	passive follow	80337	35.7	Yes
18	23-Jan-10	Female	high transmission	16-Dec-10	active follow			
19	23-Jan-10	Female	high transmission	21-Jan-11	active follow			
1	5-Dec-09	Female	low transmission	11-Jan-10	active follow			No
2	5-Dec-09	Female	low transmission	10-Feb-10	active follow			No
3	5-Dec-09	Female	low transmission	12-Mar-10	active follow	9	36.7	No
4	5-Dec-09	Female	low transmission	18-Mar-10	passive follow	9877	38.3	Yes
5	5-Dec-09	Female	low transmission	16-Apr-10	active follow	4963	36.4	No
6	5-Dec-09	Female	low transmission	22-May-10	active follow		36.4	No
7	5-Dec-09	Female	low transmission	23-Jun-10	active follow	4791	36.4	No
8	5-Dec-09	Female	low transmission	19-Jul-10	passive follow	1673	37.5	Yes
9	5-Dec-09	Female	low transmission	7-Aug-10	passive follow	48649	36.1	Yes
10	5-Dec-09	Female	low transmission	28-Aug-10	active follow	1643	36.1	No
11	5-Dec-09	Female	low transmission	15-Sep-10	passive follow	6159	36	Yes
12	5-Dec-09	Female	low transmission	30-Sep-10	active follow	9	36	No
13	5-Dec-09	Female	low transmission	11-Oct-10	passive follow	22196	38.2	Yes
14	5-Dec-09	Female	low transmission	25-Oct-10	active follow	9	36.7	No
15	5-Dec-09	Female	low transmission	23-Nov-10	active follow		37.9	Yes
1	28-Jan-10	Male	high transmission	29-Jan-10	passive follow		38.5	No
2	28-Jan-10	Male	high transmission	24-Feb-10	active follow	9	36.6	No
3	28-Jan-10	Male	high transmission	24-Mar-10	active follow	9	36.6	No
4	28-Jan-10	Male	high transmission	21-Apr-10	active follow	9	36.3	Yes
5	28-Jan-10	Male	high transmission	20-May-10	active follow	9	36.7	Yes
6	28-Jan-10	Male	high transmission	31-May-10	passive follow	9	35.4	Yes
7	28-Jan-10	Male	high transmission	16-Jun-10	active follow	9		No
8	28-Jan-10	Male	high transmission	14-Jul-10	active follow	1005	35.7	No
9	28-Jan-10	Male	high transmission	10-Aug-10	active follow		35.7	No
10	28-Jan-10	Male	high transmission	14-Aug-10	passive follow	9	35.7	Yes
11	28-Jan-10	Male	high transmission	7-Sep-10	active follow	9	35.7	No
12	28-Jan-10	Male	high transmission	5-Oct-10	active follow	9	36	No
13	28-Jan-10	Male	high transmission	20-Oct-10	passive follow	9	35.7	Yes
14	28-Jan-10	Male	high transmission	4-Nov-10	active follow	9	36	No
15	28-Jan-10	Male	high transmission	30-Nov-10	active follow	9	35.7	No
16	28-Jan-10	Male	high transmission	1-Jan-11	active follow	36915	36	No
17	28-Jan-10	Male	high transmission	19-Jan-11	passive follow	6931	36.7	Yes
1	26-Jan-10	Female	high transmission	27-Feb-10	active follow	9	36.6	No
2	26-Jan-10	Female	high transmission	9-Apr-10	active follow	9	36.3	No
3	26-Jan-10	Female	high transmission	30-Apr-10	passive follow	9	36.7	No
4	26-Jan-10	Female	high transmission	16-May-10	active follow			
5	26-Jan-10	Female	high transmission	9-Jun-10	passive follow	3395	39.3	Yes
6	26-Jan-10	Female	high transmission	19-Jun-10	active follow	9	36.8	No
7	26-Jan-10	Female	high transmission	21-Jul-10	active follow	686	36.1	No
8	26-Jan-10	Female	high transmission	26-Jul-10	passive follow	159861	39.2	Yes

9	26-Jan-10	Female	high transmission	18-Aug-10	active follow	9	36	No
10	26-Jan-10	Female	high transmission	25-Aug-10	passive follow	555535	37.3	Yes
11	26-Jan-10	Female	high transmission	25-Sep-10	active follow	6218	36.6	No
12	26-Jan-10	Female	high transmission	25-Oct-10	active follow	75604	36.8	No
13	26-Jan-10	Female	high transmission	29-Nov-10	active follow	22767	36.7	No
14	26-Jan-10	Female	high transmission	3-Dec-10	passive follow	27755	37.8	Yes
15	26-Jan-10	Female	high transmission	3-Jan-11	active follow	6490	36.3	No
1	28-Jan-10	Male	high transmission	28-Feb-10	active follow	9	36.6	No
2	28-Jan-10	Male	high transmission	9-Apr-10	active follow	9	36.8	No
3	28-Jan-10	Male	high transmission	14-May-10	active follow	9	37	No
4	28-Jan-10	Male	high transmission	19-Jun-10	active follow	9	36.6	No
5	28-Jan-10	Male	high transmission	24-Jul-10	active follow	9	36.6	No
6	28-Jan-10	Male	high transmission	12-Aug-10	passive follow	1353	36.8	Yes
7	28-Jan-10	Male	high transmission	26-Aug-10	active follow	9	36	No
8	28-Jan-10	Male	high transmission	27-Sep-10	active follow	903	36	No
9	28-Jan-10	Male	high transmission	30-Oct-10	active follow	3271	36.7	No
10	28-Jan-10	Male	high transmission	4-Dec-10	active follow	9	37	No
11	28-Jan-10	Male	high transmission	9-Jan-11	active follow	9	36.6	No
13	28-Jan-10	Male	high transmission	28-Jan-11	passive follow	2952	37.4	Yes
1	18-Nov-08	Male	high transmission	16-Dec-08	active follow	9	36.3	No
2	18-Nov-08	Male	high transmission	20-Jan-09	active follow		36.3	No
3	18-Nov-08	Male	high transmission	21-Feb-09	active follow	9	36.5	No
4	18-Nov-08	Male	high transmission	31-Mar-09	active follow	9	36	No
5	18-Nov-08	Male	high transmission	6-May-09	active follow	9	36.4	No
6	18-Nov-08	Male	high transmission	10-Jun-09	active follow	9	36.6	No
7	18-Nov-08	Male	high transmission	24-Jun-09	passive follow	556	37.1	Yes
8	18-Nov-08	Male	high transmission	15-Jul-09	active follow	9	36.4	No
9	18-Nov-08	Male	high transmission	14-Aug-09	active follow	91	36.4	No
10	18-Nov-08	Male	high transmission	19-Aug-09	passive follow	9	39.1	Yes
11	18-Nov-08	Male	high transmission	20-Sep-09	active follow			
12	18-Nov-08	Male	high transmission	26-Oct-09	active follow	9	36.2	No
13	18-Nov-08	Male	high transmission	10-Nov-09	passive follow	63780		
1	29-Jan-10	Female	high transmission	7-Mar-10	active follow	9	36.4	No
2	29-Jan-10	Female	high transmission	25-Mar-10	passive follow	9	37.9	Yes
3	29-Jan-10	Female	high transmission	9-Apr-10	active follow	9	35.8	No
4	29-Jan-10	Female	high transmission	23-Apr-10	passive follow		36.8	Yes
5	29-Jan-10	Female	high transmission	13-May-10	active follow	9	36.4	No
6	29-Jan-10	Female	high transmission	12-Jun-10	active follow	9	35.4	No
7	29-Jan-10	Female	high transmission	13-Jul-10	active follow	9	35	No
8	29-Jan-10	Female	high transmission	12-Aug-10	active follow	9	36.2	No
9	29-Jan-10	Female	high transmission	7-Sep-10	active follow	9	36.5	No
10	29-Jan-10	Female	high transmission	7-Oct-10	active follow	4344	36.5	Yes
11	29-Jan-10	Female	high transmission	4-Nov-10	active follow	247	36	No
12	29-Jan-10	Female	high transmission	30-Nov-10	active follow	557	36.8	No
13	29-Jan-10	Female	high transmission	28-Dec-10	active follow	524	36.2	No
14	29-Jan-10	Female	high transmission	25-Jan-11	active follow	713	36.6	Yes
1	26-Jan-10	Female	high transmission	2-Mar-10	active follow	9	36.6	No

2	26-Jan-10	Female	high transmission	2-Apr-10	active follow	9	36.3	No
3	26-Jan-10	Female	high transmission	5-Apr-10	passive follow	9	37.3	No
4	26-Jan-10	Female	high transmission	4-May-10	active follow	9	37.1	No
5	26-Jan-10	Female	high transmission	2-Jun-10	active follow	9	36	No
6	26-Jan-10	Female	high transmission	4-Jul-10	active follow	9	36.3	No
7	26-Jan-10	Female	high transmission	6-Aug-10	active follow	22856	36.5	No
8	26-Jan-10	Female	high transmission	9-Sep-10	active follow		36	No
9	26-Jan-10	Female	high transmission	8-Oct-10	active follow	2859	36	No
10	26-Jan-10	Female	high transmission	12-Oct-10	passive follow	317411	37.1	No
11	26-Jan-10	Female	high transmission	12-Nov-10	active follow	9	36	No
12	26-Jan-10	Female	high transmission	16-Dec-10	active follow	9	36.1	No
13	26-Jan-10	Female	high transmission	18-Jan-11	active follow	7002	36	No
1	31-Jan-10	Male	high transmission	9-Mar-10	active follow		36.5	No
2	31-Jan-10	Male	high transmission	6-Apr-10	passive follow	9	36.6	Yes
3	31-Jan-10	Male	high transmission	8-May-10	active follow	9	36.3	No
4	31-Jan-10	Male	high transmission	9-Jun-10	active follow	443	36	No
5	31-Jan-10	Male	high transmission	12-Jul-10	active follow	540	36.1	No
6	31-Jan-10	Male	high transmission	7-Aug-10	passive follow		36.2	Yes
7	31-Jan-10	Male	high transmission	18-Aug-10	active follow	9	36.3	No
8	31-Jan-10	Male	high transmission	16-Sep-10	active follow	9	36.6	No
9	31-Jan-10	Male	high transmission	27-Sep-10	passive follow	9	36	Yes
10	31-Jan-10	Male	high transmission	13-Oct-10	active follow	2128	38.4	Yes
11	31-Jan-10	Male	high transmission	16-Nov-10	active follow	9	36.2	No
12	31-Jan-10	Male	high transmission	20-Dec-10	active follow	9	36.2	No
13	31-Jan-10	Male	high transmission	29-Dec-10	passive follow	9	39.3	Yes
14	31-Jan-10	Male	high transmission	19-Jan-11	active follow	9	36.5	No
1	27-Jan-10	Female	high transmission	3-Mar-10	active follow	9	36.6	No
2	27-Jan-10	Female	high transmission	10-Mar-10	passive follow	9	36.1	Yes
3	27-Jan-10	Female	high transmission	8-Apr-10	active follow	778	36.5	No
4	27-Jan-10	Female	high transmission	26-Apr-10	passive follow	497	37.4	Yes
5	27-Jan-10	Female	high transmission	11-May-10	active follow	9	37.1	No
6	27-Jan-10	Female	high transmission	17-May-10	passive follow		38.3	Yes
7	27-Jan-10	Female	high transmission	18-May-10	passive follow	10383		
8	27-Jan-10	Female	high transmission	10-Jun-10	active follow	9	36	No
9	27-Jan-10	Female	high transmission	11-Jun-10	passive follow	9	37.6	Yes
10	27-Jan-10	Female	high transmission	8-Jul-10	active follow	855	36.2	No
11	27-Jan-10	Female	high transmission	20-Jul-10	passive follow	27553	38.3	Yes
12	27-Jan-10	Female	high transmission	31-Jul-10	active follow	17657	38.4	Yes
13	27-Jan-10	Female	high transmission	27-Aug-10	active follow		39.3	Yes
14	27-Jan-10	Female	high transmission	29-Sep-10	active follow	9	35.5	No
15	27-Jan-10	Female	high transmission	2-Nov-10	active follow	9	36.2	No
16	27-Jan-10	Female	high transmission	8-Nov-10	passive follow	9	37.9	No
17	27-Jan-10	Female	high transmission	29-Nov-10	active follow	9	36.6	No
18	27-Jan-10	Female	high transmission	4-Jan-11	active follow	9	36.6	No
1	30-Jan-10	Female	high transmission	20-Feb-10	passive follow		36.3	Yes
2	30-Jan-10	Female	high transmission	6-Mar-10	active follow	9	36	No
3	30-Jan-10	Female	high transmission	7-Apr-10	active follow	9	35.8	No

4	30-Jan-10	Female	high transmission	6-May-10	active follow	9	36.1	No
5	30-Jan-10	Female	high transmission	28-May-10	passive follow	9	36.2	Yes
6	30-Jan-10	Female	high transmission	7-Jun-10	active follow	2638	36.2	No
7	30-Jan-10	Female	high transmission	12-Jun-10	passive follow	152557		
8	30-Jan-10	Female	high transmission	7-Jul-10	active follow	9	36	No
9	30-Jan-10	Female	high transmission	20-Jul-10	passive follow	9	36.8	Yes
10	30-Jan-10	Female	high transmission	12-Aug-10	active follow	943	36	No
11	30-Jan-10	Female	high transmission	5-Sep-10	passive follow	5673	36.7	Yes
12	30-Jan-10	Female	high transmission	14-Sep-10	active follow	9	36.5	No
13	30-Jan-10	Female	high transmission	14-Oct-10	active follow	9	36.1	No
14	30-Jan-10	Female	high transmission	16-Oct-10	passive follow	9	36.2	Yes
15	30-Jan-10	Female	high transmission	27-Oct-10	passive follow		37.2	Yes
16	30-Jan-10	Female	high transmission	29-Oct-10	passive follow	9		
17	30-Jan-10	Female	high transmission	10-Nov-10	active follow	9	35.8	No
18	30-Jan-10	Female	high transmission	10-Dec-10	active follow	9	37	Yes
19	30-Jan-10	Female	high transmission	19-Dec-10	passive follow	440782	38	Yes
20	30-Jan-10	Female	high transmission	13-Jan-11	active follow	9	35.3	Yes
1	28-Jan-10	Female	high transmission	23-Feb-10	active follow	9	36.8	No
2	28-Jan-10	Female	high transmission	25-Mar-10	active follow	9	36.3	No
3	28-Jan-10	Female	high transmission	22-Apr-10	active follow	9	36.5	No
4	28-Jan-10	Female	high transmission	28-May-10	active follow		36.6	No
5	28-Jan-10	Female	high transmission	30-Jun-10	active follow	4749	36.8	No
6	28-Jan-10	Female	high transmission	27-Jul-10	active follow	9	37.3	No
7	28-Jan-10	Female	high transmission	26-Aug-10	active follow		38.6	Yes
8	28-Jan-10	Female	high transmission	29-Sep-10	active follow	9	36.3	No
9	28-Jan-10	Female	high transmission	9-Nov-10	active follow		36.9	No
10	28-Jan-10	Female	high transmission	11-Dec-10	active follow		36.3	No
11	28-Jan-10	Female	high transmission	16-Dec-10	passive follow	200	37.1	Yes
12	28-Jan-10	Female	high transmission	12-Jan-11	active follow	9	36.4	No
1	11-Feb-10	Female	low transmission	16-Mar-10	active follow	9	36.5	No
2	11-Feb-10	Female	low transmission	19-Apr-10	active follow	9	36.6	No
3	11-Feb-10	Female	low transmission	20-May-10	active follow		36.8	No
4	11-Feb-10	Female	low transmission	21-Jun-10	active follow	9	36.3	No
5	11-Feb-10	Female	low transmission	28-Jul-10	active follow	9	36.2	No
6	11-Feb-10	Female	low transmission	8-Sep-10	active follow	9	36.1	No
7	11-Feb-10	Female	low transmission	11-Oct-10	passive follow	9	36.2	Yes
8	11-Feb-10	Female	low transmission	15-Nov-10	active follow	24528	38.7	Yes
9	11-Feb-10	Female	low transmission	16-Dec-10	active follow	39824	37.2	No
10	11-Feb-10	Female	low transmission	21-Jan-11	active follow	9	36.5	No
1	11-Feb-10	Female	low transmission	19-Mar-10	active follow	9	36.5	No
2	11-Feb-10	Female	low transmission	28-Apr-10	active follow	9	36.5	No
3	11-Feb-10	Female	low transmission	28-May-10	active follow	9	36.3	No
4	11-Feb-10	Female	low transmission	8-Jul-10	active follow	9	36.2	No
5	11-Feb-10	Female	low transmission	9-Aug-10	active follow	27223	37.5	Yes
6	11-Feb-10	Female	low transmission	10-Sep-10	active follow	9	36.6	No
7	11-Feb-10	Female	low transmission	18-Oct-10	active follow	2142	36.5	No
8	11-Feb-10	Female	low transmission	19-Nov-10	active follow	319	37	No

9	11-Feb-10	Female	low transmission	10-Dec-10	passive follow	139		
10	11-Feb-10	Female	low transmission	11-Dec-10	passive follow		37.4	Yes
11	11-Feb-10	Female	low transmission	31-Dec-10	active follow	9	36.8	No
12	11-Feb-10	Female	low transmission	3-Feb-11	active follow	9	36.6	No
1	10-Feb-10	Female	low transmission	15-Mar-10	active follow	9	36.8	No
2	10-Feb-10	Female	low transmission	19-Mar-10	passive follow		36.6	Yes
3	10-Feb-10	Female	low transmission	17-Apr-10	active follow		36.7	No
4	10-Feb-10	Female	low transmission	27-May-10	active follow	9	36	No
5	10-Feb-10	Female	low transmission	31-May-10	passive follow	9	36.3	No
6	10-Feb-10	Female	low transmission	7-Jul-10	active follow	9	36.6	No
7	10-Feb-10	Female	low transmission	29-Jul-10	passive follow	4135	36.5	No
8	10-Feb-10	Female	low transmission	9-Aug-10	active follow	9	36.4	No
9	10-Feb-10	Female	low transmission	17-Sep-10	active follow	2191	36.3	No
10	10-Feb-10	Female	low transmission	1-Nov-10	active follow	9	36	No
11	10-Feb-10	Female	low transmission	13-Dec-10	active follow	320	36.7	No
12	10-Feb-10	Female	low transmission	3-Jan-11	passive follow	9	36.3	No
13	10-Feb-10	Female	low transmission	17-Jan-11	active follow		36.5	No
1	15-Feb-10	Female	low transmission	17-Mar-10	active follow	9	36.8	No
2	15-Feb-10	Female	low transmission	30-Mar-10	passive follow	9	36.9	Yes
3	15-Feb-10	Female	low transmission	16-Apr-10	active follow		35.8	No
4	15-Feb-10	Female	low transmission	27-May-10	active follow	9	36.6	No
5	15-Feb-10	Female	low transmission	8-Jun-10	passive follow	3798	36.8	No
6	15-Feb-10	Female	low transmission	25-Jun-10	active follow	1337	36.3	No
7	15-Feb-10	Female	low transmission	27-Jul-10	active follow	3190	36.1	No
8	15-Feb-10	Female	low transmission	26-Aug-10	active follow	18081	36.6	No
9	15-Feb-10	Female	low transmission	21-Sep-10	active follow	4543	36	No
10	15-Feb-10	Female	low transmission	24-Sep-10	passive follow	4983	36.8	Yes
11	15-Feb-10	Female	low transmission	28-Oct-10	active follow			
12	15-Feb-10	Female	low transmission	27-Nov-10	active follow			
13	15-Feb-10	Female	low transmission	28-Dec-10	active follow	621	36.3	No
14	15-Feb-10	Female	low transmission	1-Feb-11	active follow	1421	36	No
1	9-Feb-10	Female	low transmission	13-Mar-10	active follow	9	36.7	No
2	9-Feb-10	Female	low transmission	26-Apr-10	active follow	9	36.3	No
3	9-Feb-10	Female	low transmission	24-May-10	active follow		35.8	No
4	9-Feb-10	Female	low transmission	24-Jun-10	active follow	598	35.2	No
5	9-Feb-10	Female	low transmission	20-Jul-10	active follow	9	36.2	No
6	9-Feb-10	Female	low transmission	18-Aug-10	active follow	2394	35.5	No
7	9-Feb-10	Female	low transmission	23-Aug-10	passive follow	186	35	Yes
8	9-Feb-10	Female	low transmission	29-Sep-10	active follow		36.3	No
9	9-Feb-10	Female	low transmission	12-Nov-10	active follow	9	36	Yes
10	9-Feb-10	Female	low transmission	13-Dec-10	active follow	9	35.7	No
11	9-Feb-10	Female	low transmission	19-Jan-11	active follow		36.9	No
12	9-Feb-10	Female	low transmission	28-Jan-11	passive follow		36.6	Yes
1	14-Feb-10	Female	low transmission	23-Mar-10	active follow	9	36.3	No
2	14-Feb-10	Female	low transmission	29-Apr-10	active follow	9	36	No
3	14-Feb-10	Female	low transmission	25-May-10	active follow	9	36.3	No
4	14-Feb-10	Female	low transmission	24-Jun-10	active follow	9	36.1	No

5	14-Feb-10	Female	low transmission	20-Jul-10	active follow	9	35.4	No
6	14-Feb-10	Female	low transmission	19-Aug-10	active follow	1857	36.8	No
7	14-Feb-10	Female	low transmission	17-Sep-10	active follow	3233	37.2	No
8	14-Feb-10	Female	low transmission	24-Sep-10	passive follow		37	Yes
9	14-Feb-10	Female	low transmission	22-Oct-10	active follow	2397	36.9	No
10	14-Feb-10	Female	low transmission	24-Nov-10	active follow	15108	36.3	No
11	14-Feb-10	Female	low transmission	25-Dec-10	active follow	9	36.3	No
12	14-Feb-10	Female	low transmission	17-Jan-11	passive follow	68354	38.6	Yes
13	14-Feb-10	Female	low transmission	29-Jan-11	active follow	9	36.5	No
1	1-Dec-08	Female	low transmission	31-Dec-08	active follow	9	36.8	No
2	1-Dec-08	Female	low transmission	14-Jan-09	passive follow		37.3	No
3	1-Dec-08	Female	low transmission	6-Feb-09	active follow	9	36.8	No
4	1-Dec-08	Female	low transmission	17-Mar-09	active follow	9	36.1	No
5	1-Dec-08	Female	low transmission	22-Apr-09	active follow	51829	37.1	No
6	1-Dec-08	Female	low transmission	23-May-09	active follow	32074	36.9	No
7	1-Dec-08	Female	low transmission	25-May-09	passive follow		37.8	Yes
8	1-Dec-08	Female	low transmission	1-Jul-09	active follow	9	36.3	No
9	1-Dec-08	Female	low transmission	25-Jul-09	active follow	55309	39.1	Yes
10	1-Dec-08	Female	low transmission	19-Aug-09	active follow	9	36.2	No
11	1-Dec-08	Female	low transmission	21-Sep-09	active follow	9	36.3	No
12	1-Dec-08	Female	low transmission	17-Oct-09	passive follow	9	36.2	Yes
13	1-Dec-08	Female	low transmission	28-Oct-09	active follow		36.2	Yes
14	1-Dec-08	Female	low transmission	16-Nov-09	passive follow	9		
15	1-Dec-08	Female	low transmission	26-Nov-09	active follow	9	36.2	No
1	27-Feb-10	Female	low transmission	13-Mar-10	passive follow	9	36.9	No
2	27-Feb-10	Female	low transmission	7-Apr-10	active follow	9	37.4	Yes
3	27-Feb-10	Female	low transmission	8-May-10	active follow	9	36.9	No
4	27-Feb-10	Female	low transmission	8-Jun-10	active follow	9	36.1	No
5	27-Feb-10	Female	low transmission	6-Jul-10	active follow	9	35	No
6	27-Feb-10	Female	low transmission	12-Aug-10	active follow			
7	27-Feb-10	Female	low transmission	1-Sep-10	passive follow	20636	35.7	Yes
8	27-Feb-10	Female	low transmission	13-Sep-10	active follow	188	36	No
9	27-Feb-10	Female	low transmission	19-Sep-10	passive follow	14870	35.7	Yes
10	27-Feb-10	Female	low transmission	12-Oct-10	active follow	1767	35.7	No
11	27-Feb-10	Female	low transmission	21-Oct-10	passive follow	22406	35.7	Yes
12	27-Feb-10	Female	low transmission	9-Nov-10	active follow	9	35.7	No
13	27-Feb-10	Female	low transmission	10-Nov-10	passive follow	9	35.7	Yes
14	27-Feb-10	Female	low transmission	7-Dec-10	active follow	9	36.2	No
15	27-Feb-10	Female	low transmission	3-Jan-11	active follow	9	37	Yes
16	27-Feb-10	Female	low transmission	1-Feb-11	active follow	9	37	Yes
1	22-Feb-10	Male	low transmission	9-Mar-10	passive follow		36.9	No
2	22-Feb-10	Male	low transmission	23-Mar-10	active follow	9	36.8	No
3	22-Feb-10	Male	low transmission	26-Apr-10	active follow	9	36.5	No
4	22-Feb-10	Male	low transmission	5-May-10	passive follow	9	36.4	Yes
5	22-Feb-10	Male	low transmission	24-May-10	active follow		36.4	No
6	22-Feb-10	Male	low transmission	24-Jun-10	active follow	9	36	No
7	22-Feb-10	Male	low transmission	19-Jul-10	active follow	195	36.3	No

8	22-Feb-10	Male	low transmission	29-Jul-10	passive follow	9	36.3	Yes
9	22-Feb-10	Male	low transmission	28-Aug-10	active follow		37.5	Yes
10	22-Feb-10	Male	low transmission	6-Oct-10	active follow		36.3	No
11	22-Feb-10	Male	low transmission	30-Oct-10	passive follow	57845	37.9	Yes
12	22-Feb-10	Male	low transmission	6-Dec-10	active follow	9	36.7	No
13	22-Feb-10	Male	low transmission	13-Dec-10	passive follow	9	37	No
14	22-Feb-10	Male	low transmission	12-Jan-11	active follow			
15	22-Feb-10	Male	low transmission	29-Jan-11	passive follow	9	36.8	No
16	22-Feb-10	Male	low transmission	15-Feb-11	active follow	9	36.6	No
1	20-Feb-10	Male	low transmission	26-Mar-10	active follow	9	36.8	No
2	20-Feb-10	Male	low transmission	28-Apr-10	active follow	9	36.8	No
3	20-Feb-10	Male	low transmission	3-Jun-10	active follow	9	36.6	No
4	20-Feb-10	Male	low transmission	7-Jul-10	active follow		37.6	Yes
5	20-Feb-10	Male	low transmission	9-Jul-10	active follow	9		
6	20-Feb-10	Male	low transmission	19-Jul-10	passive follow		37.6	Yes
7	20-Feb-10	Male	low transmission	6-Aug-10	active follow	9	36.3	Yes
8	20-Feb-10	Male	low transmission	9-Aug-10	passive follow	9	36.5	Yes
9	20-Feb-10	Male	low transmission	13-Sep-10	active follow	9	36.8	No
10	20-Feb-10	Male	low transmission	7-Oct-10	passive follow	217634	39.9	Yes
11	20-Feb-10	Male	low transmission	21-Oct-10	active follow	9	36.7	No
12	20-Feb-10	Male	low transmission	2-Nov-10	passive follow	9		
13	20-Feb-10	Male	low transmission	19-Nov-10	active follow	256	36.7	No
14	20-Feb-10	Male	low transmission	23-Dec-10	active follow		36.5	No
15	20-Feb-10	Male	low transmission	21-Jan-11	active follow	2081	37.1	No
16	20-Feb-10	Male	low transmission	16-Feb-11	active follow	9	36.3	No
1	28-Feb-10	Male	low transmission	5-Apr-10	active follow	9	36.8	No
2	28-Feb-10	Male	low transmission	5-May-10	active follow	9	36.3	No
3	28-Feb-10	Male	low transmission	4-Jun-10	active follow	1148	36.1	No
4	28-Feb-10	Male	low transmission	28-Jun-10	passive follow	34318	37.6	Yes
5	28-Feb-10	Male	low transmission	9-Jul-10	active follow	9	36.4	No
6	28-Feb-10	Male	low transmission	13-Aug-10	active follow	9	36.5	No
7	28-Feb-10	Male	low transmission	16-Sep-10	active follow	9	36.7	No
8	28-Feb-10	Male	low transmission	23-Oct-10	active follow			
9	28-Feb-10	Male	low transmission	29-Nov-10	active follow			
10	28-Feb-10	Male	low transmission	6-Jan-11	active follow	9	36.3	No
11	28-Feb-10	Male	low transmission	13-Feb-11	active follow	9	36.9	No
1	5-Mar-10	Female	low transmission	7-Apr-10	active follow	9	35.8	No
2	5-Mar-10	Female	low transmission	6-May-10	active follow		36.1	No
3	5-Mar-10	Female	low transmission	17-May-10	passive follow	9	37.6	Yes
4	5-Mar-10	Female	low transmission	5-Jun-10	active follow	9	36.1	No
5	5-Mar-10	Female	low transmission	24-Jun-10	passive follow	9	36.4	No
6	5-Mar-10	Female	low transmission	6-Jul-10	active follow	9	35.9	No
7	5-Mar-10	Female	low transmission	6-Aug-10	active follow	9	36.1	No
8	5-Mar-10	Female	low transmission	11-Aug-10	passive follow	9	36.4	No
9	5-Mar-10	Female	low transmission	14-Sep-10	active follow	9	36.1	No
10	5-Mar-10	Female	low transmission	22-Sep-10	passive follow	9	37.2	Yes
11	5-Mar-10	Female	low transmission	14-Oct-10	active follow	120	36	No

12	5-Mar-10	Female	low transmission	27-Oct-10	passive follow	28123	36.2	Yes
13	5-Mar-10	Female	low transmission	12-Nov-10	active follow		37.1	Yes
14	5-Mar-10	Female	low transmission	8-Dec-10	passive follow	9	38.9	Yes
15	5-Mar-10	Female	low transmission	14-Dec-10	active follow	9	36.2	No
16	5-Mar-10	Female	low transmission	5-Jan-11	passive follow	134540	39.7	Yes
17	5-Mar-10	Female	low transmission	18-Jan-11	active follow	9	36.1	No
18	5-Mar-10	Female	low transmission	15-Feb-11	active follow	9	36.4	No
1	7-Dec-08	Male	low transmission	8-Jan-09	active follow	9	36.7	No
2	7-Dec-08	Male	low transmission	6-Feb-09	passive follow		36.5	Yes
3	7-Dec-08	Male	low transmission	8-Feb-09	active follow	9	36.7	No
4	7-Dec-08	Male	low transmission	12-Mar-09	active follow	9	36.5	No
5	7-Dec-08	Male	low transmission	14-Apr-09	active follow	9	36.7	No
6	7-Dec-08	Male	low transmission	20-May-09	active follow	9	36.5	No
7	7-Dec-08	Male	low transmission	22-Jun-09	active follow	9	36.5	No
8	7-Dec-08	Male	low transmission	29-Jun-09	passive follow	25487	40.3	Yes
9	7-Dec-08	Male	low transmission	22-Jul-09	active follow	5167	36.4	No
10	7-Dec-08	Male	low transmission	15-Aug-09	passive follow	60793	37.8	Yes
11	7-Dec-08	Male	low transmission	20-Aug-09	active follow		36.5	No
12	7-Dec-08	Male	low transmission	24-Sep-09	active follow	9	36.2	No
13	7-Dec-08	Male	low transmission	21-Oct-09	active follow	9	37.2	Yes
14	7-Dec-08	Male	low transmission	16-Nov-09	active follow	9	36.3	No
1	5-Mar-10	Female	low transmission	10-Apr-10	active follow	9	37.2	No
2	5-Mar-10	Female	low transmission	13-May-10	active follow	9	37	No
3	5-Mar-10	Female	low transmission	16-Jun-10	active follow	21227	36.9	No
4	5-Mar-10	Female	low transmission	16-Jul-10	active follow	9	35.8	No
5	5-Mar-10	Female	low transmission	24-Aug-10	active follow	3019	36.5	No
6	5-Mar-10	Female	low transmission	18-Sep-10	passive follow	182514		
7	5-Mar-10	Female	low transmission	19-Sep-10	passive follow		37.7	Yes
8	5-Mar-10	Female	low transmission	27-Sep-10	active follow	9	36.7	No
9	5-Mar-10	Female	low transmission	26-Oct-10	active follow	9	37.1	No
10	5-Mar-10	Female	low transmission	2-Dec-10	active follow	9	37.3	No
11	5-Mar-10	Female	low transmission	6-Jan-11	active follow	9	37.1	No
12	5-Mar-10	Female	low transmission	12-Feb-11	active follow			
1	3-Mar-10	Male	low transmission	6-Apr-10	active follow	9	36.6	No
2	3-Mar-10	Male	low transmission	4-May-10	active follow	9	36.5	No
3	3-Mar-10	Male	low transmission	2-Jun-10	active follow	13641	35.8	No
4	3-Mar-10	Male	low transmission	5-Jul-10	active follow		36.2	No
5	3-Mar-10	Male	low transmission	5-Aug-10	active follow	9	36.6	No
6	3-Mar-10	Male	low transmission	3-Sep-10	active follow	22913	36.8	Yes
7	3-Mar-10	Male	low transmission	8-Oct-10	active follow	3694	36.3	No
8	3-Mar-10	Male	low transmission	18-Oct-10	passive follow	5037	36.4	Yes
9	3-Mar-10	Male	low transmission	11-Nov-10	active follow	9	36.4	No
10	3-Mar-10	Male	low transmission	19-Nov-10	passive follow	9	36.5	No
11	3-Mar-10	Male	low transmission	8-Dec-10	active follow	9	36	No
12	3-Mar-10	Male	low transmission	5-Jan-11	active follow	9	36.1	No
13	3-Mar-10	Male	low transmission	1-Feb-11	passive follow	9	36	Yes
14	3-Mar-10	Male	low transmission	10-Feb-11	active follow			

1	3-Mar-10	Male	low transmission	5-Apr-10	active follow	9	36.7	No
2	3-Mar-10	Male	low transmission	4-May-10	active follow	9	36.5	No
3	3-Mar-10	Male	low transmission	5-Jun-10	active follow	9	36.2	No
4	3-Mar-10	Male	low transmission	9-Jul-10	active follow	60	36.5	No
5	3-Mar-10	Male	low transmission	14-Aug-10	active follow	2908	36.8	No
6	3-Mar-10	Male	low transmission	24-Aug-10	passive follow	9	36.5	Yes
7	3-Mar-10	Male	low transmission	16-Sep-10	active follow	9	36.5	No
8	3-Mar-10	Male	low transmission	14-Oct-10	active follow		38.4	Yes
9	3-Mar-10	Male	low transmission	12-Nov-10	active follow	9	35.7	No
10	3-Mar-10	Male	low transmission	15-Dec-10	passive follow	56810	38.8	Yes
11	3-Mar-10	Male	low transmission	15-Jan-11	active follow	9	36	No
12	3-Mar-10	Male	low transmission	9-Feb-11	passive follow	9	38.8	Yes
13	3-Mar-10	Male	low transmission	21-Feb-11	active follow	9	36	No
1	16-Mar-10	Male	low transmission	20-Apr-10	active follow	9	36.7	No
2	16-Mar-10	Male	low transmission	28-May-10	active follow	158	36.5	No
3	16-Mar-10	Male	low transmission	8-Jul-10	active follow	9	36.6	No
4	16-Mar-10	Male	low transmission	23-Jul-10	passive follow	83876	37.9	Yes
5	16-Mar-10	Male	low transmission	9-Aug-10	active follow	9	36.6	No
6	16-Mar-10	Male	low transmission	10-Sep-10	active follow	9	35.6	No
7	16-Mar-10	Male	low transmission	22-Sep-10	passive follow	77231	36.8	Yes
8	16-Mar-10	Male	low transmission	18-Oct-10	active follow	9	36.6	No
9	16-Mar-10	Male	low transmission	5-Nov-10	passive follow	560238	36.5	Yes
10	16-Mar-10	Male	low transmission	19-Nov-10	active follow	9	36.3	No
11	16-Mar-10	Male	low transmission	26-Dec-10	active follow	9	36.4	No
12	16-Mar-10	Male	low transmission	27-Jan-11	active follow	5080	36	No
13	16-Mar-10	Male	low transmission	23-Feb-11	active follow	417	36	No
14	16-Mar-10	Male	low transmission	6-Mar-11	passive follow	440187		
1	14-Mar-10	Female	low transmission	16-Apr-10	active follow	9	36.6	No
2	14-Mar-10	Female	low transmission	24-May-10	active follow	9	36	No
3	14-Mar-10	Female	low transmission	12-Jun-10	passive follow		36.8	No
4	14-Mar-10	Female	low transmission	29-Jun-10	active follow		36.5	No
5	14-Mar-10	Female	low transmission	27-Jul-10	active follow		36.8	No
6	14-Mar-10	Female	low transmission	28-Aug-10	active follow	8903	36.5	No
7	14-Mar-10	Female	low transmission	24-Sep-10	active follow	15046	37.3	Yes
8	14-Mar-10	Female	low transmission	11-Oct-10	passive follow	150977	37.6	Yes
9	14-Mar-10	Female	low transmission	31-Oct-10	active follow	9	36.6	No
10	14-Mar-10	Female	low transmission	1-Dec-10	active follow	33133	39.1	Yes
11	14-Mar-10	Female	low transmission	29-Dec-10	active follow	3465	37.3	Yes
12	14-Mar-10	Female	low transmission	26-Jan-11	active follow	9	36.4	No
13	14-Mar-10	Female	low transmission	1-Feb-11	passive follow	9	36.2	No
14	14-Mar-10	Female	low transmission	21-Feb-11	active follow	9	36.5	No
1	5-Dec-08	Male	low transmission	21-Jan-09	active follow	9	36.6	No
2	5-Dec-08	Male	low transmission	27-Feb-09	active follow			
3	5-Dec-08	Male	low transmission	30-Mar-09	active follow	20595	36.5	No
4	5-Dec-08	Male	low transmission	27-Apr-09	active follow	644	36	No
5	5-Dec-08	Male	low transmission	12-May-09	passive follow		37.3	Yes
6	5-Dec-08	Male	low transmission	1-Jun-09	active follow	9	36.8	No

7	5-Dec-08	Male	low transmission	7-Jul-09	active follow	9	36.3	No
8	5-Dec-08	Male	low transmission	8-Aug-09	passive follow	20869	38.7	Yes
9	5-Dec-08	Male	low transmission	10-Aug-09	active follow	9	36	No
10	5-Dec-08	Male	low transmission	10-Sep-09	active follow	9	36.5	No
11	5-Dec-08	Male	low transmission	17-Oct-09	active follow			
12	5-Dec-08	Male	low transmission	30-Oct-09	passive follow	9	38.4	Yes
13	5-Dec-08	Male	low transmission	23-Nov-09	active follow			
1	18-Mar-10	Female	low transmission	16-Apr-10	active follow	9	36.5	No
2	18-Mar-10	Female	low transmission	28-May-10	active follow	9	36.6	No
3	18-Mar-10	Female	low transmission	9-Jul-10	active follow	9	35.9	No
4	18-Mar-10	Female	low transmission	27-Jul-10	passive follow	9	36.6	No
5	18-Mar-10	Female	low transmission	11-Aug-10	active follow	9	36.6	No
6	18-Mar-10	Female	low transmission	7-Sep-10	active follow	9	36.8	No
7	18-Mar-10	Female	low transmission	7-Oct-10	active follow	9	36.5	Yes
8	18-Mar-10	Female	low transmission	25-Oct-10	passive follow	142747	39.4	Yes
9	18-Mar-10	Female	low transmission	9-Nov-10	active follow	9	36.5	No
10	18-Mar-10	Female	low transmission	7-Dec-10	active follow	776	36.2	No
11	18-Mar-10	Female	low transmission	4-Jan-11	active follow	9	36.5	Yes
12	18-Mar-10	Female	low transmission	5-Feb-11	active follow	9	36	No
13	18-Mar-10	Female	low transmission	15-Feb-11	passive follow	9	36.3	Yes
14	18-Mar-10	Female	low transmission	10-Mar-11	active follow	9	36.9	No
1	17-Mar-10	Female	low transmission	15-Apr-10	active follow	9	36	No
2	17-Mar-10	Female	low transmission	16-May-10	active follow	9	36.6	No
3	17-Mar-10	Female	low transmission	8-Jun-10	passive follow	9	37.8	Yes
4	17-Mar-10	Female	low transmission	24-Jun-10	active follow	9	36.5	No
5	17-Mar-10	Female	low transmission	3-Aug-10	active follow	9	36.3	No
6	17-Mar-10	Female	low transmission	27-Aug-10	passive follow	9	36.5	No
7	17-Mar-10	Female	low transmission	7-Sep-10	active follow	2343	36.7	No
8	17-Mar-10	Female	low transmission	18-Oct-10	active follow	9	36.6	No
9	17-Mar-10	Female	low transmission	17-Nov-10	active follow	123320	38.2	Yes
10	17-Mar-10	Female	low transmission	19-Dec-10	active follow	9	36.5	No
11	17-Mar-10	Female	low transmission	23-Jan-11	active follow	9	37.2	No
12	17-Mar-10	Female	low transmission	22-Feb-11	active follow	9	36.5	No
1	25-Mar-10	Male	low transmission	27-Apr-10	active follow	9	36.5	No
2	25-Mar-10	Male	low transmission	25-May-10	active follow	9	36.5	No
3	25-Mar-10	Male	low transmission	24-Jun-10	active follow	9	35.4	No
4	25-Mar-10	Male	low transmission	20-Jul-10	active follow	9	36.7	No
5	25-Mar-10	Male	low transmission	6-Aug-10	passive follow	21333	37.5	Yes
6	25-Mar-10	Male	low transmission	26-Aug-10	active follow	9	36.3	No
7	25-Mar-10	Male	low transmission	27-Sep-10	active follow	1260	36.4	No
8	25-Mar-10	Male	low transmission	27-Oct-10	active follow	1554	37.3	No
9	25-Mar-10	Male	low transmission	2-Nov-10	passive follow	9	37.2	Yes
10	25-Mar-10	Male	low transmission	26-Nov-10	active follow	9	35.5	No
11	25-Mar-10	Male	low transmission	29-Dec-10	active follow	9	36.3	No
12	25-Mar-10	Male	low transmission	31-Jan-11	active follow	9	36.7	No
13	25-Mar-10	Male	low transmission	14-Feb-11	passive follow	9	36.8	Yes
14	25-Mar-10	Male	low transmission	4-Mar-11	active follow	9	37	No

1	18-Mar-10	Female	low transmission	20-Apr-10	active follow	9	36.6	No
2	18-Mar-10	Female	low transmission	4-May-10	passive follow	29	36.8	No
3	18-Mar-10	Female	low transmission	26-May-10	active follow	9	36.5	No
4	18-Mar-10	Female	low transmission	29-Jun-10	passive follow	43412	36	No
5	18-Mar-10	Female	low transmission	28-Jul-10	active follow	22568	39.3	Yes
6	18-Mar-10	Female	low transmission	3-Sep-10	active follow			
7	18-Mar-10	Female	low transmission	7-Oct-10	active follow	443266	38.9	Yes
8	18-Mar-10	Female	low transmission	27-Oct-10	passive follow	9	37.1	Yes
9	18-Mar-10	Female	low transmission	15-Nov-10	active follow		37	No
10	18-Mar-10	Female	low transmission	23-Dec-10	active follow	9	36.6	No
11	18-Mar-10	Female	low transmission	29-Jan-11	active follow			
12	18-Mar-10	Female	low transmission	5-Mar-11	active follow	16669	36.7	No
1	23-Mar-10	Male	low transmission	28-Apr-10	active follow	9	36.5	No
2	23-Mar-10	Male	low transmission	27-May-10	active follow	9	36.3	No
3	23-Mar-10	Male	low transmission	2-Jul-10	active follow	4758	36.5	No
4	23-Mar-10	Male	low transmission	14-Jul-10	passive follow	4944	38	Yes
5	23-Mar-10	Male	low transmission	6-Aug-10	active follow	6716	36.1	No
6	23-Mar-10	Male	low transmission	9-Sep-10	active follow	9	36.5	No
7	23-Mar-10	Male	low transmission	22-Sep-10	passive follow	9	36.9	No
8	23-Mar-10	Male	low transmission	8-Oct-10	active follow	9	36.5	No
9	23-Mar-10	Male	low transmission	12-Nov-10	active follow	11304	36.1	No
10	23-Mar-10	Male	low transmission	16-Dec-10	active follow	9	36.8	No
11	23-Mar-10	Male	low transmission	20-Jan-11	active follow		37	No
12	23-Mar-10	Male	low transmission	18-Feb-11	active follow	9	36.2	No
13	23-Mar-10	Male	low transmission	14-Mar-11	active follow	9	36.7	No
1	23-Mar-10	Female	low transmission	25-Apr-10	active follow	30	36.8	No
2	23-Mar-10	Female	low transmission	29-Apr-10	passive follow	9	36.9	Yes
3	23-Mar-10	Female	low transmission	2-Jun-10	active follow	9	35.3	No
4	23-Mar-10	Female	low transmission	1-Jul-10	active follow	9	36.6	No
5	23-Mar-10	Female	low transmission	10-Jul-10	active follow	9		
6	23-Mar-10	Female	low transmission	19-Jul-10	passive follow		36	No
7	23-Mar-10	Female	low transmission	4-Aug-10	active follow	9	36.2	No
8	23-Mar-10	Female	low transmission	18-Aug-10	passive follow	9	37.1	No
9	23-Mar-10	Female	low transmission	3-Sep-10	active follow	9	36.6	No
10	23-Mar-10	Female	low transmission	5-Oct-10	active follow	9	37.2	No
11	23-Mar-10	Female	low transmission	7-Oct-10	passive follow	9	36	Yes
12	23-Mar-10	Female	low transmission	4-Nov-10	passive follow	35334	36.8	Yes
13	23-Mar-10	Female	low transmission	16-Nov-10	active follow	9	36.5	No
14	23-Mar-10	Female	low transmission	23-Dec-10	active follow			
15	23-Mar-10	Female	low transmission	29-Dec-10	passive follow	9	36.9	Yes
16	23-Mar-10	Female	low transmission	22-Jan-11	active follow			
17	23-Mar-10	Female	low transmission	19-Feb-11	active follow	9	36.8	No
18	23-Mar-10	Female	low transmission	21-Feb-11	passive follow	9	38.3	Yes
19	23-Mar-10	Female	low transmission	19-Mar-11	active follow	9	36.5	No
1	2-Apr-10	Male	high transmission	3-May-10	active follow	9	36.5	No
2	2-Apr-10	Male	high transmission	4-Jun-10	active follow	9	36.5	No
3	2-Apr-10	Male	high transmission	8-Jul-10	active follow	9	36.6	No

4	2-Apr-10	Male	high transmission	4-Aug-10	active follow	9	36.4	No
5	2-Apr-10	Male	high transmission	8-Sep-10	active follow	9	35.8	No
6	2-Apr-10	Male	high transmission	12-Oct-10	active follow	9	36.7	No
7	2-Apr-10	Male	high transmission	10-Nov-10	active follow	9	37.2	No
8	2-Apr-10	Male	high transmission	10-Dec-10	active follow	12491	36.8	No
9	2-Apr-10	Male	high transmission	16-Jan-11	active follow		37.1	No
10	2-Apr-10	Male	high transmission	18-Feb-11	active follow	10023	37.5	No
11	2-Apr-10	Male	high transmission	18-Mar-11	active follow		36	No
12	2-Apr-10	Male	high transmission	21-Mar-11	passive follow	9	39.3	Yes
1	27-Mar-10	Male	low transmission	30-Apr-10	active follow	9	36.5	No
2	27-Mar-10	Male	low transmission	30-Apr-10	active follow	9	36.5	No
3	27-Mar-10	Male	low transmission	3-Jun-10	active follow	9	36.5	No
4	27-Mar-10	Male	low transmission	3-Jun-10	active follow	3717	36.5	No
5	27-Mar-10	Male	low transmission	30-Jun-10	active follow		35.5	No
6	27-Mar-10	Male	low transmission	30-Jun-10	active follow	9	36.5	No
7	27-Mar-10	Male	low transmission	28-Jul-10	active follow	9	36.3	Yes
8	27-Mar-10	Male	low transmission	28-Jul-10	active follow	9	36.6	Yes
9	27-Mar-10	Male	low transmission	26-Aug-10	active follow	9	36.3	No
10	27-Mar-10	Male	low transmission	26-Aug-10	active follow	9	36.5	No
11	27-Mar-10	Male	low transmission	21-Sep-10	active follow	9	36.5	Yes
12	27-Mar-10	Male	low transmission	21-Sep-10	active follow	385101	36.9	Yes
13	27-Mar-10	Male	low transmission	28-Oct-10	active follow			
14	27-Mar-10	Male	low transmission	28-Oct-10	active follow			
15	27-Mar-10	Male	low transmission	9-Nov-10	passive follow	9	37.1	Yes
16	27-Mar-10	Male	low transmission	9-Nov-10	passive follow	9	37.2	Yes
17	27-Mar-10	Male	low transmission	27-Nov-10	active follow			
18	27-Mar-10	Male	low transmission	27-Nov-10	active follow			
19	27-Mar-10	Male	low transmission	30-Dec-10	active follow	9	35.4	No
20	27-Mar-10	Male	low transmission	30-Dec-10	active follow	4616	36.3	No
21	27-Mar-10	Male	low transmission	11-Jan-11	passive follow	78065	37.6	Yes
22	27-Mar-10	Male	low transmission	11-Jan-11	passive follow	9	36.5	Yes
23	27-Mar-10	Male	low transmission	30-Jan-11	active follow	9	35.8	No
24	27-Mar-10	Male	low transmission	30-Jan-11	active follow	2476	36.7	No
25	27-Mar-10	Male	low transmission	3-Mar-11	active follow	9	36	No
26	27-Mar-10	Male	low transmission	3-Mar-11	active follow	9	36.1	No
1	3-Apr-10	Male	high transmission	6-May-10	active follow	9	36.3	No
2	3-Apr-10	Male	high transmission	3-Jun-10	active follow	9	36.5	No
3	3-Apr-10	Male	high transmission	10-Jul-10	active follow	9	36	No
4	3-Apr-10	Male	high transmission	15-Aug-10	active follow	3370	36.2	No
5	3-Apr-10	Male	high transmission	10-Sep-10	active follow		36.2	No
6	3-Apr-10	Male	high transmission	14-Oct-10	active follow		36.8	No
7	3-Apr-10	Male	high transmission	16-Oct-10	passive follow	6878	38.4	Yes
8	3-Apr-10	Male	high transmission	10-Nov-10	active follow	9	35.4	No
9	3-Apr-10	Male	high transmission	9-Dec-10	active follow	9	36	No
10	3-Apr-10	Male	high transmission	23-Dec-10	passive follow	9	35.8	No
11	3-Apr-10	Male	high transmission	7-Jan-11	active follow	9	35.8	No
12	3-Apr-10	Male	high transmission	9-Feb-11	active follow	9	36.2	No

13	3-Apr-10	Male	high transmission	3-Mar-11	active follow	9	36.1	No
14	3-Apr-10	Male	high transmission	29-Mar-11	active follow	9	36	No
1	28-Mar-10	Female	low transmission	30-Apr-10	active follow	9	36.1	No
2	28-Mar-10	Female	low transmission	2-Jun-10	active follow	9	36.3	No
3	28-Mar-10	Female	low transmission	6-Jul-10	active follow	9	36.2	No
4	28-Mar-10	Female	low transmission	9-Aug-10	active follow	712	36	No
5	28-Mar-10	Female	low transmission	9-Sep-10	active follow	9	36.4	No
6	28-Mar-10	Female	low transmission	8-Oct-10	active follow	9	36.3	No
7	28-Mar-10	Female	low transmission	12-Nov-10	active follow	9	36.8	No
8	28-Mar-10	Female	low transmission	19-Nov-10	passive follow	343592	36.7	No
9	28-Mar-10	Female	low transmission	16-Dec-10	active follow	9	36.5	No
10	28-Mar-10	Female	low transmission	18-Jan-11	active follow	9	36	No
11	28-Mar-10	Female	low transmission	18-Feb-11	active follow	9	36.1	No
12	28-Mar-10	Female	low transmission	16-Mar-11	active follow	9	36.4	No
1	30-Mar-10	Male	low transmission	5-May-10	active follow	9	36.6	No
2	30-Mar-10	Male	low transmission	5-Jun-10	active follow	9	36.7	No
3	30-Mar-10	Male	low transmission	14-Jul-10	active follow	9	36.2	No
4	30-Mar-10	Male	low transmission	27-Jul-10	passive follow	9	36.6	Yes
5	30-Mar-10	Male	low transmission	14-Aug-10	active follow	9	36.3	No
6	30-Mar-10	Male	low transmission	2-Sep-10	passive follow	9	36.8	No
7	30-Mar-10	Male	low transmission	14-Sep-10	active follow		36.8	No
8	30-Mar-10	Male	low transmission	21-Oct-10	active follow			
9	30-Mar-10	Male	low transmission	27-Nov-10	active follow			
10	30-Mar-10	Male	low transmission	7-Dec-10	passive follow	891542	38.6	Yes
11	30-Mar-10	Male	low transmission	27-Dec-10	active follow			
12	30-Mar-10	Male	low transmission	26-Jan-11	active follow			
13	30-Mar-10	Male	low transmission	18-Feb-11	active follow	3920	36	No
14	30-Mar-10	Male	low transmission	13-Mar-11	active follow		36	No
1	12-Apr-10	Female	high transmission	20-May-10	active follow	9	36.9	No
2	12-Apr-10	Female	high transmission	8-Jun-10	passive follow	9	37.1	Yes
3	12-Apr-10	Female	high transmission	28-Jun-10	active follow	135	36.5	No
4	12-Apr-10	Female	high transmission	3-Aug-10	active follow	2822	36.1	No
5	12-Apr-10	Female	high transmission	8-Sep-10	active follow	16207	38.6	Yes
6	12-Apr-10	Female	high transmission	19-Oct-10	active follow	3256	38.9	Yes
7	12-Apr-10	Female	high transmission	23-Nov-10	active follow	9	36.4	No
8	12-Apr-10	Female	high transmission	2-Jan-11	active follow	9	36.1	No
9	12-Apr-10	Female	high transmission	6-Feb-11	active follow	9	36.3	No
10	12-Apr-10	Female	high transmission	14-Mar-11	active follow			
11	12-Apr-10	Female	high transmission	7-Apr-11	active follow	9	37.6	Yes
1	13-Dec-08	Male	low transmission	14-Jan-09	active follow		36.6	No
2	13-Dec-08	Male	low transmission	21-Feb-09	active follow	9	36	No
3	13-Dec-08	Male	low transmission	23-Mar-09	active follow	1818	37	No
4	13-Dec-08	Male	low transmission	3-May-09	active follow	9	36	No
5	13-Dec-08	Male	low transmission	4-Jun-09	active follow	9	36.8	Yes
6	13-Dec-08	Male	low transmission	12-Jul-09	active follow	9	36.6	No
7	13-Dec-08	Male	low transmission	17-Aug-09	active follow	1206	36	No
8	13-Dec-08	Male	low transmission	16-Sep-09	active follow	9	36	No

9	13-Dec-08	Male	low transmission	23-Oct-09	active follow			
10	13-Dec-08	Male	low transmission	26-Oct-09	passive follow	226	37	Yes
11	13-Dec-08	Male	low transmission	29-Nov-09	active follow		36.8	Yes
1	8-Apr-10	Male	high transmission	5-May-10	active follow	9	36.6	No
2	8-Apr-10	Male	high transmission	4-Jun-10	active follow	9	36.2	No
3	8-Apr-10	Male	high transmission	10-Jul-10	active follow	9	35.9	No
4	8-Apr-10	Male	high transmission	14-Aug-10	active follow	9	35.3	No
5	8-Apr-10	Male	high transmission	14-Sep-10	active follow	9	36	No
6	8-Apr-10	Male	high transmission	4-Oct-10	passive follow	22691	38.9	Yes
7	8-Apr-10	Male	high transmission	18-Oct-10	active follow	9	36.3	No
8	8-Apr-10	Male	high transmission	28-Oct-10	passive follow	151302		
9	8-Apr-10	Male	high transmission	22-Nov-10	active follow	1653	36.3	No
10	8-Apr-10	Male	high transmission	29-Dec-10	active follow	9	36.8	No
11	8-Apr-10	Male	high transmission	4-Feb-11	active follow			
12	8-Apr-10	Male	high transmission	27-Feb-11	active follow	11784	36	No
13	8-Apr-10	Male	high transmission	24-Mar-11	active follow	4345	36.1	No
1	13-Apr-10	Female	high transmission	11-May-10	active follow	9	36.4	No
2	13-Apr-10	Female	high transmission	10-Jun-10	active follow	9	36	No
3	13-Apr-10	Female	high transmission	7-Jul-10	active follow	9	35.8	No
4	13-Apr-10	Female	high transmission	5-Aug-10	active follow	9	36	No
5	13-Apr-10	Female	high transmission	8-Sep-10	active follow	9	36	No
6	13-Apr-10	Female	high transmission	9-Oct-10	active follow	9	36.3	No
7	13-Apr-10	Female	high transmission	21-Oct-10	passive follow	9		
8	13-Apr-10	Female	high transmission	11-Nov-10	active follow	902	36.6	No
9	13-Apr-10	Female	high transmission	19-Nov-10	passive follow	90233		
10	13-Apr-10	Female	high transmission	10-Dec-10	active follow	9	36	No
11	13-Apr-10	Female	high transmission	17-Jan-11	active follow			
12	13-Apr-10	Female	high transmission	15-Feb-11	active follow	9	36	No
13	13-Apr-10	Female	high transmission	22-Mar-11	active follow			
1	13-Apr-10	Female	high transmission	11-May-10	active follow	9	36	No
2	13-Apr-10	Female	high transmission	3-Jun-10	passive follow	9	36.1	No
3	13-Apr-10	Female	high transmission	16-Jun-10	active follow	9	36	No
4	13-Apr-10	Female	high transmission	14-Jul-10	active follow	9	35.8	No
5	13-Apr-10	Female	high transmission	17-Aug-10	passive follow	9	36	Yes
6	13-Apr-10	Female	high transmission	17-Sep-10	active follow	9	36.7	No
7	13-Apr-10	Female	high transmission	22-Oct-10	active follow	27171	38.6	Yes
8	13-Apr-10	Female	high transmission	22-Nov-10	active follow	9355	36.1	No
9	13-Apr-10	Female	high transmission	21-Dec-10	active follow	1647	36.2	No
10	13-Apr-10	Female	high transmission	21-Jan-11	active follow	9	37	No
11	13-Apr-10	Female	high transmission	24-Feb-11	active follow	4547	36.4	No
12	13-Apr-10	Female	high transmission	17-Mar-11	active follow	9	35.6	No
13	13-Apr-10	Female	high transmission	28-Mar-11	passive follow		38.4	Yes
14	13-Apr-10	Female	high transmission	11-Apr-11	active follow		36.4	No
1	15-Apr-10	Female	high transmission	20-Apr-10	passive follow		36.7	No
2	15-Apr-10	Female	high transmission	22-Apr-10	passive follow	9		
3	15-Apr-10	Female	high transmission	11-May-10	active follow	9	37	No
4	15-Apr-10	Female	high transmission	8-Jun-10	active follow	9	36.3	No

5	15-Apr-10	Female	high transmission	12-Jul-10	active follow	9	36.5	No
6	15-Apr-10	Female	high transmission	13-Jul-10	passive follow		37	Yes
7	15-Apr-10	Female	high transmission	18-Aug-10	active follow	9	35.4	No
8	15-Apr-10	Female	high transmission	24-Sep-10	active follow			
9	15-Apr-10	Female	high transmission	16-Oct-10	passive follow	145988	39.1	Yes
10	15-Apr-10	Female	high transmission	24-Oct-10	active follow			
11	15-Apr-10	Female	high transmission	9-Nov-10	passive follow	9	35.8	Yes
12	15-Apr-10	Female	high transmission	23-Nov-10	active follow			
13	15-Apr-10	Female	high transmission	23-Dec-10	active follow			
14	15-Apr-10	Female	high transmission	29-Jan-11	active follow	4464	36	No
15	15-Apr-10	Female	high transmission	12-Feb-11	passive follow	9	38.1	Yes
16	15-Apr-10	Female	high transmission	5-Mar-11	active follow	9	36.4	No
17	15-Apr-10	Female	high transmission	5-Apr-11	active follow	670	36.3	No
1	17-Apr-10	Male	high transmission	20-May-10	active follow	9	36.6	No
2	17-Apr-10	Male	high transmission	25-May-10	passive follow	9	36.5	No
3	17-Apr-10	Male	high transmission	28-Jun-10	active follow	9	36.4	No
4	17-Apr-10	Male	high transmission	27-Jul-10	passive follow		36.4	No
5	17-Apr-10	Male	high transmission	2-Aug-10	active follow	9	36.7	No
6	17-Apr-10	Male	high transmission	31-Aug-10	active follow	9	36.8	No
7	17-Apr-10	Male	high transmission	7-Oct-10	active follow			
8	17-Apr-10	Male	high transmission	8-Nov-10	active follow	2654	37.7	Yes
9	17-Apr-10	Male	high transmission	19-Dec-10	active follow	6295	37	No
10	17-Apr-10	Male	high transmission	3-Jan-11	passive follow		38.9	Yes
11	17-Apr-10	Male	high transmission	25-Jan-11	active follow		36.2	No
12	17-Apr-10	Male	high transmission	2-Feb-11	passive follow	9	36.9	Yes
13	17-Apr-10	Male	high transmission	28-Feb-11	active follow	9	36.8	No
14	17-Apr-10	Male	high transmission	31-Mar-11	active follow	9	36	No
1	16-Apr-10	Male	high transmission	22-May-10	active follow		36.5	No
2	16-Apr-10	Male	high transmission	1-Jul-10	active follow	9	36.6	No
3	16-Apr-10	Male	high transmission	6-Aug-10	active follow	9	36	No
4	16-Apr-10	Male	high transmission	8-Sep-10	active follow	253	36	No
5	16-Apr-10	Male	high transmission	15-Oct-10	active follow	6986	36.8	No
6	16-Apr-10	Male	high transmission	23-Nov-10	active follow	12573	36.3	No
7	16-Apr-10	Male	high transmission	30-Dec-10	active follow			
8	16-Apr-10	Male	high transmission	12-Jan-11	passive follow	81	36.4	No
9	16-Apr-10	Male	high transmission	1-Feb-11	active follow	9	36.1	No
10	16-Apr-10	Male	high transmission	3-Mar-11	active follow	9	36	No
11	16-Apr-10	Male	high transmission	8-Apr-11	active follow	9	36.5	No
1	17-Apr-10	Female	high transmission	20-May-10	active follow	9	36.3	No
2	17-Apr-10	Female	high transmission	15-Jun-10	active follow	9	36.6	No
3	17-Apr-10	Female	high transmission	15-Jul-10	active follow	9	36.4	No
4	17-Apr-10	Female	high transmission	27-Aug-10	active follow	789	36.3	No
5	17-Apr-10	Female	high transmission	30-Aug-10	passive follow	9	36	No
6	17-Apr-10	Female	high transmission	27-Sep-10	passive follow	9	36	No
7	17-Apr-10	Female	high transmission	8-Oct-10	active follow	9	36.2	No
8	17-Apr-10	Female	high transmission	14-Nov-10	active follow			
9	17-Apr-10	Female	high transmission	21-Dec-10	active follow			

10	17-Apr-10	Female	high transmission	7-Jan-11	passive follow	9	36.1	No
11	17-Apr-10	Female	high transmission	27-Jan-11	active follow	9	36.9	No
12	17-Apr-10	Female	high transmission	26-Feb-11	passive follow	173647	39.1	Yes
13	17-Apr-10	Female	high transmission	28-Feb-11	active follow		36.6	No
14	17-Apr-10	Female	high transmission	3-Apr-11	active follow	2964	36.5	No
1	19-Apr-10	Female	high transmission	26-May-10	active follow	9	36.2	No
2	19-Apr-10	Female	high transmission	2-Jul-10	active follow	6986	36.5	No
3	19-Apr-10	Female	high transmission	17-Aug-10	active follow	9	36.1	No
4	19-Apr-10	Female	high transmission	21-Sep-10	active follow	9	36.8	No
5	19-Apr-10	Female	high transmission	25-Oct-10	active follow	9	36.5	No
6	19-Apr-10	Female	high transmission	1-Dec-10	active follow			
7	19-Apr-10	Female	high transmission	7-Dec-10	passive follow	9	37	Yes
8	19-Apr-10	Female	high transmission	7-Jan-11	active follow	9	36.7	No
9	19-Apr-10	Female	high transmission	20-Jan-11	passive follow	9	40	Yes
10	19-Apr-10	Female	high transmission	13-Feb-11	active follow	9	36	No
11	19-Apr-10	Female	high transmission	15-Feb-11	passive follow	9	36.1	No
12	19-Apr-10	Female	high transmission	14-Mar-11	active follow	9	36.3	No
13	19-Apr-10	Female	high transmission	15-Apr-11	active follow	235968	40.1	Yes
1	27-Apr-10	Female	high transmission	26-May-10	active follow	9	36.8	No
2	27-Apr-10	Female	high transmission	29-Jun-10	active follow	9	36.7	No
3	27-Apr-10	Female	high transmission	27-Jul-10	active follow		36.7	No
4	27-Apr-10	Female	high transmission	26-Aug-10	active follow	2550	37.1	No
5	27-Apr-10	Female	high transmission	9-Sep-10	passive follow	35056	36.9	Yes
6	27-Apr-10	Female	high transmission	28-Sep-10	active follow	1529	36.8	No
7	27-Apr-10	Female	high transmission	25-Oct-10	active follow	2657	36.7	No
8	27-Apr-10	Female	high transmission	22-Nov-10	active follow	860	36.8	No
9	27-Apr-10	Female	high transmission	30-Nov-10	passive follow	5953	37.6	Yes
10	27-Apr-10	Female	high transmission	20-Dec-10	active follow	9	36	No
11	27-Apr-10	Female	high transmission	18-Jan-11	active follow	9	36	No
12	27-Apr-10	Female	high transmission	14-Feb-11	active follow	9	36.3	No
13	27-Apr-10	Female	high transmission	11-Mar-11	active follow	9	36.3	No
14	27-Apr-10	Female	high transmission	31-Mar-11	active follow	156683	37.1	Yes
1	27-Apr-10	Male	high transmission	28-May-10	active follow	9	36.6	No
2	27-Apr-10	Male	high transmission	8-Jul-10	active follow	9	36.7	No
3	27-Apr-10	Male	high transmission	9-Aug-10	active follow	111659	36.3	No
4	27-Apr-10	Male	high transmission	10-Sep-10	active follow	9	36.8	No
5	27-Apr-10	Male	high transmission	4-Oct-10	passive follow	1035	37.6	Yes
6	27-Apr-10	Male	high transmission	18-Oct-10	active follow	9	36.2	No
7	27-Apr-10	Male	high transmission	19-Nov-10	active follow	9	36.9	No
8	27-Apr-10	Male	high transmission	26-Dec-10	active follow	9	36.2	No
9	27-Apr-10	Male	high transmission	27-Jan-11	active follow	9	36	No
10	27-Apr-10	Male	high transmission	23-Feb-11	active follow	9	36.6	No
11	27-Apr-10	Male	high transmission	4-Mar-11	passive follow	9	37.1	No
12	27-Apr-10	Male	high transmission	23-Mar-11	active follow	9	36.5	No
13	27-Apr-10	Male	high transmission	18-Apr-11	active follow	9	37.1	No
1	29-Apr-10	Male	high transmission	24-May-10	active follow		36	No
2	29-Apr-10	Male	high transmission	28-Jun-10	active follow	9	36.5	No

3	29-Apr-10	Male	high transmission	29-Jul-10	active follow	9	35.3	No
4	29-Apr-10	Male	high transmission	6-Sep-10	active follow	9	36.4	No
5	29-Apr-10	Male	high transmission	13-Oct-10	active follow			
6	29-Apr-10	Male	high transmission	19-Nov-10	active follow	9	36.4	No
7	29-Apr-10	Male	high transmission	21-Dec-10	active follow		36.3	No
8	29-Apr-10	Male	high transmission	25-Jan-11	active follow	177	36.8	No
9	29-Apr-10	Male	high transmission	26-Feb-11	active follow	9	35.4	No
10	29-Apr-10	Male	high transmission	28-Mar-11	active follow	9	36.4	No
11	29-Apr-10	Male	high transmission	20-Apr-11	active follow	202945	38.1	Yes
1	22-Mar-10	Male	low transmission	29-Apr-10	active follow			
2	22-Mar-10	Male	low transmission	29-May-10	active follow			
3	22-Mar-10	Male	low transmission	27-Jun-10	active follow	9	36.8	No
4	22-Mar-10	Male	low transmission	3-Aug-10	active follow	9	36.4	No
5	22-Mar-10	Male	low transmission	6-Aug-10	passive follow	9	37.1	Yes
6	22-Mar-10	Male	low transmission	15-Sep-10	active follow	9	36.3	No
7	22-Mar-10	Male	low transmission	20-Oct-10	active follow	9	36.4	No
8	22-Mar-10	Male	low transmission	24-Nov-10	active follow	9	36.8	No
9	22-Mar-10	Male	low transmission	30-Dec-10	active follow	148	36.6	No
10	22-Mar-10	Male	low transmission	7-Jan-11	passive follow	7056	37.5	Yes
11	22-Mar-10	Male	low transmission	27-Jan-11	active follow	9	36.6	No
12	22-Mar-10	Male	low transmission	3-Mar-11	active follow	9	36.2	No
13	22-Mar-10	Male	low transmission	15-Mar-11	passive follow	9	39.1	Yes
1	18-Dec-08	Female	low transmission	28-Jan-09	active follow	9	36	No
2	18-Dec-08	Female	low transmission	3-Mar-09	active follow			
3	18-Dec-08	Female	low transmission	10-Mar-09	passive follow	625		
4	18-Dec-08	Female	low transmission	2-Apr-09	active follow	9	36.1	No
5	18-Dec-08	Female	low transmission	28-Apr-09	active follow	9	36	No
6	18-Dec-08	Female	low transmission	2-Jun-09	active follow	9	36.5	No
7	18-Dec-08	Female	low transmission	7-Jul-09	active follow	106	36.2	No
8	18-Dec-08	Female	low transmission	10-Aug-09	active follow	208	36.2	No
9	18-Dec-08	Female	low transmission	18-Aug-09	passive follow	1915	36.3	Yes
10	18-Dec-08	Female	low transmission	9-Sep-09	active follow	9	36.4	No
11	18-Dec-08	Female	low transmission	9-Oct-09	active follow		36	No
12	18-Dec-08	Female	low transmission	14-Oct-09	passive follow	9	37.1	Yes
13	18-Dec-08	Female	low transmission	11-Nov-09	active follow	9	35.5	No
14	18-Dec-08	Female	low transmission	19-Nov-09	passive follow		39.8	Yes
15	18-Dec-08	Female	low transmission	18-Dec-09	active follow			
1	26-Apr-10	Female	high transmission	2-Jun-10	active follow	9	36.6	No
2	26-Apr-10	Female	high transmission	6-Jul-10	active follow	9	36.4	No
3	26-Apr-10	Female	high transmission	7-Aug-10	active follow	14337	36	No
4	26-Apr-10	Female	high transmission	12-Aug-10	passive follow	54734	36.8	Yes
5	26-Apr-10	Female	high transmission	13-Sep-10	active follow			
6	26-Apr-10	Female	high transmission	13-Oct-10	active follow			
7	26-Apr-10	Female	high transmission	19-Oct-10	passive follow	15755	38	Yes
8	26-Apr-10	Female	high transmission	10-Nov-10	passive follow	132166	38.7	Yes
9	26-Apr-10	Female	high transmission	12-Nov-10	active follow			
10	26-Apr-10	Female	high transmission	12-Dec-10	active follow			

11	26-Apr-10	Female	high transmission	11-Jan-11	active follow			
12	26-Apr-10	Female	high transmission	10-Feb-11	active follow			
13	26-Apr-10	Female	high transmission	28-Feb-11	active follow	586	36	No
14	26-Apr-10	Female	high transmission	23-Mar-11	active follow	9	35.7	No
15	26-Apr-10	Female	high transmission	16-Apr-11	active follow		35.8	No
1	1-Apr-10	Male	high transmission	8-May-10	active follow			
2	1-Apr-10	Male	high transmission	11-Jun-10	active follow	9	36.6	No
3	1-Apr-10	Male	high transmission	15-Jun-10	passive follow	9	36.5	Yes
4	1-Apr-10	Male	high transmission	5-Jul-10	active follow	9	36.5	No
5	1-Apr-10	Male	high transmission	10-Aug-10	active follow	5347	36.5	Yes
6	1-Apr-10	Male	high transmission	16-Aug-10	active follow	5347	36.1	Yes
7	1-Apr-10	Male	high transmission	17-Sep-10	active follow	23633	37.4	Yes
8	1-Apr-10	Male	high transmission	25-Oct-10	active follow	13771	36.9	No
9	1-Apr-10	Male	high transmission	26-Oct-10	passive follow		36.9	Yes
10	1-Apr-10	Male	high transmission	27-Nov-10	active follow	9	36.8	No
11	1-Apr-10	Male	high transmission	21-Dec-10	passive follow	22373	39.1	Yes
12	1-Apr-10	Male	high transmission	31-Dec-10	active follow		36.7	No
13	1-Apr-10	Male	high transmission	5-Jan-11	passive follow	9	36.8	Yes
14	1-Apr-10	Male	high transmission	6-Feb-11	active follow	9	36.9	No
15	1-Apr-10	Male	high transmission	15-Feb-11	passive follow	51182	39.2	Yes
16	1-Apr-10	Male	high transmission	7-Mar-11	active follow	9	36.3	No
1	22-Apr-10	Female	high transmission	22-May-10	active follow	9	36.5	No
2	22-Apr-10	Female	high transmission	26-Jun-10	active follow		36.5	No
3	22-Apr-10	Female	high transmission	29-Jul-10	active follow	3656	36.2	No
4	22-Apr-10	Female	high transmission	24-Aug-10	active follow	3137	36.4	No
5	22-Apr-10	Female	high transmission	23-Sep-10	active follow	11990		
6	22-Apr-10	Female	high transmission	24-Sep-10	passive follow		37	Yes
7	22-Apr-10	Female	high transmission	29-Sep-10	active follow	11990	37	Yes
8	22-Apr-10	Female	high transmission	18-Oct-10	passive follow	77862	37.3	Yes
9	22-Apr-10	Female	high transmission	26-Oct-10	active follow	9	36.1	No
10	22-Apr-10	Female	high transmission	23-Nov-10	active follow	9	36.3	Yes
11	22-Apr-10	Female	high transmission	21-Dec-10	active follow	9	36.3	Yes
12	22-Apr-10	Female	high transmission	20-Jan-11	active follow	9	36.5	Yes
13	22-Apr-10	Female	high transmission	15-Feb-11	active follow	9	36.7	No
14	22-Apr-10	Female	high transmission	10-Mar-11	active follow	9	36.3	No
15	22-Apr-10	Female	high transmission	23-Mar-11	passive follow	9	36.2	Yes
16	22-Apr-10	Female	high transmission	4-Apr-11	active follow	9	36.6	No
17	22-Apr-10	Female	high transmission	12-Apr-11	passive follow	9		
1	22-Apr-10	Female	high transmission	25-May-10	active follow	9	36.8	No
2	22-Apr-10	Female	high transmission	30-Jun-10	active follow	9	35.5	No
3	22-Apr-10	Female	high transmission	14-Jul-10	passive follow	9	36.2	Yes
4	22-Apr-10	Female	high transmission	29-Jul-10	active follow	9	36	No
5	22-Apr-10	Female	high transmission	2-Aug-10	active follow	9		
6	22-Apr-10	Female	high transmission	27-Aug-10	active follow	9	36	Yes
7	22-Apr-10	Female	high transmission	30-Sep-10	active follow	264	36.3	No
8	22-Apr-10	Female	high transmission	24-Oct-10	passive follow		36	Yes
9	22-Apr-10	Female	high transmission	25-Oct-10	passive follow	9		

10	22-Apr-10	Female	high transmission	6-Nov-10	active follow			
11	22-Apr-10	Female	high transmission	6-Dec-10	active follow			
12	22-Apr-10	Female	high transmission	9-Dec-10	passive follow	183604		
13	22-Apr-10	Female	high transmission	5-Jan-11	active follow			
14	22-Apr-10	Female	high transmission	11-Feb-11	active follow			
15	22-Apr-10	Female	high transmission	28-Feb-11	passive follow	9	36.2	Yes
16	22-Apr-10	Female	high transmission	6-Mar-11	active follow		36.2	No
17	22-Apr-10	Female	high transmission	5-Apr-11	active follow	9	36.5	No
18	22-Apr-10	Female	high transmission	13-Apr-11	passive follow	9	36.5	Yes
1	29-Apr-10	Female	high transmission	26-May-10	active follow	9	36	No
2	29-Apr-10	Female	high transmission	24-Jun-10	active follow	9	36.7	No
3	29-Apr-10	Female	high transmission	19-Jul-10	active follow	9	36	No
4	29-Apr-10	Female	high transmission	16-Aug-10	active follow	9	36.8	No
5	29-Apr-10	Female	high transmission	9-Sep-10	passive follow	7878	36	Yes
6	29-Apr-10	Female	high transmission	15-Sep-10	active follow	9	35.1	No
7	29-Apr-10	Female	high transmission	22-Oct-10	active follow	9	37	No
8	29-Apr-10	Female	high transmission	27-Nov-10	active follow	5327	37.2	No
9	29-Apr-10	Female	high transmission	31-Dec-10	active follow	5449	36.8	No
10	29-Apr-10	Female	high transmission	5-Feb-11	active follow	718		No
11	29-Apr-10	Female	high transmission	23-Feb-11	passive follow	9	37.8	Yes
12	29-Apr-10	Female	high transmission	3-Mar-11	active follow	9	36.3	No
13	29-Apr-10	Female	high transmission	26-Mar-11	active follow	9	35.7	No
14	29-Apr-10	Female	high transmission	18-Apr-11	active follow	555	36.5	No
1	28-Apr-10	Male	high transmission	25-May-10	active follow	9	36.8	No
2	28-Apr-10	Male	high transmission	21-Jun-10	passive follow	9	36.2	No
3	28-Apr-10	Male	high transmission	28-Jun-10	active follow	9	36.6	No
4	28-Apr-10	Male	high transmission	28-Jul-10	active follow		36.6	No
5	28-Apr-10	Male	high transmission	13-Aug-10	passive follow	3506	37.7	Yes
6	28-Apr-10	Male	high transmission	26-Aug-10	active follow	9	36.8	No
7	28-Apr-10	Male	high transmission	30-Sep-10	active follow	9	36.5	No
8	28-Apr-10	Male	high transmission	1-Nov-10	active follow	111051	37	No
9	28-Apr-10	Male	high transmission	7-Dec-10	active follow	9	36.2	No
10	28-Apr-10	Male	high transmission	7-Jan-11	active follow	9	36.9	No
11	28-Apr-10	Male	high transmission	14-Feb-11	active follow	9	36.6	No
12	28-Apr-10	Male	high transmission	11-Mar-11	active follow	9	36.7	Yes
13	28-Apr-10	Male	high transmission	5-Apr-11	active follow	9	36	No
1	7-May-10	Female	high transmission	10-Jun-10	active follow	9	36.6	No
2	7-May-10	Female	high transmission	6-Jul-10	active follow	9	36.1	No
3	7-May-10	Female	high transmission	6-Aug-10	active follow	9	35.4	No
4	7-May-10	Female	high transmission	18-Aug-10	passive follow	9	37	Yes
5	7-May-10	Female	high transmission	8-Sep-10	active follow	9	36.7	No
6	7-May-10	Female	high transmission	7-Oct-10	active follow	9	36.2	No
7	7-May-10	Female	high transmission	4-Nov-10	active follow	1265	35.8	No
8	7-May-10	Female	high transmission	2-Dec-10	active follow	9	36	No
9	7-May-10	Female	high transmission	6-Dec-10	passive follow		36.7	Yes
10	7-May-10	Female	high transmission	29-Dec-10	active follow	57383	38.4	Yes
11	7-May-10	Female	high transmission	26-Jan-11	active follow	6571	36.5	No

12	7-May-10	Female	high transmission	5-Feb-11	passive follow	98780	38.9	Yes
13	7-May-10	Female	high transmission	24-Feb-11	active follow	9	35.8	No
14	7-May-10	Female	high transmission	21-Mar-11	active follow	90049	37.4	Yes
15	7-May-10	Female	high transmission	14-Apr-11	active follow	1641	36.5	No
16	7-May-10	Female	high transmission	7-May-11	passive follow	435160	38.2	Yes
1	7-May-10	Female	high transmission	8-Jun-10	active follow	9	36.4	No
2	7-May-10	Female	high transmission	12-Jun-10	passive follow	9	37.3	Yes
3	7-May-10	Female	high transmission	2-Jul-10	passive follow	9	36.4	Yes
4	7-May-10	Female	high transmission	7-Jul-10	active follow	9	36.7	No
5	7-May-10	Female	high transmission	13-Aug-10	active follow			
6	7-May-10	Female	high transmission	16-Sep-10	active follow	9	36.2	No
7	7-May-10	Female	high transmission	14-Oct-10	active follow	1440	36.4	No
8	7-May-10	Female	high transmission	17-Oct-10	passive follow	25242	37.5	Yes
9	7-May-10	Female	high transmission	20-Nov-10	active follow			
10	7-May-10	Female	high transmission	27-Dec-10	active follow			
11	7-May-10	Female	high transmission	1-Feb-11	active follow			
12	7-May-10	Female	high transmission	2-Mar-11	active follow	18732	36.8	No
13	7-May-10	Female	high transmission	24-Mar-11	passive follow		38.1	Yes
14	7-May-10	Female	high transmission	6-Apr-11	active follow		36.7	No
15	7-May-10	Female	high transmission	20-Apr-11	passive follow	98340	37.8	Yes
1	10-May-10	Male	high transmission	10-Jun-10	active follow	9	36.3	No
2	10-May-10	Male	high transmission	12-Jul-10	active follow	9	36.3	No
3	10-May-10	Male	high transmission	18-Aug-10	active follow			
4	10-May-10	Male	high transmission	22-Sep-10	active follow	706	35.3	No
5	10-May-10	Male	high transmission	30-Oct-10	active follow			
6	10-May-10	Male	high transmission	29-Nov-10	active follow	11227	37.6	Yes
7	10-May-10	Male	high transmission	29-Dec-10	active follow	9	36.3	No
8	10-May-10	Male	high transmission	4-Jan-11	passive follow	9	36	No
9	10-May-10	Male	high transmission	21-Jan-11	passive follow	9	37.4	Yes
10	10-May-10	Male	high transmission	31-Jan-11	active follow	9	36.3	No
11	10-May-10	Male	high transmission	4-Mar-11	active follow	9	36.5	No
12	10-May-10	Male	high transmission	28-Mar-11	active follow	9	36.3	Yes
13	10-May-10	Male	high transmission	22-Apr-11	active follow	9	36.8	No
1	1-May-10	Male	high transmission	4-Jun-10	active follow	9	36	No
2	1-May-10	Male	high transmission	11-Jul-10	active follow			
3	1-May-10	Male	high transmission	1-Aug-10	passive follow		36.8	No
4	1-May-10	Male	high transmission	10-Aug-10	active follow			
5	1-May-10	Male	high transmission	30-Aug-10	passive follow	203801	37.4	Yes
6	1-May-10	Male	high transmission	9-Sep-10	active follow	9	36.3	No
7	1-May-10	Male	high transmission	7-Oct-10	active follow	264044	36.5	Yes
8	1-May-10	Male	high transmission	2-Nov-10	active follow	9	36.9	No
9	1-May-10	Male	high transmission	14-Nov-10	passive follow	166314	39.8	Yes
10	1-May-10	Male	high transmission	30-Nov-10	active follow	9	36.8	No
11	1-May-10	Male	high transmission	29-Dec-10	active follow	6435	36.3	No
12	1-May-10	Male	high transmission	3-Jan-11	passive follow	26355	37.2	Yes
13	1-May-10	Male	high transmission	31-Jan-11	active follow	9	36.3	No
14	1-May-10	Male	high transmission	3-Mar-11	active follow	4045	36	No

15	1-May-10	Male	high transmission	6-Mar-11	passive follow	109427	37.3	Yes
16	1-May-10	Male	high transmission	26-Mar-11	active follow	21313	36	No
17	1-May-10	Male	high transmission	9-Apr-11	passive follow	103989	37.2	Yes
18	1-May-10	Male	high transmission	23-Apr-11	active follow	9	36	No
1	3-May-10	Female	high transmission	24-May-10	passive follow		37.3	No
2	3-May-10	Female	high transmission	5-Jun-10	active follow	9	36.1	No
3	3-May-10	Female	high transmission	9-Jul-10	active follow			
4	3-May-10	Female	high transmission	31-Jul-10	passive follow	9	36.2	No
5	3-May-10	Female	high transmission	8-Aug-10	active follow			
6	3-May-10	Female	high transmission	30-Sep-10	active follow	885	36.5	No
7	3-May-10	Female	high transmission	31-Oct-10	active follow	71	38	Yes
8	3-May-10	Female	high transmission	8-Dec-10	active follow	9	36.6	No
9	3-May-10	Female	high transmission	14-Jan-11	active follow	55203	39	Yes
10	3-May-10	Female	high transmission	15-Feb-11	active follow	1403	36.4	No
11	3-May-10	Female	high transmission	17-Mar-11	active follow	465906	39.3	Yes
12	3-May-10	Female	high transmission	19-Apr-11	active follow	16685	36.8	No
13	3-May-10	Female	high transmission	23-Apr-11	passive follow	49564	36.5	Yes
1	8-May-10	Female	high transmission	16-Jun-10	active follow	9	36.3	No
2	8-May-10	Female	high transmission	16-Jul-10	passive follow	9	36.9	No
3	8-May-10	Female	high transmission	26-Jul-10	active follow			
4	8-May-10	Female	high transmission	31-Aug-10	active follow	9	36	No
5	8-May-10	Female	high transmission	2-Oct-10	active follow	9	36.4	Yes
6	8-May-10	Female	high transmission	23-Oct-10	passive follow	20332	36.5	Yes
7	8-May-10	Female	high transmission	8-Nov-10	active follow	8377	37	Yes
8	8-May-10	Female	high transmission	13-Nov-10	active follow	8377	36.2	No
9	8-May-10	Female	high transmission	15-Nov-10	passive follow	9		
10	8-May-10	Female	high transmission	15-Dec-10	active follow	9	36.5	No
11	8-May-10	Female	high transmission	26-Jan-11	active follow			
12	8-May-10	Female	high transmission	21-Feb-11	active follow	3237	36.1	No
13	8-May-10	Female	high transmission	25-Feb-11	passive follow	29239	37.2	Yes
14	8-May-10	Female	high transmission	18-Mar-11	active follow	9	36.8	No
15	8-May-10	Female	high transmission	11-Apr-11	active follow	9	36	No
16	8-May-10	Female	high transmission	4-May-11	active follow	242402	37.4	Yes
1	9-May-10	Female	high transmission	16-Jun-10	active follow	9	36.9	No
2	9-May-10	Female	high transmission	21-Jul-10	active follow	9	36.2	No
3	9-May-10	Female	high transmission	27-Aug-10	active follow	11277	36.6	No
4	9-May-10	Female	high transmission	8-Oct-10	active follow	9	36.4	No
5	9-May-10	Female	high transmission	9-Nov-10	active follow	9	37.5	Yes
6	9-May-10	Female	high transmission	9-Dec-10	active follow	9	36.7	No
7	9-May-10	Female	high transmission	3-Jan-11	passive follow		38.6	Yes
8	9-May-10	Female	high transmission	12-Jan-11	active follow	9	36	No
9	9-May-10	Female	high transmission	15-Feb-11	active follow	9	36.8	No
10	9-May-10	Female	high transmission	21-Feb-11	passive follow	9	36.5	Yes
11	9-May-10	Female	high transmission	16-Mar-11	active follow		37.9	Yes
12	9-May-10	Female	high transmission	17-Apr-11	active follow		36.6	No
13	9-May-10	Female	high transmission	26-Apr-11	passive follow	29689	38.9	Yes
1	5-May-10	Male	high transmission	25-Jun-10	active follow		35.1	No

2	5-May-10	Male	high transmission	26-Jun-10	passive follow	9		
3	5-May-10	Male	high transmission	29-Jul-10	active follow	9	36.3	No
4	5-May-10	Male	high transmission	30-Aug-10	active follow	9	36.3	No
5	5-May-10	Male	high transmission	4-Oct-10	active follow	9	36.4	No
6	5-May-10	Male	high transmission	10-Nov-10	active follow	755	36	No
7	5-May-10	Male	high transmission	6-Dec-10	passive follow	1245		
8	5-May-10	Male	high transmission	11-Dec-10	active follow	9	36.4	No
9	5-May-10	Male	high transmission	30-Dec-10	passive follow	9		
10	5-May-10	Male	high transmission	16-Jan-11	active follow			
11	5-May-10	Male	high transmission	9-Feb-11	passive follow		37.7	Yes
12	5-May-10	Male	high transmission	12-Feb-11	passive follow	9		
13	5-May-10	Male	high transmission	17-Feb-11	active follow	9	36.1	No
14	5-May-10	Male	high transmission	22-Feb-11	passive follow		37.5	Yes
15	5-May-10	Male	high transmission	20-Mar-11	active follow	9	36.5	No
16	5-May-10	Male	high transmission	13-Apr-11	active follow	9	36.2	No
1	1-May-10	Male	high transmission	1-Jun-10	active follow	9	36.5	No
2	1-May-10	Male	high transmission	22-Jun-10	passive follow	9	36.8	Yes
3	1-May-10	Male	high transmission	1-Jul-10	active follow	9	36.2	No
4	1-May-10	Male	high transmission	2-Aug-10	active follow	9	36.6	No
5	1-May-10	Male	high transmission	12-Sep-10	active follow	10092	36.3	No
6	1-May-10	Male	high transmission	18-Oct-10	active follow	10712	36.8	No
7	1-May-10	Male	high transmission	25-Oct-10	passive follow	62721	37.1	Yes
8	1-May-10	Male	high transmission	9-Nov-10	passive follow	9	36.6	Yes
9	1-May-10	Male	high transmission	24-Nov-10	active follow			
10	1-May-10	Male	high transmission	6-Dec-10	passive follow	122929	37.7	Yes
11	1-May-10	Male	high transmission	30-Dec-10	active follow		36.6	No
12	1-May-10	Male	high transmission	17-Jan-11	passive follow	9	36.8	Yes
13	1-May-10	Male	high transmission	6-Feb-11	active follow		36.1	No
14	1-May-10	Male	high transmission	3-Mar-11	active follow	9	37.5	Yes
15	1-May-10	Male	high transmission	29-Mar-11	active follow	9	36.2	No
16	1-May-10	Male	high transmission	11-Apr-11	passive follow	247558	38.5	Yes
17	1-May-10	Male	high transmission	26-Apr-11	active follow	9	36.3	No
1	11-May-10	Male	high transmission	9-Jun-10	active follow	9	36.5	No
2	11-May-10	Male	high transmission	8-Jul-10	active follow	9	36.5	No
3	11-May-10	Male	high transmission	5-Aug-10	active follow	9	36.5	No
4	11-May-10	Male	high transmission	13-Aug-10	passive follow	9	37.1	Yes
5	11-May-10	Male	high transmission	8-Sep-10	active follow	9	37	No
6	11-May-10	Male	high transmission	10-Oct-10	active follow	9	36.3	No
7	11-May-10	Male	high transmission	11-Nov-10	active follow	9	36.7	No
8	11-May-10	Male	high transmission	13-Dec-10	active follow	9	36.3	No
9	11-May-10	Male	high transmission	11-Jan-11	active follow	9	35.9	No
10	11-May-10	Male	high transmission	11-Feb-11	active follow	103	37.1	No
11	11-May-10	Male	high transmission	10-Mar-11	active follow	9	36.5	No
12	11-May-10	Male	high transmission	14-Mar-11	passive follow	6834	37.7	Yes
13	11-May-10	Male	high transmission	1-Apr-11	active follow	9	36.3	No
14	11-May-10	Male	high transmission	25-Apr-11	active follow	9	36.8	No
1	11-May-10	Male	high transmission	13-Jun-10	active follow	9	36.3	No

2	11-May-10	Male	high transmission	15-Jul-10	active follow	9	38.2	Yes
3	11-May-10	Male	high transmission	20-Aug-10	passive follow		36.6	Yes
4	11-May-10	Male	high transmission	25-Aug-10	active follow	9	36.5	No
5	11-May-10	Male	high transmission	8-Oct-10	active follow	12570	36.7	No
6	11-May-10	Male	high transmission	4-Nov-10	passive follow	5224	36.4	No
7	11-May-10	Male	high transmission	16-Nov-10	active follow	9	36.4	No
8	11-May-10	Male	high transmission	6-Dec-10	passive follow	9	37	No
9	11-May-10	Male	high transmission	4-Jan-11	active follow		36.2	No
10	11-May-10	Male	high transmission	5-Jan-11	active follow	9		
11	11-May-10	Male	high transmission	21-Jan-11	passive follow	77843	39.8	Yes
12	11-May-10	Male	high transmission	11-Feb-11	active follow	9	36.5	No
13	11-May-10	Male	high transmission	28-Feb-11	passive follow	85590	39.6	Yes
14	11-May-10	Male	high transmission	9-Mar-11	active follow	9	37.4	No
15	11-May-10	Male	high transmission	5-Apr-11	active follow		37	No
16	11-May-10	Male	high transmission	12-Apr-11	passive follow	86519	39	Yes
17	11-May-10	Male	high transmission	1-May-11	active follow	9	37.1	No
1	16-May-10	Female	high transmission	17-Jun-10	active follow	9	35.8	No
2	16-May-10	Female	high transmission	13-Jul-10	active follow	9	36	Yes
3	16-May-10	Female	high transmission	18-Aug-10	active follow	9	35.9	No
4	16-May-10	Female	high transmission	7-Sep-10	passive follow	9	36.4	No
5	16-May-10	Female	high transmission	17-Sep-10	active follow	372	36.8	No
6	16-May-10	Female	high transmission	30-Oct-10	active follow	3050	37.1	No
7	16-May-10	Female	high transmission	6-Dec-10	active follow	5565	36.6	No
8	16-May-10	Female	high transmission	6-Jan-11	active follow	6216	37	No
9	16-May-10	Female	high transmission	1-Feb-11	active follow	499	35.7	No
10	16-May-10	Female	high transmission	14-Feb-11	passive follow	9	38.2	Yes
11	16-May-10	Female	high transmission	3-Mar-11	active follow	9	37.2	No
12	16-May-10	Female	high transmission	28-Mar-11	active follow	9	36.6	No
13	16-May-10	Female	high transmission	20-Apr-11	active follow	9941	37.2	Yes
14	16-May-10	Female	high transmission	11-May-11	passive follow	9		
1	13-May-10	Male	high transmission	10-Jun-10	active follow	9	36.1	No
2	13-May-10	Male	high transmission	12-Jul-10	active follow	9	36.3	No
3	13-May-10	Male	high transmission	20-Jul-10	passive follow	9	36.4	Yes
4	13-May-10	Male	high transmission	18-Aug-10	active follow	3677	36.2	No
5	13-May-10	Male	high transmission	22-Sep-10	active follow	15599	36.5	No
6	13-May-10	Male	high transmission	27-Oct-10	active follow	2432	36.3	No
7	13-May-10	Male	high transmission	1-Dec-10	active follow	3810	35.6	No
8	13-May-10	Male	high transmission	31-Dec-10	active follow	7452	36.3	No
9	13-May-10	Male	high transmission	1-Feb-11	active follow	1020	35.8	Yes
10	13-May-10	Male	high transmission	5-Mar-11	active follow	9	37	Yes
11	13-May-10	Male	high transmission	31-Mar-11	active follow	9	36.8	No
12	13-May-10	Male	high transmission	28-Apr-11	active follow	9	36.1	No
1	19-May-10	Male	high transmission	2-Jun-10	passive follow	9		
2	19-May-10	Male	high transmission	17-Jun-10	active follow	9	36.5	No
3	19-May-10	Male	high transmission	26-Jul-10	active follow	9	36.8	No
4	19-May-10	Male	high transmission	10-Aug-10	passive follow	9		
5	19-May-10	Male	high transmission	28-Aug-10	active follow	1687	36.5	No

6	19-May-10	Male	high transmission	10-Sep-10	passive follow	772	37	Yes
7	19-May-10	Male	high transmission	30-Sep-10	active follow	9	36.7	No
8	19-May-10	Male	high transmission	7-Oct-10	passive follow	1022	38.5	Yes
9	19-May-10	Male	high transmission	1-Nov-10	active follow	9	37.2	No
10	19-May-10	Male	high transmission	29-Nov-10	active follow	234946	36.4	No
11	19-May-10	Male	high transmission	30-Nov-10	passive follow	23881	38.2	Yes
12	19-May-10	Male	high transmission	28-Dec-10	active follow	9	36.6	No
13	19-May-10	Male	high transmission	14-Jan-11	passive follow		37.8	Yes
14	19-May-10	Male	high transmission	29-Jan-11	active follow	9	36.4	No
15	19-May-10	Male	high transmission	27-Feb-11	active follow	9	36.7	No
16	19-May-10	Male	high transmission	27-Mar-11	active follow	9	35.5	No
17	19-May-10	Male	high transmission	7-Apr-11	passive follow	9	36.3	Yes
18	19-May-10	Male	high transmission	28-Apr-11	active follow	9	36.4	No
1	16-May-10	Female	high transmission	20-Jun-10	active follow	9	36.8	No
2	16-May-10	Female	high transmission	20-Jun-10	active follow	9	36.7	No
3	16-May-10	Female	high transmission	20-Jul-10	active follow	9	36.5	No
4	16-May-10	Female	high transmission	20-Jul-10	active follow	9	35.7	No
5	16-May-10	Female	high transmission	18-Aug-10	active follow	9	36.7	No
6	16-May-10	Female	high transmission	18-Aug-10	active follow	9	36.7	No
7	16-May-10	Female	high transmission	7-Sep-10	passive follow	9	36	Yes
8	16-May-10	Female	high transmission	7-Sep-10	passive follow	9	36	Yes
9	16-May-10	Female	high transmission	22-Sep-10	active follow	9	36.5	No
10	16-May-10	Female	high transmission	22-Sep-10	active follow	9	36	No
11	16-May-10	Female	high transmission	14-Oct-10	passive follow	9	36.7	Yes
12	16-May-10	Female	high transmission	26-Oct-10	active follow	9	36.1	No
13	16-May-10	Female	high transmission	26-Oct-10	active follow	9	36.1	No
14	16-May-10	Female	high transmission	10-Nov-10	passive follow	16797	37.6	Yes
15	16-May-10	Female	high transmission	26-Nov-10	active follow		36.4	No
16	16-May-10	Female	high transmission	26-Nov-10	active follow	9	36.1	No
17	16-May-10	Female	high transmission	29-Dec-10	active follow	9	36.3	No
18	16-May-10	Female	high transmission	29-Dec-10	active follow	9	36.1	No
19	16-May-10	Female	high transmission	31-Jan-11	active follow	9	36.1	No
20	16-May-10	Female	high transmission	31-Jan-11	active follow	9	36.3	No
21	16-May-10	Female	high transmission	2-Feb-11	passive follow	9	36.5	No
22	16-May-10	Female	high transmission	2-Feb-11	passive follow	9	36.5	No
23	16-May-10	Female	high transmission	5-Mar-11	active follow	9	36.3	No
24	16-May-10	Female	high transmission	5-Mar-11	active follow	9	36	No
25	16-May-10	Female	high transmission	4-Apr-11	active follow	9	36.5	No
26	16-May-10	Female	high transmission	4-Apr-11	active follow	69	37	No
27	16-May-10	Female	high transmission	27-Apr-11	active follow	963	36	No
28	16-May-10	Female	high transmission	27-Apr-11	active follow	9	36.3	No
1	18-May-10	Male	high transmission	26-May-10	passive follow		37	Yes
2	18-May-10	Male	high transmission	9-Jun-10	passive follow	9	36.7	No
3	18-May-10	Male	high transmission	16-Jun-10	active follow	9	36	No
4	18-May-10	Male	high transmission	30-Jun-10	passive follow	9	35.7	Yes
5	18-May-10	Male	high transmission	20-Jul-10	active follow			
6	18-May-10	Male	high transmission	28-Jul-10	passive follow	9	37.9	Yes

7	18-May-10	Male	high transmission	17-Aug-10	active follow	2185	36	No
8	18-May-10	Male	high transmission	21-Aug-10	passive follow	1012	38.5	Yes
9	18-May-10	Male	high transmission	8-Sep-10	passive follow	9	36.6	No
10	18-May-10	Male	high transmission	13-Sep-10	active follow		36	No
11	18-May-10	Male	high transmission	22-Sep-10	passive follow		37.2	Yes
12	18-May-10	Male	high transmission	13-Oct-10	active follow	1133	36.1	No
13	18-May-10	Male	high transmission	16-Oct-10	passive follow		38.5	Yes
14	18-May-10	Male	high transmission	25-Oct-10	passive follow	9	36.9	No
15	18-May-10	Male	high transmission	4-Nov-10	passive follow		37.7	Yes
16	18-May-10	Male	high transmission	9-Nov-10	active follow		36.1	No
17	18-May-10	Male	high transmission	14-Nov-10	passive follow		37.1	Yes
18	18-May-10	Male	high transmission	15-Nov-10	passive follow	9		
19	18-May-10	Male	high transmission	7-Dec-10	active follow	9	37.1	Yes
20	18-May-10	Male	high transmission	24-Dec-10	passive follow		38.4	Yes
21	18-May-10	Male	high transmission	3-Jan-11	active follow	9	36.1	No
22	18-May-10	Male	high transmission	21-Jan-11	passive follow	9	36.8	Yes
23	18-May-10	Male	high transmission	1-Feb-11	active follow	9	36	No
24	18-May-10	Male	high transmission	7-Feb-11	passive follow	9	35.6	Yes
25	18-May-10	Male	high transmission	15-Feb-11	passive follow	9	37	Yes
26	18-May-10	Male	high transmission	3-Mar-11	active follow	9	37.2	Yes
27	18-May-10	Male	high transmission	11-Mar-11	passive follow	12114	38.4	Yes
28	18-May-10	Male	high transmission	21-Mar-11	active follow	9	36.4	Yes
29	18-May-10	Male	high transmission	23-Apr-11	active follow			
30	18-May-10	Male	high transmission	6-May-11	passive follow	9	37.2	Yes
31	18-May-10	Male	high transmission	12-May-11	active follow	9	36.5	Yes
1	15-May-10	Female	high transmission	14-Jun-10	active follow	9	36.4	No
2	15-May-10	Female	high transmission	15-Jul-10	active follow	9	36.6	No
3	15-May-10	Female	high transmission	20-Jul-10	passive follow	9	36.8	No
4	15-May-10	Female	high transmission	13-Aug-10	passive follow	1264	36.7	No
5	15-May-10	Female	high transmission	19-Sep-10	active follow		36.8	No
6	15-May-10	Female	high transmission	25-Oct-10	active follow	9	39.3	Yes
7	15-May-10	Female	high transmission	9-Nov-10	passive follow	9485	38.9	Yes
8	15-May-10	Female	high transmission	3-Dec-10	active follow	9	36.2	No
9	15-May-10	Female	high transmission	15-Dec-10	passive follow	73101	36.8	No
10	15-May-10	Female	high transmission	9-Jan-11	active follow	9	36.6	No
11	15-May-10	Female	high transmission	17-Feb-11	active follow	174	36.2	No
12	15-May-10	Female	high transmission	23-Mar-11	active follow			
13	15-May-10	Female	high transmission	12-Apr-11	passive follow	9	36.7	No
14	15-May-10	Female	high transmission	26-Apr-11	active follow			
15	15-May-10	Female	high transmission	3-May-11	passive follow	83715	40.1	Yes
1	17-May-10	Female	high transmission	16-Jun-10	active follow	9	36.5	No
2	17-May-10	Female	high transmission	14-Jul-10	active follow	9	36.4	No
3	17-May-10	Female	high transmission	14-Aug-10	active follow	1291	36.2	No
4	17-May-10	Female	high transmission	17-Sep-10	active follow	3678	37.1	No
5	17-May-10	Female	high transmission	22-Oct-10	active follow	9	36.8	No
6	17-May-10	Female	high transmission	24-Nov-10	active follow	577	36.2	No
7	17-May-10	Female	high transmission	29-Nov-10	passive follow	865	36.2	No

8	17-May-10	Female	high transmission	21-Dec-10	active follow	9	36.1	No
9	17-May-10	Female	high transmission	18-Jan-11	active follow	9	36.6	No
10	17-May-10	Female	high transmission	15-Feb-11	active follow	18597	37.7	Yes
11	17-May-10	Female	high transmission	17-Feb-11	passive follow	16169	39	Yes
12	17-May-10	Female	high transmission	11-Mar-11	active follow	9	36.3	No
13	17-May-10	Female	high transmission	4-Apr-11	active follow	9	36.6	No
14	17-May-10	Female	high transmission	16-Apr-11	passive follow	67940	36.6	Yes
15	17-May-10	Female	high transmission	28-Apr-11	active follow	9	36	No
1	30-May-10	Female	high transmission	2-Jul-10	active follow	9	36.8	No
2	30-May-10	Female	high transmission	9-Aug-10	active follow	9	36.9	No
3	30-May-10	Female	high transmission	9-Sep-10	active follow	9	36.4	No
4	30-May-10	Female	high transmission	11-Oct-10	active follow		36.6	No
5	30-May-10	Female	high transmission	12-Nov-10	active follow	9	36.6	No
6	30-May-10	Female	high transmission	6-Dec-10	passive follow	144769	39.5	Yes
7	30-May-10	Female	high transmission	24-Dec-10	active follow	95	36.2	No
8	30-May-10	Female	high transmission	30-Jan-11	active follow	1128	36.6	No
9	30-May-10	Female	high transmission	6-Mar-11	active follow	786	36.3	No
10	30-May-10	Female	high transmission	4-Apr-11	active follow	19472	36.6	Yes
11	30-May-10	Female	high transmission	2-May-11	active follow	3354	37.6	Yes
1	30-May-10	Male	high transmission	7-Jul-10	active follow	9	36.8	No
2	30-May-10	Male	high transmission	6-Aug-10	active follow		36.6	No
3	30-May-10	Male	high transmission	10-Sep-10	active follow		35.5	No
4	30-May-10	Male	high transmission	18-Oct-10	active follow	9	36	No
5	30-May-10	Male	high transmission	19-Nov-10	active follow	9	36.4	No
6	30-May-10	Male	high transmission	2-Dec-10	passive follow	3419	38.9	Yes
7	30-May-10	Male	high transmission	23-Dec-10	active follow	9	38.3	Yes
8	30-May-10	Male	high transmission	22-Jan-11	active follow	9	36.3	No
9	30-May-10	Male	high transmission	26-Feb-11	active follow	9	36.7	No
10	30-May-10	Male	high transmission	10-Mar-11	passive follow	30388	36.7	Yes
11	30-May-10	Male	high transmission	23-Mar-11	active follow	9	36	No
12	30-May-10	Male	high transmission	12-Apr-11	passive follow	34821	37.6	Yes
13	30-May-10	Male	high transmission	21-Apr-11	active follow	32279	36.3	No
14	30-May-10	Male	high transmission	14-May-11	active follow	2320	36.4	No
1	2-Jun-10	Female	high transmission	30-Jun-10	active follow	9	37	No
2	2-Jun-10	Female	high transmission	6-Jul-10	passive follow	9		
3	2-Jun-10	Female	high transmission	29-Jul-10	active follow	9	36.4	No
4	2-Jun-10	Female	high transmission	6-Aug-10	passive follow		36.8	No
5	2-Jun-10	Female	high transmission	26-Aug-10	active follow	9	36	No
6	2-Jun-10	Female	high transmission	2-Oct-10	active follow		36	No
7	2-Jun-10	Female	high transmission	3-Nov-10	active follow	9	36.5	No
8	2-Jun-10	Female	high transmission	30-Nov-10	active follow	10450	35.5	No
9	2-Jun-10	Female	high transmission	28-Dec-10	active follow	5322	36.5	No
10	2-Jun-10	Female	high transmission	25-Jan-11	active follow	26877	36.3	No
11	2-Jun-10	Female	high transmission	3-Feb-11	passive follow	1359	35.5	No
12	2-Jun-10	Female	high transmission	24-Feb-11	active follow	9	36.4	No
13	2-Jun-10	Female	high transmission	7-Mar-11	passive follow	9	36.1	Yes
14	2-Jun-10	Female	high transmission	27-Mar-11	active follow	9	36.1	No

15	2-Jun-10	Female	high transmission	20-Apr-11	active follow	9	36.3	No
16	2-Jun-10	Female	high transmission	7-May-11	active follow	128322	38.6	Yes
1	2-Jun-10	Male	high transmission	1-Jul-10	active follow	9	36	No
2	2-Jun-10	Male	high transmission	28-Jul-10	active follow	9	36.6	No
3	2-Jun-10	Male	high transmission	2-Aug-10	passive follow	9	37.3	Yes
4	2-Jun-10	Male	high transmission	25-Aug-10	active follow	9	35.5	No
5	2-Jun-10	Male	high transmission	29-Sep-10	active follow		36	No
6	2-Jun-10	Male	high transmission	1-Nov-10	active follow	9	36.6	No
7	2-Jun-10	Male	high transmission	8-Dec-10	active follow	18175	36.5	No
8	2-Jun-10	Male	high transmission	12-Jan-11	active follow	9	36.5	No
9	2-Jun-10	Male	high transmission	15-Feb-11	active follow	9	36.7	No
10	2-Jun-10	Male	high transmission	15-Mar-11	active follow	9	36.3	No
11	2-Jun-10	Male	high transmission	25-Mar-11	passive follow	9	37.6	Yes
12	2-Jun-10	Male	high transmission	17-Apr-11	active follow	9	36.3	No
13	2-Jun-10	Male	high transmission	12-May-11	active follow	9	36	No
14	2-Jun-10	Male	high transmission	23-May-11	passive follow	256847	38.2	Yes
1	4-Jun-10	Male	high transmission	12-Jul-10	active follow	9	36.8	No
2	4-Jun-10	Male	high transmission	12-Jul-10	active follow	9	36.7	No
3	4-Jun-10	Male	high transmission	12-Jul-10	active follow			
4	4-Jun-10	Male	high transmission	24-Jul-10	passive follow	72001	39.7	Yes
5	4-Jun-10	Male	high transmission	24-Jul-10	passive follow	4919	38.1	Yes
6	4-Jun-10	Male	high transmission	18-Aug-10	active follow			
7	4-Jun-10	Male	high transmission	18-Aug-10	active follow		36.4	No
8	4-Jun-10	Male	high transmission	18-Aug-10	active follow		36.3	No
9	4-Jun-10	Male	high transmission	21-Sep-10	active follow			
10	4-Jun-10	Male	high transmission	21-Sep-10	active follow			
11	4-Jun-10	Male	high transmission	25-Sep-10	active follow		36.8	Yes
12	4-Jun-10	Male	high transmission	21-Oct-10	active follow			
13	4-Jun-10	Male	high transmission	25-Oct-10	active follow	4353	36.6	No
14	4-Jun-10	Male	high transmission	28-Oct-10	active follow			
15	4-Jun-10	Male	high transmission	20-Nov-10	active follow			
16	4-Jun-10	Male	high transmission	29-Nov-10	active follow	9	36.8	No
17	4-Jun-10	Male	high transmission	1-Dec-10	active follow			
18	4-Jun-10	Male	high transmission	20-Dec-10	active follow			
19	4-Jun-10	Male	high transmission	4-Jan-11	active follow			
20	4-Jun-10	Male	high transmission	4-Jan-11	active follow	9	36.2	No
21	4-Jun-10	Male	high transmission	20-Jan-11	active follow			
22	4-Jun-10	Male	high transmission	2-Feb-11	active follow			
23	4-Jun-10	Male	high transmission	10-Feb-11	active follow	9	38.2	Yes
24	4-Jun-10	Male	high transmission	26-Feb-11	active follow			
25	4-Jun-10	Male	high transmission	5-Mar-11	active follow			
26	4-Jun-10	Male	high transmission	13-Mar-11	active follow	9	36.3	No
27	4-Jun-10	Male	high transmission	31-Mar-11	active follow			
28	4-Jun-10	Male	high transmission	8-Apr-11	active follow			
29	4-Jun-10	Male	high transmission	12-Apr-11	active follow	9	36.7	No
30	4-Jun-10	Male	high transmission	1-May-11	active follow			
31	4-Jun-10	Male	high transmission	5-May-11	active follow			

32	4-Jun-10	Male	high transmission	15-May-11	active follow	9	36.2	No
33	4-Jun-10	Male	high transmission	24-May-11	active follow			
1	21-Dec-08	Female	low transmission	28-Jan-09	active follow	9	36.4	No
2	21-Dec-08	Female	low transmission	27-Feb-09	active follow	9	36.5	No
3	21-Dec-08	Female	low transmission	4-Apr-09	active follow	9	37.2	Yes
4	21-Dec-08	Female	low transmission	7-May-09	active follow	9	36.7	No
5	21-Dec-08	Female	low transmission	4-Jun-09	active follow	2158	36.2	No
6	21-Dec-08	Female	low transmission	7-Jul-09	active follow	6298	36.8	No
7	21-Dec-08	Female	low transmission	5-Aug-09	active follow	6679	36.5	No
8	21-Dec-08	Female	low transmission	28-Aug-09	passive follow	80818	39.5	Yes
9	21-Dec-08	Female	low transmission	9-Sep-09	active follow	9	36.6	No
10	21-Dec-08	Female	low transmission	26-Sep-09	passive follow	314492	37.8	Yes
11	21-Dec-08	Female	low transmission	16-Oct-09	active follow		36.5	No
12	21-Dec-08	Female	low transmission	25-Nov-09	active follow	4926	36.5	No
1	7-Jun-10	Male	high transmission	13-Jul-10	active follow	9	36.4	No
2	7-Jun-10	Male	high transmission	28-Jul-10	passive follow	9	37.1	Yes
3	7-Jun-10	Male	high transmission	18-Aug-10	active follow	9	36.2	No
4	7-Jun-10	Male	high transmission	23-Sep-10	active follow	9	36.3	No
5	7-Jun-10	Male	high transmission	19-Oct-10	active follow	9	37.1	Yes
6	7-Jun-10	Male	high transmission	17-Nov-10	active follow	28747	36.3	No
7	7-Jun-10	Male	high transmission	5-Dec-10	passive follow		38.5	Yes
8	7-Jun-10	Male	high transmission	14-Dec-10	active follow	9	36.1	No
9	7-Jun-10	Male	high transmission	12-Jan-11	active follow	9	36.2	No
10	7-Jun-10	Male	high transmission	17-Jan-11	passive follow	157	38.9	Yes
11	7-Jun-10	Male	high transmission	9-Feb-11	active follow		35.8	No
12	7-Jun-10	Male	high transmission	11-Feb-11	active follow	9		
13	7-Jun-10	Male	high transmission	4-Mar-11	active follow	9	36.2	No
14	7-Jun-10	Male	high transmission	8-Apr-11	active follow	9	36.1	No
15	7-Jun-10	Male	high transmission	10-Apr-11	passive follow	9	38	Yes
16	7-Jun-10	Male	high transmission	14-May-11	active follow			
1	19-Dec-08	Female	low transmission	13-Jan-09	passive follow		36.5	No
2	19-Dec-08	Female	low transmission	24-Jan-09	active follow	9	36.5	No
3	19-Dec-08	Female	low transmission	23-Feb-09	active follow	9	36	No
4	19-Dec-08	Female	low transmission	1-Apr-09	active follow	9	36	No
5	19-Dec-08	Female	low transmission	11-Apr-09	passive follow	9	36	No
6	19-Dec-08	Female	low transmission	8-May-09	active follow	16200	36.3	No
7	19-Dec-08	Female	low transmission	20-Jun-09	active follow	982	36	No
8	19-Dec-08	Female	low transmission	15-Jul-09	passive follow	109356	37	Yes
9	19-Dec-08	Female	low transmission	17-Jul-09	passive follow		37	Yes
10	19-Dec-08	Female	low transmission	19-Aug-09	passive follow	999999	37.2	Yes
11	19-Dec-08	Female	low transmission	25-Sep-09	active follow		36.3	No
12	19-Dec-08	Female	low transmission	31-Oct-09	active follow	9	36.4	No
13	19-Dec-08	Female	low transmission	10-Nov-09	passive follow	9	36.4	Yes
14	19-Dec-08	Female	low transmission	3-Dec-09	active follow	9	36	No
1	6-Jun-10	Male	high transmission	6-Jul-10	active follow	9	37	No
2	6-Jun-10	Male	high transmission	5-Aug-10	active follow	9	35.7	No
3	6-Jun-10	Male	high transmission	3-Sep-10	active follow		36	Yes

4	6-Jun-10	Male	high transmission	4-Sep-10	active follow	9	35.7	Yes
5	6-Jun-10	Male	high transmission	12-Oct-10	active follow		35.7	No
6	6-Jun-10	Male	high transmission	21-Oct-10	passive follow	5723	35.7	Yes
7	6-Jun-10	Male	high transmission	9-Nov-10	active follow	2326	36	No
8	6-Jun-10	Male	high transmission	24-Nov-10	passive follow	1284	36	Yes
9	6-Jun-10	Male	high transmission	28-Nov-10	passive follow	9	35.7	Yes
10	6-Jun-10	Male	high transmission	10-Dec-10	active follow	9	35.7	No
11	6-Jun-10	Male	high transmission	30-Dec-10	passive follow	9	36.5	Yes
12	6-Jun-10	Male	high transmission	11-Jan-11	active follow	9	36	No
13	6-Jun-10	Male	high transmission	31-Jan-11	passive follow	9	36.4	Yes
14	6-Jun-10	Male	high transmission	11-Feb-11	active follow		36.2	No
15	6-Jun-10	Male	high transmission	14-Mar-11	active follow		36.2	No
16	6-Jun-10	Male	high transmission	7-Apr-11	active follow		36.2	No
17	6-Jun-10	Male	high transmission	2-May-11	active follow		36	No
1	9-Jun-10	Female	high transmission	8-Jul-10	active follow	9	36.5	No
2	9-Jun-10	Female	high transmission	2-Aug-10	passive follow	9	36.3	Yes
3	9-Jun-10	Female	high transmission	13-Aug-10	active follow	9	36.3	No
4	9-Jun-10	Female	high transmission	17-Sep-10	active follow	9	35.5	No
5	9-Jun-10	Female	high transmission	22-Oct-10	active follow	9	36	No
6	9-Jun-10	Female	high transmission	24-Nov-10	active follow	16797	37	No
7	9-Jun-10	Female	high transmission	23-Dec-10	active follow	6798	36.3	No
8	9-Jun-10	Female	high transmission	21-Jan-11	active follow	25481	35.8	No
9	9-Jun-10	Female	high transmission	11-Feb-11	passive follow	4554	38.8	Yes
10	9-Jun-10	Female	high transmission	21-Feb-11	active follow	9	35.7	No
11	9-Jun-10	Female	high transmission	17-Mar-11	active follow	9	36.2	No
12	9-Jun-10	Female	high transmission	2-Apr-11	passive follow	9	37.6	Yes
13	9-Jun-10	Female	high transmission	14-Apr-11	active follow		36.4	No
14	9-Jun-10	Female	high transmission	7-May-11	active follow	9	35.1	No
1	15-Jun-10	Female	high transmission	14-Jul-10	active follow	43	36.7	No
2	15-Jun-10	Female	high transmission	11-Aug-10	active follow		36.7	No
3	15-Jun-10	Female	high transmission	7-Sep-10	active follow		36.7	Yes
4	15-Jun-10	Female	high transmission	4-Oct-10	active follow	9	36	No
5	15-Jun-10	Female	high transmission	28-Oct-10	active follow	8228	36.4	No
6	15-Jun-10	Female	high transmission	25-Nov-10	active follow	9	36.7	No
7	15-Jun-10	Female	high transmission	17-Dec-10	active follow	81156	37.7	Yes
8	15-Jun-10	Female	high transmission	19-Jan-11	active follow		36.2	No
9	15-Jun-10	Female	high transmission	12-Feb-11	active follow	9	36.1	No
10	15-Jun-10	Female	high transmission	10-Mar-11	active follow	330901	39.8	Yes
11	15-Jun-10	Female	high transmission	5-Apr-11	active follow	1942	36.5	No
12	15-Jun-10	Female	high transmission	9-Apr-11	passive follow	155368	39	Yes
13	15-Jun-10	Female	high transmission	29-Apr-11	passive follow	9	36.4	Yes
14	15-Jun-10	Female	high transmission	4-May-11	active follow	9	36	No
1	20-Dec-08	Female	low transmission	19-Jan-09	active follow	9	36.8	No
2	20-Dec-08	Female	low transmission	18-Feb-09	active follow	9	37	No
3	20-Dec-08	Female	low transmission	21-Mar-09	active follow	9	36	No
4	20-Dec-08	Female	low transmission	22-Apr-09	active follow	9	36.8	No
5	20-Dec-08	Female	low transmission	26-May-09	active follow	2146	36.3	No

6	20-Dec-08	Female	low transmission	26-Jun-09	active follow	4434	36.5	No
7	20-Dec-08	Female	low transmission	29-Jul-09	active follow	210	36.2	No
8	20-Dec-08	Female	low transmission	10-Aug-09	passive follow	9		
9	20-Dec-08	Female	low transmission	26-Aug-09	active follow	9	36.9	No
10	20-Dec-08	Female	low transmission	26-Sep-09	active follow	15246	38	No
11	20-Dec-08	Female	low transmission	8-Oct-09	passive follow		36.4	Yes
12	20-Dec-08	Female	low transmission	14-Oct-09	passive follow	5297		
13	20-Dec-08	Female	low transmission	16-Oct-09	passive follow		36	Yes
14	20-Dec-08	Female	low transmission	31-Oct-09	active follow	2603	36.5	No
15	20-Dec-08	Female	low transmission	5-Dec-09	active follow		36.5	No
1	16-Jun-10	Male	high transmission	13-Jul-10	active follow	9	36.5	No
2	16-Jun-10	Male	high transmission	27-Jul-10	passive follow	9	36.7	Yes
3	16-Jun-10	Male	high transmission	11-Aug-10	active follow		36.7	Yes
4	16-Jun-10	Male	high transmission	7-Sep-10	active follow	9	36.4	No
5	16-Jun-10	Male	high transmission	10-Oct-10	active follow	9	36.3	No
6	16-Jun-10	Male	high transmission	18-Oct-10	passive follow	9	36.1	Yes
7	16-Jun-10	Male	high transmission	16-Nov-10	active follow			
8	16-Jun-10	Male	high transmission	26-Nov-10	passive follow	3505		
9	16-Jun-10	Male	high transmission	15-Dec-10	active follow	9	37.8	Yes
10	16-Jun-10	Male	high transmission	14-Jan-11	active follow	11434	36.7	No
11	16-Jun-10	Male	high transmission	28-Jan-11	passive follow	945	37	Yes
12	16-Jun-10	Male	high transmission	7-Feb-11	active follow	3025	35.6	No
13	16-Jun-10	Male	high transmission	12-Mar-11	active follow	708	35.4	No
14	16-Jun-10	Male	high transmission	16-Mar-11	passive follow	3866	36.8	Yes
15	16-Jun-10	Male	high transmission	9-Apr-11	active follow	9	36.9	No
16	16-Jun-10	Male	high transmission	16-May-11	active follow			
1	20-Jun-10	Female	high transmission	27-Jul-10	active follow	9	36.7	No
2	20-Jun-10	Female	high transmission	26-Aug-10	active follow	9	36.5	No
3	20-Jun-10	Female	high transmission	8-Sep-10	passive follow	9	36.7	Yes
4	20-Jun-10	Female	high transmission	21-Sep-10	passive follow	9	36.7	Yes
5	20-Jun-10	Female	high transmission	25-Sep-10	active follow		36.4	No
6	20-Jun-10	Female	high transmission	27-Oct-10	active follow	9	36.9	Yes
7	20-Jun-10	Female	high transmission	17-Nov-10	active follow	9	38.9	Yes
8	20-Jun-10	Female	high transmission	4-Dec-10	passive follow	133488	38.9	Yes
9	20-Jun-10	Female	high transmission	13-Dec-10	active follow	9	36.1	No
10	20-Jun-10	Female	high transmission	10-Jan-11	active follow	22162	36.4	No
11	20-Jun-10	Female	high transmission	7-Feb-11	active follow	653	36.6	No
12	20-Jun-10	Female	high transmission	12-Feb-11	passive follow	881	37	Yes
13	20-Jun-10	Female	high transmission	5-Mar-11	active follow	9	36.1	No
14	20-Jun-10	Female	high transmission	16-Mar-11	passive follow	9	35.9	Yes
15	20-Jun-10	Female	high transmission	8-Apr-11	active follow	43	36.4	No
16	20-Jun-10	Female	high transmission	15-Apr-11	passive follow	303374	37.6	Yes
17	20-Jun-10	Female	high transmission	3-May-11	active follow	9	36.4	No
18	20-Jun-10	Female	high transmission	15-May-11	passive follow	130515	37.1	Yes
1	12-Jun-10	Male	high transmission	25-Jun-10	passive follow		37.7	Yes
2	12-Jun-10	Male	high transmission	15-Jul-10	active follow	9	36.5	No
3	12-Jun-10	Male	high transmission	12-Aug-10	active follow	9	36.1	No

4	12-Jun-10	Male	high transmission	18-Aug-10	passive follow	9	36.8	No
5	12-Jun-10	Male	high transmission	13-Sep-10	active follow	9	36.5	No
6	12-Jun-10	Male	high transmission	15-Oct-10	active follow		36.4	No
7	12-Jun-10	Male	high transmission	15-Nov-10	active follow	9	36.6	Yes
8	12-Jun-10	Male	high transmission	14-Dec-10	active follow	9	36.5	No
9	12-Jun-10	Male	high transmission	4-Jan-11	active follow	9	39.1	Yes
10	12-Jun-10	Male	high transmission	1-Feb-11	active follow	9	36.3	No
11	12-Jun-10	Male	high transmission	19-Feb-11	passive follow	9	36.2	Yes
12	12-Jun-10	Male	high transmission	3-Mar-11	active follow	38594	37	No
13	12-Jun-10	Male	high transmission	14-Mar-11	passive follow		37.2	Yes
14	12-Jun-10	Male	high transmission	24-Mar-11	active follow	33129	39.4	Yes
15	12-Jun-10	Male	high transmission	14-Apr-11	active follow	9	36.8	No
16	12-Jun-10	Male	high transmission	4-May-11	active follow	9	36.8	Yes
1	19-Jun-10	Female	high transmission	26-Jul-10	active follow			
2	19-Jun-10	Female	high transmission	25-Aug-10	active follow			
3	19-Jun-10	Female	high transmission	24-Sep-10	active follow			
4	19-Jun-10	Female	high transmission	27-Oct-10	active follow	9	36.3	No
5	19-Jun-10	Female	high transmission	25-Nov-10	active follow	9	36.6	No
6	19-Jun-10	Female	high transmission	13-Dec-10	passive follow	583	37.1	Yes
7	19-Jun-10	Female	high transmission	30-Dec-10	active follow	2131	36.8	Yes
8	19-Jun-10	Female	high transmission	25-Jan-11	active follow	633	36	No
9	19-Jun-10	Female	high transmission	8-Feb-11	passive follow	96803	37.2	Yes
10	19-Jun-10	Female	high transmission	24-Feb-11	active follow	9	36.6	No
11	19-Jun-10	Female	high transmission	7-Mar-11	passive follow	9	36.5	Yes
12	19-Jun-10	Female	high transmission	21-Mar-11	active follow	9	36.6	No
13	19-Jun-10	Female	high transmission	19-Apr-11	active follow	9	36.5	No
14	19-Jun-10	Female	high transmission	14-May-11	active follow	9	36.1	No
1	16-Jun-10	Female	high transmission	16-Jul-10	active follow	9	36.4	No
2	16-Jun-10	Female	high transmission	18-Aug-10	active follow	271	36.8	No
3	16-Jun-10	Female	high transmission	19-Sep-10	active follow		36.2	No
4	16-Jun-10	Female	high transmission	16-Oct-10	passive follow	29462	35.8	Yes
5	16-Jun-10	Female	high transmission	22-Oct-10	active follow	9	36.1	No
6	16-Jun-10	Female	high transmission	26-Nov-10	active follow	9	36.5	No
7	16-Jun-10	Female	high transmission	8-Dec-10	passive follow	4742	36.9	Yes
8	16-Jun-10	Female	high transmission	28-Dec-10	active follow	9	36	No
9	16-Jun-10	Female	high transmission	14-Jan-11	passive follow	9	36.6	No
10	16-Jun-10	Female	high transmission	26-Jan-11	passive follow		37.2	Yes
11	16-Jun-10	Female	high transmission	27-Jan-11	active follow	9	36.7	No
12	16-Jun-10	Female	high transmission	21-Feb-11	active follow	9	36	Yes
13	16-Jun-10	Female	high transmission	16-Mar-11	active follow	9	36	No
14	16-Jun-10	Female	high transmission	14-Apr-11	active follow	9	36	No
15	16-Jun-10	Female	high transmission	25-Apr-11	passive follow	15372	37.7	Yes
16	16-Jun-10	Female	high transmission	7-May-11	active follow	9	36.4	No
1	21-Jun-10	Female	high transmission	24-Jul-10	active follow	9	36.2	No
2	21-Jun-10	Female	high transmission	28-Aug-10	active follow	9	36.5	No
3	21-Jun-10	Female	high transmission	2-Oct-10	active follow	2178	35.5	No
4	21-Jun-10	Female	high transmission	5-Oct-10	passive follow	9	36.4	No

5	21-Jun-10	Female	high transmission	8-Nov-10	active follow			
6	21-Jun-10	Female	high transmission	8-Dec-10	active follow			
7	21-Jun-10	Female	high transmission	21-Dec-10	passive follow	11329	36.3	Yes
8	21-Jun-10	Female	high transmission	7-Jan-11	active follow			
9	21-Jun-10	Female	high transmission	13-Feb-11	active follow	9	36	No
10	21-Jun-10	Female	high transmission	10-Mar-11	active follow	9	36.5	No
11	21-Jun-10	Female	high transmission	25-Mar-11	passive follow	321334	38.8	Yes
12	21-Jun-10	Female	high transmission	8-Apr-11	active follow	9	36.4	No
13	21-Jun-10	Female	high transmission	4-May-11	active follow	9	36.4	No
14	21-Jun-10	Female	high transmission	10-May-11	passive follow		38	Yes
1	27-Jun-10	Female	high transmission	29-Jul-10	active follow	9	36.9	No
2	27-Jun-10	Female	high transmission	24-Aug-10	active follow	9	37	No
3	27-Jun-10	Female	high transmission	23-Sep-10	active follow	2697	35.8	No
4	27-Jun-10	Female	high transmission	11-Oct-10	passive follow	9	36.6	Yes
5	27-Jun-10	Female	high transmission	22-Oct-10	active follow	9	36.2	No
6	27-Jun-10	Female	high transmission	27-Nov-10	active follow	12762	38.5	Yes
7	27-Jun-10	Female	high transmission	15-Dec-10	passive follow	9	37	No
8	27-Jun-10	Female	high transmission	31-Dec-10	active follow	9	36.2	Yes
9	27-Jun-10	Female	high transmission	31-Jan-11	active follow	9	38.3	Yes
10	27-Jun-10	Female	high transmission	9-Mar-11	active follow			
11	27-Jun-10	Female	high transmission	16-Mar-11	passive follow		37.8	Yes
12	27-Jun-10	Female	high transmission	29-Mar-11	passive follow	9	37	Yes
13	27-Jun-10	Female	high transmission	7-Apr-11	active follow	36899	36.2	No
14	27-Jun-10	Female	high transmission	3-May-11	active follow	9	36	No
1	28-Jun-10	Female	high transmission	5-Aug-10	active follow	645	36.5	No
2	28-Jun-10	Female	high transmission	3-Sep-10	active follow	2071	36.6	No
3	28-Jun-10	Female	high transmission	9-Sep-10	passive follow	14532	38.6	Yes
4	28-Jun-10	Female	high transmission	15-Oct-10	active follow	9	36.8	No
5	28-Jun-10	Female	high transmission	30-Nov-10	active follow		36.6	No
6	28-Jun-10	Female	high transmission	2-Jan-11	active follow	33687	36.8	No
7	28-Jun-10	Female	high transmission	2-Feb-11	active follow	9	36	No
8	28-Jun-10	Female	high transmission	4-Feb-11	passive follow	9	37.5	Yes
9	28-Jun-10	Female	high transmission	3-Mar-11	active follow	9	36.5	No
10	28-Jun-10	Female	high transmission	5-Apr-11	active follow	91772	37.1	No
11	28-Jun-10	Female	high transmission	7-May-11	active follow	9	36.7	No
1	25-Jun-10	Male	high transmission	2-Aug-10	active follow	9	35.8	No
2	25-Jun-10	Male	high transmission	7-Sep-10	active follow	4680	36.6	No
3	25-Jun-10	Male	high transmission	22-Sep-10	passive follow	19775	37.8	Yes
4	25-Jun-10	Male	high transmission	8-Oct-10	active follow	9	36.9	No
5	25-Jun-10	Male	high transmission	11-Nov-10	active follow	9	37.6	Yes
6	25-Jun-10	Male	high transmission	9-Dec-10	active follow	9	37	No
7	25-Jun-10	Male	high transmission	12-Jan-11	active follow	9	36.7	No
8	25-Jun-10	Male	high transmission	14-Feb-11	active follow	9	36.9	No
9	25-Jun-10	Male	high transmission	25-Feb-11	passive follow	154858	37.9	Yes
10	25-Jun-10	Male	high transmission	18-Mar-11	active follow	9	37	No
11	25-Jun-10	Male	high transmission	29-Mar-11	passive follow	59103	38.3	Yes
12	25-Jun-10	Male	high transmission	17-Apr-11	active follow	9	36.4	No

13	25-Jun-10	Male	high transmission	11-May-11	active follow	9	36	No
14	25-Jun-10	Male	high transmission	17-May-11	passive follow	48505	38.4	Yes
1	20-Jun-10	Male	high transmission	23-Jul-10	active follow	9	36.4	No
2	20-Jun-10	Male	high transmission	26-Aug-10	active follow	9	36.2	No
3	20-Jun-10	Male	high transmission	27-Sep-10	active follow	9	36.5	No
4	20-Jun-10	Male	high transmission	19-Oct-10	active follow	9	36.6	Yes
5	20-Jun-10	Male	high transmission	23-Nov-10	active follow	53	36	No
6	20-Jun-10	Male	high transmission	9-Dec-10	passive follow	607		
7	20-Jun-10	Male	high transmission	20-Dec-10	active follow		37.7	Yes
8	20-Jun-10	Male	high transmission	18-Jan-11	active follow	2105	36.6	No
9	20-Jun-10	Male	high transmission	22-Jan-11	passive follow	3885	37.1	Yes
10	20-Jun-10	Male	high transmission	15-Feb-11	active follow	9	36.6	No
11	20-Jun-10	Male	high transmission	8-Mar-11	active follow	9	36.5	No
12	20-Jun-10	Male	high transmission	19-Mar-11	passive follow	9	38.3	Yes
13	20-Jun-10	Male	high transmission	12-Apr-11	active follow		36.6	No
14	20-Jun-10	Male	high transmission	5-May-11	active follow	9	36.3	No
15	20-Jun-10	Male	high transmission	17-May-11	passive follow	9	36.3	No
1	5-Jul-10	Male	high transmission	6-Aug-10	active follow	9	36.7	No
2	5-Jul-10	Male	high transmission	5-Sep-10	passive follow	9	37	Yes
3	5-Jul-10	Male	high transmission	9-Sep-10	active follow	9	36.7	No
4	5-Jul-10	Male	high transmission	7-Oct-10	active follow	9	36.6	No
5	5-Jul-10	Male	high transmission	3-Nov-10	active follow	9	36	No
6	5-Jul-10	Male	high transmission	1-Dec-10	active follow	9	35.9	No
7	5-Jul-10	Male	high transmission	30-Dec-10	active follow	553	37.1	No
8	5-Jul-10	Male	high transmission	11-Jan-11	passive follow	29286		
9	5-Jul-10	Male	high transmission	12-Jan-11	passive follow		37.9	Yes
10	5-Jul-10	Male	high transmission	27-Jan-11	active follow	9	36.2	No
11	5-Jul-10	Male	high transmission	6-Feb-11	passive follow	9	37.3	Yes
12	5-Jul-10	Male	high transmission	24-Feb-11	active follow	9	36.1	No
13	5-Jul-10	Male	high transmission	22-Mar-11	active follow	9	35.9	No
14	5-Jul-10	Male	high transmission	14-Apr-11	active follow	9	36.2	No
15	5-Jul-10	Male	high transmission	8-May-11	active follow	9	36.2	No
1	11-Jul-10	Male	high transmission	10-Aug-10	active follow	9	36	No
2	11-Jul-10	Male	high transmission	7-Sep-10	active follow	350	36.8	No
3	11-Jul-10	Male	high transmission	11-Oct-10	active follow	9	36.6	No
4	11-Jul-10	Male	high transmission	7-Nov-10	active follow	9	35.7	Yes
5	11-Jul-10	Male	high transmission	7-Dec-10	active follow	9	35.7	No
6	11-Jul-10	Male	high transmission	4-Jan-11	active follow	9	36	No
7	11-Jul-10	Male	high transmission	12-Jan-11	passive follow	168640	38	Yes
8	11-Jul-10	Male	high transmission	2-Feb-11	active follow	9	36.6	No
9	11-Jul-10	Male	high transmission	3-Mar-11	active follow	9	36.2	No
10	11-Jul-10	Male	high transmission	30-Mar-11	active follow	9	36.6	Yes
11	11-Jul-10	Male	high transmission	24-Apr-11	active follow	9	36	No
12	11-Jul-10	Male	high transmission	30-Apr-11	passive follow	249185	38.4	No
13	11-Jul-10	Male	high transmission	21-May-11	active follow	9	36	No
1	10-Jul-10	Female	high transmission	14-Aug-10	active follow	9	36.5	No
2	10-Jul-10	Female	high transmission	26-Sep-10	active follow	9	36.8	No

3	10-Jul-10	Female	high transmission	27-Sep-10	passive follow	9	36.8	Yes
4	10-Jul-10	Female	high transmission	2-Nov-10	active follow	9	36.3	No
5	10-Jul-10	Female	high transmission	19-Nov-10	passive follow	9	36.9	Yes
6	10-Jul-10	Female	high transmission	2-Dec-10	active follow	9	36.7	No
7	10-Jul-10	Female	high transmission	9-Jan-11	active follow	1340	36.4	No
8	10-Jul-10	Female	high transmission	26-Jan-11	passive follow	50264	38.9	Yes
9	10-Jul-10	Female	high transmission	14-Feb-11	active follow	2456	36.7	No
10	10-Jul-10	Female	high transmission	26-Feb-11	passive follow	9313	36.8	No
11	10-Jul-10	Female	high transmission	23-Mar-11	active follow	9	36.6	No
12	10-Jul-10	Female	high transmission	1-Apr-11	passive follow	9	37	Yes
13	10-Jul-10	Female	high transmission	17-Apr-11	active follow	9	36	No
14	10-Jul-10	Female	high transmission	10-May-11	passive follow		37.5	Yes
1	7-Jul-10	Male	high transmission	5-Aug-10	active follow	9	36.1	No
2	7-Jul-10	Male	high transmission	11-Sep-10	active follow			
3	7-Jul-10	Male	high transmission	18-Oct-10	active follow	9	36.5	Yes
4	7-Jul-10	Male	high transmission	20-Nov-10	active follow	9	36.2	Yes
5	7-Jul-10	Male	high transmission	8-Dec-10	passive follow	9	37.8	Yes
6	7-Jul-10	Male	high transmission	21-Dec-10	active follow	454	36.8	No
7	7-Jul-10	Male	high transmission	19-Jan-11	active follow	7360	36	Yes
8	7-Jul-10	Male	high transmission	9-Feb-11	passive follow	9	36.7	No
9	7-Jul-10	Male	high transmission	16-Feb-11	active follow	9	36.5	No
10	7-Jul-10	Male	high transmission	12-Mar-11	active follow	9	36	No
11	7-Jul-10	Male	high transmission	16-Mar-11	passive follow		37.1	Yes
12	7-Jul-10	Male	high transmission	6-Apr-11	active follow	9	36.6	No
13	7-Jul-10	Male	high transmission	20-Apr-11	passive follow	1379	39.8	Yes
14	7-Jul-10	Male	high transmission	3-May-11	active follow	9	36.5	No
1	14-Jul-10	Female	high transmission	27-Aug-10	active follow	9	36.7	No
2	14-Jul-10	Female	high transmission	14-Sep-10	passive follow	9	36.3	No
3	14-Jul-10	Female	high transmission	10-Oct-10	active follow	9	36.7	No
4	14-Jul-10	Female	high transmission	9-Nov-10	active follow	6363	36.9	No
5	14-Jul-10	Female	high transmission	10-Dec-10	active follow	9	36.6	No
6	14-Jul-10	Female	high transmission	21-Jan-11	active follow	399	37.3	Yes
7	14-Jul-10	Female	high transmission	8-Feb-11	active follow	9	36.3	No
8	14-Jul-10	Female	high transmission	14-Mar-11	active follow			
9	14-Jul-10	Female	high transmission	17-Apr-11	active follow			
10	14-Jul-10	Female	high transmission	13-May-11	active follow	126770	36.1	No
11	14-Jul-10	Female	high transmission	17-May-11	passive follow	585571	37.6	Yes
1	16-Jul-10	Male	high transmission	27-Aug-10	active follow	9	36.5	No
2	16-Jul-10	Male	high transmission	14-Sep-10	passive follow	9	37.6	Yes
3	16-Jul-10	Male	high transmission	8-Oct-10	active follow	9	36.2	No
4	16-Jul-10	Male	high transmission	12-Nov-10	active follow	9	36.6	No
5	16-Jul-10	Male	high transmission	19-Dec-10	active follow	61	36.6	No
6	16-Jul-10	Male	high transmission	18-Jan-11	active follow	5022	36.2	No
7	16-Jul-10	Male	high transmission	15-Feb-11	active follow	9	36.1	No
8	16-Jul-10	Male	high transmission	23-Feb-11	passive follow	48606	36.8	Yes
9	16-Jul-10	Male	high transmission	1-Mar-11	passive follow		36.4	No
10	16-Jul-10	Male	high transmission	18-Mar-11	active follow	9	36.8	No

11	16-Jul-10	Male	high transmission	18-Apr-11	active follow	9	36.2	No
12	16-Jul-10	Male	high transmission	11-May-11	active follow	9	36.2	No
1	15-Jul-10	Female	high transmission	11-Aug-10	active follow	9	36.3	No
2	15-Jul-10	Female	high transmission	31-Aug-10	passive follow	2590	37	Yes
3	15-Jul-10	Female	high transmission	9-Sep-10	active follow	9	37	Yes
4	15-Jul-10	Female	high transmission	7-Oct-10	active follow	712	36.8	No
5	15-Jul-10	Female	high transmission	4-Nov-10	active follow	1190	36.5	No
6	15-Jul-10	Female	high transmission	23-Nov-10	passive follow	985		
7	15-Jul-10	Female	high transmission	24-Nov-10	passive follow		36.3	Yes
8	15-Jul-10	Female	high transmission	2-Dec-10	active follow	9	36.2	No
9	15-Jul-10	Female	high transmission	21-Dec-10	passive follow	9	36.2	Yes
10	15-Jul-10	Female	high transmission	30-Dec-10	active follow	9	35.8	No
11	15-Jul-10	Female	high transmission	30-Jan-11	active follow	9	36	No
12	15-Jul-10	Female	high transmission	9-Mar-11	active follow	9	36.5	No
13	15-Jul-10	Female	high transmission	10-Mar-11	passive follow		36.5	No
14	15-Jul-10	Female	high transmission	6-Apr-11	active follow		36.7	No
15	15-Jul-10	Female	high transmission	12-Apr-11	passive follow	122479	38.5	Yes
16	15-Jul-10	Female	high transmission	4-May-11	active follow	23279	37	No
1	15-Jul-10	Male	high transmission	2-Sep-10	active follow			
2	15-Jul-10	Male	high transmission	11-Oct-10	active follow	9	36	No
3	15-Jul-10	Male	high transmission	13-Nov-10	active follow	1308	36.6	No
4	15-Jul-10	Male	high transmission	25-Nov-10	passive follow	9	36.9	Yes
5	15-Jul-10	Male	high transmission	20-Dec-10	active follow	9	37.3	Yes
6	15-Jul-10	Male	high transmission	19-Jan-11	active follow		36.4	No
7	15-Jul-10	Male	high transmission	15-Feb-11	active follow	9	36.4	No
8	15-Jul-10	Male	high transmission	14-Mar-11	active follow	9	36.1	No
9	15-Jul-10	Male	high transmission	26-Mar-11	passive follow	9	37	Yes
10	15-Jul-10	Male	high transmission	11-Apr-11	active follow	9	36.3	No
11	15-Jul-10	Male	high transmission	28-Apr-11	passive follow	273560	38.7	Yes
12	15-Jul-10	Male	high transmission	7-May-11	active follow	9	36	No
1	17-Dec-08	Female	low transmission	22-Jan-09	active follow	9	36.2	No
2	17-Dec-08	Female	low transmission	21-Feb-09	active follow	9	36	No
3	17-Dec-08	Female	low transmission	24-Mar-09	active follow	9	36.3	No
4	17-Dec-08	Female	low transmission	24-Apr-09	active follow	9	36.1	No
5	17-Dec-08	Female	low transmission	29-May-09	active follow	49361	38.4	Yes
6	17-Dec-08	Female	low transmission	4-Jul-09	active follow	9	36.5	No
7	17-Dec-08	Female	low transmission	7-Aug-09	active follow	9	36	No
8	17-Dec-08	Female	low transmission	10-Sep-09	active follow	9	36.1	No
9	17-Dec-08	Female	low transmission	17-Sep-09	passive follow	1459	39.8	Yes
10	17-Dec-08	Female	low transmission	7-Oct-09	active follow		36.5	No
11	17-Dec-08	Female	low transmission	5-Nov-09	active follow	20926	36.2	No
12	17-Dec-08	Female	low transmission	5-Dec-09	active follow	5978	36.5	No
1	27-Aug-10	Male	high transmission	28-Sep-10	active follow	9	36.5	No
2	27-Aug-10	Male	high transmission	27-Oct-10	active follow	3822	36.1	No
3	27-Aug-10	Male	high transmission	25-Nov-10	active follow	9	35.1	No
4	27-Aug-10	Male	high transmission	21-Dec-10	active follow	9	35.6	No
5	27-Aug-10	Male	high transmission	12-Jan-11	passive follow	9	36.5	No

6	27-Aug-10	Male	high transmission	18-Jan-11	active follow	9	36.3	No
7	27-Aug-10	Male	high transmission	15-Feb-11	active follow	9	37.6	Yes
8	27-Aug-10	Male	high transmission	11-Mar-11	active follow	129158	37.5	Yes
9	27-Aug-10	Male	high transmission	3-Apr-11	active follow	9	36.4	No
10	27-Aug-10	Male	high transmission	4-Apr-11	passive follow	9	37.8	Yes
11	27-Aug-10	Male	high transmission	1-May-11	active follow	9	36.3	Yes
12	27-Aug-10	Male	high transmission	24-May-11	active follow	9	36.3	No
1	26-Aug-10	Male	high transmission	24-Sep-10	active follow	9	36.9	No
2	26-Aug-10	Male	high transmission	26-Oct-10	active follow	14596	36	No
3	26-Aug-10	Male	high transmission	3-Dec-10	active follow	933	36.7	No
4	26-Aug-10	Male	high transmission	11-Jan-11	active follow	29286	36.2	Yes
5	26-Aug-10	Male	high transmission	11-Feb-11	active follow	9	36.2	No
6	26-Aug-10	Male	high transmission	8-Mar-11	active follow	9	36.9	No
7	26-Aug-10	Male	high transmission	31-Mar-11	passive follow		36.8	Yes
8	26-Aug-10	Male	high transmission	12-Apr-11	active follow		36.5	No
9	26-Aug-10	Male	high transmission	18-Apr-11	passive follow	9	36.8	No
10	26-Aug-10	Male	high transmission	10-May-11	active follow	9	36.8	No
1	5-Sep-10	Female	high transmission	29-Sep-10	passive follow	9	36.6	No
2	5-Sep-10	Female	high transmission	5-Oct-10	active follow	9	36.1	No
3	5-Sep-10	Female	high transmission	2-Nov-10	active follow	9	36.1	No
4	5-Sep-10	Female	high transmission	30-Nov-10	active follow	2890	36.1	No
5	5-Sep-10	Female	high transmission	8-Dec-10	passive follow	3061	36.9	Yes
6	5-Sep-10	Female	high transmission	24-Dec-10	passive follow	9	36.7	Yes
7	5-Sep-10	Female	high transmission	27-Dec-10	active follow	9	36.1	No
8	5-Sep-10	Female	high transmission	26-Jan-11	active follow	9	36	No
9	5-Sep-10	Female	high transmission	4-Feb-11	passive follow	9	36	Yes
10	5-Sep-10	Female	high transmission	22-Feb-11	active follow	9	36.6	No
11	5-Sep-10	Female	high transmission	18-Mar-11	active follow	9	36	No
12	5-Sep-10	Female	high transmission	25-Mar-11	passive follow	9	36.5	No
13	5-Sep-10	Female	high transmission	6-Apr-11	active follow	9	36.6	No
14	5-Sep-10	Female	high transmission	7-Apr-11	active follow		36.6	No
15	5-Sep-10	Female	high transmission	12-Apr-11	passive follow	9	37.6	Yes
16	5-Sep-10	Female	high transmission	23-Apr-11	active follow	9	39.4	Yes
17	5-Sep-10	Female	high transmission	17-May-11	active follow	9	36	No
1	14-Sep-10	Male	high transmission	12-Oct-10	active follow	9	36.7	No
2	14-Sep-10	Male	high transmission	9-Nov-10	active follow	9	36.5	No
3	14-Sep-10	Male	high transmission	15-Nov-10	passive follow	9	36.8	Yes
4	14-Sep-10	Male	high transmission	8-Dec-10	active follow	10815	36.7	No
5	14-Sep-10	Male	high transmission	3-Jan-11	active follow	6299	36.7	No
6	14-Sep-10	Male	high transmission	3-Feb-11	active follow	4088	36.4	No
7	14-Sep-10	Male	high transmission	5-Mar-11	active follow	6054	36.2	No
8	14-Sep-10	Male	high transmission	9-Apr-11	active follow	5384	36.4	No
9	14-Sep-10	Male	high transmission	5-May-11	passive follow	1321	37.3	Yes
1	10-Oct-10	Female	high transmission	14-Nov-10	active follow	9	36.9	No
2	10-Oct-10	Female	high transmission	19-Dec-10	active follow	9	36.2	No
3	10-Oct-10	Female	high transmission	20-Jan-11	active follow		36.5	No
4	10-Oct-10	Female	high transmission	17-Feb-11	active follow	4729	36	No

5	10-Oct-10	Female	high transmission	15-Mar-11	active follow	1642	36.5	No
6	10-Oct-10	Female	high transmission	22-Mar-11	passive follow		38	Yes
7	10-Oct-10	Female	high transmission	4-Apr-11	passive follow	9	36.9	No
8	10-Oct-10	Female	high transmission	19-Apr-11	active follow	9	37	No
9	10-Oct-10	Female	high transmission	4-May-11	passive follow	419202	37.8	Yes
10	10-Oct-10	Female	high transmission	14-May-11	active follow	9	37	No
1	23-Oct-10	Female	high transmission	24-Nov-10	active follow	9	36.2	No
2	23-Oct-10	Female	high transmission	23-Dec-10	active follow	9	36.3	No
3	23-Oct-10	Female	high transmission	4-Jan-11	passive follow	9	36.7	Yes
4	23-Oct-10	Female	high transmission	20-Jan-11	active follow		36	No
5	23-Oct-10	Female	high transmission	10-Feb-11	passive follow	9	35.2	Yes
6	23-Oct-10	Female	high transmission	23-Feb-11	active follow	9	36.8	No
7	23-Oct-10	Female	high transmission	9-Mar-11	passive follow	9	36.4	No
8	23-Oct-10	Female	high transmission	22-Mar-11	active follow	9	36.4	No
9	23-Oct-10	Female	high transmission	7-Apr-11	passive follow	9	38.7	Yes
10	23-Oct-10	Female	high transmission	20-Apr-11	active follow	1326	36.4	No
11	23-Oct-10	Female	high transmission	4-May-11	passive follow	23933	37.6	Yes
12	23-Oct-10	Female	high transmission	16-May-11	active follow	9	36.9	No
13	23-Oct-10	Female	high transmission	21-May-11	passive follow	9	37.7	Yes
1	17-Oct-10	Female	high transmission	9-Nov-10	passive follow	9	38.2	Yes
2	17-Oct-10	Female	high transmission	19-Nov-10	active follow	9	36.7	No
3	17-Oct-10	Female	high transmission	21-Dec-10	active follow	9	36.4	No
4	17-Oct-10	Female	high transmission	21-Jan-11	active follow	9	36.8	No
5	17-Oct-10	Female	high transmission	15-Feb-11	active follow	28774	37	No
6	17-Oct-10	Female	high transmission	17-Feb-11	passive follow	13276		
7	17-Oct-10	Female	high transmission	12-Mar-11	active follow	9	36.8	No
8	17-Oct-10	Female	high transmission	11-Apr-11	active follow	113	36.4	No
9	17-Oct-10	Female	high transmission	20-Apr-11	passive follow	32958	39.7	Yes
10	17-Oct-10	Female	high transmission	4-May-11	active follow	9	35.9	No
1	14-Jan-09	Male	high transmission	13-Feb-09	active follow	9	37	No
2	14-Jan-09	Male	high transmission	19-Mar-09	active follow	9	37	No
3	14-Jan-09	Male	high transmission	18-Apr-09	active follow	9	36.3	No
4	14-Jan-09	Male	high transmission	21-May-09	active follow	9	36.5	No
5	14-Jan-09	Male	high transmission	17-Jun-09	active follow	188	36.6	No
6	14-Jan-09	Male	high transmission	19-Jul-09	active follow	754	36.2	No
7	14-Jan-09	Male	high transmission	27-Jul-09	passive follow	19791	36.3	Yes
8	14-Jan-09	Male	high transmission	25-Aug-09	active follow	9	36.5	No
9	14-Jan-09	Male	high transmission	1-Oct-09	active follow			
10	14-Jan-09	Male	high transmission	7-Nov-09	active follow			
11	14-Jan-09	Male	high transmission	15-Dec-09	active follow			
1	3-Jan-09	Male	high transmission	6-Feb-09	active follow	9	36.7	No
2	3-Jan-09	Male	high transmission	11-Mar-09	active follow	9	36.4	No
3	3-Jan-09	Male	high transmission	14-Apr-09	active follow	9	36	No
4	3-Jan-09	Male	high transmission	15-Apr-09	active follow	9		
5	3-Jan-09	Male	high transmission	18-May-09	active follow	9	36.2	No
6	3-Jan-09	Male	high transmission	24-Jun-09	active follow			
7	3-Jan-09	Male	high transmission	28-Jul-09	active follow	540	36.2	No

8	3-Jan-09	Male	high transmission	4-Aug-09	passive follow	81144	38.1	Yes
9	3-Jan-09	Male	high transmission	10-Aug-09	passive follow	9		
10	3-Jan-09	Male	high transmission	2-Sep-09	active follow			
11	3-Jan-09	Male	high transmission	26-Sep-09	passive follow	9	39.2	Yes
12	3-Jan-09	Male	high transmission	5-Oct-09	active follow		36.7	No
13	3-Jan-09	Male	high transmission	7-Nov-09	active follow	9	36.1	No
14	3-Jan-09	Male	high transmission	23-Nov-09	passive follow	15638	37.1	Yes
15	3-Jan-09	Male	high transmission	14-Dec-09	active follow	9	36.6	No
1	19-Jan-09	Male	high transmission	20-Feb-09	active follow	9	35.5	No
2	19-Jan-09	Male	high transmission	26-Mar-09	active follow	9	35.8	No
3	19-Jan-09	Male	high transmission	28-Apr-09	active follow	9	36	No
4	19-Jan-09	Male	high transmission	31-May-09	active follow	2694	36.3	No
5	19-Jan-09	Male	high transmission	6-Jul-09	active follow	4593	35.8	Yes
6	19-Jan-09	Male	high transmission	9-Aug-09	active follow	9	36	No
7	19-Jan-09	Male	high transmission	15-Sep-09	active follow	9	36.2	Yes
8	19-Jan-09	Male	high transmission	19-Oct-09	active follow	9	36.6	Yes
9	19-Jan-09	Male	high transmission	16-Nov-09	active follow	9	36.3	No
10	19-Jan-09	Male	high transmission	22-Dec-09	active follow	9	36	No
1	16-Jan-09	Female	high transmission	16-Feb-09	active follow	9	36.7	No
2	16-Jan-09	Female	high transmission	16-Mar-09	active follow	9	36.5	No
3	16-Jan-09	Female	high transmission	17-Apr-09	active follow	9	36	No
4	16-Jan-09	Female	high transmission	18-May-09	passive follow	1686	38.4	Yes
5	16-Jan-09	Female	high transmission	24-May-09	active follow			
6	16-Jan-09	Female	high transmission	30-Jun-09	active follow			
7	16-Jan-09	Female	high transmission	3-Aug-09	active follow	8142	36.3	No
8	16-Jan-09	Female	high transmission	3-Sep-09	active follow	9	36.3	No
9	16-Jan-09	Female	high transmission	30-Sep-09	active follow		36.7	No
10	16-Jan-09	Female	high transmission	5-Oct-09	passive follow	9600		
11	16-Jan-09	Female	high transmission	29-Oct-09	active follow		36.7	No
12	16-Jan-09	Female	high transmission	23-Nov-09	passive follow	4840		
13	16-Jan-09	Female	high transmission	29-Nov-09	active follow		36.6	No
14	16-Jan-09	Female	high transmission	3-Jan-10	active follow	9	35.8	No
1	26-Jan-09	Female	high transmission	25-Feb-09	active follow	9	36	No
2	26-Jan-09	Female	high transmission	26-Mar-09	active follow	9	36	No
3	26-Jan-09	Female	high transmission	22-Apr-09	active follow	9	36	No
4	26-Jan-09	Female	high transmission	19-May-09	active follow	9	36.1	No
5	26-Jan-09	Female	high transmission	25-Jun-09	active follow			
6	26-Jan-09	Female	high transmission	4-Jul-09	passive follow	9	37.7	Yes
7	26-Jan-09	Female	high transmission	22-Jul-09	active follow	9	36.2	No
8	26-Jan-09	Female	high transmission	19-Aug-09	active follow	3158	36.1	No
9	26-Jan-09	Female	high transmission	25-Aug-09	passive follow	167810	37.1	Yes
10	26-Jan-09	Female	high transmission	16-Sep-09	active follow	9	36	No
11	26-Jan-09	Female	high transmission	14-Oct-09	active follow	3338	36	No
12	26-Jan-09	Female	high transmission	30-Oct-09	passive follow	9	37.1	Yes
13	26-Jan-09	Female	high transmission	14-Nov-09	active follow	9	36	No
14	26-Jan-09	Female	high transmission	27-Nov-09	passive follow	55016	36	Yes
15	26-Jan-09	Female	high transmission	19-Dec-09	active follow	9	36.6	No

16	26-Jan-09	Female	high transmission	22-Jan-10	active follow	9	36	No
1	2-Feb-09	Male	low transmission	13-Feb-09	passive follow		36.6	No
2	2-Feb-09	Male	low transmission	4-Mar-09	active follow	9	36.8	No
3	2-Feb-09	Male	low transmission	6-Apr-09	active follow	9	36.5	No
4	2-Feb-09	Male	low transmission	13-May-09	active follow	9	36	No
5	2-Feb-09	Male	low transmission	12-Jun-09	active follow	9	36	No
6	2-Feb-09	Male	low transmission	15-Jul-09	active follow	9	36.3	No
7	2-Feb-09	Male	low transmission	18-Aug-09	active follow	1527	35.8	No
8	2-Feb-09	Male	low transmission	31-Aug-09	passive follow	71864	36.7	Yes
9	2-Feb-09	Male	low transmission	16-Sep-09	active follow	11331	35.4	No
10	2-Feb-09	Male	low transmission	19-Oct-09	active follow	9	35.5	No
11	2-Feb-09	Male	low transmission	28-Oct-09	passive follow	9	37.8	Yes
12	2-Feb-09	Male	low transmission	23-Nov-09	active follow	9	36.5	No
13	2-Feb-09	Male	low transmission	14-Dec-09	passive follow	9	36.9	Yes
14	2-Feb-09	Male	low transmission	28-Dec-09	active follow	9	36	No
15	2-Feb-09	Male	low transmission	29-Jan-10	active follow	9	36	No
1	1-Feb-09	Female	low transmission	3-Mar-09	active follow	9	36.5	No
2	1-Feb-09	Female	low transmission	2-Apr-09	active follow	9	36.5	No
3	1-Feb-09	Female	low transmission	6-May-09	active follow	9	36.6	No
4	1-Feb-09	Female	low transmission	10-Jun-09	active follow	9	36.1	No
5	1-Feb-09	Female	low transmission	11-Jul-09	active follow	1087	36.3	No
6	1-Feb-09	Female	low transmission	31-Jul-09	passive follow	9	38.8	Yes
7	1-Feb-09	Female	low transmission	14-Aug-09	active follow	9	37.4	No
8	1-Feb-09	Female	low transmission	19-Sep-09	active follow	9	36	No
9	1-Feb-09	Female	low transmission	16-Oct-09	passive follow	22915	35.6	Yes
10	1-Feb-09	Female	low transmission	30-Oct-09	active follow	632	36.3	Yes
11	1-Feb-09	Female	low transmission	14-Dec-09	active follow		36.2	No
12	1-Feb-09	Female	low transmission	15-Dec-09	active follow	9		
13	1-Feb-09	Female	low transmission	14-Jan-10	active follow		36.4	No
1	7-Feb-09	Male	low transmission	11-Mar-09	active follow	9	36.5	No
2	7-Feb-09	Male	low transmission	15-Apr-09	active follow	9	36.3	No
3	7-Feb-09	Male	low transmission	22-May-09	active follow	9	36.3	Yes
4	7-Feb-09	Male	low transmission	5-Jun-09	passive follow	9	36.3	No
5	7-Feb-09	Male	low transmission	24-Jun-09	active follow	9	36	No
6	7-Feb-09	Male	low transmission	23-Jul-09	active follow	9	36.1	No
7	7-Feb-09	Male	low transmission	31-Aug-09	active follow			
8	7-Feb-09	Male	low transmission	7-Sep-09	passive follow		36.1	No
9	7-Feb-09	Male	low transmission	11-Sep-09	passive follow	9		
10	7-Feb-09	Male	low transmission	29-Sep-09	active follow	4250	36.2	No
11	7-Feb-09	Male	low transmission	26-Oct-09	passive follow	47867		
12	7-Feb-09	Male	low transmission	5-Nov-09	active follow		36.3	No
13	7-Feb-09	Male	low transmission	9-Dec-09	active follow		35.9	No
14	7-Feb-09	Male	low transmission	18-Dec-09	passive follow	155538	38.7	Yes
15	7-Feb-09	Male	low transmission	5-Jan-10	active follow		36.2	No
1	12-Feb-09	Male	low transmission	16-Mar-09	active follow	9	36	No
2	12-Feb-09	Male	low transmission	21-Apr-09	active follow	9	36	No
3	12-Feb-09	Male	low transmission	22-May-09	active follow	9	36.6	No

4	12-Feb-09	Male	low transmission	20-Jun-09	passive follow	9	36.3	Yes
5	12-Feb-09	Male	low transmission	28-Jun-09	active follow	9	36.3	No
6	12-Feb-09	Male	low transmission	31-Jul-09	active follow	850	37.7	No
7	12-Feb-09	Male	low transmission	5-Sep-09	active follow	286	36.3	No
8	12-Feb-09	Male	low transmission	8-Oct-09	active follow	273	36.3	No
9	12-Feb-09	Male	low transmission	20-Oct-09	passive follow	9	38	Yes
10	12-Feb-09	Male	low transmission	7-Nov-09	active follow	9	36	No
11	12-Feb-09	Male	low transmission	15-Nov-09	passive follow	9		
12	12-Feb-09	Male	low transmission	16-Nov-09	passive follow		36	Yes
13	12-Feb-09	Male	low transmission	15-Dec-09	active follow	9		No
14	12-Feb-09	Male	low transmission	15-Jan-10	active follow	9	35.5	No
1	9-Feb-09	Female	low transmission	16-Mar-09	active follow	9	36.6	No
2	9-Feb-09	Female	low transmission	16-Apr-09	active follow	9	36.5	No
3	9-Feb-09	Female	low transmission	18-May-09	active follow	9	36.7	No
4	9-Feb-09	Female	low transmission	22-Jun-09	active follow	9	36.3	No
5	9-Feb-09	Female	low transmission	25-Jul-09	active follow	1889	36.4	No
6	9-Feb-09	Female	low transmission	28-Aug-09	active follow	9	36.2	No
7	9-Feb-09	Female	low transmission	1-Oct-09	active follow	9	36.5	Yes
8	9-Feb-09	Female	low transmission	21-Oct-09	passive follow	1131		
9	9-Feb-09	Female	low transmission	26-Oct-09	passive follow		35.9	No
10	9-Feb-09	Female	low transmission	2-Nov-09	passive follow		37	No
11	9-Feb-09	Female	low transmission	7-Nov-09	active follow		36.3	Yes
12	9-Feb-09	Female	low transmission	26-Nov-09	passive follow	12323	36.8	Yes
13	9-Feb-09	Female	low transmission	21-Dec-09	active follow	9	36.1	No
14	9-Feb-09	Female	low transmission	29-Jan-10	active follow	9	36.6	No
16	9-Feb-09	Female	low transmission	9-Feb-10	passive follow	9	35.4	No
1	15-Feb-09	Male	low transmission	17-Mar-09	active follow	9	36.1	No
2	15-Feb-09	Male	low transmission	15-Apr-09	active follow	942	36.2	No
3	15-Feb-09	Male	low transmission	22-May-09	active follow	1373	36	No
4	15-Feb-09	Male	low transmission	26-May-09	passive follow		37.5	Yes
5	15-Feb-09	Male	low transmission	26-Jun-09	active follow	101	36.2	No
6	15-Feb-09	Male	low transmission	7-Jul-09	passive follow	9	37.1	Yes
7	15-Feb-09	Male	low transmission	30-Jul-09	active follow	9	36.5	No
8	15-Feb-09	Male	low transmission	27-Aug-09	active follow	21584	36.1	No
9	15-Feb-09	Male	low transmission	25-Sep-09	active follow	261	36.5	No
10	15-Feb-09	Male	low transmission	2-Oct-09	passive follow	16011		
11	15-Feb-09	Male	low transmission	27-Oct-09	active follow		36	No
12	15-Feb-09	Male	low transmission	8-Nov-09	passive follow		37	Yes
13	15-Feb-09	Male	low transmission	19-Nov-09	passive follow	9		
14	15-Feb-09	Male	low transmission	27-Nov-09	active follow	9	36.2	No
15	15-Feb-09	Male	low transmission	30-Dec-09	active follow	9	36.5	No
16	15-Feb-09	Male	low transmission	26-Jan-10	active follow	4500	37.1	No
1	17-Feb-09	Female	low transmission	16-Mar-09	active follow	9	36.3	No
2	17-Feb-09	Female	low transmission	17-Apr-09	active follow	9	36.8	No
3	17-Feb-09	Female	low transmission	21-May-09	active follow	9	36.3	No
4	17-Feb-09	Female	low transmission	19-Jun-09	passive follow	9	36.7	Yes
5	17-Feb-09	Female	low transmission	22-Jun-09	active follow	9	36.4	No

6	17-Feb-09	Female	low transmission	2-Jul-09	passive follow	5898	37.4	No
7	17-Feb-09	Female	low transmission	23-Jul-09	active follow	40	36.3	No
8	17-Feb-09	Female	low transmission	27-Aug-09	active follow	29896	36.3	Yes
9	17-Feb-09	Female	low transmission	26-Sep-09	active follow	2848	36.5	No
10	17-Feb-09	Female	low transmission	30-Oct-09	active follow	1133	36.5	No
11	17-Feb-09	Female	low transmission	5-Nov-09	passive follow	402	36.4	Yes
12	17-Feb-09	Female	low transmission	4-Dec-09	active follow	9	36.6	No
13	17-Feb-09	Female	low transmission	30-Dec-09	passive follow	9	36	No
14	17-Feb-09	Female	low transmission	8-Jan-10	active follow	9	36.6	No
15	17-Feb-09	Female	low transmission	20-Jan-10	passive follow	9	36.2	No
16	17-Feb-09	Female	low transmission	12-Feb-10	active follow	9	36.7	No
1	15-Feb-09	Female	low transmission	17-Mar-09	active follow	9	36.7	No
2	15-Feb-09	Female	low transmission	19-Apr-09	active follow	9	36	No
3	15-Feb-09	Female	low transmission	22-May-09	active follow	9	36.3	No
4	15-Feb-09	Female	low transmission	24-Jun-09	active follow	9	36.3	No
5	15-Feb-09	Female	low transmission	22-Jul-09	active follow	51140	37	No
6	15-Feb-09	Female	low transmission	20-Aug-09	active follow	11802	36.4	No
7	15-Feb-09	Female	low transmission	17-Sep-09	active follow	632		No
8	15-Feb-09	Female	low transmission	20-Oct-09	active follow	546	35.8	No
9	15-Feb-09	Female	low transmission	10-Nov-09	passive follow	3800	38.6	Yes
10	15-Feb-09	Female	low transmission	27-Nov-09	active follow		36	No
11	15-Feb-09	Female	low transmission	5-Jan-10	active follow	122	36.4	No
12	15-Feb-09	Female	low transmission	9-Feb-10	active follow	9	35.8	No
1	15-Feb-09	Female	low transmission	17-Mar-09	active follow	9	36.8	No
2	15-Feb-09	Female	low transmission	23-Mar-09	passive follow	9	36.5	No
3	15-Feb-09	Female	low transmission	22-Apr-09	active follow	9	36.3	No
4	15-Feb-09	Female	low transmission	23-May-09	active follow	911	36.6	No
5	15-Feb-09	Female	low transmission	27-Jun-09	active follow	8444	36.9	No
6	15-Feb-09	Female	low transmission	16-Jul-09	passive follow	1391	37	Yes
7	15-Feb-09	Female	low transmission	30-Jul-09	active follow	9	36.8	No
8	15-Feb-09	Female	low transmission	27-Aug-09	active follow	9	36.8	No
9	15-Feb-09	Female	low transmission	26-Sep-09	active follow	9	36.5	No
10	15-Feb-09	Female	low transmission	29-Oct-09	active follow	392	36.3	No
11	15-Feb-09	Female	low transmission	24-Nov-09	passive follow	5722	37.2	No
12	15-Feb-09	Female	low transmission	3-Dec-09	active follow	3004	36.5	No
13	15-Feb-09	Female	low transmission	1-Jan-10	active follow	9	36.3	No
14	15-Feb-09	Female	low transmission	8-Feb-10	active follow	9	36.7	No
1	22-Feb-09	Female	low transmission	27-Mar-09	active follow	9	36.3	No
2	22-Feb-09	Female	low transmission	4-May-09	active follow	9	36.3	No
3	22-Feb-09	Female	low transmission	7-Jun-09	active follow	9	36.8	No
4	22-Feb-09	Female	low transmission	11-Jul-09	active follow	9	36	No
5	22-Feb-09	Female	low transmission	14-Aug-09	active follow		36.2	No
6	22-Feb-09	Female	low transmission	17-Sep-09	passive follow	342	40.1	Yes
7	22-Feb-09	Female	low transmission	21-Sep-09	active follow		40.1	Yes
8	22-Feb-09	Female	low transmission	19-Oct-09	passive follow		38	Yes
9	22-Feb-09	Female	low transmission	24-Oct-09	active follow		36	No
10	22-Feb-09	Female	low transmission	3-Nov-09	passive follow	9	36.8	Yes

11	22-Feb-09	Female	low transmission	26-Nov-09	active follow	9	36.2	No
12	22-Feb-09	Female	low transmission	26-Dec-09	active follow	8537	36.4	No
13	22-Feb-09	Female	low transmission	16-Jan-10	passive follow	9	36.3	No
14	22-Feb-09	Female	low transmission	30-Jan-10	active follow	9	36.3	No
1	19-Feb-09	Male	low transmission	21-Mar-09	active follow	9	36.8	No
2	19-Feb-09	Male	low transmission	24-Apr-09	active follow	9	36.6	No
3	19-Feb-09	Male	low transmission	30-May-09	active follow	250	36.3	No
4	19-Feb-09	Male	low transmission	26-Jun-09	passive follow	25766	37.4	Yes
5	19-Feb-09	Male	low transmission	7-Jul-09	active follow			
6	19-Feb-09	Male	low transmission	27-Jul-09	passive follow	9	36.9	Yes
7	19-Feb-09	Male	low transmission	12-Aug-09	active follow	9	36.1	No
8	19-Feb-09	Male	low transmission	14-Sep-09	active follow	9	35.8	No
9	19-Feb-09	Male	low transmission	29-Sep-09	passive follow	10336	38.8	Yes
10	19-Feb-09	Male	low transmission	17-Oct-09	active follow		37	No
11	19-Feb-09	Male	low transmission	16-Nov-09	active follow	9	36.5	No
12	19-Feb-09	Male	low transmission	30-Nov-09	passive follow	9	36	Yes
13	19-Feb-09	Male	low transmission	21-Dec-09	active follow	9	37.5	Yes
14	19-Feb-09	Male	low transmission	27-Jan-10	active follow			
1	23-Feb-09	Female	low transmission	26-Mar-09	active follow	9	36.7	No
2	23-Feb-09	Female	low transmission	28-Apr-09	active follow	9		
3	23-Feb-09	Female	low transmission	29-Apr-09	active follow	9	35.9	No
4	23-Feb-09	Female	low transmission	30-May-09	active follow	9	36.7	No
5	23-Feb-09	Female	low transmission	6-Jul-09	active follow	9	36.5	No
6	23-Feb-09	Female	low transmission	30-Jul-09	passive follow	9	36.5	No
7	23-Feb-09	Female	low transmission	7-Aug-09	active follow	9	35.9	No
8	23-Feb-09	Female	low transmission	11-Sep-09	active follow	3570	36.3	No
9	23-Feb-09	Female	low transmission	7-Oct-09	passive follow	29	38.2	Yes
10	23-Feb-09	Female	low transmission	14-Oct-09	active follow		36.5	Yes
11	23-Feb-09	Female	low transmission	12-Nov-09	active follow	9	36.5	No
12	23-Feb-09	Female	low transmission	20-Nov-09	passive follow	9		
13	23-Feb-09	Female	low transmission	12-Dec-09	active follow	9	36.5	No
14	23-Feb-09	Female	low transmission	15-Jan-10	active follow	9	36.6	No
15	23-Feb-09	Female	low transmission	17-Feb-10	active follow	9	36.3	No
1	15-Feb-09	Male	low transmission	17-Mar-09	active follow	9	37	Yes
2	15-Feb-09	Male	low transmission	15-Apr-09	active follow	9	36	No
3	15-Feb-09	Male	low transmission	22-May-09	active follow	196	36	No
4	15-Feb-09	Male	low transmission	26-Jun-09	active follow	1401	36.6	Yes
5	15-Feb-09	Male	low transmission	30-Jul-09	active follow	9	36.5	No
6	15-Feb-09	Male	low transmission	27-Aug-09	active follow	3861	36	No
7	15-Feb-09	Male	low transmission	9-Sep-09	passive follow	21759		
8	15-Feb-09	Male	low transmission	14-Sep-09	passive follow		36.9	Yes
9	15-Feb-09	Male	low transmission	25-Sep-09	active follow	9	36	No
10	15-Feb-09	Male	low transmission	27-Oct-09	active follow		36.1	No
11	15-Feb-09	Male	low transmission	27-Nov-09	active follow	9	36.3	No
12	15-Feb-09	Male	low transmission	16-Dec-09	passive follow	231619	39.4	Yes
13	15-Feb-09	Male	low transmission	2-Jan-10	active follow	9	36.3	No
14	15-Feb-09	Male	low transmission	13-Jan-10	passive follow	9	38	Yes

15	15-Feb-09	Male	low transmission	8-Feb-10	active follow		36.8	No
17	15-Feb-09	Male	low transmission	15-Feb-10	passive follow	27116	38	Yes
1	17-Feb-09	Male	low transmission	19-Mar-09	active follow	9	37	No
2	17-Feb-09	Male	low transmission	20-Apr-09	active follow	9	36.6	No
3	17-Feb-09	Male	low transmission	26-May-09	active follow	9	36.5	No
4	17-Feb-09	Male	low transmission	30-Jun-09	active follow	9	36	No
5	17-Feb-09	Male	low transmission	3-Aug-09	active follow	9	36.7	No
6	17-Feb-09	Male	low transmission	7-Sep-09	active follow	9	36.8	No
7	17-Feb-09	Male	low transmission	14-Oct-09	active follow	2768	36.8	No
8	17-Feb-09	Male	low transmission	15-Oct-09	passive follow	2847	37.6	Yes
9	17-Feb-09	Male	low transmission	20-Nov-09	active follow	10479	36.8	No
10	17-Feb-09	Male	low transmission	25-Dec-09	active follow	969	36.7	No
11	17-Feb-09	Male	low transmission	2-Feb-10	active follow		36.3	No
1	1-Mar-09	Male	low transmission	31-Mar-09	active follow	9	36.3	No
2	1-Mar-09	Male	low transmission	28-Apr-09	active follow	9	36.7	No
3	1-Mar-09	Male	low transmission	31-May-09	active follow	9861	36.3	No
4	1-Mar-09	Male	low transmission	1-Jul-09	active follow		36.4	No
5	1-Mar-09	Male	low transmission	29-Jul-09	active follow	43131	36.9	No
6	1-Mar-09	Male	low transmission	31-Jul-09	passive follow	2042	37	Yes
7	1-Mar-09	Male	low transmission	24-Aug-09	passive follow	4393	36.8	Yes
8	1-Mar-09	Male	low transmission	28-Aug-09	active follow		36	No
9	1-Mar-09	Male	low transmission	29-Sep-09	active follow			
10	1-Mar-09	Male	low transmission	30-Sep-09	passive follow	4906	37	Yes
11	1-Mar-09	Male	low transmission	8-Oct-09	passive follow	872	36.6	No
12	1-Mar-09	Male	low transmission	27-Oct-09	active follow	2616	36.1	Yes
13	1-Mar-09	Male	low transmission	26-Nov-09	active follow	163	36.5	No
14	1-Mar-09	Male	low transmission	7-Dec-09	passive follow	9	36.1	No
15	1-Mar-09	Male	low transmission	31-Dec-09	active follow	9	35.5	No
16	1-Mar-09	Male	low transmission	3-Feb-10	passive follow	9	36.5	No
1	7-Mar-09	Female	low transmission	10-Apr-09	active follow	9	36.7	No
2	7-Mar-09	Female	low transmission	13-May-09	active follow	9	36.4	No
3	7-Mar-09	Female	low transmission	13-Jun-09	active follow	425	36.3	No
4	7-Mar-09	Female	low transmission	15-Jul-09	active follow	9	36.2	No
5	7-Mar-09	Female	low transmission	15-Aug-09	active follow	30386	37.1	Yes
6	7-Mar-09	Female	low transmission	17-Aug-09	passive follow	696	36.4	Yes
7	7-Mar-09	Female	low transmission	16-Sep-09	active follow	9	36	No
8	7-Mar-09	Female	low transmission	23-Oct-09	active follow			
9	7-Mar-09	Female	low transmission	10-Nov-09	passive follow	9		
10	7-Mar-09	Female	low transmission	29-Nov-09	active follow			
11	7-Mar-09	Female	low transmission	23-Dec-09	passive follow	9		
12	7-Mar-09	Female	low transmission	2-Jan-10	active follow	9	35.8	No
13	7-Mar-09	Female	low transmission	23-Jan-10	passive follow		36.9	Yes
14	7-Mar-09	Female	low transmission	26-Jan-10	passive follow	9		
15	7-Mar-09	Female	low transmission	3-Feb-10	passive follow	9	35.6	No
16	7-Mar-09	Female	low transmission	6-Feb-10	active follow	9	36.4	No
1	13-Mar-09	Female	low transmission	18-Apr-09	active follow	9	36.7	No
2	13-Mar-09	Female	low transmission	23-May-09	active follow	1032	36.6	No

3	13-Mar-09	Female	low transmission	29-Jun-09	active follow	1901	37.1	No
4	13-Mar-09	Female	low transmission	1-Aug-09	active follow	9	37	No
5	13-Mar-09	Female	low transmission	3-Sep-09	active follow		36.3	No
6	13-Mar-09	Female	low transmission	3-Oct-09	active follow	2572	37	No
7	13-Mar-09	Female	low transmission	21-Oct-09	passive follow	14340	36	No
8	13-Mar-09	Female	low transmission	7-Nov-09	active follow	9	36.5	No
9	13-Mar-09	Female	low transmission	10-Dec-09	active follow	9	36.5	No
10	13-Mar-09	Female	low transmission	16-Jan-10	active follow			
11	13-Mar-09	Female	low transmission	15-Feb-10	passive follow	9	35.8	Yes
12	13-Mar-09	Female	low transmission	22-Feb-10	active follow	9	36.3	No
13	13-Mar-09	Female	low transmission	8-Mar-10	passive follow	9	36	No
1	9-Mar-09	Male	low transmission	14-Apr-09	active follow	9	36.3	No
2	9-Mar-09	Male	low transmission	15-May-09	active follow	9	36.3	No
3	9-Mar-09	Male	low transmission	19-Jun-09	active follow	9	36.3	No
4	9-Mar-09	Male	low transmission	23-Jul-09	active follow	9	36.1	No
5	9-Mar-09	Male	low transmission	25-Aug-09	passive follow	1570	39	Yes
6	9-Mar-09	Male	low transmission	28-Aug-09	active follow	9	36	No
7	9-Mar-09	Male	low transmission	26-Sep-09	passive follow	148227	36.8	Yes
8	9-Mar-09	Male	low transmission	3-Oct-09	active follow	132	36	No
9	9-Mar-09	Male	low transmission	9-Nov-09	active follow	2562	36	No
10	9-Mar-09	Male	low transmission	8-Dec-09	passive follow	751144	38.1	Yes
11	9-Mar-09	Male	low transmission	15-Dec-09	active follow	9	36.6	No
12	9-Mar-09	Male	low transmission	4-Jan-10	passive follow	54287	38.5	Yes
13	9-Mar-09	Male	low transmission	21-Jan-10	active follow	9	36.6	No
14	9-Mar-09	Male	low transmission	20-Feb-10	active follow	9	36	No
1	10-Mar-09	Male	low transmission	10-Apr-09	active follow	9	36.5	No
2	10-Mar-09	Male	low transmission	16-May-09	active follow	9	36.8	No
3	10-Mar-09	Male	low transmission	6-Jun-09	passive follow	9	37.1	Yes
4	10-Mar-09	Male	low transmission	22-Jun-09	active follow	9	36.6	No
5	10-Mar-09	Male	low transmission	24-Jun-09	passive follow	9	36.7	No
6	10-Mar-09	Male	low transmission	13-Jul-09	passive follow	9	36.7	No
7	10-Mar-09	Male	low transmission	28-Jul-09	active follow	9	36.8	No
8	10-Mar-09	Male	low transmission	28-Aug-09	active follow		36.3	No
9	10-Mar-09	Male	low transmission	7-Sep-09	passive follow	3797	36.9	No
10	10-Mar-09	Male	low transmission	30-Sep-09	active follow	1262	36.5	No
11	10-Mar-09	Male	low transmission	29-Oct-09	active follow	504	36.5	No
12	10-Mar-09	Male	low transmission	28-Nov-09	active follow	682	36.5	No
13	10-Mar-09	Male	low transmission	10-Dec-09	passive follow	1036	37.3	Yes
14	10-Mar-09	Male	low transmission	30-Dec-09	active follow	9	36.4	No
15	10-Mar-09	Male	low transmission	26-Jan-10	active follow	9	36.7	No
16	10-Mar-09	Male	low transmission	19-Feb-10	passive follow	9	37.3	Yes
17	10-Mar-09	Male	low transmission	24-Feb-10	active follow	9	36.8	No
1	19-Mar-09	Female	low transmission	18-Apr-09	active follow	9	36.5	No
2	19-Mar-09	Female	low transmission	21-May-09	active follow	9	36.5	No
3	19-Mar-09	Female	low transmission	18-Jun-09	active follow	9	36.3	No
4	19-Mar-09	Female	low transmission	16-Jul-09	active follow	4732	36.5	No
5	19-Mar-09	Female	low transmission	10-Aug-09	passive follow	64219	37.5	Yes

6	19-Mar-09	Female	low transmission	12-Sep-09	active follow	9	36.2	No
7	19-Mar-09	Female	low transmission	18-Oct-09	active follow	9	36	No
8	19-Mar-09	Female	low transmission	2-Nov-09	passive follow	371	36.6	No
9	19-Mar-09	Female	low transmission	23-Nov-09	active follow		37.1	No
10	19-Mar-09	Female	low transmission	31-Dec-09	active follow	9	36.6	No
11	19-Mar-09	Female	low transmission	3-Feb-10	active follow	9	36.7	No
12	19-Mar-09	Female	low transmission	6-Mar-10	active follow		36.5	No
13	19-Mar-09	Female	low transmission	18-Mar-10	passive follow	9	36.4	Yes
1	1-Apr-09	Female	high transmission	10-Apr-09	passive follow	9	37.2	Yes
2	1-Apr-09	Female	high transmission	29-Apr-09	active follow	9	36.5	No
3	1-Apr-09	Female	high transmission	2-Jun-09	active follow	9	36.5	No
4	1-Apr-09	Female	high transmission	1-Jul-09	active follow	9	37.6	Yes
5	1-Apr-09	Female	high transmission	29-Jul-09	active follow	9	37	No
6	1-Apr-09	Female	high transmission	26-Aug-09	active follow	9	36.8	No
7	1-Apr-09	Female	high transmission	24-Sep-09	active follow	9	37	No
8	1-Apr-09	Female	high transmission	14-Oct-09	passive follow	963	36.8	Yes
9	1-Apr-09	Female	high transmission	26-Oct-09	passive follow	9	37.1	Yes
10	1-Apr-09	Female	high transmission	26-Nov-09	active follow	36199	36.8	Yes
11	1-Apr-09	Female	high transmission	25-Dec-09	active follow	103600	36	No
12	1-Apr-09	Female	high transmission	28-Jan-10	active follow		37	No
13	1-Apr-09	Female	high transmission	26-Feb-10	active follow	9	36	No
14	1-Apr-09	Female	high transmission	31-Mar-10	active follow	9	36.1	Yes
1	1-Apr-09	Male	high transmission	6-May-09	active follow	9	36.3	No
2	1-Apr-09	Male	high transmission	10-Jun-09	active follow	9	36.4	Yes
3	1-Apr-09	Male	high transmission	10-Jul-09	active follow	9	36.6	No
4	1-Apr-09	Male	high transmission	29-Jul-09	passive follow	524	37.9	Yes
5	1-Apr-09	Male	high transmission	16-Aug-09	active follow			
6	1-Apr-09	Male	high transmission	24-Sep-09	active follow	1448	36.5	Yes
7	1-Apr-09	Male	high transmission	24-Oct-09	active follow	3739	36.2	No
8	1-Apr-09	Male	high transmission	18-Nov-09	passive follow	9	37.3	Yes
9	1-Apr-09	Male	high transmission	1-Dec-09	active follow	1192	36.5	No
10	1-Apr-09	Male	high transmission	10-Dec-09	passive follow	119180	37	Yes
11	1-Apr-09	Male	high transmission	31-Dec-09	active follow	9	36.5	No
12	1-Apr-09	Male	high transmission	28-Jan-10	active follow	9	38	Yes
13	1-Apr-09	Male	high transmission	24-Feb-10	active follow	9	36.4	No
14	1-Apr-09	Male	high transmission	25-Mar-10	active follow	9	36.7	No
15	1-Apr-09	Male	high transmission	30-Mar-10	passive follow	9	36	No
1	31-Mar-09	Male	low transmission	6-May-09	active follow	9	36.3	No
2	31-Mar-09	Male	low transmission	7-Jun-09	active follow	9	36.2	No
3	31-Mar-09	Male	low transmission	16-Jul-09	active follow	9	36	No
4	31-Mar-09	Male	low transmission	17-Aug-09	active follow	9	36.1	No
5	31-Mar-09	Male	low transmission	20-Sep-09	active follow	9	36	No
6	31-Mar-09	Male	low transmission	27-Sep-09	passive follow	75616	38.4	No
7	31-Mar-09	Male	low transmission	27-Oct-09	active follow		36.1	No
8	31-Mar-09	Male	low transmission	1-Dec-09	active follow	9	36	No
9	31-Mar-09	Male	low transmission	5-Jan-10	active follow	10983	36.6	No
10	31-Mar-09	Male	low transmission	3-Feb-10	passive follow	1820	36.3	Yes

11	31-Mar-09	Male	low transmission	11-Feb-10	active follow	9	36.5	No
12	31-Mar-09	Male	low transmission	25-Feb-10	passive follow	5533	37.7	Yes
13	31-Mar-09	Male	low transmission	20-Mar-10	active follow	10167	36.4	No
1	5-Apr-09	Male	high transmission	12-Apr-09	passive follow	9	36.6	No
2	5-Apr-09	Male	high transmission	11-May-09	active follow	9	36.5	No
3	5-Apr-09	Male	high transmission	12-Jun-09	active follow	9	36.6	No
4	5-Apr-09	Male	high transmission	15-Jul-09	active follow	9	36	No
5	5-Apr-09	Male	high transmission	14-Aug-09	active follow	898	35.8	No
6	5-Apr-09	Male	high transmission	17-Aug-09	passive follow	4423	38.1	No
7	5-Apr-09	Male	high transmission	16-Sep-09	active follow	4203	35.4	No
8	5-Apr-09	Male	high transmission	13-Oct-09	active follow	38579	36.8	Yes
9	5-Apr-09	Male	high transmission	13-Nov-09	active follow	34870	36.2	No
10	5-Apr-09	Male	high transmission	1-Dec-09	passive follow	2297	36.3	No
11	5-Apr-09	Male	high transmission	15-Dec-09	passive follow	8159	37.1	Yes
12	5-Apr-09	Male	high transmission	19-Dec-09	active follow	5086	35.9	No
13	5-Apr-09	Male	high transmission	28-Dec-09	passive follow	9	35.5	No
14	5-Apr-09	Male	high transmission	13-Jan-10	passive follow	7359	37.1	Yes
15	5-Apr-09	Male	high transmission	28-Jan-10	active follow	9	36.5	No
16	5-Apr-09	Male	high transmission	22-Feb-10	active follow	9		
17	5-Apr-09	Male	high transmission	23-Feb-10	active follow	9	37.1	Yes
18	5-Apr-09	Male	high transmission	21-Mar-10	passive follow	9	35.5	Yes
19	5-Apr-09	Male	high transmission	30-Mar-10	active follow	9	36.5	No
20	5-Apr-09	Male	high transmission	31-Mar-10	active follow	9		
1	7-Apr-09	Female	high transmission	7-May-09	active follow	9	36.6	No
2	7-Apr-09	Female	high transmission	9-Jun-09	active follow	9	36	No
3	7-Apr-09	Female	high transmission	13-Jul-09	active follow	2734	36.6	No
4	7-Apr-09	Female	high transmission	9-Aug-09	active follow	9	39.4	Yes
5	7-Apr-09	Female	high transmission	10-Aug-09	passive follow	9		
6	7-Apr-09	Female	high transmission	14-Aug-09	passive follow	9		
7	7-Apr-09	Female	high transmission	14-Sep-09	active follow	9	38	Yes
8	7-Apr-09	Female	high transmission	17-Oct-09	passive follow	9	37.1	Yes
9	7-Apr-09	Female	high transmission	19-Nov-09	active follow	9	36.8	No
10	7-Apr-09	Female	high transmission	21-Dec-09	active follow	23496	37	Yes
11	7-Apr-09	Female	high transmission	24-Jan-10	active follow	9	36.3	No
12	7-Apr-09	Female	high transmission	8-Feb-10	passive follow	9	36.4	Yes
13	7-Apr-09	Female	high transmission	25-Feb-10	active follow	9	35.7	No
14	7-Apr-09	Female	high transmission	9-Mar-10	passive follow	9	35.7	No
15	7-Apr-09	Female	high transmission	27-Mar-10	active follow	9	36	No
1	7-Apr-09	Female	high transmission	8-May-09	active follow	9	36.6	No
2	7-Apr-09	Female	high transmission	13-Jun-09	active follow	9	36	No
3	7-Apr-09	Female	high transmission	20-Jul-09	active follow			
4	7-Apr-09	Female	high transmission	28-Aug-09	active follow	9	36.3	No
5	7-Apr-09	Female	high transmission	1-Oct-09	active follow	9	36.3	No
6	7-Apr-09	Female	high transmission	2-Nov-09	active follow	7016	36.5	No
7	7-Apr-09	Female	high transmission	6-Nov-09	passive follow	3734	38.4	Yes
8	7-Apr-09	Female	high transmission	7-Dec-09	active follow	9	36.7	No
9	7-Apr-09	Female	high transmission	8-Jan-10	active follow	9	36.5	No

10	7-Apr-09	Female	high transmission	13-Feb-10	active follow	5342	36.2	No
11	7-Apr-09	Female	high transmission	23-Feb-10	passive follow	2199	35.4	Yes
12	7-Apr-09	Female	high transmission	20-Mar-10	active follow	9	36.7	No
13	7-Apr-09	Female	high transmission	29-Mar-10	passive follow	9	36.7	Yes
1	10-Apr-09	Male	high transmission	15-May-09	active follow	9	36.6	No
2	10-Apr-09	Male	high transmission	22-Jun-09	active follow	9	37	Yes
3	10-Apr-09	Male	high transmission	28-Jul-09	active follow	964	36.6	No
4	10-Apr-09	Male	high transmission	10-Aug-09	passive follow	23108	37.6	Yes
5	10-Apr-09	Male	high transmission	30-Aug-09	active follow		36.7	No
6	10-Apr-09	Male	high transmission	30-Sep-09	active follow	9	36.5	No
7	10-Apr-09	Male	high transmission	2-Oct-09	passive follow	9	35.5	No
8	10-Apr-09	Male	high transmission	29-Oct-09	active follow	9	37.4	No
9	10-Apr-09	Male	high transmission	4-Nov-09	passive follow	93167	36.8	Yes
10	10-Apr-09	Male	high transmission	30-Nov-09	active follow	6201	36.2	No
11	10-Apr-09	Male	high transmission	2-Dec-09	passive follow			
12	10-Apr-09	Male	high transmission	30-Dec-09	active follow	9	35.5	No
13	10-Apr-09	Male	high transmission	20-Jan-10	passive follow	4376	35.4	No
14	10-Apr-09	Male	high transmission	26-Jan-10	active follow	9	35.5	No
15	10-Apr-09	Male	high transmission	24-Feb-10	active follow	2231	35.5	No
16	10-Apr-09	Male	high transmission	22-Mar-10	active follow	5056	35.5	No
1	9-Apr-09	Male	high transmission	12-May-09	active follow	9	36.6	No
2	9-Apr-09	Male	high transmission	13-Jun-09	active follow	9	36.8	No
3	9-Apr-09	Male	high transmission	17-Jul-09	active follow	593	36.1	No
4	9-Apr-09	Male	high transmission	27-Aug-09	active follow		36.1	No
5	9-Apr-09	Male	high transmission	2-Sep-09	passive follow		36.5	No
6	9-Apr-09	Male	high transmission	28-Sep-09	active follow	8585	36.3	No
7	9-Apr-09	Male	high transmission	2-Oct-09	passive follow	1830		
8	9-Apr-09	Male	high transmission	3-Oct-09	passive follow		36.1	No
9	9-Apr-09	Male	high transmission	4-Nov-09	active follow		36.5	No
10	9-Apr-09	Male	high transmission	23-Nov-09	passive follow	19663	36.7	No
11	9-Apr-09	Male	high transmission	9-Dec-09	active follow	4598	36.1	No
12	9-Apr-09	Male	high transmission	7-Jan-10	active follow	9	36.4	No
13	9-Apr-09	Male	high transmission	8-Feb-10	active follow	1677	35.8	No
14	9-Apr-09	Male	high transmission	18-Mar-10	active follow	9	35.8	No
1	12-Apr-09	Female	high transmission	29-Apr-09	passive follow		39.9	Yes
2	12-Apr-09	Female	high transmission	18-May-09	active follow	9	35.7	No
3	12-Apr-09	Female	high transmission	5-Jun-09	passive follow	9	37.3	Yes
4	12-Apr-09	Female	high transmission	17-Jun-09	passive follow		36.5	No
5	12-Apr-09	Female	high transmission	18-Jun-09	active follow	9	35.5	No
6	12-Apr-09	Female	high transmission	8-Jul-09	passive follow	9	38.4	Yes
7	12-Apr-09	Female	high transmission	16-Jul-09	active follow	9	36.1	No
8	12-Apr-09	Female	high transmission	14-Aug-09	active follow	521	35.7	No
9	12-Apr-09	Female	high transmission	16-Sep-09	active follow	2274	36.3	No
10	12-Apr-09	Female	high transmission	20-Sep-09	passive follow		36.5	No
11	12-Apr-09	Female	high transmission	13-Oct-09	active follow	1461	36.2	No
12	12-Apr-09	Female	high transmission	28-Oct-09	passive follow	9	38.3	Yes
13	12-Apr-09	Female	high transmission	13-Nov-09	active follow	9	36.5	No

14	12-Apr-09	Female	high transmission	10-Dec-09	passive follow	721875	39.1	Yes
15	12-Apr-09	Female	high transmission	19-Dec-09	active follow	9	36.3	No
16	12-Apr-09	Female	high transmission	30-Dec-09	passive follow	9	36.2	No
17	12-Apr-09	Female	high transmission	23-Jan-10	active follow	9	35.5	No
18	12-Apr-09	Female	high transmission	24-Feb-10	active follow	9	36.8	No
19	12-Apr-09	Female	high transmission	25-Mar-10	active follow	9	35.7	No
1	4-Apr-09	Male	high transmission	8-May-09	active follow	9	36.5	No
2	4-Apr-09	Male	high transmission	11-Jun-09	active follow	9	36.6	No
3	4-Apr-09	Male	high transmission	15-Jul-09	active follow	953	36.2	No
4	4-Apr-09	Male	high transmission	1-Aug-09	passive follow	6306	37.3	Yes
5	4-Apr-09	Male	high transmission	18-Aug-09	active follow	9	36.5	No
6	4-Apr-09	Male	high transmission	24-Sep-09	active follow	9	36	No
7	4-Apr-09	Male	high transmission	8-Oct-09	passive follow	9	36.3	No
8	4-Apr-09	Male	high transmission	30-Oct-09	active follow	9	36.5	No
9	4-Apr-09	Male	high transmission	4-Dec-09	active follow	9	36	No
10	4-Apr-09	Male	high transmission	15-Dec-09	passive follow	9	36.5	No
11	4-Apr-09	Male	high transmission	8-Jan-10	active follow	9	36.2	No
12	4-Apr-09	Male	high transmission	13-Feb-10	active follow	9	36	No
13	4-Apr-09	Male	high transmission	16-Feb-10	passive follow	9	36.1	No
14	4-Apr-09	Male	high transmission	9-Mar-10	passive follow	9		
15	4-Apr-09	Male	high transmission	18-Mar-10	active follow	9	36.4	No
1	17-Apr-09	Male	high transmission	27-May-09	active follow	9	36.3	No
2	17-Apr-09	Male	high transmission	24-Jun-09	active follow	9	38.7	No
3	17-Apr-09	Male	high transmission	22-Jul-09	active follow	9	36.4	No
4	17-Apr-09	Male	high transmission	14-Aug-09	passive follow	478	36.8	No
5	17-Apr-09	Male	high transmission	28-Aug-09	active follow		36	No
6	17-Apr-09	Male	high transmission	29-Sep-09	active follow	1704	36	No
7	17-Apr-09	Male	high transmission	7-Oct-09	passive follow	5880		
8	17-Apr-09	Male	high transmission	27-Oct-09	active follow	9	36	No
9	17-Apr-09	Male	high transmission	10-Nov-09	passive follow	9782	36.8	Yes
10	17-Apr-09	Male	high transmission	28-Nov-09	active follow	9	36	No
11	17-Apr-09	Male	high transmission	25-Dec-09	passive follow	9	37.5	Yes
12	17-Apr-09	Male	high transmission	3-Jan-10	active follow		36.3	No
13	17-Apr-09	Male	high transmission	12-Jan-10	passive follow	99875	38.5	Yes
14	17-Apr-09	Male	high transmission	6-Feb-10	active follow	9	36.6	No
15	17-Apr-09	Male	high transmission	15-Mar-10	active follow	9	36.6	Yes
16	17-Apr-09	Male	high transmission	15-Apr-10	active follow	9	36	Yes
1	22-Apr-09	Male	high transmission	21-May-09	active follow	9	36.9	No
2	22-Apr-09	Male	high transmission	22-Jun-09	active follow	4968	36.9	No
3	22-Apr-09	Male	high transmission	23-Jul-09	active follow	16271	36.2	No
4	22-Apr-09	Male	high transmission	26-Aug-09	active follow	3383	37	No
5	22-Apr-09	Male	high transmission	27-Sep-09	active follow	5466	38	Yes
6	22-Apr-09	Male	high transmission	3-Nov-09	active follow	69229	37	Yes
7	22-Apr-09	Male	high transmission	4-Dec-09	active follow	9	35.5	No
8	22-Apr-09	Male	high transmission	8-Jan-10	active follow	1534	36.6	No
9	22-Apr-09	Male	high transmission	9-Feb-10	passive follow	2580	36.8	No
10	22-Apr-09	Male	high transmission	14-Feb-10	active follow		36.8	No

11	22-Apr-09	Male	high transmission	17-Mar-10	active follow	4402	36.8	No
12	22-Apr-09	Male	high transmission	16-Apr-10	active follow	1876	36.5	No
1	27-Apr-09	Male	high transmission	29-May-09	active follow	9	36.5	No
2	27-Apr-09	Male	high transmission	23-Jun-09	passive follow	9	36.6	Yes
3	27-Apr-09	Male	high transmission	1-Jul-09	active follow	9	36.9	No
4	27-Apr-09	Male	high transmission	31-Jul-09	active follow	9	36	No
5	27-Apr-09	Male	high transmission	12-Aug-09	passive follow	9	37.1	No
6	27-Apr-09	Male	high transmission	2-Sep-09	active follow	9	36.8	No
7	27-Apr-09	Male	high transmission	2-Oct-09	active follow	9	36.3	No
8	27-Apr-09	Male	high transmission	1-Nov-09	passive follow	4696	36.5	Yes
9	27-Apr-09	Male	high transmission	5-Nov-09	active follow		36.3	No
10	27-Apr-09	Male	high transmission	3-Dec-09	active follow	11546	35.4	No
11	27-Apr-09	Male	high transmission	9-Jan-10	active follow			
12	27-Apr-09	Male	high transmission	15-Feb-10	active follow			
13	27-Apr-09	Male	high transmission	17-Mar-10	active follow			
14	27-Apr-09	Male	high transmission	23-Apr-10	active follow			
1	29-Apr-09	Female	high transmission	4-Jun-09	active follow	9	36.3	No
2	29-Apr-09	Female	high transmission	8-Jul-09	active follow	9	36.5	No
3	29-Apr-09	Female	high transmission	13-Aug-09	active follow	9	36.8	No
4	29-Apr-09	Female	high transmission	17-Sep-09	active follow	9	36.8	No
5	29-Apr-09	Female	high transmission	22-Oct-09	active follow	1825	36.8	No
6	29-Apr-09	Female	high transmission	2-Nov-09	passive follow	9	37.8	Yes
7	29-Apr-09	Female	high transmission	24-Nov-09	active follow	9	36	No
8	29-Apr-09	Female	high transmission	31-Dec-09	active follow			
9	29-Apr-09	Female	high transmission	2-Feb-10	active follow		36	No
10	29-Apr-09	Female	high transmission	5-Mar-10	active follow	219	37.1	Yes
11	29-Apr-09	Female	high transmission	9-Apr-10	active follow	9	36.1	No
12	29-Apr-09	Female	high transmission	17-Apr-10	passive follow	9	38.4	Yes
1	1-May-09	Female	high transmission	8-Jun-09	active follow	9	36.2	No
2	1-May-09	Female	high transmission	9-Jul-09	active follow	9	36.3	No
3	1-May-09	Female	high transmission	10-Aug-09	active follow	9	35.5	No
4	1-May-09	Female	high transmission	10-Sep-09	active follow	9	36.4	No
5	1-May-09	Female	high transmission	7-Oct-09	active follow	9	36.4	No
6	1-May-09	Female	high transmission	6-Nov-09	active follow		36.3	No
7	1-May-09	Female	high transmission	26-Nov-09	passive follow	14753	38.8	Yes
8	1-May-09	Female	high transmission	9-Dec-09	active follow	9		
9	1-May-09	Female	high transmission	10-Dec-09	active follow	9	36.5	No
10	1-May-09	Female	high transmission	6-Jan-10	active follow	1319	36.2	No
11	1-May-09	Female	high transmission	5-Feb-10	active follow	3959	36.1	No
12	1-May-09	Female	high transmission	16-Mar-10	active follow	11711	36.5	No
13	1-May-09	Female	high transmission	14-Apr-10	active follow	201	36.2	No
1	6-May-09	Male	high transmission	13-Jun-09	active follow		36.6	No
2	6-May-09	Male	high transmission	17-Jul-09	active follow	9	36.2	No
3	6-May-09	Male	high transmission	22-Aug-09	active follow	119	37	No
4	6-May-09	Male	high transmission	24-Sep-09	active follow	9	36.4	No
5	6-May-09	Male	high transmission	22-Oct-09	active follow	9	36	No
6	6-May-09	Male	high transmission	1-Nov-09	passive follow	9	37.3	No

7	6-May-09	Male	high transmission	18-Nov-09	active follow	6982	36.3	No
8	6-May-09	Male	high transmission	17-Dec-09	active follow	14710	36.5	No
9	6-May-09	Male	high transmission	27-Dec-09	passive follow	11994	36.1	Yes
10	6-May-09	Male	high transmission	30-Dec-09	passive follow	9		
11	6-May-09	Male	high transmission	16-Jan-10	active follow	9	36	No
12	6-May-09	Male	high transmission	18-Feb-10	active follow	9	36	No
13	6-May-09	Male	high transmission	18-Mar-10	active follow	9	36.9	Yes
14	6-May-09	Male	high transmission	16-Apr-10	passive follow	220816	36.6	Yes
1	5-May-09	Female	high transmission	6-Jun-09	active follow	9	36.6	No
2	5-May-09	Female	high transmission	10-Jul-09	active follow	9	36.5	No
3	5-May-09	Female	high transmission	3-Aug-09	passive follow	9		
4	5-May-09	Female	high transmission	10-Aug-09	passive follow		36.2	No
5	5-May-09	Female	high transmission	16-Aug-09	active follow			
6	5-May-09	Female	high transmission	10-Sep-09	passive follow	9	36.8	Yes
7	5-May-09	Female	high transmission	22-Sep-09	active follow			
8	5-May-09	Female	high transmission	22-Oct-09	active follow			No
9	5-May-09	Female	high transmission	10-Nov-09	passive follow	9	38.3	Yes
10	5-May-09	Female	high transmission	21-Nov-09	active follow		38.3	No
11	5-May-09	Female	high transmission	21-Dec-09	active follow			No
12	5-May-09	Female	high transmission	25-Jan-10	active follow	9	35.4	No
13	5-May-09	Female	high transmission	25-Feb-10	active follow		35.4	No
14	5-May-09	Female	high transmission	6-Mar-10	passive follow	9	35.5	Yes
15	5-May-09	Female	high transmission	23-Mar-10	passive follow	9	36.2	No
16	5-May-09	Female	high transmission	20-Apr-10	passive follow	198996	39.5	Yes
17	5-May-09	Female	high transmission	25-Apr-10	active follow	198996	35.6	No
1	5-May-09	Female	high transmission	5-Jun-09	active follow	9	36.5	No
2	5-May-09	Female	high transmission	8-Jul-09	active follow	9	36.1	No
3	5-May-09	Female	high transmission	7-Aug-09	active follow	9	36.8	No
4	5-May-09	Female	high transmission	13-Sep-09	active follow			
5	5-May-09	Female	high transmission	18-Oct-09	active follow	5209	36.3	No
6	5-May-09	Female	high transmission	27-Oct-09	passive follow	67792		
7	5-May-09	Female	high transmission	24-Nov-09	active follow	554277	36.5	No
8	5-May-09	Female	high transmission	26-Dec-09	active follow	18795	36.4	No
9	5-May-09	Female	high transmission	28-Jan-10	active follow	14296	36.4	No
10	5-May-09	Female	high transmission	28-Feb-10	active follow	270	37	No
11	5-May-09	Female	high transmission	2-Apr-10	active follow	5587	36.8	No
1	10-May-09	Male	high transmission	13-Jun-09	active follow	9	36.9	No
2	10-May-09	Male	high transmission	6-Jul-09	passive follow	9	37	Yes
3	10-May-09	Male	high transmission	20-Jul-09	active follow	9	36.3	No
4	10-May-09	Male	high transmission	4-Aug-09	passive follow	9	37.2	No
5	10-May-09	Male	high transmission	19-Aug-09	active follow	9	36.2	No
6	10-May-09	Male	high transmission	23-Sep-09	active follow	9	36.7	Yes
7	10-May-09	Male	high transmission	22-Oct-09	active follow	33161	36.1	No
8	10-May-09	Male	high transmission	11-Nov-09	passive follow	82932	36.5	Yes
9	10-May-09	Male	high transmission	22-Nov-09	active follow		36.8	No
10	10-May-09	Male	high transmission	13-Dec-09	passive follow	9	38.2	No
11	10-May-09	Male	high transmission	24-Dec-09	active follow	9	36.5	No

12	10-May-09	Male	high transmission	30-Jan-10	active follow			
13	10-May-09	Male	high transmission	10-Mar-10	active follow	9	36.2	No
14	10-May-09	Male	high transmission	7-Apr-10	active follow	9	36.1	No
15	10-May-09	Male	high transmission	4-May-10	active follow	9	36.6	No
1	10-May-09	Male	high transmission	17-Jun-09	active follow	9	36.6	No
2	10-May-09	Male	high transmission	14-Jul-09	active follow	9	36.3	No
3	10-May-09	Male	high transmission	17-Aug-09	active follow	9	36.1	No
4	10-May-09	Male	high transmission	16-Sep-09	active follow	9	36.2	No
5	10-May-09	Male	high transmission	14-Oct-09	active follow	1957	36.4	No
6	10-May-09	Male	high transmission	12-Nov-09	active follow	9	35.4	No
7	10-May-09	Male	high transmission	19-Dec-09	active follow			
8	10-May-09	Male	high transmission	8-Jan-10	passive follow	25292	35.7	Yes
9	10-May-09	Male	high transmission	25-Jan-10	active follow			
10	10-May-09	Male	high transmission	24-Feb-10	active follow	12749	36	No
11	10-May-09	Male	high transmission	23-Mar-10	active follow	1670	36.2	No
12	10-May-09	Male	high transmission	22-Apr-10	active follow	3201	36	No
1	15-May-09	Female	high transmission	22-Jun-09	active follow		36.1	No
2	15-May-09	Female	high transmission	23-Jul-09	active follow	9	36.6	No
3	15-May-09	Female	high transmission	25-Aug-09	active follow		36.5	No
4	15-May-09	Female	high transmission	25-Sep-09	active follow	30593	36.6	No
5	15-May-09	Female	high transmission	2-Nov-09	active follow	5792	35.6	No
6	15-May-09	Female	high transmission	9-Dec-09	active follow	9	36.6	No
7	15-May-09	Female	high transmission	11-Jan-10	active follow	2681	36.4	No
8	15-May-09	Female	high transmission	15-Feb-10	active follow	524	36.5	No
9	15-May-09	Female	high transmission	20-Mar-10	active follow		36.9	Yes
10	15-May-09	Female	high transmission	25-Mar-10	passive follow	512	37.3	Yes
11	15-May-09	Female	high transmission	21-Apr-10	active follow	9	36.5	No
1	19-May-09	Male	high transmission	22-Jun-09	active follow	9	36.7	No
2	19-May-09	Male	high transmission	24-Jun-09	passive follow	9	37	Yes
3	19-May-09	Male	high transmission	27-Jul-09	active follow	9	36.2	No
4	19-May-09	Male	high transmission	28-Aug-09	active follow	9	37.1	Yes
5	19-May-09	Male	high transmission	12-Sep-09	passive follow	1391	37.1	Yes
6	19-May-09	Male	high transmission	28-Sep-09	active follow	16067	35.4	No
7	19-May-09	Male	high transmission	5-Nov-09	active follow	9	36	No
8	19-May-09	Male	high transmission	17-Nov-09	passive follow	2793	37.3	Yes
9	19-May-09	Male	high transmission	8-Dec-09	active follow	19807	36.3	No
10	19-May-09	Male	high transmission	10-Jan-10	active follow	9	36.3	No
11	19-May-09	Male	high transmission	16-Feb-10	active follow	9	36.2	No
12	19-May-09	Male	high transmission	17-Mar-10	active follow	9	37	No
13	19-May-09	Male	high transmission	8-Apr-10	passive follow		37.1	No
14	19-May-09	Male	high transmission	17-Apr-10	active follow	9	36.3	No
15	19-May-09	Male	high transmission	23-Apr-10	passive follow	9	37	Yes
1	27-May-09	Male	high transmission	25-Jun-09	active follow	9	36	No
2	27-May-09	Male	high transmission	22-Jul-09	active follow	9	36.7	No
3	27-May-09	Male	high transmission	22-Aug-09	active follow	382	36.1	No
4	27-May-09	Male	high transmission	23-Sep-09	passive follow	5856	37.2	Yes
5	27-May-09	Male	high transmission	20-Oct-09	passive follow	9	36.3	Yes

6	27-May-09	Male	high transmission	26-Oct-09	active follow	9	36.3	No
7	27-May-09	Male	high transmission	25-Nov-09	passive follow	117495	37.4	No
8	27-May-09	Male	high transmission	2-Dec-09	active follow	117495	36.7	No
9	27-May-09	Male	high transmission	31-Dec-09	passive follow	9	36.3	Yes
10	27-May-09	Male	high transmission	2-Jan-10	active follow		36	No
11	27-May-09	Male	high transmission	5-Feb-10	active follow	9	36	No
12	27-May-09	Male	high transmission	17-Mar-10	active follow	9	35.6	No
13	27-May-09	Male	high transmission	31-Mar-10	passive follow	9		
14	27-May-09	Male	high transmission	1-Apr-10	passive follow		36.5	Yes
15	27-May-09	Male	high transmission	13-Apr-10	passive follow	9	36.7	Yes
16	27-May-09	Male	high transmission	14-Apr-10	active follow	9	36.1	No
17	27-May-09	Male	high transmission	15-Apr-10	active follow	9		
18	27-May-09	Male	high transmission	11-May-10	active follow	9	36	No
19	27-May-09	Male	high transmission	13-May-10	passive follow	9	39.2	Yes
1	27-May-09	Male	high transmission	16-Jun-09	passive follow	9	36	Yes
2	27-May-09	Male	high transmission	30-Jun-09	active follow	9	36	No
3	27-May-09	Male	high transmission	2-Aug-09	active follow	9	36	No
4	27-May-09	Male	high transmission	5-Sep-09	active follow	9	36.1	No
5	27-May-09	Male	high transmission	25-Sep-09	passive follow	9	37.3	Yes
6	27-May-09	Male	high transmission	12-Oct-09	active follow		36	Yes
7	27-May-09	Male	high transmission	13-Nov-09	active follow	9	36.5	No
8	27-May-09	Male	high transmission	26-Nov-09	passive follow	9	36.6	No
9	27-May-09	Male	high transmission	19-Dec-09	active follow	9	36	No
10	27-May-09	Male	high transmission	21-Dec-09	active follow	9		
11	27-May-09	Male	high transmission	23-Jan-10	active follow	2021	36.2	No
12	27-May-09	Male	high transmission	2-Feb-10	passive follow	133596	37.7	Yes
13	27-May-09	Male	high transmission	1-Mar-10	active follow			
14	27-May-09	Male	high transmission	4-Apr-10	active follow			
15	27-May-09	Male	high transmission	6-May-10	active follow			
1	4-Jun-09	Female	high transmission	2-Jul-09	active follow	9	36.5	No
2	4-Jun-09	Female	high transmission	2-Aug-09	active follow	9	36.1	No
3	4-Jun-09	Female	high transmission	5-Sep-09	active follow	9	36	No
4	4-Jun-09	Female	high transmission	23-Sep-09	passive follow	16275	37	Yes
5	4-Jun-09	Female	high transmission	9-Oct-09	active follow	2650	36	No
6	4-Jun-09	Female	high transmission	4-Nov-09	passive follow	9	36.5	No
7	4-Jun-09	Female	high transmission	13-Nov-09	active follow	9	36.4	No
8	4-Jun-09	Female	high transmission	21-Dec-09	active follow	9	35.8	No
9	4-Jun-09	Female	high transmission	24-Jan-10	active follow		36.1	No
10	4-Jun-09	Female	high transmission	26-Feb-10	active follow	15648	36.1	No
11	4-Jun-09	Female	high transmission	3-Apr-10	active follow	9	36.4	No
12	4-Jun-09	Female	high transmission	10-May-10	active follow	9	36.1	No
1	27-May-09	Female	high transmission	26-Jun-09	active follow	9	36.9	No
2	27-May-09	Female	high transmission	3-Aug-09	active follow	9	36	No
3	27-May-09	Female	high transmission	5-Sep-09	active follow	9	36.1	No
4	27-May-09	Female	high transmission	9-Oct-09	active follow	9	36.3	No
5	27-May-09	Female	high transmission	6-Nov-09	passive follow	9	36.9	Yes
6	27-May-09	Female	high transmission	16-Nov-09	active follow	9	36.3	No

7	27-May-09	Female	high transmission	22-Dec-09	active follow	796	36.3	No
8	27-May-09	Female	high transmission	25-Jan-10	active follow		36.5	No
9	27-May-09	Female	high transmission	3-Feb-10	passive follow		36.3	No
10	27-May-09	Female	high transmission	1-Mar-10	active follow	9	36	No
11	27-May-09	Female	high transmission	4-Mar-10	passive follow	1035	36.5	Yes
12	27-May-09	Female	high transmission	8-Apr-10	active follow	9	36.3	No
13	27-May-09	Female	high transmission	10-May-10	active follow	9	36.4	No
1	4-Jun-09	Female	high transmission	8-Jul-09	active follow	9	36.8	No
2	4-Jun-09	Female	high transmission	12-Aug-09	active follow	9	36.6	No
3	4-Jun-09	Female	high transmission	11-Sep-09	active follow	9	36.5	No
4	4-Jun-09	Female	high transmission	15-Oct-09	active follow	9	36.3	No
5	4-Jun-09	Female	high transmission	21-Oct-09	passive follow	9	36.5	Yes
6	4-Jun-09	Female	high transmission	11-Nov-09	active follow	9	35.7	No
7	4-Jun-09	Female	high transmission	13-Dec-09	active follow	9	36	No
8	4-Jun-09	Female	high transmission	13-Jan-10	active follow	9	36.1	No
9	4-Jun-09	Female	high transmission	12-Feb-10	active follow	9	36.3	No
10	4-Jun-09	Female	high transmission	11-Mar-10	passive follow	9	37.5	Yes
11	4-Jun-09	Female	high transmission	17-Mar-10	active follow	9	35.8	No
12	4-Jun-09	Female	high transmission	14-Apr-10	active follow	52	36	No
13	4-Jun-09	Female	high transmission	15-May-10	active follow	9	36.7	No
14	4-Jun-09	Female	high transmission	31-May-10	passive follow	320081	38.4	Yes
1	13-Jun-09	Male	high transmission	15-Jul-09	active follow	9	36.5	No
2	13-Jun-09	Male	high transmission	14-Aug-09	active follow	9	36.4	No
3	13-Jun-09	Male	high transmission	19-Sep-09	active follow	9	36.4	No
4	13-Jun-09	Male	high transmission	26-Oct-09	active follow			
5	13-Jun-09	Male	high transmission	28-Nov-09	active follow	26147	36.1	No
6	13-Jun-09	Male	high transmission	14-Dec-09	passive follow	5097	38.8	Yes
7	13-Jun-09	Male	high transmission	4-Jan-10	active follow		36.1	No
8	13-Jun-09	Male	high transmission	21-Jan-10	passive follow	5917	38.7	Yes
9	13-Jun-09	Male	high transmission	4-Feb-10	active follow	9	36	No
10	13-Jun-09	Male	high transmission	24-Feb-10	passive follow	9	36.6	Yes
11	13-Jun-09	Male	high transmission	3-Mar-10	active follow	9	36.1	No
12	13-Jun-09	Male	high transmission	9-Apr-10	active follow			
13	13-Jun-09	Male	high transmission	12-May-10	active follow	9	36.1	No
14	13-Jun-09	Male	high transmission	8-Jun-10	active follow	123764	40.3	Yes
1	15-Jun-09	Female	high transmission	17-Jul-09	active follow		36.3	No
2	15-Jun-09	Female	high transmission	10-Aug-09	passive follow	9		
3	15-Jun-09	Female	high transmission	25-Aug-09	active follow	9	36.2	No
4	15-Jun-09	Female	high transmission	23-Sep-09	active follow	754	36.5	No
5	15-Jun-09	Female	high transmission	28-Sep-09	passive follow	1368	38.5	Yes
6	15-Jun-09	Female	high transmission	8-Oct-09	passive follow		37.6	No
7	15-Jun-09	Female	high transmission	30-Oct-09	active follow	10192	36.1	No
8	15-Jun-09	Female	high transmission	4-Nov-09	passive follow	9	37.5	No
9	15-Jun-09	Female	high transmission	9-Dec-09	active follow	303	36.2	No
10	15-Jun-09	Female	high transmission	7-Jan-10	active follow		36.1	No
11	15-Jun-09	Female	high transmission	11-Feb-10	active follow	9	36.6	No
12	15-Jun-09	Female	high transmission	23-Mar-10	active follow		36.3	No

13	15-Jun-09	Female	high transmission	26-Apr-10	active follow		36.5	No
14	15-Jun-09	Female	high transmission	4-Jun-10	active follow		36.3	No
1	3-Jun-09	Male	high transmission	3-Jul-09	active follow			
2	3-Jun-09	Male	high transmission	9-Aug-09	active follow			
3	3-Jun-09	Male	high transmission	14-Sep-09	active follow	9	36.5	No
4	3-Jun-09	Male	high transmission	16-Oct-09	passive follow	9	36.5	Yes
5	3-Jun-09	Male	high transmission	21-Oct-09	active follow	9	36.2	No
6	3-Jun-09	Male	high transmission	26-Nov-09	active follow	169	36.2	No
7	3-Jun-09	Male	high transmission	29-Dec-09	active follow	9	36.4	No
8	3-Jun-09	Male	high transmission	11-Jan-10	passive follow	7264	35.8	Yes
9	3-Jun-09	Male	high transmission	2-Feb-10	active follow	9	36	No
10	3-Jun-09	Male	high transmission	11-Mar-10	active follow			
11	3-Jun-09	Male	high transmission	17-Apr-10	active follow			
12	3-Jun-09	Male	high transmission	18-May-10	active follow	9	36	No
1	10-Jun-09	Male	high transmission	14-Jul-09	active follow	9	36.3	No
2	10-Jun-09	Male	high transmission	15-Aug-09	active follow	9	36.3	No
3	10-Jun-09	Male	high transmission	21-Sep-09	active follow	9	36.3	No
4	10-Jun-09	Male	high transmission	12-Oct-09	passive follow		36.9	No
5	10-Jun-09	Male	high transmission	28-Oct-09	active follow			
6	10-Jun-09	Male	high transmission	18-Nov-09	passive follow	1153	36.8	Yes
7	10-Jun-09	Male	high transmission	1-Dec-09	active follow		36.3	No
8	10-Jun-09	Male	high transmission	2-Jan-10	active follow	9	35.6	No
9	10-Jun-09	Male	high transmission	13-Jan-10	passive follow	1186	36.5	Yes
10	10-Jun-09	Male	high transmission	6-Feb-10	active follow	6545	35.7	No
11	10-Jun-09	Male	high transmission	18-Mar-10	active follow	2340	37.6	Yes
12	10-Jun-09	Male	high transmission	2-Apr-10	passive follow	9	36.3	No
13	10-Jun-09	Male	high transmission	19-Apr-10	active follow	9	36	No
14	10-Jun-09	Male	high transmission	26-Apr-10	passive follow	112364	37	Yes
15	10-Jun-09	Male	high transmission	5-May-10	passive follow	9	36.8	No
16	10-Jun-09	Male	high transmission	19-May-10	active follow	9	36.3	No
17	10-Jun-09	Male	high transmission	31-May-10	passive follow	26027	37.7	No
1	21-Jun-09	Female	high transmission	23-Jul-09	active follow	9	36.3	No
2	21-Jun-09	Female	high transmission	27-Aug-09	active follow	9	35.8	No
3	21-Jun-09	Female	high transmission	5-Sep-09	passive follow	9	36.6	No
4	21-Jun-09	Female	high transmission	26-Sep-09	active follow	9	36.5	No
5	21-Jun-09	Female	high transmission	4-Oct-09	passive follow	1503	39.9	Yes
6	21-Jun-09	Female	high transmission	2-Nov-09	active follow	9	36.5	No
7	21-Jun-09	Female	high transmission	11-Nov-09	passive follow	13036	36.5	No
8	21-Jun-09	Female	high transmission	25-Nov-09	passive follow	334	35.8	No
9	21-Jun-09	Female	high transmission	9-Dec-09	active follow	1292	36.4	No
10	21-Jun-09	Female	high transmission	16-Dec-09	passive follow	1527	36.5	No
11	21-Jun-09	Female	high transmission	8-Jan-10	active follow	5016	36.7	No
12	21-Jun-09	Female	high transmission	9-Jan-10	passive follow	60905	36.4	Yes
13	21-Jun-09	Female	high transmission	12-Feb-10	active follow	9	36.8	No
14	21-Jun-09	Female	high transmission	19-Mar-10	active follow	9	36.6	No
15	21-Jun-09	Female	high transmission	19-Apr-10	passive follow		36.6	No
16	21-Jun-09	Female	high transmission	25-Apr-10	active follow			

17	21-Jun-09	Female	high transmission	26-May-10	active follow	2090	36.5	No
1	21-Jun-09	Male	high transmission	27-Jul-09	active follow	9	36.2	No
2	21-Jun-09	Male	high transmission	31-Aug-09	active follow	9	36.3	No
3	21-Jun-09	Male	high transmission	6-Oct-09	active follow	9	36.5	No
4	21-Jun-09	Male	high transmission	9-Nov-09	active follow	9	36.5	No
5	21-Jun-09	Male	high transmission	2-Dec-09	passive follow	137508	39	Yes
6	21-Jun-09	Male	high transmission	15-Dec-09	active follow	524	36.1	No
7	21-Jun-09	Male	high transmission	18-Jan-10	active follow	782	36	No
8	21-Jun-09	Male	high transmission	15-Feb-10	passive follow	1848	36.5	Yes
9	21-Jun-09	Male	high transmission	20-Feb-10	active follow	9	36	Yes
10	21-Jun-09	Male	high transmission	29-Mar-10	active follow	9	36	No
11	21-Jun-09	Male	high transmission	26-Apr-10	active follow	9	38.4	Yes
12	21-Jun-09	Male	high transmission	3-May-10	passive follow	9	36.8	Yes
13	21-Jun-09	Male	high transmission	27-May-10	active follow	9	36.2	Yes
14	21-Jun-09	Male	high transmission	10-Jun-10	passive follow	6172	36.8	Yes
1	28-Jun-09	Female	high transmission	3-Aug-09	active follow	9	36	No
2	28-Jun-09	Female	high transmission	3-Sep-09	active follow	9	36.6	No
3	28-Jun-09	Female	high transmission	1-Oct-09	active follow		36.2	No
4	28-Jun-09	Female	high transmission	27-Oct-09	active follow	58888	36	No
5	28-Jun-09	Female	high transmission	28-Nov-09	active follow	29363	36.4	No
6	28-Jun-09	Female	high transmission	2-Dec-09	passive follow	23261	37.6	Yes
7	28-Jun-09	Female	high transmission	31-Dec-09	active follow	9	35.8	No
8	28-Jun-09	Female	high transmission	26-Jan-10	active follow	9	36	No
9	28-Jun-09	Female	high transmission	23-Feb-10	active follow	9	36	No
10	28-Jun-09	Female	high transmission	23-Mar-10	active follow	9	36.4	No
11	28-Jun-09	Female	high transmission	22-Apr-10	active follow	9	36.4	No
12	28-Jun-09	Female	high transmission	21-May-10	active follow		36.1	No
13	28-Jun-09	Female	high transmission	23-Jun-10	active follow	78	35.8	No
1	12-Jul-09	Female	high transmission	13-Aug-09	active follow	9	36.6	No
2	12-Jul-09	Female	high transmission	19-Aug-09	passive follow	9	37.8	Yes
3	12-Jul-09	Female	high transmission	10-Sep-09	active follow	9	36.3	No
4	12-Jul-09	Female	high transmission	8-Oct-09	active follow	9	36	No
5	12-Jul-09	Female	high transmission	5-Nov-09	active follow	6349	36.8	No
6	12-Jul-09	Female	high transmission	1-Dec-09	passive follow			
7	12-Jul-09	Female	high transmission	11-Dec-09	active follow	9	37.1	Yes
8	12-Jul-09	Female	high transmission	23-Dec-09	passive follow	5421		
9	12-Jul-09	Female	high transmission	14-Jan-10	active follow	10641	36	No
10	12-Jul-09	Female	high transmission	10-Feb-10	passive follow	25319	38	Yes
11	12-Jul-09	Female	high transmission	15-Feb-10	active follow	2866	36.3	No
12	12-Jul-09	Female	high transmission	10-Mar-10	passive follow	3934		
13	12-Jul-09	Female	high transmission	21-Mar-10	active follow	17256	36.2	No
14	12-Jul-09	Female	high transmission	26-Mar-10	passive follow		37.1	Yes
15	12-Jul-09	Female	high transmission	22-Apr-10	active follow	7363	36.5	No
16	12-Jul-09	Female	high transmission	1-May-10	passive follow	629	37.4	No
17	12-Jul-09	Female	high transmission	29-May-10	active follow			
18	12-Jul-09	Female	high transmission	5-Jul-10	active follow			
1	17-Jul-09	Male	high transmission	27-Aug-09	active follow	9	36	No

2	17-Jul-09	Male	high transmission	23-Sep-09	active follow	9	36	No
3	17-Jul-09	Male	high transmission	30-Oct-09	active follow	4565	37.2	Yes
4	17-Jul-09	Male	high transmission	9-Dec-09	active follow	359	36.3	No
5	17-Jul-09	Male	high transmission	5-Jan-10	active follow	850	36.1	No
6	17-Jul-09	Male	high transmission	11-Feb-10	active follow	4797	36	No
7	17-Jul-09	Male	high transmission	10-Mar-10	passive follow	185	37.8	Yes
8	17-Jul-09	Male	high transmission	22-Mar-10	active follow		36.9	Yes
9	17-Jul-09	Male	high transmission	28-Apr-10	active follow	9	36.1	No
10	17-Jul-09	Male	high transmission	2-Jun-10	active follow	9	36.4	No
11	17-Jul-09	Male	high transmission	5-Jul-10	active follow	9	36.1	No
1	28-Jul-09	Female	high transmission	31-Aug-09	active follow	9	36.8	No
2	28-Jul-09	Female	high transmission	6-Oct-09	active follow	9	36.7	No
3	28-Jul-09	Female	high transmission	19-Oct-09	passive follow	9	37.4	Yes
4	28-Jul-09	Female	high transmission	3-Nov-09	active follow	9	35.3	No
5	28-Jul-09	Female	high transmission	5-Dec-09	active follow	79887	35.7	No
6	28-Jul-09	Female	high transmission	18-Dec-09	passive follow	50354	37.3	Yes
7	28-Jul-09	Female	high transmission	5-Jan-10	passive follow		37.4	Yes
8	28-Jul-09	Female	high transmission	9-Jan-10	active follow	9	36.3	No
9	28-Jul-09	Female	high transmission	14-Jan-10	passive follow	9	36	Yes
10	28-Jul-09	Female	high transmission	13-Feb-10	active follow	9	35.5	No
11	28-Jul-09	Female	high transmission	22-Mar-10	active follow	69581	36	No
12	28-Jul-09	Female	high transmission	29-Mar-10	passive follow	432	37.2	Yes
13	28-Jul-09	Female	high transmission	26-Apr-10	active follow	432	36.4	No
14	28-Jul-09	Female	high transmission	29-Apr-10	passive follow	9	37.2	Yes
15	28-Jul-09	Female	high transmission	13-May-10	passive follow	9		
16	28-Jul-09	Female	high transmission	26-May-10	active follow	9	36	No
17	28-Jul-09	Female	high transmission	14-Jun-10	passive follow	9	36.5	Yes
18	28-Jul-09	Female	high transmission	24-Jun-10	active follow	9	36.3	No
19	28-Jul-09	Female	high transmission	21-Jul-10	active follow	9	36.4	No
1	28-Jul-09	Male	high transmission	27-Aug-09	active follow	9	36.7	No
2	28-Jul-09	Male	high transmission	31-Aug-09	passive follow	9	36.6	Yes
3	28-Jul-09	Male	high transmission	25-Sep-09	passive follow	9	37.1	Yes
4	28-Jul-09	Male	high transmission	26-Sep-09	active follow		36.9	Yes
5	28-Jul-09	Male	high transmission	21-Oct-09	passive follow		37	Yes
6	28-Jul-09	Male	high transmission	29-Oct-09	active follow	9	37.3	No
7	28-Jul-09	Male	high transmission	30-Nov-09	active follow	9	36.9	No
8	28-Jul-09	Male	high transmission	23-Dec-09	passive follow	9	38.4	Yes
9	28-Jul-09	Male	high transmission	4-Jan-10	active follow	9	36.5	No
10	28-Jul-09	Male	high transmission	6-Feb-10	active follow	9	36	No
11	28-Jul-09	Male	high transmission	11-Mar-10	passive follow	9	35.9	Yes
12	28-Jul-09	Male	high transmission	15-Mar-10	active follow		36	No
13	28-Jul-09	Male	high transmission	16-Apr-10	active follow	9	36.6	No
14	28-Jul-09	Male	high transmission	22-Apr-10	passive follow	9	37.6	Yes
15	28-Jul-09	Male	high transmission	19-May-10	active follow	9	37.3	Yes
16	28-Jul-09	Male	high transmission	7-Jun-10	passive follow	124460	39.3	Yes
17	28-Jul-09	Male	high transmission	11-Jun-10	passive follow	73688	36.8	Yes
18	28-Jul-09	Male	high transmission	17-Jun-10	active follow		36	No

19	28-Jul-09	Male	high transmission	10-Jul-10	active follow	37157	35.5	No
1	21-Jul-09	Female	high transmission	4-Aug-09	passive follow		36.9	No
2	21-Jul-09	Female	high transmission	27-Aug-09	active follow	9	36.2	No
3	21-Jul-09	Female	high transmission	3-Sep-09	passive follow	9	36	No
4	21-Jul-09	Female	high transmission	24-Sep-09	active follow	9	36.5	No
5	21-Jul-09	Female	high transmission	26-Oct-09	passive follow	9	36.3	No
6	21-Jul-09	Female	high transmission	31-Oct-09	active follow	9	36.5	No
7	21-Jul-09	Female	high transmission	24-Nov-09	passive follow	9	37	No
8	21-Jul-09	Female	high transmission	6-Dec-09	active follow	9	36.4	No
9	21-Jul-09	Female	high transmission	8-Jan-10	active follow	9	36.5	No
10	21-Jul-09	Female	high transmission	12-Jan-10	passive follow	9		
11	21-Jul-09	Female	high transmission	13-Feb-10	active follow	2202	37.2	No
12	21-Jul-09	Female	high transmission	15-Feb-10	passive follow	3740	36.2	No
13	21-Jul-09	Female	high transmission	14-Mar-10	passive follow	9		
14	21-Jul-09	Female	high transmission	18-Mar-10	active follow	1404	36.6	No
15	21-Jul-09	Female	high transmission	24-Mar-10	passive follow	9	36.1	No
16	21-Jul-09	Female	high transmission	14-Apr-10	passive follow	9	36.9	No
17	21-Jul-09	Female	high transmission	20-Apr-10	active follow	9	36.8	No
18	21-Jul-09	Female	high transmission	12-May-10	passive follow	9	37.4	No
19	21-Jul-09	Female	high transmission	18-May-10	passive follow	9	37.4	Yes
20	21-Jul-09	Female	high transmission	1-Jun-10	active follow	9	37.3	No
21	21-Jul-09	Female	high transmission	17-Jun-10	passive follow	736	39.3	Yes
22	21-Jul-09	Female	high transmission	29-Jun-10	active follow	9	36.5	No
1	4-Aug-09	Male	high transmission	31-Aug-09	passive follow	9		
2	4-Aug-09	Male	high transmission	4-Sep-09	active follow	9	36.7	No
3	4-Aug-09	Male	high transmission	9-Oct-09	active follow	9	37.6	Yes
4	4-Aug-09	Male	high transmission	12-Nov-09	active follow	9	35.3	No
5	4-Aug-09	Male	high transmission	9-Dec-09	active follow	9	35.7	No
6	4-Aug-09	Male	high transmission	6-Jan-10	active follow	9	36.1	No
7	4-Aug-09	Male	high transmission	6-Feb-10	active follow	9	36.3	No
8	4-Aug-09	Male	high transmission	10-Feb-10	passive follow	9		
9	4-Aug-09	Male	high transmission	14-Mar-10	active follow	9	36	No
10	4-Aug-09	Male	high transmission	7-Apr-10	passive follow	9	36.5	Yes
11	4-Aug-09	Male	high transmission	15-Apr-10	active follow	9	36.3	No
12	4-Aug-09	Male	high transmission	11-May-10	active follow	9	36.2	No
13	4-Aug-09	Male	high transmission	2-Jun-10	passive follow	9		
14	4-Aug-09	Male	high transmission	4-Jun-10	passive follow		35.6	No
15	4-Aug-09	Male	high transmission	9-Jun-10	active follow	733	36.1	No
16	4-Aug-09	Male	high transmission	16-Jun-10	passive follow			No
17	4-Aug-09	Male	high transmission	7-Jul-10	active follow	9423		No
18	4-Aug-09	Male	high transmission	20-Jul-10	passive follow	6134	35.7	Yes
1	11-Aug-09	Male	high transmission	11-Sep-09	active follow	9	36.9	No
2	11-Aug-09	Male	high transmission	21-Sep-09	passive follow	9		
3	11-Aug-09	Male	high transmission	5-Oct-09	passive follow	9		
4	11-Aug-09	Male	high transmission	18-Oct-09	active follow	9	36.6	No
5	11-Aug-09	Male	high transmission	23-Nov-09	active follow	163	36.2	No
6	11-Aug-09	Male	high transmission	17-Dec-09	passive follow	113026	38.1	Yes

7	11-Aug-09	Male	high transmission	28-Dec-09	active follow		36.5	No
8	11-Aug-09	Male	high transmission	26-Jan-10	active follow	1800	35.5	No
9	11-Aug-09	Male	high transmission	24-Feb-10	active follow	16982	36.8	No
10	11-Aug-09	Male	high transmission	26-Feb-10	passive follow	8480	37.4	Yes
11	11-Aug-09	Male	high transmission	22-Mar-10	active follow	9	36.4	No
12	11-Aug-09	Male	high transmission	30-Mar-10	passive follow		37.4	Yes
13	11-Aug-09	Male	high transmission	22-Apr-10	active follow	3565	36.9	No
14	11-Aug-09	Male	high transmission	24-May-10	active follow	346	36.4	No
15	11-Aug-09	Male	high transmission	3-Jun-10	passive follow	154804	36.8	Yes
16	11-Aug-09	Male	high transmission	7-Jun-10	passive follow	323684	37.6	Yes
17	11-Aug-09	Male	high transmission	30-Jun-10	active follow		36.4	No
18	11-Aug-09	Male	high transmission	2-Aug-10	active follow		35.4	No
1	11-Aug-09	Female	high transmission	12-Sep-09	active follow	9	36.2	No
2	11-Aug-09	Female	high transmission	13-Oct-09	active follow	9	36.5	No
3	11-Aug-09	Female	high transmission	9-Nov-09	passive follow	9	37.2	Yes
4	11-Aug-09	Female	high transmission	16-Nov-09	active follow	511	36.6	No
5	11-Aug-09	Female	high transmission	18-Dec-09	passive follow	10461	36.2	No
6	11-Aug-09	Female	high transmission	29-Dec-09	active follow	9		
7	11-Aug-09	Female	high transmission	22-Jan-10	active follow	9	36.5	No
8	11-Aug-09	Female	high transmission	8-Feb-10	passive follow	9	37.7	Yes
9	11-Aug-09	Female	high transmission	27-Feb-10	active follow	9	36.3	No
10	11-Aug-09	Female	high transmission	3-Apr-10	active follow	9	36.6	No
11	11-Aug-09	Female	high transmission	24-Apr-10	passive follow	9	36.7	Yes
12	11-Aug-09	Female	high transmission	10-May-10	active follow	9	36.5	No
13	11-Aug-09	Female	high transmission	9-Jun-10	active follow	882	36	No
14	11-Aug-09	Female	high transmission	13-Jun-10	passive follow	630	36.6	Yes
15	11-Aug-09	Female	high transmission	10-Jul-10	active follow	9	36	No
16	11-Aug-09	Female	high transmission	5-Aug-10	passive follow	55438	39.3	Yes
1	17-Aug-09	Male	high transmission	21-Sep-09	active follow	9	36.8	No
2	17-Aug-09	Male	high transmission	27-Oct-09	active follow			
3	17-Aug-09	Male	high transmission	28-Nov-09	active follow		36	No
4	17-Aug-09	Male	high transmission	15-Dec-09	passive follow	9	36.3	Yes
5	17-Aug-09	Male	high transmission	2-Jan-10	active follow	9	36	No
6	17-Aug-09	Male	high transmission	7-Feb-10	active follow		36	No
7	17-Aug-09	Male	high transmission	15-Mar-10	active follow	9	36.5	No
8	17-Aug-09	Male	high transmission	5-Apr-10	passive follow	462	36.1	Yes
9	17-Aug-09	Male	high transmission	20-Apr-10	active follow	2172	36.2	No
10	17-Aug-09	Male	high transmission	23-May-10	active follow	9	36.4	No
11	17-Aug-09	Male	high transmission	28-May-10	passive follow	9	36.8	No
12	17-Aug-09	Male	high transmission	3-Jun-10	passive follow	234	36.4	No
13	17-Aug-09	Male	high transmission	21-Jun-10	active follow	9	35.8	No
14	17-Aug-09	Male	high transmission	20-Jul-10	active follow	9	35.6	No
1	18-Aug-09	Male	high transmission	16-Sep-09	active follow	9	36.5	No
2	18-Aug-09	Male	high transmission	15-Oct-09	active follow	9	36.6	No
3	18-Aug-09	Male	high transmission	20-Oct-09	passive follow	9		
4	18-Aug-09	Male	high transmission	11-Nov-09	active follow	9	36.3	No
5	18-Aug-09	Male	high transmission	15-Dec-09	active follow	9	36.6	No

6	18-Aug-09	Male	high transmission	29-Dec-09	passive follow		37.8	Yes
7	18-Aug-09	Male	high transmission	13-Jan-10	active follow		37.1	No
8	18-Aug-09	Male	high transmission	17-Jan-10	passive follow	474672		
9	18-Aug-09	Male	high transmission	15-Feb-10	active follow	9	36.2	No
10	18-Aug-09	Male	high transmission	1-Mar-10	passive follow	9	38.1	Yes
11	18-Aug-09	Male	high transmission	17-Mar-10	active follow	9	36.3	No
12	18-Aug-09	Male	high transmission	31-Mar-10	passive follow	45422	37.7	Yes
13	18-Aug-09	Male	high transmission	14-Apr-10	active follow	10206	38.1	Yes
14	18-Aug-09	Male	high transmission	17-May-10	active follow	29396	36.8	No
15	18-Aug-09	Male	high transmission	31-May-10	passive follow		40	Yes
16	18-Aug-09	Male	high transmission	16-Jun-10	active follow	287121	37.4	No
17	18-Aug-09	Male	high transmission	14-Jul-10	active follow	9	36	No
18	18-Aug-09	Male	high transmission	13-Aug-10	active follow	21413	36.1	No
1	20-Aug-09	Male	high transmission	16-Sep-09	active follow	9	36.3	No
2	20-Aug-09	Male	high transmission	16-Oct-09	active follow		37	No
3	20-Aug-09	Male	high transmission	18-Nov-09	active follow	9	36.7	No
4	20-Aug-09	Male	high transmission	25-Dec-09	active follow			No
5	20-Aug-09	Male	high transmission	4-Jan-10	passive follow	9	36.6	Yes
6	20-Aug-09	Male	high transmission	24-Jan-10	active follow			No
7	20-Aug-09	Male	high transmission	23-Feb-10	active follow			No
8	20-Aug-09	Male	high transmission	27-Feb-10	passive follow	9	37.1	Yes
9	20-Aug-09	Male	high transmission	18-Mar-10	passive follow	9	36.1	Yes
10	20-Aug-09	Male	high transmission	22-Mar-10	active follow	9	36.2	No
11	20-Aug-09	Male	high transmission	19-Apr-10	active follow	13225	37.5	Yes
12	20-Aug-09	Male	high transmission	19-May-10	passive follow		36.8	Yes
13	20-Aug-09	Male	high transmission	28-May-10	passive follow	9	36.7	No
14	20-Aug-09	Male	high transmission	16-Jun-10	active follow	822	36.4	No
15	20-Aug-09	Male	high transmission	22-Jun-10	passive follow	9	36.2	No
16	20-Aug-09	Male	high transmission	14-Jul-10	active follow	922	36	No
17	20-Aug-09	Male	high transmission	30-Jul-10	passive follow	3402	36.5	Yes
18	20-Aug-09	Male	high transmission	11-Aug-10	active follow	9	36.4	No
1	29-Aug-09	Female	high transmission	30-Sep-09	active follow	9	36.4	No
2	29-Aug-09	Female	high transmission	31-Oct-09	active follow	9	36.6	No
3	29-Aug-09	Female	high transmission	4-Dec-09	passive follow	2423	37.7	Yes
4	29-Aug-09	Female	high transmission	9-Jan-10	active follow	9	36.2	No
5	29-Aug-09	Female	high transmission	15-Feb-10	active follow			
6	29-Aug-09	Female	high transmission	16-Mar-10	active follow	9	35.8	Yes
7	29-Aug-09	Female	high transmission	3-Apr-10	passive follow	103817	38.9	Yes
8	29-Aug-09	Female	high transmission	20-Apr-10	active follow		36.5	No
9	29-Aug-09	Female	high transmission	25-Apr-10	passive follow	346541	37.8	Yes
10	29-Aug-09	Female	high transmission	26-May-10	passive follow	204004	38.2	Yes
11	29-Aug-09	Female	high transmission	27-May-10	active follow		36.8	No
12	29-Aug-09	Female	high transmission	24-Jun-10	active follow	26260	36.2	No
13	29-Aug-09	Female	high transmission	21-Jul-10	active follow	9440	36.6	No
14	29-Aug-09	Female	high transmission	19-Aug-10	active follow	1487	36.2	No
1	28-Aug-09	Female	high transmission	3-Oct-09	active follow	9	36.8	No
2	28-Aug-09	Female	high transmission	9-Nov-09	active follow	9	36.6	No

3	28-Aug-09	Female	high transmission	15-Dec-09	active follow	2249	36.8	No
4	28-Aug-09	Female	high transmission	21-Dec-09	passive follow	9	37.9	Yes
5	28-Aug-09	Female	high transmission	15-Jan-10	active follow	9	36.3	No
6	28-Aug-09	Female	high transmission	27-Jan-10	passive follow	9	36.5	No
7	28-Aug-09	Female	high transmission	18-Feb-10	active follow	9	36.6	No
8	28-Aug-09	Female	high transmission	1-Mar-10	passive follow	9	36	No
9	28-Aug-09	Female	high transmission	24-Mar-10	active follow	9	36.2	No
10	28-Aug-09	Female	high transmission	8-Apr-10	passive follow	98514	36.3	Yes
11	28-Aug-09	Female	high transmission	23-Apr-10	active follow			
12	28-Aug-09	Female	high transmission	26-May-10	active follow			
13	28-Aug-09	Female	high transmission	25-Jun-10	active follow		36.1	Yes
14	28-Aug-09	Female	high transmission	25-Jul-10	active follow			
15	28-Aug-09	Female	high transmission	10-Aug-10	passive follow	402973	39	Yes
1	3-Sep-09	Female	high transmission	2-Oct-09	active follow	9	36.8	No
2	3-Sep-09	Female	high transmission	7-Nov-09	active follow	9	36.5	No
3	3-Sep-09	Female	high transmission	15-Dec-09	active follow	9	36.2	No
4	3-Sep-09	Female	high transmission	20-Jan-10	active follow	9	36.5	No
5	3-Sep-09	Female	high transmission	18-Feb-10	active follow	9	36.1	No
6	3-Sep-09	Female	high transmission	22-Feb-10	passive follow	9	36.5	Yes
7	3-Sep-09	Female	high transmission	19-Mar-10	active follow	9	35.8	No
8	3-Sep-09	Female	high transmission	12-Apr-10	passive follow	9	37.8	Yes
9	3-Sep-09	Female	high transmission	28-Apr-10	active follow	9	36.5	No
10	3-Sep-09	Female	high transmission	25-May-10	active follow		36.4	No
11	3-Sep-09	Female	high transmission	7-Jun-10	passive follow	9	36.9	No
12	3-Sep-09	Female	high transmission	25-Jun-10	active follow	640	36.2	No
13	3-Sep-09	Female	high transmission	30-Jun-10	passive follow	9	38.1	Yes
14	3-Sep-09	Female	high transmission	29-Jul-10	active follow	9	36.1	No
15	3-Sep-09	Female	high transmission	28-Aug-10	active follow	2153	36.2	No
16	3-Sep-09	Female	high transmission	2-Sep-10	passive follow	4115	37.1	Yes
1	2-Sep-09	Female	high transmission	29-Sep-09	active follow	9	36.5	No
2	2-Sep-09	Female	high transmission	29-Oct-09	active follow	9	36	No
3	2-Sep-09	Female	high transmission	9-Nov-09	passive follow	9	36.8	Yes
4	2-Sep-09	Female	high transmission	26-Nov-09	active follow		36.3	No
5	2-Sep-09	Female	high transmission	24-Dec-09	active follow	9	36.2	No
6	2-Sep-09	Female	high transmission	8-Jan-10	passive follow	9	35.3	Yes
7	2-Sep-09	Female	high transmission	23-Jan-10	active follow	9	36	No
8	2-Sep-09	Female	high transmission	17-Feb-10	passive follow	9	35.4	Yes
9	2-Sep-09	Female	high transmission	26-Feb-10	active follow	9	36.1	No
10	2-Sep-09	Female	high transmission	18-Mar-10	passive follow	9	36.6	Yes
11	2-Sep-09	Female	high transmission	3-Apr-10	active follow	9	36	No
12	2-Sep-09	Female	high transmission	21-Apr-10	passive follow	9	36.8	No
13	2-Sep-09	Female	high transmission	5-May-10	active follow	9	36	No
14	2-Sep-09	Female	high transmission	6-May-10	passive follow	9	36.4	No
15	2-Sep-09	Female	high transmission	18-May-10	passive follow	9	36	Yes
16	2-Sep-09	Female	high transmission	27-May-10	passive follow	9		
17	2-Sep-09	Female	high transmission	2-Jun-10	active follow	9	36	No
18	2-Sep-09	Female	high transmission	9-Jun-10	passive follow	534		

19	2-Sep-09	Female	high transmission	30-Jun-10	active follow	9	36	No
20	2-Sep-09	Female	high transmission	3-Jul-10	passive follow	10144	36.3	No
21	2-Sep-09	Female	high transmission	15-Jul-10	passive follow	9	36	No
22	2-Sep-09	Female	high transmission	28-Jul-10	active follow	7874	36	No
23	2-Sep-09	Female	high transmission	29-Jul-10	passive follow		38.3	Yes
24	2-Sep-09	Female	high transmission	25-Aug-10	active follow	9	36	No
26	2-Sep-09	Female	high transmission	2-Sep-10	passive follow	9	36.7	Yes
1	5-Sep-09	Female	high transmission	8-Oct-09	passive follow	9	37	Yes
2	5-Sep-09	Female	high transmission	19-Oct-09	passive follow	9		
3	5-Sep-09	Female	high transmission	6-Nov-09	active follow	9	36.5	No
4	5-Sep-09	Female	high transmission	10-Dec-09	active follow	9	36.6	No
5	5-Sep-09	Female	high transmission	7-Jan-10	active follow	9		
6	5-Sep-09	Female	high transmission	8-Jan-10	active follow	9	36	No
7	5-Sep-09	Female	high transmission	10-Jan-10	passive follow	9	36.3	Yes
8	5-Sep-09	Female	high transmission	12-Feb-10	active follow		36.5	No
9	5-Sep-09	Female	high transmission	19-Mar-10	active follow	9	36	No
10	5-Sep-09	Female	high transmission	26-Apr-10	active follow	9	36.5	No
11	5-Sep-09	Female	high transmission	25-May-10	active follow		36.8	No
12	5-Sep-09	Female	high transmission	4-Jun-10	passive follow	9	36.5	Yes
13	5-Sep-09	Female	high transmission	25-Jun-10	active follow	62	36.2	No
14	5-Sep-09	Female	high transmission	1-Jul-10	passive follow		37.6	Yes
15	5-Sep-09	Female	high transmission	1-Aug-10	active follow	203021	40.3	Yes
16	5-Sep-09	Female	high transmission	2-Sep-10	active follow	6903	36.1	No
17	5-Sep-09	Female	high transmission	5-Sep-10	passive follow	101986	37.2	Yes
1	5-Sep-09	Female	high transmission	7-Oct-09	active follow	9	36.3	No
2	5-Sep-09	Female	high transmission	16-Oct-09	passive follow	9		
3	5-Sep-09	Female	high transmission	9-Nov-09	active follow	624	36.4	No
4	5-Sep-09	Female	high transmission	23-Nov-09	passive follow	4096	36.8	No
5	5-Sep-09	Female	high transmission	7-Dec-09	passive follow	9	36.6	Yes
6	5-Sep-09	Female	high transmission	10-Dec-09	active follow	9	36.5	No
7	5-Sep-09	Female	high transmission	8-Jan-10	active follow	9	36.4	No
8	5-Sep-09	Female	high transmission	12-Feb-10	active follow	9	36.4	No
9	5-Sep-09	Female	high transmission	3-Mar-10	passive follow	9	35.9	Yes
10	5-Sep-09	Female	high transmission	18-Mar-10	active follow	9	36.8	No
11	5-Sep-09	Female	high transmission	12-Apr-10	active follow	9	36.8	Yes
12	5-Sep-09	Female	high transmission	11-May-10	active follow	9	36.7	No
13	5-Sep-09	Female	high transmission	17-Jun-10	active follow	9	36.5	No
14	5-Sep-09	Female	high transmission	12-Jul-10	active follow	40029	37.8	Yes
15	5-Sep-09	Female	high transmission	11-Aug-10	active follow	9	36.4	No
1	10-Sep-09	Female	high transmission	9-Oct-09	active follow	9	36.5	No
2	10-Sep-09	Female	high transmission	15-Nov-09	active follow			
3	10-Sep-09	Female	high transmission	20-Dec-09	active follow	9	36.4	No
4	10-Sep-09	Female	high transmission	7-Jan-10	passive follow		39.1	Yes
5	10-Sep-09	Female	high transmission	22-Jan-10	active follow	9	36.6	No
6	10-Sep-09	Female	high transmission	25-Feb-10	active follow	9	36	No
7	10-Sep-09	Female	high transmission	23-Mar-10	active follow	9	36.8	No
8	10-Sep-09	Female	high transmission	22-Apr-10	active follow	9	36.5	Yes

9	10-Sep-09	Female	high transmission	22-May-10	active follow			
10	10-Sep-09	Female	high transmission	28-May-10	passive follow	158541	40.1	Yes
11	10-Sep-09	Female	high transmission	23-Jun-10	active follow	18713	36.3	No
12	10-Sep-09	Female	high transmission	21-Jul-10	active follow	5976	36.2	No
13	10-Sep-09	Female	high transmission	20-Aug-10	active follow	43698	37	No
1	7-Sep-09	Male	high transmission	10-Oct-09	active follow	9	36.8	No
2	7-Sep-09	Male	high transmission	20-Oct-09	passive follow		36.4	No
3	7-Sep-09	Male	high transmission	12-Nov-09	active follow	9	36.6	No
4	7-Sep-09	Male	high transmission	24-Nov-09	passive follow		36.7	No
5	7-Sep-09	Male	high transmission	14-Dec-09	active follow	9	36.2	No
6	7-Sep-09	Male	high transmission	15-Jan-10	active follow	9	36.3	No
7	7-Sep-09	Male	high transmission	7-Feb-10	passive follow		36.5	No
8	7-Sep-09	Male	high transmission	20-Feb-10	active follow	9	36.6	No
9	7-Sep-09	Male	high transmission	23-Feb-10	passive follow	9		
10	7-Sep-09	Male	high transmission	25-Mar-10	active follow	9	36.6	No
11	7-Sep-09	Male	high transmission	10-Apr-10	passive follow	4821	36.5	Yes
12	7-Sep-09	Male	high transmission	22-Apr-10	active follow		36.8	No
13	7-Sep-09	Male	high transmission	23-May-10	active follow	166687	36.6	No
14	7-Sep-09	Male	high transmission	24-May-10	passive follow		39.8	Yes
15	7-Sep-09	Male	high transmission	28-Jun-10	active follow	9	36.4	No
16	7-Sep-09	Male	high transmission	2-Aug-10	active follow			
17	7-Sep-09	Male	high transmission	5-Sep-10	active follow			
1	8-Sep-09	Male	high transmission	10-Oct-09	active follow	9	37.4	No
2	8-Sep-09	Male	high transmission	28-Oct-09	passive follow	9	36.5	Yes
3	8-Sep-09	Male	high transmission	12-Nov-09	active follow	9	36.3	No
4	8-Sep-09	Male	high transmission	17-Dec-09	active follow	9	35.9	No
5	8-Sep-09	Male	high transmission	15-Jan-10	active follow	9	36.5	No
6	8-Sep-09	Male	high transmission	24-Jan-10	passive follow	9	37.2	Yes
7	8-Sep-09	Male	high transmission	18-Feb-10	active follow	9	36.3	No
8	8-Sep-09	Male	high transmission	16-Mar-10	active follow	9	35.8	No
9	8-Sep-09	Male	high transmission	15-Apr-10	active follow	9	35.8	No
10	8-Sep-09	Male	high transmission	19-May-10	active follow	9	36.9	Yes
11	8-Sep-09	Male	high transmission	22-Jun-10	active follow	3967	36.4	No
12	8-Sep-09	Male	high transmission	23-Jun-10	passive follow		38.7	Yes
13	8-Sep-09	Male	high transmission	22-Jul-10	active follow	8201	35.7	No
14	8-Sep-09	Male	high transmission	31-Jul-10	passive follow	13867	35.7	Yes
15	8-Sep-09	Male	high transmission	28-Aug-10	active follow			
1	13-Sep-09	Female	high transmission	17-Oct-09	active follow	9	36.8	No
2	13-Sep-09	Female	high transmission	2-Nov-09	passive follow	9	37	Yes
3	13-Sep-09	Female	high transmission	24-Nov-09	active follow	9	36.6	No
4	13-Sep-09	Female	high transmission	31-Dec-09	active follow	9	36.5	No
5	13-Sep-09	Female	high transmission	3-Feb-10	active follow	9	36.3	No
6	13-Sep-09	Female	high transmission	3-Mar-10	active follow	9	36.3	No
7	13-Sep-09	Female	high transmission	9-Apr-10	active follow			
8	13-Sep-09	Female	high transmission	24-May-10	active follow	9	36.5	Yes
9	13-Sep-09	Female	high transmission	25-Jun-10	active follow	1497	36.5	No
10	13-Sep-09	Female	high transmission	29-Jul-10	active follow	9	36.8	No

11	13-Sep-09	Female	high transmission	17-Aug-10	passive follow	579836	36.2	Yes
12	13-Sep-09	Female	high transmission	25-Aug-10	active follow		36.6	No
1	9-Sep-09	Male	high transmission	30-Sep-09	passive follow	9	36.3	No
2	9-Sep-09	Male	high transmission	9-Oct-09	active follow	9	36.3	No
3	9-Sep-09	Male	high transmission	24-Oct-09	passive follow	9	36.5	No
4	9-Sep-09	Male	high transmission	13-Nov-09	active follow	9	36.3	No
5	9-Sep-09	Male	high transmission	1-Dec-09	passive follow		36.1	No
6	9-Sep-09	Male	high transmission	19-Dec-09	active follow	3469	36	No
7	9-Sep-09	Male	high transmission	1-Jan-10	passive follow	9	36.5	Yes
8	9-Sep-09	Male	high transmission	28-Jan-10	active follow	176	36.5	No
9	9-Sep-09	Male	high transmission	14-Feb-10	passive follow			No
10	9-Sep-09	Male	high transmission	26-Feb-10	active follow	1245	35.5	No
11	9-Sep-09	Male	high transmission	6-Apr-10	active follow	424220	39.1	Yes
12	9-Sep-09	Male	high transmission	28-Apr-10	passive follow	9	38	Yes
13	9-Sep-09	Male	high transmission	15-May-10	active follow	9	36.2	No
14	9-Sep-09	Male	high transmission	25-May-10	passive follow	9	36.7	Yes
15	9-Sep-09	Male	high transmission	15-Jun-10	active follow	9	36.9	No
16	9-Sep-09	Male	high transmission	28-Jun-10	passive follow	4224	36.1	No
17	9-Sep-09	Male	high transmission	3-Jul-10	passive follow	55485	35.7	Yes
18	9-Sep-09	Male	high transmission	14-Jul-10	active follow	9	35.7	No
19	9-Sep-09	Male	high transmission	10-Aug-10	active follow	9	35.7	No
20	9-Sep-09	Male	high transmission	18-Aug-10	passive follow	9	35.7	No
1	15-Sep-09	Female	high transmission	14-Oct-09	active follow	9	36.3	No
2	15-Sep-09	Female	high transmission	3-Nov-09	passive follow	9	36.3	No
3	15-Sep-09	Female	high transmission	14-Nov-09	active follow	9	36.6	No
4	15-Sep-09	Female	high transmission	20-Dec-09	active follow	27492	35.8	No
5	15-Sep-09	Female	high transmission	22-Jan-10	active follow	17976	36.2	No
6	15-Sep-09	Female	high transmission	25-Feb-10	active follow	9	36.2	No
7	15-Sep-09	Female	high transmission	23-Mar-10	active follow	9	36.5	No
8	15-Sep-09	Female	high transmission	22-Apr-10	active follow	2961	36.3	No
9	15-Sep-09	Female	high transmission	30-Apr-10	passive follow	56702	37.7	Yes
10	15-Sep-09	Female	high transmission	29-May-10	active follow			
11	15-Sep-09	Female	high transmission	5-Jul-10	active follow			
12	15-Sep-09	Female	high transmission	26-Jul-10	passive follow	198603	39.7	Yes
13	15-Sep-09	Female	high transmission	8-Aug-10	active follow	9	36.4	No
14	15-Sep-09	Female	high transmission	8-Sep-10	active follow	9	37.9	No
1	16-Sep-09	Male	high transmission	15-Oct-09	active follow	9	36.8	No
2	16-Sep-09	Male	high transmission	26-Oct-09	passive follow	9		
3	16-Sep-09	Male	high transmission	14-Nov-09	active follow	29	36.4	No
4	16-Sep-09	Male	high transmission	22-Dec-09	active follow	1407	36.4	No
5	16-Sep-09	Male	high transmission	25-Jan-10	active follow	451	36.4	No
6	16-Sep-09	Male	high transmission	26-Feb-10	active follow	5916	35.9	No
7	16-Sep-09	Male	high transmission	8-Apr-10	active follow	9	36.4	No
8	16-Sep-09	Male	high transmission	16-Apr-10	passive follow	3967	37.1	Yes
9	16-Sep-09	Male	high transmission	7-May-10	active follow		38	Yes
10	16-Sep-09	Male	high transmission	24-May-10	passive follow		36.8	Yes
11	16-Sep-09	Male	high transmission	14-Jun-10	active follow	3829	36	No

12	16-Sep-09	Male	high transmission	13-Jul-10	active follow	9	36.2	No
13	16-Sep-09	Male	high transmission	3-Aug-10	passive follow	260992	37.1	Yes
14	16-Sep-09	Male	high transmission	18-Aug-10	active follow	9	36.1	No
1	17-Sep-09	Male	high transmission	19-Oct-09	active follow	9	36.8	No
2	17-Sep-09	Male	high transmission	17-Nov-09	passive follow	9		
3	17-Sep-09	Male	high transmission	24-Nov-09	active follow	159	35.5	No
4	17-Sep-09	Male	high transmission	9-Dec-09	passive follow	1005	36	Yes
5	17-Sep-09	Male	high transmission	27-Dec-09	active follow	9	36.1	No
6	17-Sep-09	Male	high transmission	9-Jan-10	passive follow	3257	35.6	Yes
7	17-Sep-09	Male	high transmission	26-Jan-10	active follow	9	35.6	No
8	17-Sep-09	Male	high transmission	11-Feb-10	passive follow	43489	37.9	Yes
9	17-Sep-09	Male	high transmission	26-Feb-10	active follow	9	36.8	No
10	17-Sep-09	Male	high transmission	5-Mar-10	passive follow	9	36.6	No
11	17-Sep-09	Male	high transmission	4-Apr-10	active follow	9	36	No
12	17-Sep-09	Male	high transmission	21-Apr-10	passive follow	9	36.5	Yes
13	17-Sep-09	Male	high transmission	6-May-10	active follow	9	36.6	No
14	17-Sep-09	Male	high transmission	12-Jun-10	passive follow	146928	38.1	Yes
15	17-Sep-09	Male	high transmission	14-Jun-10	passive follow	1077	36.1	Yes
16	17-Sep-09	Male	high transmission	22-Jun-10	passive follow	9	37.3	Yes
17	17-Sep-09	Male	high transmission	24-Jun-10	active follow	9	36.2	No
18	17-Sep-09	Male	high transmission	26-Jul-10	active follow	26769	39.4	Yes
19	17-Sep-09	Male	high transmission	9-Aug-10	passive follow	9	37.1	Yes
20	17-Sep-09	Male	high transmission	1-Sep-10	active follow	9	36	No
1	23-Sep-09	Male	high transmission	22-Oct-09	active follow	9	36.2	No
2	23-Sep-09	Male	high transmission	5-Nov-09	passive follow	9	38	Yes
3	23-Sep-09	Male	high transmission	22-Nov-09	active follow	9	35.6	No
4	23-Sep-09	Male	high transmission	24-Dec-09	active follow	9	36	No
5	23-Sep-09	Male	high transmission	23-Jan-10	active follow	9	36.2	No
6	23-Sep-09	Male	high transmission	27-Feb-10	active follow	9	36.5	No
7	23-Sep-09	Male	high transmission	29-Mar-10	passive follow	9156	37.7	Yes
8	23-Sep-09	Male	high transmission	26-Apr-10	active follow	9	36.5	No
9	23-Sep-09	Male	high transmission	1-May-10	passive follow	101409	37.7	Yes
10	23-Sep-09	Male	high transmission	25-May-10	active follow	9	36.1	No
11	23-Sep-09	Male	high transmission	22-Jun-10	active follow	9	36.6	No
12	23-Sep-09	Male	high transmission	6-Jul-10	passive follow	9	36.2	No
13	23-Sep-09	Male	high transmission	31-Jul-10	active follow	5233	36	No
14	23-Sep-09	Male	high transmission	1-Aug-10	active follow	5233		
15	23-Sep-09	Male	high transmission	26-Aug-10	passive follow	36842	38.6	Yes
16	23-Sep-09	Male	high transmission	17-Sep-10	active follow	55	36	No
1	24-Sep-09	Male	high transmission	22-Oct-09	active follow	9	36.3	No
2	24-Sep-09	Male	high transmission	17-Nov-09	active follow	9	36.3	No
3	24-Sep-09	Male	high transmission	17-Dec-09	active follow	9	36	No
4	24-Sep-09	Male	high transmission	13-Jan-10	active follow	9	36	No
5	24-Sep-09	Male	high transmission	13-Feb-10	active follow	9	36.3	No
6	24-Sep-09	Male	high transmission	17-Mar-10	active follow	9	36.3	No
7	24-Sep-09	Male	high transmission	16-Apr-10	active follow	9	36	No
8	24-Sep-09	Male	high transmission	19-May-10	active follow		36.5	No

9	24-Sep-09	Male	high transmission	14-Jun-10	active follow	9	36.5	No
10	24-Sep-09	Male	high transmission	14-Jul-10	active follow	7504	36	No
11	24-Sep-09	Male	high transmission	18-Aug-10	active follow	9186	36.8	No
12	24-Sep-09	Male	high transmission	30-Aug-10	passive follow	13320	37.2	Yes
13	24-Sep-09	Male	high transmission	19-Sep-10	active follow	9	36.2	No
1	27-Sep-09	Male	high transmission	29-Oct-09	active follow	9	36.6	No
2	27-Sep-09	Male	high transmission	26-Nov-09	active follow	9	36.3	No
3	27-Sep-09	Male	high transmission	25-Dec-09	active follow	9	35.5	No
4	27-Sep-09	Male	high transmission	18-Jan-10	passive follow	577	37.4	Yes
5	27-Sep-09	Male	high transmission	28-Jan-10	active follow	9	36.1	No
6	27-Sep-09	Male	high transmission	28-Feb-10	active follow	9	36.2	No
7	27-Sep-09	Male	high transmission	18-Mar-10	passive follow	9		
8	27-Sep-09	Male	high transmission	6-Apr-10	active follow	9	36	No
9	27-Sep-09	Male	high transmission	14-Apr-10	passive follow	9	39.1	Yes
10	27-Sep-09	Male	high transmission	5-May-10	active follow	9	36.3	No
11	27-Sep-09	Male	high transmission	14-May-10	passive follow	9	36.9	Yes
12	27-Sep-09	Male	high transmission	2-Jun-10	active follow	9	36	No
13	27-Sep-09	Male	high transmission	19-Jun-10	passive follow	34120	36.5	Yes
14	27-Sep-09	Male	high transmission	30-Jun-10	active follow	9	36	No
15	27-Sep-09	Male	high transmission	16-Jul-10	passive follow	119266	39.6	Yes
16	27-Sep-09	Male	high transmission	28-Jul-10	active follow		36	No
17	27-Sep-09	Male	high transmission	28-Aug-10	active follow	210	36	No
18	27-Sep-09	Male	high transmission	12-Sep-10	passive follow	129191	38.4	Yes
1	28-Sep-09	Female	high transmission	24-Oct-09	passive follow	9	37.1	No
2	28-Sep-09	Female	high transmission	3-Nov-09	active follow	9	36.8	No
3	28-Sep-09	Female	high transmission	10-Dec-09	active follow	9	36.5	No
4	28-Sep-09	Female	high transmission	11-Jan-10	active follow	9	36.7	No
5	28-Sep-09	Female	high transmission	29-Jan-10	passive follow	9		
6	28-Sep-09	Female	high transmission	13-Feb-10	active follow	9	36.3	No
7	28-Sep-09	Female	high transmission	19-Mar-10	active follow	9	36.6	No
8	28-Sep-09	Female	high transmission	2-Apr-10	passive follow	7488	38.7	Yes
9	28-Sep-09	Female	high transmission	26-Apr-10	active follow	99	36.3	No
10	28-Sep-09	Female	high transmission	21-May-10	passive follow	1380	37.1	No
11	28-Sep-09	Female	high transmission	5-Jun-10	active follow	3935	36.6	No
12	28-Sep-09	Female	high transmission	12-Jul-10	active follow	9	36.2	No
13	28-Sep-09	Female	high transmission	13-Aug-10	active follow	9	36.2	No
14	28-Sep-09	Female	high transmission	14-Sep-10	active follow		36.7	No
15	28-Sep-09	Female	high transmission	26-Sep-10	passive follow	10669	40	Yes
1	25-Sep-09	Female	high transmission	23-Oct-09	passive follow		37.1	No
2	25-Sep-09	Female	high transmission	1-Nov-09	active follow			
3	25-Sep-09	Female	high transmission	6-Dec-09	active follow	5160	35.4	No
4	25-Sep-09	Female	high transmission	1-Jan-10	passive follow	9	36.6	Yes
5	25-Sep-09	Female	high transmission	11-Jan-10	active follow	5519	36.8	No
6	25-Sep-09	Female	high transmission	15-Feb-10	active follow	20882	36.2	No
7	25-Sep-09	Female	high transmission	23-Feb-10	passive follow		36.7	Yes
8	25-Sep-09	Female	high transmission	27-Feb-10	passive follow	9039		
9	25-Sep-09	Female	high transmission	15-Mar-10	passive follow	8652	38.3	Yes

10	25-Sep-09	Female	high transmission	20-Mar-10	active follow		36.5	No
11	25-Sep-09	Female	high transmission	30-Mar-10	passive follow	13474	38.3	Yes
12	25-Sep-09	Female	high transmission	28-Apr-10	active follow	16350	36.7	No
13	25-Sep-09	Female	high transmission	4-Jun-10	active follow			
14	25-Sep-09	Female	high transmission	29-Jun-10	passive follow		38.9	Yes
15	25-Sep-09	Female	high transmission	2-Jul-10	passive follow		38.5	Yes
16	25-Sep-09	Female	high transmission	8-Jul-10	active follow		36.4	No
17	25-Sep-09	Female	high transmission	26-Jul-10	passive follow	19153	38.1	Yes
18	25-Sep-09	Female	high transmission	8-Aug-10	active follow	9	36.4	No
19	25-Sep-09	Female	high transmission	13-Sep-10	passive follow		38.9	Yes
20	25-Sep-09	Female	high transmission	14-Sep-10	active follow	19692	38.2	Yes
21	25-Sep-09	Female	high transmission	23-Sep-10	passive follow	9	35.7	Yes
1	25-Sep-09	Female	high transmission	11-Oct-09	passive follow		36.5	No
2	25-Sep-09	Female	high transmission	19-Oct-09	passive follow	9		
3	25-Sep-09	Female	high transmission	19-Oct-09	passive follow		37	Yes
4	25-Sep-09	Female	high transmission	1-Nov-09	active follow		36.5	No
5	25-Sep-09	Female	high transmission	1-Nov-09	active follow			
6	25-Sep-09	Female	high transmission	6-Dec-09	active follow			
7	25-Sep-09	Female	high transmission	6-Dec-09	active follow	9	36	No
8	25-Sep-09	Female	high transmission	18-Dec-09	passive follow	9	36.4	No
9	25-Sep-09	Female	high transmission	10-Jan-10	active follow			
10	25-Sep-09	Female	high transmission	10-Jan-10	active follow	9	36	No
11	25-Sep-09	Female	high transmission	13-Feb-10	active follow	9	36.6	No
12	25-Sep-09	Female	high transmission	13-Feb-10	active follow			
13	25-Sep-09	Female	high transmission	21-Mar-10	active follow	9	36.5	No
14	25-Sep-09	Female	high transmission	21-Mar-10	active follow			
15	25-Sep-09	Female	high transmission	6-Apr-10	passive follow	9	37.9	Yes
16	25-Sep-09	Female	high transmission	27-Apr-10	active follow			
17	25-Sep-09	Female	high transmission	27-Apr-10	active follow			
18	25-Sep-09	Female	high transmission	7-May-10	passive follow	18589	37.6	Yes
19	25-Sep-09	Female	high transmission	30-May-10	active follow		36.7	No
20	25-Sep-09	Female	high transmission	30-May-10	active follow			
21	25-Sep-09	Female	high transmission	29-Jun-10	active follow	594	36.6	Yes
22	25-Sep-09	Female	high transmission	1-Jul-10	active follow			
23	25-Sep-09	Female	high transmission	29-Jul-10	active follow			
24	25-Sep-09	Female	high transmission	29-Jul-10	active follow	14985	36.5	No
25	25-Sep-09	Female	high transmission	30-Jul-10	passive follow		38.4	Yes
26	25-Sep-09	Female	high transmission	26-Aug-10	active follow			
27	25-Sep-09	Female	high transmission	26-Aug-10	active follow	9	36	No
28	25-Sep-09	Female	high transmission	31-Aug-10	passive follow	9	36.2	Yes
29	25-Sep-09	Female	high transmission	23-Sep-10	active follow			
30	25-Sep-09	Female	high transmission	23-Sep-10	active follow	9	36.5	No
1	5-Oct-09	Male	high transmission	7-Nov-09	active follow	9	36.6	No
2	5-Oct-09	Male	high transmission	9-Dec-09	passive follow	9		
3	5-Oct-09	Male	high transmission	15-Dec-09	active follow	9	36.4	No
4	5-Oct-09	Male	high transmission	13-Jan-10	active follow	9	35.9	No
5	5-Oct-09	Male	high transmission	12-Feb-10	active follow	9	36	No

6	5-Oct-09	Male	high transmission	28-Feb-10	passive follow	9	36	Yes
7	5-Oct-09	Male	high transmission	17-Mar-10	active follow	90	36.5	No
8	5-Oct-09	Male	high transmission	20-Mar-10	passive follow	9	38	Yes
9	5-Oct-09	Male	high transmission	14-Apr-10	active follow	9	36	No
10	5-Oct-09	Male	high transmission	16-Apr-10	passive follow	9	37.4	Yes
11	5-Oct-09	Male	high transmission	15-May-10	active follow	9	36.8	No
12	5-Oct-09	Male	high transmission	2-Jun-10	passive follow		36.2	No
13	5-Oct-09	Male	high transmission	16-Jun-10	active follow	9	36.8	No
14	5-Oct-09	Male	high transmission	29-Jun-10	passive follow	15926	37	Yes
15	5-Oct-09	Male	high transmission	7-Jul-10	passive follow	9	36.6	No
16	5-Oct-09	Male	high transmission	21-Jul-10	active follow	9	35.7	No
17	5-Oct-09	Male	high transmission	28-Jul-10	passive follow	137424	38.4	Yes
18	5-Oct-09	Male	high transmission	16-Aug-10	active follow	9	39.8	Yes
19	5-Oct-09	Male	high transmission	14-Sep-10	active follow	9	36	No
1	7-Oct-09	Male	high transmission	7-Nov-09	active follow	9	36.8	Yes
2	7-Oct-09	Male	high transmission	1-Dec-09	passive follow	9	36.6	Yes
3	7-Oct-09	Male	high transmission	14-Dec-09	active follow	9	36.6	No
4	7-Oct-09	Male	high transmission	18-Jan-10	passive follow	9	36	No
5	7-Oct-09	Male	high transmission	18-Feb-10	active follow	9	36.3	No
6	7-Oct-09	Male	high transmission	25-Mar-10	active follow		36.6	No
7	7-Oct-09	Male	high transmission	24-Apr-10	active follow	9	36.2	No
8	7-Oct-09	Male	high transmission	26-May-10	active follow	9	36.3	No
9	7-Oct-09	Male	high transmission	11-Jun-10	passive follow	9		
10	7-Oct-09	Male	high transmission	24-Jun-10	active follow	45389	37.7	Yes
11	7-Oct-09	Male	high transmission	26-Jul-10	active follow	4461	36	No
12	7-Oct-09	Male	high transmission	27-Aug-10	active follow	1643	35.9	No
13	7-Oct-09	Male	high transmission	1-Sep-10	passive follow	9		
14	7-Oct-09	Male	high transmission	28-Sep-10	active follow	3168	36.4	No
1	18-Oct-09	Female	high transmission	23-Nov-09	active follow	9	36.6	No
2	18-Oct-09	Female	high transmission	30-Nov-09	passive follow	9		
3	18-Oct-09	Female	high transmission	24-Dec-09	passive follow	9		
4	18-Oct-09	Female	high transmission	28-Dec-09	active follow	9	36	No
5	18-Oct-09	Female	high transmission	2-Feb-10	active follow	9	36.3	No
6	18-Oct-09	Female	high transmission	18-Feb-10	passive follow	9	35.9	No
7	18-Oct-09	Female	high transmission	9-Mar-10	active follow	9	36.3	Yes
8	18-Oct-09	Female	high transmission	12-Apr-10	active follow	9	36.6	No
9	18-Oct-09	Female	high transmission	21-Apr-10	passive follow	9		
10	18-Oct-09	Female	high transmission	10-May-10	active follow	9	38	Yes
11	18-Oct-09	Female	high transmission	27-May-10	passive follow	9	36.7	Yes
12	18-Oct-09	Female	high transmission	9-Jun-10	active follow	9	36.7	No
13	18-Oct-09	Female	high transmission	21-Jun-10	passive follow	9	36.6	No
14	18-Oct-09	Female	high transmission	6-Jul-10	active follow	9	36.6	No
15	18-Oct-09	Female	high transmission	8-Aug-10	active follow	9	36	No
16	18-Oct-09	Female	high transmission	24-Aug-10	passive follow	1064		
17	18-Oct-09	Female	high transmission	1-Sep-10	passive follow	1231		
18	18-Oct-09	Female	high transmission	13-Sep-10	active follow	11022	36.5	No
19	18-Oct-09	Female	high transmission	3-Oct-10	passive follow	9	36.5	Yes

20	18-Oct-09	Female	high transmission	9-Oct-10	active follow	9	40.4	Yes
1	22-Oct-09	Male	high transmission	17-Nov-09	active follow	9	36.1	No
2	22-Oct-09	Male	high transmission	27-Nov-09	passive follow	9	36.8	Yes
3	22-Oct-09	Male	high transmission	4-Dec-09	passive follow	9	37	No
4	22-Oct-09	Male	high transmission	18-Dec-09	active follow	9	36.4	No
5	22-Oct-09	Male	high transmission	21-Jan-10	active follow	9	36.9	Yes
6	22-Oct-09	Male	high transmission	19-Feb-10	active follow	9	36.4	No
7	22-Oct-09	Male	high transmission	27-Mar-10	active follow	9	36.6	No
8	22-Oct-09	Male	high transmission	15-Apr-10	passive follow		36.5	Yes
9	22-Oct-09	Male	high transmission	1-May-10	active follow		36.1	No
10	22-Oct-09	Male	high transmission	21-May-10	passive follow	9	36.5	No
11	22-Oct-09	Male	high transmission	5-Jun-10	active follow	772	36.4	No
12	22-Oct-09	Male	high transmission	7-Jul-10	active follow	1755	36.4	No
13	22-Oct-09	Male	high transmission	14-Jul-10	passive follow	20842	36.2	Yes
14	22-Oct-09	Male	high transmission	6-Aug-10	active follow	9	36	No
15	22-Oct-09	Male	high transmission	14-Aug-10	passive follow	8476	37.7	Yes
16	22-Oct-09	Male	high transmission	12-Sep-10	active follow	9	37.6	Yes
17	22-Oct-09	Male	high transmission	13-Oct-10	active follow		37.8	Yes
1	21-Oct-09	Female	high transmission	18-Nov-09	active follow	9	36.5	No
2	21-Oct-09	Female	high transmission	19-Dec-09	active follow	9	35.6	No
3	21-Oct-09	Female	high transmission	25-Jan-10	active follow	9	36.7	No
4	21-Oct-09	Female	high transmission	23-Feb-10	active follow	9	36	No
5	21-Oct-09	Female	high transmission	24-Mar-10	active follow	9	36	No
6	21-Oct-09	Female	high transmission	26-Apr-10	active follow	9	36.1	No
7	21-Oct-09	Female	high transmission	24-May-10	active follow	3107	37.5	Yes
8	21-Oct-09	Female	high transmission	31-May-10	passive follow	9	36.5	No
9	21-Oct-09	Female	high transmission	23-Jun-10	active follow	9	36.4	No
10	21-Oct-09	Female	high transmission	5-Jul-10	passive follow	9	36.8	Yes
11	21-Oct-09	Female	high transmission	21-Jul-10	active follow	9	35.9	No
12	21-Oct-09	Female	high transmission	22-Jul-10	passive follow	9	35.6	No
13	21-Oct-09	Female	high transmission	9-Aug-10	passive follow	9	37	No
14	21-Oct-09	Female	high transmission	18-Aug-10	active follow	9	36.4	No
15	21-Oct-09	Female	high transmission	26-Aug-10	passive follow	9	36.5	No
16	21-Oct-09	Female	high transmission	14-Sep-10	active follow	9	35.4	No
17	21-Oct-09	Female	high transmission	12-Oct-10	active follow	858	36	No
1	22-Oct-09	Female	high transmission	2-Nov-09	passive follow	9		
2	22-Oct-09	Female	high transmission	19-Nov-09	active follow		35.6	No
3	22-Oct-09	Female	high transmission	23-Dec-09	active follow	9	36.4	No
4	22-Oct-09	Female	high transmission	23-Jan-10	active follow		36.4	No
5	22-Oct-09	Female	high transmission	8-Feb-10	passive follow	9	36.7	Yes
6	22-Oct-09	Female	high transmission	1-Mar-10	active follow	9	36.7	No
7	22-Oct-09	Female	high transmission	8-Apr-10	active follow	9	36.8	Yes
8	22-Oct-09	Female	high transmission	17-May-10	active follow	9	37	Yes
9	22-Oct-09	Female	high transmission	29-May-10	passive follow	9	36.6	No
10	22-Oct-09	Female	high transmission	22-Jun-10	active follow	16523	36.7	No
11	22-Oct-09	Female	high transmission	4-Jul-10	passive follow	6918	36.5	Yes
12	22-Oct-09	Female	high transmission	26-Jul-10	active follow	9662	37.3	No

13	22-Oct-09	Female	high transmission	1-Sep-10	active follow			
14	22-Oct-09	Female	high transmission	8-Oct-10	active follow			
1	29-Oct-09	Male	high transmission	25-Nov-09	active follow	9	36.5	No
2	29-Oct-09	Male	high transmission	24-Dec-09	active follow	9	36.3	No
3	29-Oct-09	Male	high transmission	21-Jan-10	active follow	129	36.4	No
4	29-Oct-09	Male	high transmission	18-Feb-10	active follow	9	36.2	No
5	29-Oct-09	Male	high transmission	20-Mar-10	active follow	368	36.6	Yes
6	29-Oct-09	Male	high transmission	3-Apr-10	passive follow	9	39.2	Yes
7	29-Oct-09	Male	high transmission	27-Apr-10	active follow	9	36.1	No
8	29-Oct-09	Male	high transmission	14-May-10	passive follow	9	36	No
9	29-Oct-09	Male	high transmission	3-Jun-10	active follow			
10	29-Oct-09	Male	high transmission	10-Jul-10	active follow			
11	29-Oct-09	Male	high transmission	16-Aug-10	active follow			
12	29-Oct-09	Male	high transmission	22-Sep-10	active follow			
13	29-Oct-09	Male	high transmission	22-Oct-10	active follow	9	36	No
1	17-Oct-09	Female	high transmission	3-Nov-09	passive follow		37.3	No
2	17-Oct-09	Female	high transmission	21-Nov-09	active follow		37.1	No
3	17-Oct-09	Female	high transmission	27-Dec-09	active follow		35.8	No
4	17-Oct-09	Female	high transmission	13-Jan-10	passive follow	9	35.8	No
5	17-Oct-09	Female	high transmission	1-Feb-10	active follow	9	36	No
6	17-Oct-09	Female	high transmission	19-Feb-10	passive follow	9	36.4	No
7	17-Oct-09	Female	high transmission	9-Mar-10	active follow	9	36	No
8	17-Oct-09	Female	high transmission	22-Mar-10	passive follow	104814	40.1	Yes
9	17-Oct-09	Female	high transmission	14-Apr-10	active follow	9	36.2	No
10	17-Oct-09	Female	high transmission	10-May-10	passive follow	3605	36	Yes
11	17-Oct-09	Female	high transmission	20-May-10	active follow	9	36	No
12	17-Oct-09	Female	high transmission	28-Jun-10	active follow	80476	36	Yes
13	17-Oct-09	Female	high transmission	5-Aug-10	active follow	14512	36.1	No
14	17-Oct-09	Female	high transmission	14-Sep-10	active follow	9	36	No
15	17-Oct-09	Female	high transmission	28-Sep-10	passive follow	9		
1	30-Oct-09	Male	high transmission	2-Dec-09	active follow	9	36.5	No
2	30-Oct-09	Male	high transmission	30-Dec-09	active follow	9	36.6	No
3	30-Oct-09	Male	high transmission	27-Jan-10	active follow	9	36.6	No
4	30-Oct-09	Male	high transmission	5-Mar-10	active follow			
5	30-Oct-09	Male	high transmission	8-Apr-10	active follow	9	36.1	Yes
6	30-Oct-09	Male	high transmission	7-May-10	active follow	9	36.4	Yes
7	30-Oct-09	Male	high transmission	14-Jun-10	active follow	9	36.7	Yes
8	30-Oct-09	Male	high transmission	15-Jul-10	active follow	9	36.3	No
9	30-Oct-09	Male	high transmission	22-Jul-10	passive follow	113511	36.2	Yes
10	30-Oct-09	Male	high transmission	12-Aug-10	active follow	9	36.2	No
11	30-Oct-09	Male	high transmission	28-Aug-10	passive follow	76024	37.9	Yes
12	30-Oct-09	Male	high transmission	14-Sep-10	active follow		36.8	No
13	30-Oct-09	Male	high transmission	7-Oct-10	active follow	30047	37.2	No
1	26-Oct-09	Female	high transmission	26-Nov-09	active follow	9	36.4	No
2	26-Oct-09	Female	high transmission	26-Dec-09	active follow	9	36.4	No
3	26-Oct-09	Female	high transmission	27-Jan-10	active follow	9	36.1	No
4	26-Oct-09	Female	high transmission	26-Feb-10	active follow	9	36	No

5	26-Oct-09	Female	high transmission	1-Apr-10	active follow	9	36	No
6	26-Oct-09	Female	high transmission	12-Apr-10	passive follow	5863	36	Yes
7	26-Oct-09	Female	high transmission	10-May-10	active follow	9	36	No
8	26-Oct-09	Female	high transmission	14-Jun-10	active follow	9	35.9	No
9	26-Oct-09	Female	high transmission	16-Jul-10	active follow	7438	36	No
10	26-Oct-09	Female	high transmission	22-Aug-10	active follow			
11	26-Oct-09	Female	high transmission	23-Sep-10	active follow	3121	35.3	No
12	26-Oct-09	Female	high transmission	19-Oct-10	active follow	9	37.1	Yes
